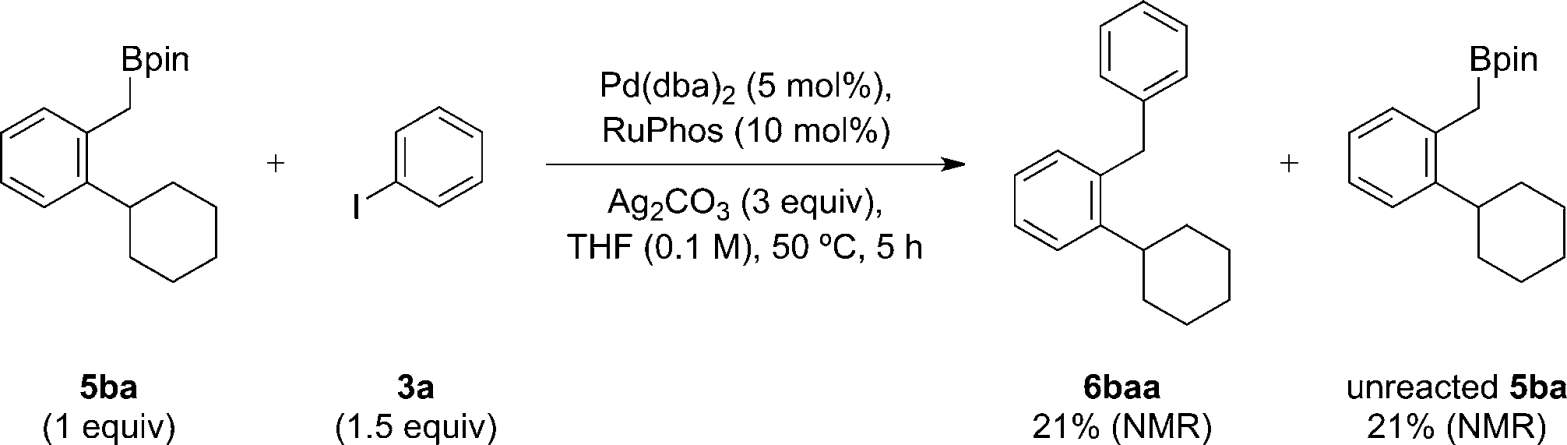# Enantiospecific Synthesis of *ortho*‐Substituted 1,1‐Diarylalkanes by a 1,2‐Metalate Rearrangement/*anti*‐S_*N*_2′ Elimination/Rearomatizing Allylic Suzuki–Miyaura Reaction Sequence

**DOI:** 10.1002/anie.201811343

**Published:** 2018-12-21

**Authors:** Belén Rubial, Beatrice S. L. Collins, Raphael Bigler, Stefan Aichhorn, Adam Noble, Varinder K. Aggarwal

**Affiliations:** ^1^ School of Chemistry University of Bristol Cantock's Close Bristol BS8 1TS UK

**Keywords:** 1,1-diarylalkane, boronic ester, cross-coupling, one-pot, stereospecific

## Abstract

The one‐pot sequential coupling of benzylamines, boronic esters, and aryl iodides has been investigated. In the presence of an N‐activator, the boronate complex formed from an *ortho*‐lithiated benzylamine and a boronic ester undergoes stereospecific 1,2‐metalate rearrangement/anti‐S_N_2′ elimination to form a dearomatized tertiary boronic ester. Treatment with an aryl iodide under palladium catalysis leads to rearomatizing γ‐selective allylic Suzuki–Miyaura cross‐coupling to generate 1,1‐diarylalkanes. When enantioenriched α‐substituted benzylamines are employed, the corresponding 1,1‐diarylalkanes are formed with high stereospecificity.

The 1,1‐diarylalkane motif is found in many biologically relevant molecules and, as a result, approaches to its stereocontrolled synthesis have garnered considerable attention in recent years.^1^ A remarkably diverse array of reactivity platforms has been developed for its synthesis, including the decarbonylation of β,β‐diarylpropionaldehydes,^2^ the hydrogenation of 1,1‐diarylalkenes,^3^ and the difunctionalization of both alkyl‐ and aryl‐substituted alkenes.^4^ A more convergent strategy is the Ni‐catalyzed cross‐coupling of benzylic electrophiles, through both enantiospecific^5^ and enantioconvergent^6^ pathways. Alternatively, benzylic nucleophiles, such as boron reagents, can be used. For example, Crudden has described the stereospecific Pd‐catalyzed cross‐coupling of chiral secondary boronic esters with aryl iodides (Scheme 1 A).^7^ Accessing the required secondary benzylic boronic esters through the asymmetric rhodium‐catalyzed hydroboration of styrene derivatives,^8^ this method proceeds with good levels of enantio‐retention to provide the desired 1,1‐diarylethane derivatives. While all these methods together provide a broad reactivity platform to access 1,1‐diarylalkanes, limitations remain with respect to substrate scope, where many methods are restricted to naphthyl‐based or sterically unencumbered substrates.

**Scheme 1:**
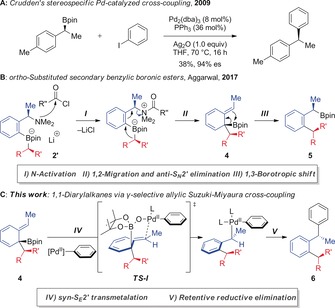
Access to 1,1‐diarylalkanes.

We recently reported a method for the enantiospecific synthesis of *ortho*‐substituted secondary benzylic boronic esters.^9^ Enantioenriched α‐methyl *o*‐bromo benzylamines were transformed into dearomatized intermediate **4** through a 1,2‐metalate rearrangement/*anti*‐S_*N*_2′ elimination reaction triggered by *N*‐activation of arylboronate complex **2′** (Scheme 1 B). Subsequent suprafacial 1,3‐borotropic shift provided the secondary α‐methyl benzylic boronic esters (**5**) with excellent levels of enantiopurity. We recognized that the stereospecific cross‐coupling of these enantioenriched benzylic boronic esters with an aryl electrophile, in line with reports from Crudden,^7^ would provide access to the valuable 1,1‐diarylalkane motif.^10^ A more direct route to such motifs, however, would be through the interruption of the cascade sequence at the dearomatized intermediate **4**, engaging this species in a rearomatizing γ‐selective allylic Suzuki–Miyaura cross‐coupling (Scheme 1 C).^11^ We envisioned that such a pathway, passing through a six‐membered ring transition state, **TS‐I**, would allow transfer of the chiral information in **4** and provide a route to enantioenriched 1,1‐diarylalkanes with extensive functionalization in the *ortho* position. Herein, we report the realization of this process, which proceeds through two consecutive stereospecific 1,3‐transpositions of stereogenicity, including a 1,2‐metalate rearrangement/*anti*‐S_*N*_2′ elimination and a *syn*‐S_*E*_2′ γ‐selective Suzuki–Miyaura reaction, to provide a one pot procedure to transform enantioenriched α‐branched benzylamines into enantioenriched 1,1‐diarylalkanes bearing considerable steric congestion in the *ortho* position.

We began our studies with dearomatized tertiary boronic ester **4 aa**, which was chosen because it can be isolated by column chromatography (see Supporting Information for details) and can be accessed through our previously reported 1,2‐metalate rearrangement/*anti*‐S_*N*_2′ elimination reaction. After optimization (see Supporting Information for details), cross‐coupled product **6 aaa** was formed in 98 % ^1^H NMR yield (Scheme 2 A). We then undertook optimization of the one‐pot procedure. Dearomatized tertiary boronic ester **4 aa** was generated by successive treatment of *ortho*‐bromo naphthylamine **1 a** with *n*BuLi, to form *ortho*‐lithiated naphthylamine; cyclohexylboronic acid pinacol ester (CyBpin, **2 a**), giving the arylboronate complex; and the *N*‐activator, Me_2_Troc‐Cl, to promote 1,2‐metalate rearrangement/*anti*‐S_*N*_2′ elimination. The reaction mixture was then treated with Ag_2_O, followed by Pd(dba)_2_, RuPhos, and iodobenzene (**3 a**) and heated to 75 °C for 6 h. While some of the desired product **6 aaa** was observed, the yield was considerably lower (8 %) than that obtained when using isolated **4 aa**. Pleasingly, changing the silver salt from Ag_2_O to Ag_2_CO_3_ and optimizing the stoichiometry led to a significant improvement in yield (90 %). Furthermore, reducing the temperature from 75 °C to 50 °C had no detrimental effect on the yield, providing **6 aaa** in 92 % yield as determined by ^1^H NMR (Scheme 2 B). Interestingly, the ^1^H NMR spectrum of the purified material contained two sets of signals in a ratio of 87:13, which were shown to interconvert through variable temperature ^1^H NMR experiments. We identified a coalescence temperature of 55 °C and determined a rate of exchange from the major to the minor species of 30.9 Hz and a rotational barrier of 17.0 kcal mol^−1^.^12^ The rate of exchange from the minor to the major species was determined to be 207.1 Hz and the rotational barrier 15.8 kcal mol^−1^ (see Supporting Information for details). In combination with two‐dimensional EXSY/NOESY NMR experiments, these studies led us to assign the two sets of signals as rotamers, **6 aaa‐*R***_***A***_ and **6 aaa‐*R***_***B***_, where interconversion occurs through rotation of the naphthyl‐cyclohexyl C−C bond (Scheme 2 C).^13^ Furthermore, NOESY correlations support the assignment of the major rotamer as **6 aaa‐*R***_***A***_. Having identified the two sets of signals as rotamers, we were then able to confirm that **6 aaa** was isolated in 88 % yield.^14^

**Scheme 2:**
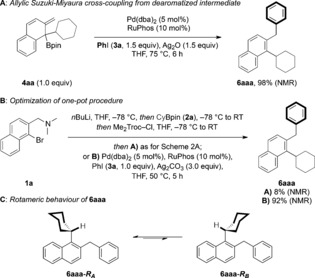
Optimization of γ‐selective allylic Suzuki–Miyaura cross‐coupling. Me_2_Troc‐Cl=2,2,2‐trichloro‐1,1‐dimethylethyl chloroformate.

With the optimized conditions in hand, we went on to investigate the scope of the three‐component coupling reaction (Table 1, part A). Symmetrical cyclic secondary boronic esters gave coupled products **6 aaa**–**6 ada** in excellent isolated yields. While cyclohexyl product **6 aaa** showed rotameric behaviour by ^1^H NMR, cyclopentyl (**6 aba**), cyclobutyl (**6 aca**), and cyclopropyl (**6 ada**) coupled products were observed as single species. An acyclic secondary boronic ester also coupled smoothly, providing **6 aea** in 84 % yield, where broadening of the methylene signal indicated restricted rotation on the ^1^H NMR timescale. For primary alkylboronic esters, the reaction was performed at room temperature for 18 h with improved yields, providing coupled product **6 afa** in 66 % yield. Interestingly, the homocoupling of the dearomatized intermediate could also be isolated in 8 % yield. We attribute this product to an alternative mechanism, involving double transmetalation at a palladium(II) center, followed by reductive elimination and re‐oxidation using Ag_2_CO_3_ as a terminal oxidant. No coupling product was observed with sterically demanding tertiary boronic ester **2 g**; use of benzylamine **1 b**, however, led to coupled product **6 bga** in excellent yield (88 %). Phenylboronic ester **2 h** also underwent coupling to provide biaryl **6 aha** in 76 % yield. In line with previous reports, the 1,2‐boron‐to‐carbon migration proceeded with excellent levels of retentive enantiospecificity, providing chiral products in high e.r. (**6 aia**, 95:5 and **6 bja**, 98:2) and d.r. (**6 aka**, >95:5 and **6 ala**, >95:5). These substrates also highlight the functional group tolerance of the process, with *tert*‐butyl carboxyesters, azides, and TBDPS‐protected alcohols tolerated.

**Table 1:** Substrate scope.^[a]^

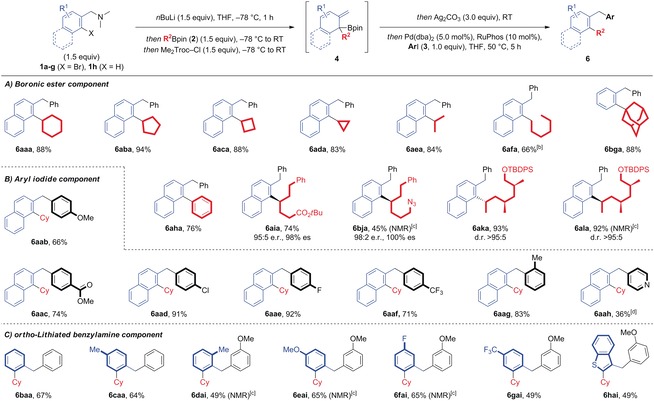

[a] Reactions were performed using 0.3 mmol of **3**, 1.5 equiv of **1**, **2**, *n*BuLi (1.6 m in hexanes) and Me_2_Troc‐Cl, 3 equiv of Ag_2_CO_3_, 5 mol % of Pd(dba)_2_, and 10 mol % of RuPhos. See Supporting Information for exact experimental procedures. Yields refer to isolated products unless otherwise indicated. Diastereomeric ratios were determined by ^1^H NMR analysis of the purified compounds. [b] Final cross‐coupling step at RT for 18 h. [c] Yield determined by ^1^H NMR analysis of the crude reaction mixture using dibromomethane as internal standard. [d] Cross‐coupling step at 75 °C for 5 h.

We then assessed the scope of the aryl iodide and benzylamine coupling partners (Table 1, parts B and C). The electronics of the aryl iodide appeared to have limited effect on reactivity and both electron‐donating (**6 aab**) and electron‐withdrawing substituents (**6 aac**) were well tolerated, as were halides (**6 aad**, **6 aae**, **6 aaf**) and *ortho*‐substitution (**6 aag**). Nitrogen heterocycles could also be incorporated, giving coupled product **6 aah**, albeit in reduced yield. Simple *ortho*‐bromo benzylamine **1 b** underwent smooth coupling to provide **6 baa** in 67 % yield. Electron‐rich and electron‐poor benzylamines were viable substrates, providing **6 caa** and products **6 dai**–**6 gai**. *Ortho*‐substitution was tolerated, as illustrated by bis‐*ortho*‐substituted product **6 dai**, and heteroaryl benzylamines could also be used, as highlighted by benzothiophenyl amine **1 h**, which provided product **6 hai** in moderate yield.^15^

We then turned our attention towards the synthesis of enantioenriched 1,1‐diarylethane derivatives. α‐Methyl benzylamine **(*R*)‐1 i** (99:1 e.r.) was subjected to the standard reaction conditions using cyclohexylboronic ester **2 a** (Scheme 3 A). Coupled product **6 iaa** was formed in 50 % ^1^H NMR yield and 91:9 e.r., corresponding to an enantiospecificity of 84 % from **(*R*)‐1 i**. Since **4 ia** is formed in 96:4 e.r.,^9^ this result indicates that the γ‐selective allylic Suzuki–Miyaura cross‐coupling (**4 ia** to **6 iaa**) proceeds in 87 % es.^16^ For comparison, we prepared and tested α‐methyl benzylic boronic ester **5 ia** (94:6 e.r.) under Crudden's cross‐coupling conditions, which provided 24 % of **6 iaa** in 88:12 e.r. (86 % es), along with 30 % β‐hydride elimination product **7**, 30 % of returned starting boronic ester **5 ia** and 4 % protodeboronation product **8** (Scheme 3 B). The lower yield and formation of side‐products is a consequence of the considerable steric hindrance of boronic ester substrate **5 ia**, highlighting a positive feature of the new process which does not suffer from the same issues.

**Scheme 3:**
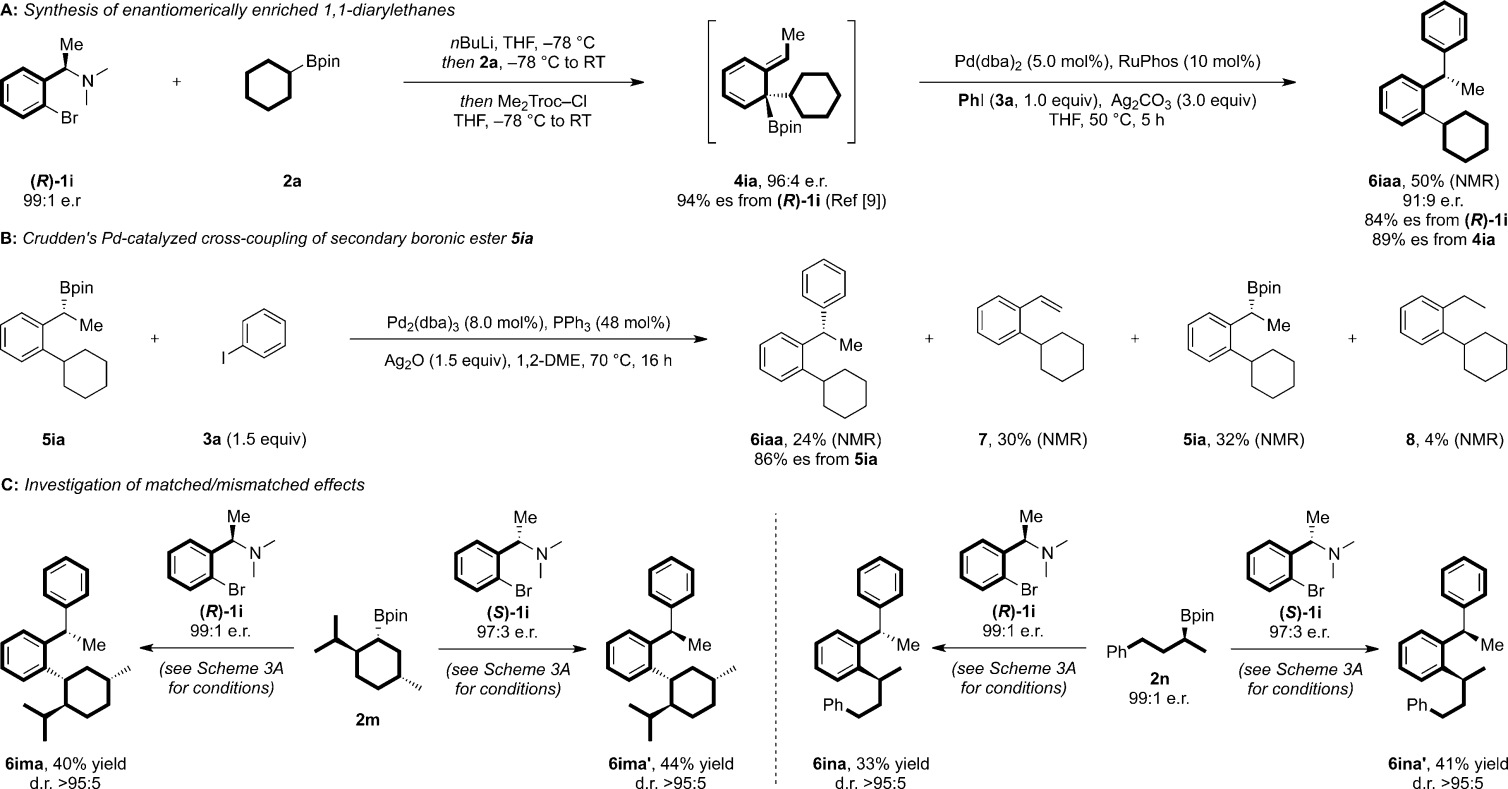
1,1‐Diarylethanes from α‐methyl benzylamines.

To further highlight the utility of this methodology, doubly stereospecific transformations were carried out using both enantiomers of α‐methyl benzylamine **1 i** and (−)‐menthol‐derived boronic ester **2 m** (Scheme 3 C). Coupling with **(*R*)‐1 i** provided product **6 ima** in 40 % isolated yield and >95:5 d.r. and the enantiomeric α‐methyl benzylamine **(*S*)‐1 i** gave the diastereomeric product **6 ima′** in 44 %, again in excellent d.r. (>95:5).^17^ Additionally, reaction of enantioenriched boronic ester **2 n** with **(*R*)‐1 i** and **(*S*)‐1 i** afforded diastereomeric products **6 ina** and **6 ina′**, respectively, both with >95:5 d.r. (Scheme 3 C). These examples indicate that no matched/mismatched effects occur between the benzylamine and boronic ester components.

While secondary boronic ester **5 ia** does undergo direct cross‐coupling to provide cross‐coupled product **6 iaa** when subjected to Crudden's conditions (Scheme 3 B), we believe that a 1,3‐borotropic shift/direct cross‐coupling pathway for α‐methyl benzylamine **(*R*)‐1 i** is unlikely. To rule out such a pathway, we subjected boronic ester **5 ia** to our reaction conditions, and observed no evidence of cross‐coupled product **6 iaa** (Scheme 4 A).^18^ Furthermore, naphthylamine **1 a**, which has been used extensively as a substrate in these studies, is stable with respect to the 1,3‐borotropic shift: heating **4 aa** in the presence of NaBPh_4_, in line with our previously reported conditions,^9^ provided no evidence of the borotropic shift product (Scheme 4 B). Moreover, heating **4 aa** in the presence of Ag_2_CO_3_, in analogy to our optimized allylic cross‐coupling conditions, also showed no reactivity towards 1,3‐borotropic shift. We thus propose that the transformation of α‐methyl benzylamines into 1,1‐diarylethane derivatives proceeds through a series of four highly stereospecific processes: 1) a stereospecific 1,2‐metalate rearrangement that occurs concurrently with 2) a stereospecific *anti*‐S_*N*_2′ elimination of the *N*‐acylated leaving group to give the dearomatized intermediate, **4**, followed by 3) a stereospecific *syn* γ‐selective allylic transmetalation via a six‐membered transition state to give intermediate **5** and 4) a stereospecific retentive reductive elimination (Scheme 4 C). In this way, the chirality in the staring α‐methyl benzylamine is transferred through four sequential processes into the final coupled product with high stereospecificity.

**Scheme 4:**
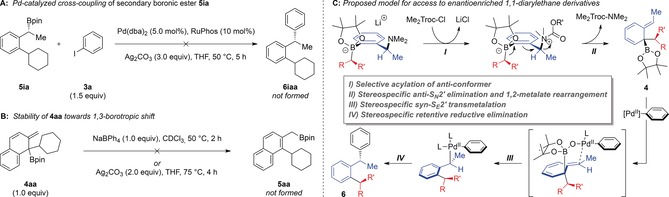
Mechanistic proposal for chirality transfer.

In conclusion, we report a new method for the synthesis of enantioenriched 1,1‐diarylethane derivatives. Through a series of four stereospecific steps, enantioenriched α‐methyl benzylamines are transformed into valuable optically active 1,1‐diarylethanes with good stereospecificity. In terms of reactivity, the key *syn* γ‐selective allylic Suzuki–Miyaura cross‐coupling process appears to overcome structural limitations encountered in the traditional direct cross‐coupling of certain sterically hindered secondary benzylic boronic esters. The highly convergent nature of this coupling process affords sterically encumbered 1,1‐diarylethanes with three readily addressable points of diversification.

## Conflict of interest

The authors declare no conflict of interest.

## Supporting information

As a service to our authors and readers, this journal provides supporting information supplied by the authors. Such materials are peer reviewed and may be re‐organized for online delivery, but are not copy‐edited or typeset. Technical support issues arising from supporting information (other than missing files) should be addressed to the authors.

Supplementary
