# Supplementary material for: Enantiospecific Synthesis of ortho‐Substituted 1,1‐Diarylalkanes by a 1,2‐Metalate Rearrangement/anti‐SN2′ Elimination/Rearomatizing Allylic Suzuki–Miyaura Reaction Sequence
Source: Angew Chem Int Ed Engl. 2018 Dec 21;58(5):1366–70. doi: 10.1002/anie.201811343 (PMC6391954; doi:10.1002/anie.201811343)

Angewandte Chemie
Eine Zeitschrift der Gesellschaft Deutscher Chemiker

Supporting Information

Enantiospecific Synthesis of *ortho*-Substituted 1,1-Diarylalkanes by a 1,2-Metalate Rearrangement/*anti*-S_N2' Elimination/Rearomatizing Allylic Suzuki–Miyaura Reaction Sequence

Belén Rubial[+], *Beatrice S. L. Collins*[+], *Raphael Bigler, Stefan Aichhorn, Adam Noble, and Varinder K. Aggarwal**

anie_201811343_sm_miscellaneous_information.pdf

Table of Contents

1. General Experimental 2

2. Synthesis of Benzylamines **1** 3

3 Synthesis of Boronic Esters **2** 7

4 Synthesis of Aryl Iodides 13

5 Optimization of Reaction Condition for Cross-Coupling Step 14

6 General Procedure 19

7 Product Characterization 20

8 Crystallographic data for product **6ima’** 48

9 Characterization of rotamers **6aaa-*R_A*** and **6aaa-*R_B*** 50

10 Analysis of the enantiospecificity of the process in its application to the synthesis of 1,1-diarylethanes 54

11 Mechanistic investigations on the γ-selective allylic transmetallation vs. 1,3-borotropic shift/direct cross-coupling pathways 59

12 References 61

13 NMR spectra 63

1. General Experimental

Solvents and Reagents. Reactions with air- or moisture-sensitive materials were carried out under a nitrogen atmosphere using Schlenk techniques. Bulk solutions were evaporated under reduced pressure using a Büchi rotary evaporator. All solvents were commercially supplied or provided by the communal stills of the School of Chemistry, University of Bristol. (+)-Sparteine was distilled under reduced pressure and over calcium sulfate prior its use. The sparteine free base readily absorbs atmospheric carbon dioxide (CO_2) and should be stored under nitrogen at –20 °C in a Schlenk tube. All other reagents were purchased from commercial sources and used as sold, unless otherwise indicated.

Chromatography. Flash column chromatography (FCC) was carried out using Sigma-Aldrich silica gel (60 Å, 230-400 mesh, 40-63 μm). All reactions were followed by thin-layer chromatography (TLC) when practical, using Merck Kieselgel 60 F_{254} fluorescent treated silica gel, which was visualised under UV light or by staining with aqueous basic potassium permanganate, acid p-anisaldehyde solution in ethanol or phophomolibdic acid solution in ethanol.

Spectroscopy and characterization techniques. ^{1}H and $^{13}C\{^{1}H\}$ NMR spectra were recorded using Jeol ECP(Eclipse) 300 MHz, Jeol ECS 400 MHz, Varian CNMR 400 MHz, Bruker Avance DPX 400 MHz, and Bruker Avance III HD 500 Cryo 500 Mz spectrometers. Variable temperature ^{1}H NMR and EXSY/NOESY experiments were performed on a Varian VNMR S500 500 MHz spectrometer. ^{1}H and ^{13}C positive chemical shifts (δ) are downfield from tetramethylsilane and are given in parts per million (ppm). Coupling constants (J) are given in Hertz (Hz). The ^{1}H NMR spectra are reported as follows: ppm (multiplicity, coupling constants, assignment). NMR assignments are made according to spin systems, using two dimensional (COSY, HSQC, HMBC) NMR spectroscopy to assist the assignment. High resolution mass spectra (HRMS) were recorded on a Bruker Daltonics UltrafleXtreme Matrix-Assisted Laser Desorption/Ionization (MALDI) or on a Bruker Daltonics micrO TOF II by Electrospray Ionization (ESI). IR spectra were recorded on a Perkin Elmer Spectrum Two FT-IR as a thin film. Only selected absorption maxima (ν_{max}) are reported in wavenumbers (cm^{-1}). Melting points were recorded in degrees Celsius (°C), using a Cole-Parmer Stuart SMP30 melting point apparatus and are reported uncorrected. Optical rotation ($[\boldsymbol{\alpha}]_D^T$) was measured on a Bellingham and Stanley Ltd. ADP220 polarimeter and is quoted in (°α mL)(g dm)$^{-1}$. Chiral HPLC was performed on a HP agilent 1100 with a Chiralpak IA, IB or IC column and monitored by DAD (Diode Array Detector). Preparative HPLC was performed on a Teledyne ISCO ACCQPrep HP125 System using a Kromasyl 60-5SIL column (21.2mm x 250 mm x 5 μm).

Naming of Compounds. Compound names are those generated by ChemBioDraw 16.0 software (PerkinElmer), following IUPAC nomenclature.

2. Synthesis of Benzylamines 1

1-(1-Bromonaphthalen-2-yl)-*N*,*N*-dimethylmethanamine (1a)

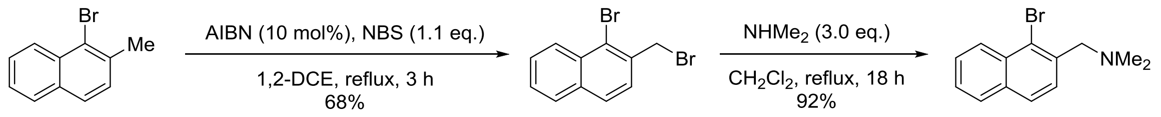

To a solution of 1-bromo-2-methylnaphthalene (3.52 mL, 22.60 mmol) and AIBN (0.37 g, 2.26 mmol) in 1,2-DCE (200 mL) was added NBS (4.47 g, 24.90 mmol) portionwise. The solution was heated to reflux for 2 h. The reaction was then cooled to r.t. and washed successively with H_2O (2 x 20 mL), $Na_2S_2O_3$ (5 wt.% aq., 20 mL) and brine (20 mL), dried over Mg_2SO_4, filtered and concentrated under reduced pressure. Purification by flash column chromatography on silica gel (pentane) afforded the dibromo interemediate, **1-bromo-2-(bromomethyl)naphthalene**, as a white solid. Yield: 4.61 g (68%). ***R*f** (pentane) = 0.33. **^{1}H NMR** (400 MHz, $CDCl_3$): δ (ppm) 8.34 (*d*, $^3J_{H,H'}$ = 8.5 Hz, 1H, Ar–*H*), 7.81 (*d*, $^3J_{H,H'}$ = 7.8 Hz, 1H, Ar–*H*), 7.79 (*d*, $^3J_{H,H'}$ = 8.9 Hz, 1H, Ar–*H*), 7.62 (*ddd*, $^3J_{H,H'}$ = 8.3, 6.7 Hz, $^4J_{H,H'}$ = 1.3 Hz, 1H, Ar–*H*), 7.55 (*d*, $^3J_{H,H'}$ = 7.6 Hz, 1H, Ar–*H*), 7.51 (*d*, $^3J_{H,H'}$ = 8.4 Hz, 1H, Ar–*H*), 4.86 (*s*, 2H, ArC*H*$_2$Br). **^{13}C{^{1}H} NMR** (101 MHz, $CDCl_3$): δ 135.1 (*C*, arom.), 134.3 (*C*, arom.), 132.6 (*C*, arom.), 128.4 (*C*H, arom.), 128.3 (*C*H, arom.), 128.0 (*C*H, arom.), 127.8 (*C*H, arom.), 127.7 (*C*H, arom.), 127.3 (*C*H, arom.), 125.1 (*C*, arom.), 34.9 (Ar*C*H$_2$Br). Analytical data are in agreement with literature.[S1]

A solution of **1-bromo-2-(bromomethyl)naphthalene** (4.53 g, 15.1 mmol) in dichloromethane (40 mL) was treated with a solution of dimethylamine in ethanol (8.0 mL, 5.6 M, 44.8 mmol) and the reaction was heated to reflux and stirred at this temperature for 18 hours. The reaction was then cooled to room temperature and treated sequentially with saturated aqueous $NaHCO_3$ solution (25 mL) and water (25 mL). The phases were separated and the aqueous phase was extracted with ethylacetate (3 x 25 mL). The chlorinated and non-chlorinated organic phases were separately washed with brine (25 mL), combined, dried over $MgSO_4$, filtered and concentrated under reduced pressure. Purification by flash column chromatography on silica gel (gradient, acetone:dichloromethane, 5:95 to 25:75) afforded **1-(1-Bromonaphthalen-2-yl)-*N*,*N*-dimethylmethanamine (1a)** as a pale yellow oil. Yield: 3.66 g (92%). ***R*f** (ethyl acetate:dichloromethane, 50:50) = 0.19. **IR** (liquid film, cm^{-1}): 2940, 2817, 2768, 1500, 1454, 1325, 1255, 1033. **^{1}H NMR** (400 MHz, $CDCl_3$): δ (ppm) 8.36 (*d*, $^3J_{H,H'}$ = 8.3 Hz, 1H, Ar–*H*), 7.82 (*d*, $^3J_{H,H'}$ = 8.1 Hz, 1H, Ar–*H*), 7.79 (*d*, $^3J_{H,H'}$ = 8.4 Hz, 1H, Ar–*H*), 7.61-7.56 (*m*, 2H, Ar–*H*), 7.51 (*ddd*, $^3J_{H,H'}$ = 8.1, 6.8 Hz, $^4J_{H,H'}$ = 1.1 Hz, 1H, Ar–*H*), 3.79 (*s*, 2H, ArC*H*$_2$), 2.35 (*s*, 6H, N(C*H*$_3$)$_2$). **^{13}C{^{1}H} NMR** (101 MHz, $CDCl_3$): δ 136.6 (*C*, arom.), 134.0 (*C*, arom.), 132.6 (*C*, arom.), 128.2 (*C*H, arom.), 128.1 (*C*H, arom.), 127.61 (*C*H, arom.), 127.55 (*C*H, arom.), 127.4 (*C*H, arom.), 126.4 (*C*H, arom.), 124.5 (*C*H, arom.), 64.3

(ArCH_2), 45.80 (N(CH_3)$_2$), 45.77 (N(CH_3)$_2$). **HRMS** (ESI): Calcd. for $C_{13}H_{15}BrN$ *m/z* 264.0382, found *m/z* 264.0387 $[M+H]^+$.

1-(2-Bromophenyl)-*N,N*-dimethylmethanamine (1b)

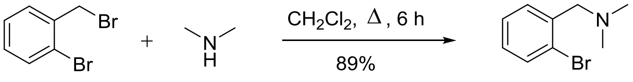

1b

Prepared according to a literature procedure in 89% yield on a 25 mmol scale. [S2]

1-(2-bromo-4-methylphenyl)-*N,N*-dimethylmethanamine (1c)

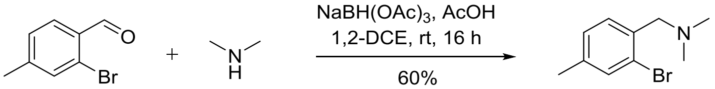

1c

Dimethylamine (7.1 mL, 5.6 M in EtOH, 40.0 mmol, 4.0 equiv) and AcOH (2.29 mL, 40.0 mmol, 4.0 equiv) were added to 2-bromo-4-methylbenzaldehyde (1.99 g, 10.0 mmol) in 1,2-DCE (20 mL) and the solution was stirred for 5 h at RT. NaBH(OAc)$_3$ (8.5 g, 40.0 mmol, 4.0 equiv) was added and the solution was stirred overnight at RT. The solution was diluted with CH_2Cl_2 (50 mL) and H_2O (60 mL), acidified to pH 0-1 by addition of 2 M aqueous HCl solution and the organic phase was separated. The aqueous phase was basified to pH 11-12 by addition of 2 M aqueous NaOH solution and extracted three times with Et_2O (3 × 50 mL). The combined Et_2O phases were washes with saturated aqueous NaCl solution (60 mL), dried over $MgSO_4$, filtered and the solvent was removed under reduced pressure. Flash column chromatography on silica gel (petrol ether:ethyl acetate = 1:1) afforded the product as a colorless oil. Yield: 2.83 g (60%). ***R***$_{\mathbf{f}}$ (petrol ether:ethyl acetate = 1:1): 0.23. **IR** (liquid film, cm^{-1}): 2973, 2941, 2856, 2817, 2769, 1690, 1607, 1562, 1489, 1387, 1360, 1251, 1209, 1173, 1150, 1097, 1043, 1025, 957, 867, 850, 815, 783, 705, 673, 572, 535, 437. **^{1}H NMR** (400 MHz, $CDCl_3$): δ 7.38 (*br dd*, $^4J_{H,H'}$ = 1.8, 0.8 Hz, 1H, Ar–*H*), 7.28 (*d*, $^3J_{H,H'}$ = 7.8 Hz, 1H, Ar–*H*), 7.08 (*br ddd*, $^3J_{H,H'}$ = 7.8 Hz, $^4J_{H,H'}$ = 1.8, 0.8 Hz, 1H, Ar–*H*), 3.48 (*s*, 2H, ArCH_2), 2.31 (*s*, 3H, ArCH_3), 2.29 (*s*, 6H, N(CH_3)$_2$). **^{13}C{^{1}H} NMR** (101 MHz, $CDCl_3$): δ 138.7 (*C*, arom.), 135.1 (*C*, arom.), 133.4 (*C*H, arom.), 131.0 (*C*H, arom.), 128.2 (*C*H, arom.), 124.7 (*C*, arom.), 63.2 (ArCH_2NMe_2), 45.6 (2CH_3, N(CH_3)$_2$), 20.8 (ArCH_3). **HRMS** (ESI): Calcd. for $C_{10}H_{15}BrN$ *m/z* 228.0392, found *m/z* 228.0382 $[M+H]^+$.

1-(2-Bromo-6-methylphenyl)-*N*,*N*-dimethylmethanamine (1d)

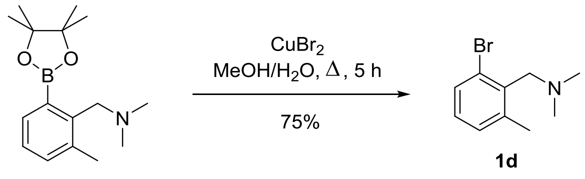

Prepared according to a literature procedure in 75% yield on a 7.5 mmol scale.[S3]

1-(2-Bromo-4-methoxyphenyl)-*N*,*N*-dimethylmethanamine (1e)

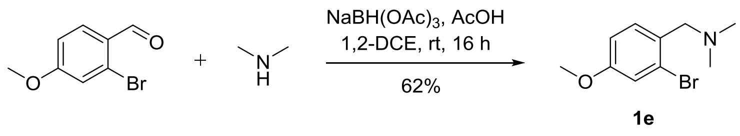

Prepared according to a literature procedure in 61% yield on a 5 mmol scale.[S3]

1-(2-bromo-4-fluorophenyl)-N,N-dimethylmethanamine (1f)

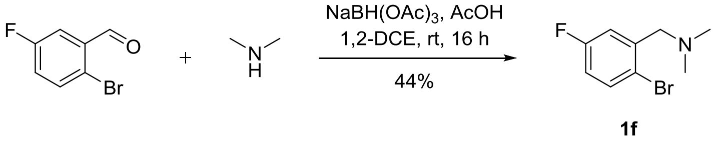

Prepared according to a literature procedure in 44% yield on a 12.3 mmol scale.[S3]

1-(2-Bromo-4-(trifluoromethyl)phenyl)-*N*,*N*-dimethylmethanamine (1g)

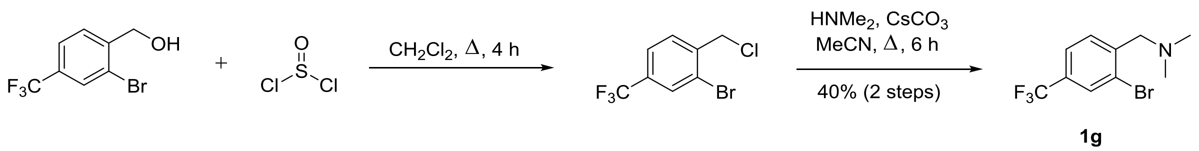

Prepared according to a literature procedure in 40% yield (two steps) on a 7.3 mmol scale.[S3]

1-(Benzo[*b*]thiophen-3-yl)-*N*,*N*-dimethylmethanamine (1h)

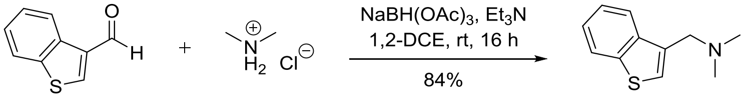

1h

Prepared according to a literature procedure in 84% yield on a 4 mmol scale.[S3]

1-(2-Bromophenyl)-*N*,*N*-dimethylethan-1-amine ((±)-1i), (*R*)-1-(2-Bromophenyl)-*N*,*N*-dimethylethan-1-amine ((*R*)-1i) and (*S*)-1-(2-Bromophenyl)-*N*,*N*-dimethylethan-1-amine ((*S*)-1i)

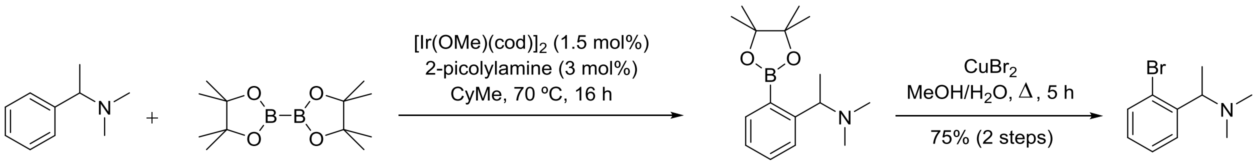

(±)-1i

Prepared according to a literature procedure in 75% yield on a 33.5 mmol scale. As reported, enantiomeric excess was obtained upon debromination. **HPLC**: Chiralpak OD-H (hexane:2-propanol:$HNEt_2$ = 99.9:0.1:0.01, flow rate 0.5 mL/min, λ = 230 nm), retention times t_R(major) = 11.0 min, t_R(minor) = 12.4 min; 98.0%ee for **(*R*)-1i** and 94.4%ee for **(*S*)-1i**.[S3]

3 Synthesis of Boronic Esters 2

Boronic acid pinacol esters **2a**, **2d, 2e, 2f**, and **2h** were obtained from commercial sources and used as received.

2-cyclopentyl-4,4,5,5-tetramethyl-1,3,2-dioxaborolane (2b)

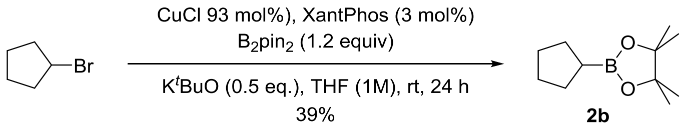

Prepared according to an addapted literature procedure in 39% yield on a 10 mmol scale.[S4] **^{1}H NMR** (400 MHz, $CDCl_3$): δ 1.81-1.68 (*m*, 2H, cyclopentyl C*H$_2$*), 1.65-1.38 (*m*, 6H, cyclopentyl C*H$_2$*), 1.29-1.10 (*m*, 1H, cyclopentyl C*H*), 1.24 (*s*, pinacol C*H$_3$*). **^{13}C{^{1}H} NMR** (101 MHz, $CDCl_3$): δ 83.0 (*C*, pinacol), 28.7 (*C*H$_2$, cyclopentyl), 27.0 (*C*H$_2$, cyclopentyl), 24.9 (*C*H$_3$). Carbon attached to boron not observed due to quadrupolar relaxation. Analytical data are in agreement with the literature.[S5]

2-cyclobutyl-4,4,5,5-tetramethyl-1,3,2-dioxaborolane (2c)

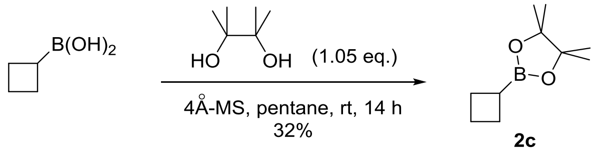

Cyclobutylboronic acid (1.0 g, 10 mmol, 1 equiv) was suspended on 30 mL of pentane and 4Å-MS were added. The mixture was cooled to 0 °C and pinacol was added slowly. The mixture was allowed to warm up to room temperature and stirred overnight . Sodium sulfate was added to eliminate any remaining moisture and the suspension was filtered through a Celite® path, washing twice with 15 mL of diethyl ether. Solvents were eliminated under reduced pressure. The product was purified by flash column chromatography on silica gel (petrol ether ; ethyl acetate = 97.5:2.5, R_f = 0.59 (petrol ether:ethyl acetate = 91:9)) to obtain 0.58 g of a colourless liquid (3.18 mmol, 32% yield). **^{1}H NMR** (400 MHz, $CDCl_3$): δ 2.18-1.83 (*m*, 7H, cyclobutyl C*H* and C*H$_2$*), 1.25 (*s*, pinacol C*H$_3$*). **^{13}C{^{1}H} NMR** (101 MHz, $CDCl_3$): δ 83.1 (*C*, pinacol), 24.9 (*C*H$_3$, pinacol), 24.1 (*C*H$_2$, cyclobutyl), 22.9 (*C*H$_2$, cyclobutyl). Carbon attached to boron not observed due to quadrupolar relaxation. Analytical data are in agreement with the literature.[S5]

2-((3r,5r,7r)-adamantan-1-yl)-4,4,5,5-tetramethyl-1,3,2-dioxaborolane (2g)

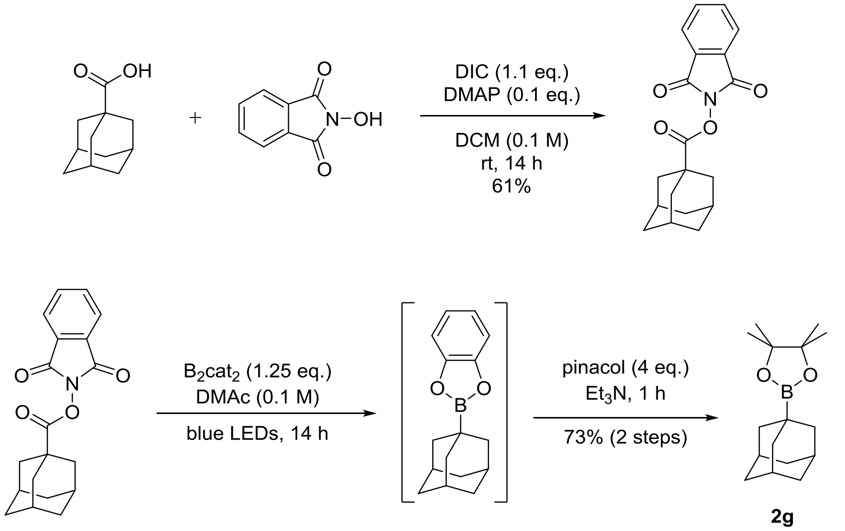

2g

Prepared according to a literature procedure in 73% yield on a 6.0 mmol scale.[S6]

***tert*-Butyl (*S*)-6-phenyl-4-(4,4,5,5-tetramethyl-1,3,2-dioxaborolan-2-yl)hexanoate ((S)-2i)**

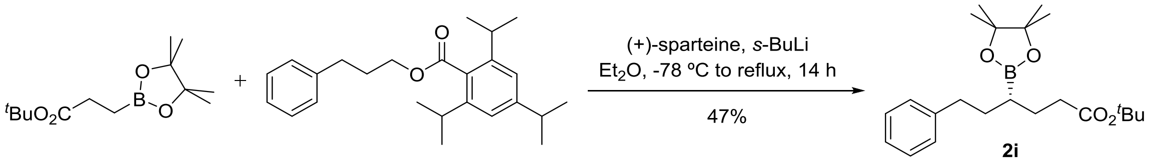

2i

Prepared according to a literature procedure in 47% yield on a 16.5 mmol scale.[S3] Enantiomeric excess was determined after oxidation to the corresponding alcohol. **HPLC**: Chiralpak IB (hexane: 2-propanol = 97:3, flow rate 0.7 mL/min, λ = 210 nm), retention times t_R(minor) = 14.4 min, t_R(major) = 20.2 min; 4:96 e.r.; 91.6%ee.

Figure S1: HPLC chromatograms and data tables for compounds **(±)-2i** and **(*S*)-2i**, respectively.

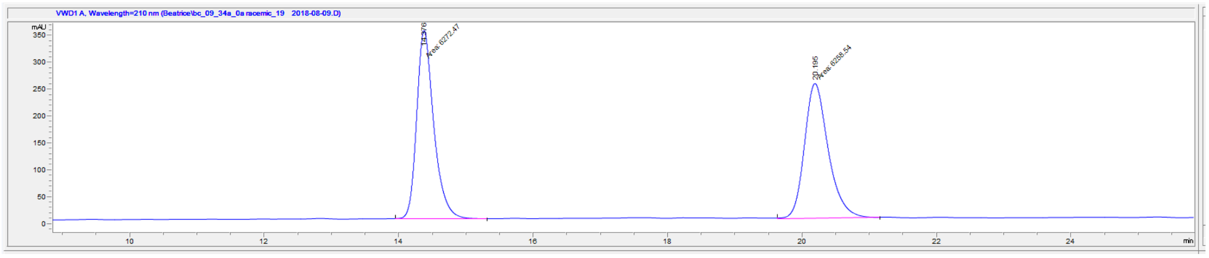

#	Time	Type	Area	Height	Width	Area%	Symmetry
1	14.376	MM	6272.5	351.8	0.2972	50.056	0.725
2	20.195	MM	6258.5	251.9	0.4141	49.944	0.769

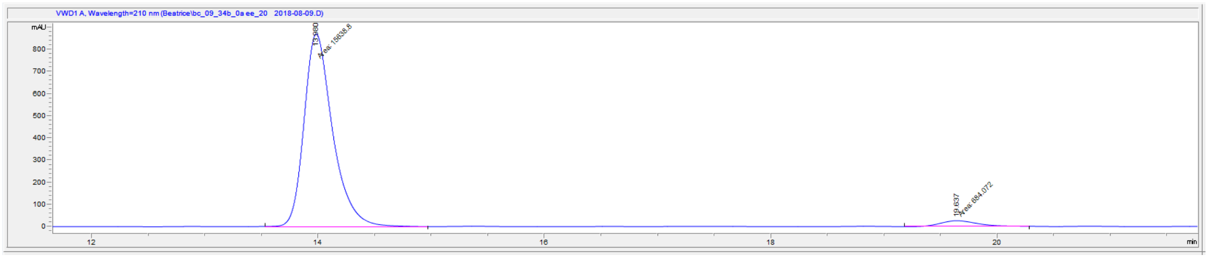

#	Time	Type	Area	Height	Width	Area%	Symmetry
1	13.98	MM	15638.8	876.2	0.2975	95.809	0.707
2	19.637	MM	684.1	27.4	0.4155	4.191	0.729

(*S*)-2-(6-Azido-1-phenylhexan-3-yl)-4,4,5,5-tetramethyl-1,3,2-dioxaborolane (2j)

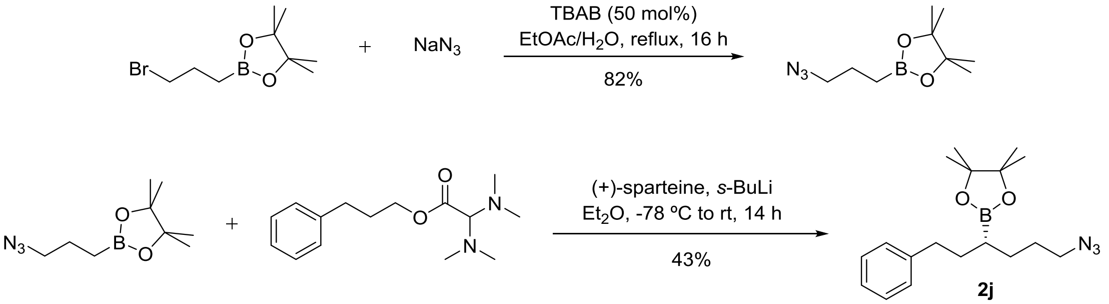

Prepared according to a literature procedure in 82% yield on a 20 mmol scale for the first step and in 43% yield on a 7.5 mmol scale for the second step.[S3] The enantiomeric excess was determined after oxidation to the corresponding alcohol. **HPLC**: Chiralpak IB (hexane:2-propanol = 97:3, flow rate 0.7 mL/min, λ = 210 nm), retention times t_R(major) = 27.8 min, t_R(minor) = 37.8 min; 98:2 e.r., 95.6%ee.

Figure S2: HPLC chromatograms and data tables for compounds **(±)-2j** and **(*S*)-2j**, respectively.

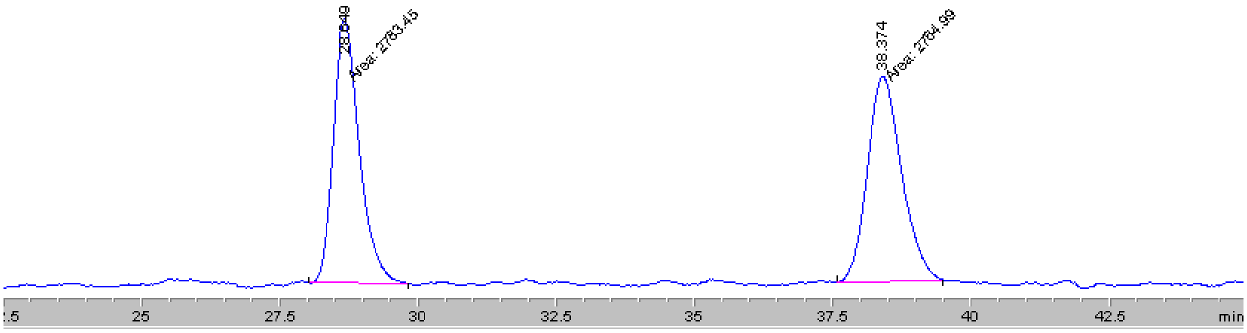

Peak #	RetTime [min]	Type	Width [min]	Area [mAU*s]	Height [mAU]	Area %
1	28.649	MM	0.5615	2783.44946	82.62399	50.1664
2	38.374	MM	0.7162	2764.98853	64.34251	49.8336

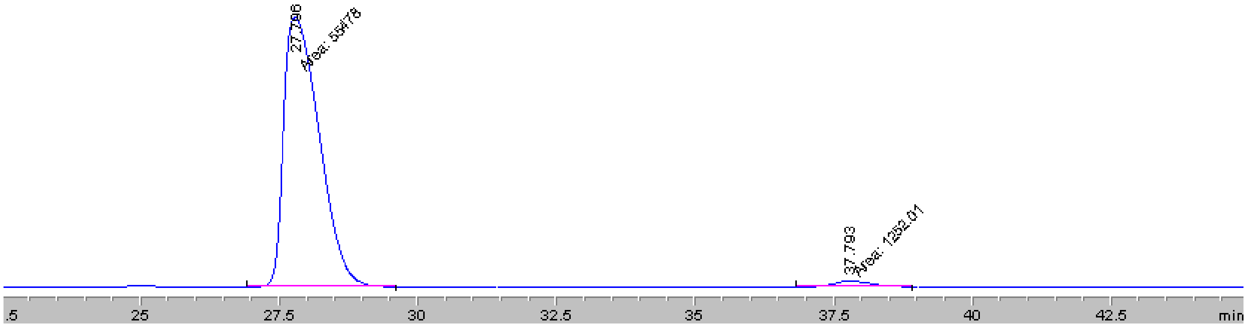

Peak #	RetTime [min]	Type	Width [min]	Area [mAU*s]	Height [mAU]	Area %
1	27.796	MM	0.7373	5.54780e4	1254.08167	97.7930
2	37.793	MM	0.7909	1252.00745	26.38247	2.2070

***tert*-Butyl(((2*R*,4*R*,6*R*)-2,4-dimethyl-6-(4,4,5,5-tetramethyl-1,3,2-dioxaborolan-2-yl)heptyl)-oxy)diphenylsilane (2k)**

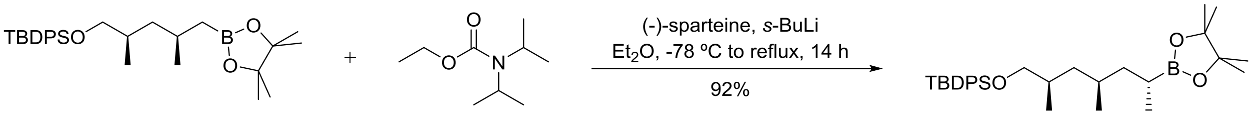

Prepared according to a literature procedure in 92% and with dr >95:5 on a 2.0 mmol scale.[S3]

***tert*-Butyl(((2*R*,4*R*,6*S*)-2,4-dimethyl-6-(4,4,5,5-tetramethyl-1,3,2-dioxaborolan-2-yl)heptyl)-oxy)diphenylsilane (2l)**

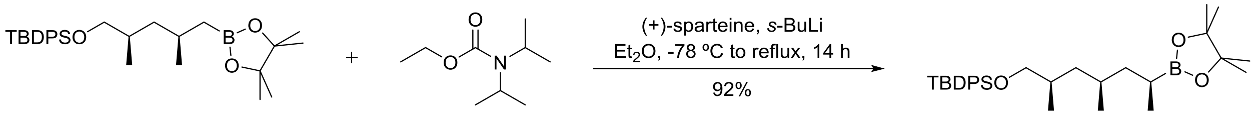

Prepared according to a literature procedure in 92% and with dr >95:5 on a 2.0 mmol scale.[S3]

2-((1*R*,2*R*,5*R*)-2-isopropyl-5-methylcyclohexyl)-4,4,5,5-tetramethyl-1,3,2-dioxaborolane (2m)

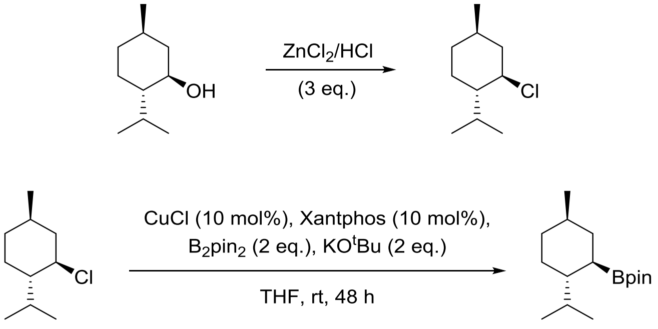

2m

Concentrated HCl (aq.) (3.4 equiv, 340 mmol, 28 mL) was poured into a 250 mL round bottom flask. After placing it into an ice bath, zinc chloride (3 equiv, 300 mmol, 41 g) was added in portions. When it was completely dissolved, the mixture was allowed to reach room temperature. **(*L*)-Menthol** (1 equiv, 100 mmol, 15.6 g) was added in one portion. The suspension was stirred over 2 days to give a colourless biphasic mixture. The reaction completion was checked by TLC using pentane as the eluent and p-anilsadehyde stain (R_f = 0.65). The aqueous layer was extracted with pentane and dried over magnesium sulfate. The solvent was evaporated to obtain a colourless liquid crude mixture. The compound was purified by flash column chromatography on silica gel pentane as eluent. 14 g of pure **(1*S*,2*R*,4*R*)-2-chloro-1-isopropyl-4-methylcyclohexane** were obtained (80 mmol, 80% yield). **^{1}H-NMR** (400 MHz, $CDCl_3$, 25 °C): δ (ppm) 3.77 (*td*, $^3J_{H,H'}$ = 11.0, 4.2 Hz, 1H, –C*H*Cl–), 2.33 (*septd*, $^3J_{H,H'}$ = 7.0, 2.9 Hz, 1H), 2.25-2.17 (*m*, 1H), 1.77-1.64 (*m*, 2H), 1.48-1.31 (*m*, 3H), 1.10-0.82 (*m*, 3H), 0.92 (*d*, $^3J_{H,H'}$ = 7.3 Hz, 3H, –CH*Me₂*), 0.91 (*d*, $^3J_{H,H'}$ = 5.8 Hz, 3H, –CH*Me*), 0.76 (*d*, $^3J_{H,H'}$ = 7.3 Hz, 3H, –CH*Me₂*). **^{13}C{^{1}H}-NMR** (101 MHz, $CDCl_3$, 25 °C): δ (ppm) 64.1 (CH), 50.6 (CH), 46.9 (CH_2), 34.4 (CH_2), 33.6 (CH), 27.3 (CH), 24.5 (CH_2), 22.1 (CH_3), 21.2 (CH_3), 15.3 (CH_3). Analytical data are in agreement with the literature.[S7]

A flame-dried 250 mL two-necked flask, under a nitrogen atmosphere, was filled with copper(I) chloride (0.35 g, 3.5 mmol, 0.1 equiv) and XantPhos (2.03 g, 3.5 mmol, 0.1 equiv). Subsequently, 2.8 mL of dry THF (35 mmol, 1 equiv) were added, followed by 17.78 g (70 mmol, 2 equiv) of B_2pin_2. The flask was put into an ice bath and 70 mL (70 mmol, 2 equiv) of a 1M potassium *tert*-butoxide solution in THF was added slowly via syringe. All the reagents were allowed to mix up at room temperature for 5 min, then the flask was put back into the ice bath and **(1*S*,2*R*,4*R*)-2-chloro-1-isopropyl-4-methylcyclohexane** was added dropwise, via syringe, over 10 minutes. The brown suspension was stirred at room temperature for 48 hours. Completion of the reaction was checked by tacking a small aliquot, filtering through a small path of silica gel, eluting with diethyl ether, and performing a TLC on the solution (100% petrol ether, R_f = 0.51, p-anisaldehyde stain). The reaction mixture was diluted with petrol ether and filtrated through a path of silica gel to eliminate insoluble and metal derivative components in the mixture, eluting with 9% ethyl acetate in petrol ether. A TLC on the filtrate (eluent: petrol ether) showed 2 spots, the product (R_f = 0.51) and another spot at the baseline. Evaporation of solvents afforded a colourless liquid crude mixture. The compound was purified by flash column chromatography on silica gel, eluting with 4% diethyl ether in petrol ether to obtain 4.4 g (32 mmol, 91% yield) of **2m** as a colourless liquid. **^{1}H-NMR** (400 MHz, $CDCl_3$, 25 ºC): δ (ppm) 1.75-1.67 (*m*, 1H), 1.66-1.57 (*m*, 3H), 1.37-1.16 (*m*, 14H), 1.01-0.86 (*m*, 4H), 0.90 (*d*, $^3J_{H,H'}$ = 6.9 Hz, 3H, –CH*Me₂*), 0.84 (*d*, $^3J_{H,H'}$ = 6.5 Hz, 3H, –CH*Me*), 0.77 (*d*, $^3J_{H,H'}$ = 6.9 Hz, 3H, –CH*Me₂*). **^{13}C{^{1}H}-NMR** (101 MHz, $CDCl_3$, 25 ºC): δ (ppm) 82.9 (2 C), 44.0 (CH), 37.4 (CH_2), 35.5 (CH_2), 33.7 (CH), 32.2 (CH), 26.1 (CH_2), 25.0 (CH_3), 24.9 (CH_3), 22.9 (CH_3), 21.9 (CH_3), 16.7 (CH_3). Analytical data are in agreement with the literature.[S4]

(*S*)-4,4,5,5-tetramethyl-2-(4-phenylbutan-2-yl)-1,3,2-dioxaborolane (2n)

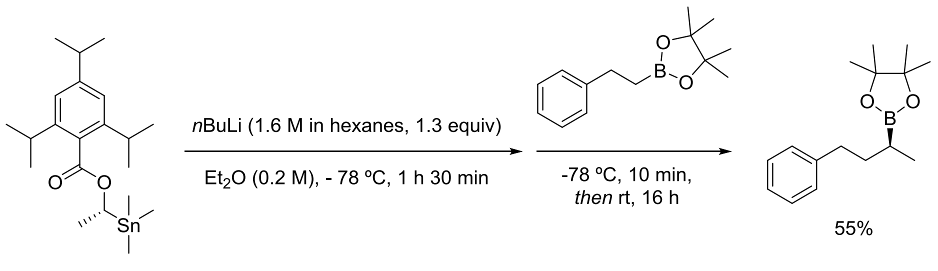

Prepared according to a literature procedure in 55% on a 1.2 mmol scale.[S8a-c] Purification was achieved by flash column chromatography (2% ethyl acetate and 9% toluene in petrol ether, R_f = 0.41), followed by preparative TLC on 1500 micron silica plates, and eluting twice with the 2% ethyl acetate and 9% toluene in petrol ether. The impurity was identified by GCMS as ethyl 2,4,6-triisopropylbenzoate. Analytical data are in agreement with the literature.[S8d] The enantiomeric excess was determined after oxidation to the

corresponding alcohol.[S8d] **HPLC**: Chiralpak IB (hexane:2-propanol = 97:3, flow rate 0.7 mL/min, λ = 210 nm), retention times t_R(minor) = 13.7 min, t_R(major) = 18.5 min; 1:99 e.r., 98.8%ee.

Figure S3: HPLC chromatograms and data tables for compounds **(±)-2n** and **(*S*)-2n**, respectively.

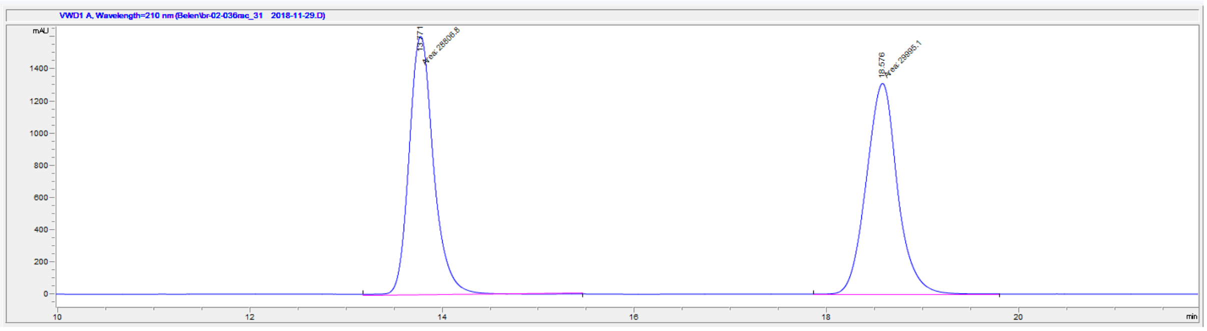

#	Time	Type	Area	Height	Width	Area%	Symmetry
1	13.771	MM	28806.8	1611.6	0.2979	48.990	0.846
2	18.576	MM	29995.1	1316.3	0.3798	51.010	1.022

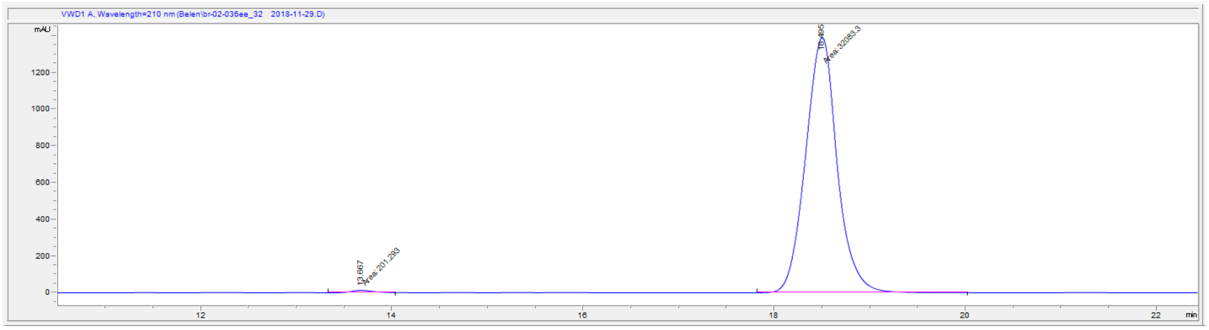

#	Time	Type	Area	Height	Width	Area%	Symmetry
1	13.667	MM	201.3	12.7	0.2651	0.623	0.719
2	18.495	MM	32083.3	1397.6	0.3826	99.377	1.056

4 Synthesis of Aryl Iodides

Aryl iodides **3a**, **3b**, **3e**, **3f**, **3g** and **3i** were obtained from commercial sources and distilled prior their use. 4-iodopyridine **3c**, **3d** and **3h** were obtained from commercial sources and used as received.

5 Optimization of Reaction Condition for Cross-Coupling Step

2-(1-Cyclohexyl-2-methylene-1,2-dihydronaphthalen-1-yl)-4,4,5,5-tetramethyl-1,3,2-dioxaborolane (4aa)

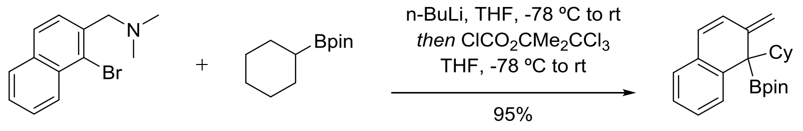

1a **2a** **4aa**

n-BuLi (984 µL, 1.57 mmol, 1.05 equiv) was added to **1a** (416 mg, 1.57 mmol, 1.05 equiv) in THF (6 mL) at –78 °C and the solution was stirred at –78 °C for 60 min. **2a** (315.2 mg, 1.50 mmol) was added and the solution was stirred at –78 °C for 15 min and at RT for 15 min. 2,2,2-Trichloro-1,1-dimethylethyl chloroformate (395.8 mg, 1.65 mmol, 1.10 equiv) was added at –78 °C and the solution was stirred at –78 °C for 15 min and at RT for 15 min. Et_2O (50 mL) was added and the solution was washed with water (50 mL) and saturated aqueous NaCl solution (50 mL), dried over $MgSO_4$ and filtered. Removal of the solvent under reduced pressure afforded the crude product as an oil, which was used without further purification. The crude product contains 1.04 equiv of 1,1,1-trichloro-2-methylpropan-2-yl dimethylcarbamate as determined by ^{1}H NMR spectroscopy, indicating a purity of 57%. Yield: 878.0 mg (95%). $\boldsymbol{R_f}$ (Et_2O:pentane = 4:96): 0.43. **IR** (liquid film, cm^{-1}): 2977, 2927, 2851, 1511, 1479, 1449, 1371, 1328, 1270, 1215, 1143, 1008. **^{1}H NMR** (400 MHz, $CDCl_3$): δ 7.20-7.15 (*m*, 1H, Ar–*H*), 7.12-7.06 (*m*, 2H, Ar–*H*), 7.02-6.96 (*m*, 1H, Ar–*H*), 6.27 (*d*, $^3J_{H,H'}$ = 9.8 Hz, 1H, =C*H*), 6.24 (*d*, $^3J_{H,H'}$ = 9.8 Hz, 1H, =C*H*), 5.19 (*s*, 1H, =C*H*H), 4.90 (*s*, 1H, =C*H*H), 1.84-1.73 (*m*, 1H, C*H*), 1.70-1.63 (*m*, 1H, C*H*H), 1.63-1.49 (*m*, 3H, C*H*H), 1.34-1.18 (*m*, 2H, C*H*H), 1.26 (*s*, 6H, CH_3 (pin)), 1.23 (*s*, 6H, CH_3 (pin)), 1.16-0.91 (*m*, 4H, C*H*H). **^{11}B{^{1}H} NMR** (128 MHz, $CDCl_3$): δ 32.4 (*s*). **^{13}C{^{1}H} NMR** (101 MHz, $CDCl_3$): δ 146.0 (=*C*), 139.4 (arom.), 133.7 (arom.), 131.2 (=*C*H), 129.0 (arom.), 127.1 (arom.), 127.0 (=*C*H), 126.4 (arom.), 125.9 (arom.), 116.0 (=CH_2), 83.7 (2C, O*C*(CH_3)$_2$), 52.1 (*C*H), 28.6 (*C*H_2), 27.7 (*C*H_2), 27.4 (*C*H_2), 27.2 (*C*H_2), 26.8 (*C*H_2), 24.7 (2C, *C*H_3 (pin)), 24.6 (2C, *C*H_3 (pin)). Carbon attached to boron not observed due to quadrupolar relaxation.

Initial optimization of reaction conditions was performed with isolated intermediate **4aa**. To this end, Ag_2O (52.1 mg, 225 µmol, 1.50 equiv), PhI (25 µL, 225 µmol, 1.50 equiv), ligand, precatalyst and solvent (1.5 mL) were added to **4aa** (92.2 mg, 57%, 150 µmol) and the solution was stirred at the indicated temperature for 6 h. 1,3,5-Trimethoxybenzene (25.2 mg, 150 µmol, 1.00 equiv) and Et_2O (5 mL) were added at room temperature and the solution was stirred for 5 min at room temperature before the solids were left to settle. The supernatant was filtered through a pad of silica gel in a pipette and the solvent was removed under reduced pressure. Conversion and yield were determined by ^{1}H NMR spectroscopy.

Note: ^{1}H NMR yields were obtained in $CDCl_3$, using 1,3,5-trimethoxybenzene as internal standard, and based on the integration of the bisbenzylic protons (Nf–CH_2–Ph) in the major rotamer (see Product Characterization, page S20). The minor rotamer equivalent signal was not visible or overlapped with other impurities. Regarding the ratio of major rotamer:minor rotamer = 87:13 obtained for pure compound **6aaa**, yields in the crude mixtures were extrapolated and the results are reported in Tables S1-S6.

Table S1: Ligand Screening for Cross-Coupling Step

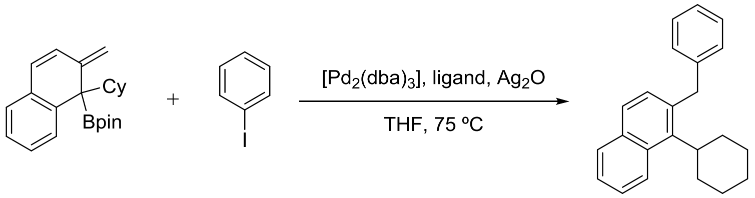

4aa **3a** **6aaa**

entry	ligand	precatalyst	solvent	T	conversion	yield
1	PPh_3 (15 mol%)	$[Pd_2(dba)_3]$ (5 mol%)	THF	75 °C	100%	62%
2	RuPhos (15 mol%)	$[Pd_2(dba)_3]$ (5 mol%)	THF	75 °C	100%	91%
3	SPhos (15 mol%)	$[Pd_2(dba)_3]$ (5 mol%)	THF	75 °C	100%	74%
4	XPhos (15 mol%)	$[Pd_2(dba)_3]$ (5 mol%)	THF	75 °C	100%	13%
5	cataCXium (15 mol%)	$[Pd_2(dba)_3]$ (5 mol%)	THF	75 °C	100%	59%
6	DavePhos (15 mol%)	$[Pd_2(dba)_3]$ (5 mol%)	THF	75 °C	100%	26%
7	dppf (15 mol%)	$[Pd_2(dba)_3]$ (5 mol%)	THF	75 °C	77%	5%

Table S2: Precatalyst Screening for Cross-Coupling Step

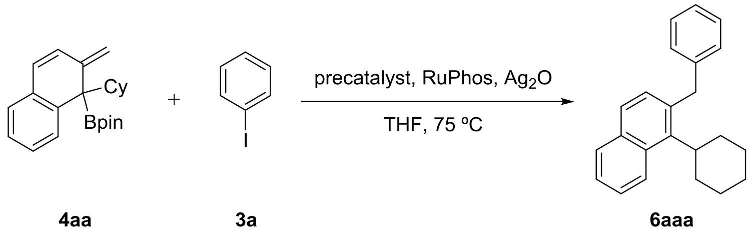

entry	ligand	precatalyst	solvent	T	conversion	yield
1	RuPhos (15 mol%)	$[Pd_2(dba)_3]$ (5 mol%)	THF	75 °C	100%	91%
2	RuPhos (15 mol%)	$[Pd(dba)_2]$ (10 mol%)	THF	75 °C	100%	97%
3	RuPhos (15 mol%)	$[PdCl(allyl)]_2$ (5 mol%)	THF	75 °C	100%	61%
4	RuPhos (15 mol%)	$[PdCl(cinnamyl)]_2$ (5 mol%)	THF	75 °C	100%	70%
5	RuPhos (15 mol%)	$Pd(OAc)_2$ (10 mol%)	THF	75 °C	100%	76%
6	RuPhos (15 mol%)	$[Pd(OMs)(C\text{-}NH_2)]_2$ (5 mol%)	THF	75 °C	100%	85%
7	RuPhos (15 mol%)	$[PdCl(C\text{-}NMe_2)]_2$ (5 mol%)	THF	75 °C	100%	91%

Table S3: Catalyst Loading Screening for Cross-Coupling Step

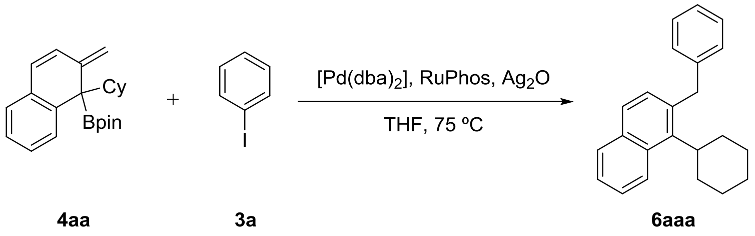

entry	ligand	precatalyst	solvent	T	conversion	yield
1	RuPhos (15 mol%)	$[Pd(dba)_2]$ (10 mol%)	THF	75 °C	100%	97%
2	RuPhos (5 mol%)	$[Pd(dba)_2]$ (5 mol%)	THF	75 °C	100%	70%
3	RuPhos (7.5 mol%)	$[Pd(dba)_2]$ (5 mol%)	THF	75 °C	100%	78%
4	RuPhos (10 mol%)	$[Pd(dba)_2]$ (5 mol%)	THF	75 °C	100%	98%
5	RuPhos (15 mol%)	$[Pd(dba)_2]$ (5 mol%)	THF	75 °C	100%	94%

Table S4: Solvent Screening for Cross-Coupling Step

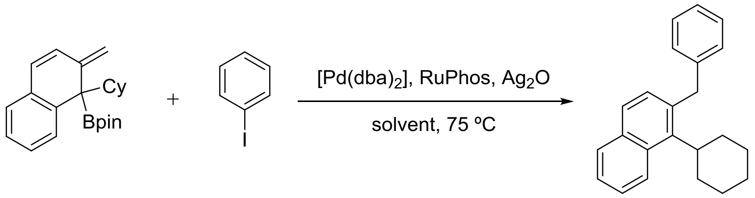

entry	ligand	precatalyst	solvent	T	conversion	yield
1	RuPhos (10 mol%)	$[Pd(dba)_2]$ (5 mol%)	THF	75 °C	100%	98%
2	RuPhos (10 mol%)	$[Pd(dba)_2]$ (5 mol%)	DME	75 °C	100%	84%
3	RuPhos (10 mol%)	$[Pd(dba)_2]$ (5 mol%)	dioxane	75 °C	22%	0%
4	RuPhos (10 mol%)	$[Pd(dba)_2]$ (5 mol%)	MeCN	75 °C	100%	91%
5	RuPhos (10 mol%)	$[Pd(dba)_2]$ (5 mol%)	PhMe	75 °C	100%	95%

Table S5: Temperature Screening for Cross-Coupling Step

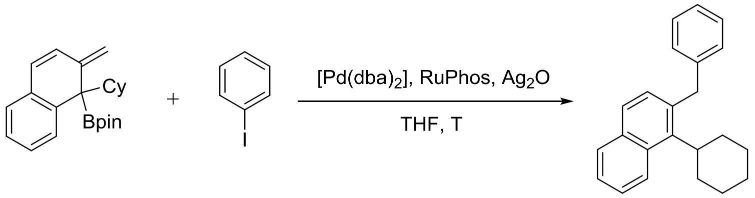

entry	ligand	precatalyst	solvent	T	conversion	yield
1	RuPhos (10 mol%)	$[Pd(dba)_2]$ (5 mol%)	THF	75 °C	100%	98%
2	RuPhos (10 mol%)	$[Pd(dba)_2]$ (5 mol%)	THF	60 °C	100%	85%
3	RuPhos (10 mol%)	$[Pd(dba)_2]$ (5 mol%)	THF	25 °C	92%	59%

After these screening studies we determined that the best ligand for the cross-coupling step was RuPhos (10 mol%) and the best palladium species was $[Pd(dba)_2]$ (5 mol%). The best solvent and temperature conditions were THF at 75 ºC.

In a second step, optimization of reaction conditions focused on the one-pot procedure, which was carried out on a 300 µmol scale for the corresponding limiting reagent indicated in Table S6. To this end, n-BuLi was added to **1a** in THF (2 mL) at -78 °C and the solution was stirred at -78 °C for 60 min. **2a** was added and the solution was stirred at -78 °C for 15 min and at room temperature for 15 min. 2,2,2-Trichloro-1,1-dimethylethyl chloroformate was added at -78 °C and the solution was stirred at -78 °C for 15 min and at room temperature for 10 min. The corresponding silver salt was added and the solution was stirred at room temperature for 10 min. A solution of RuPhos (14.0 mg, 30.0 µmol, 10 mol%), [Pd(dba)$_2$] (8.6 mg, 15.0 µmol, 5 mol%), and **3a** in THF (1 mL) was added and the reaction mixture was stirred at the temperature indicated in Table S6 and during 5 h. The reaction mixture was diluted with 5 mL of Et_2O, filtered through a pad of silica gel with Et_2O (50 mL) and the solvent was removed under reduced pressure. 1,3,5-Trimethoxybenzene (50.5 mg, 300 µmol, 1.00 equiv) was added at room temperature and the solvents were removed under reduced pressure. Conversion and yield were determined by ^{1}H NMR spectroscopy.

Table S6: Condition screening for one-pot procedure

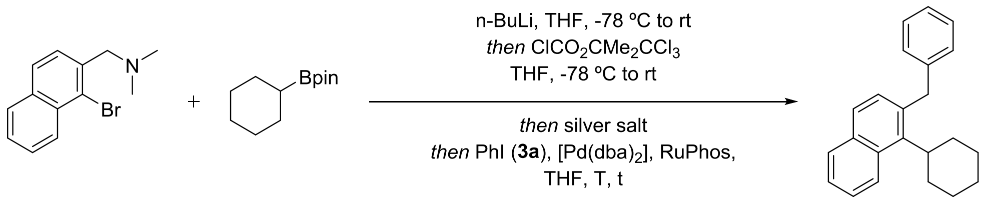

1a **2a** **6aaa**

entry	1a, 2a and $ClCO_2CMe_2CCl_3$	3a	Silver salt	T	conversion	yield
1	1 equiv	1.5 equiv	Ag_2O (1.5 equiv)	75 °C	100%	8%
2	1 equiv	1.5 equiv	Ag_2CO_3 (1.5 equiv)	75 °C	100%	67%
3	1 equiv	1.5 equiv	Ag_2CO_3 (3 equiv)	75 °C	100%	62%
4	1.5 equiv	1 equiv	Ag_2CO_3 (1.5 equiv)	75 °C	100%	84%
5	1.5 equiv	1 equiv	Ag_2CO_3 (3 equiv)	75 °C	100%	90%
6	1.5 equiv	1 equiv	Ag_2CO_3 (3 equiv)	50 °C	100%	92%

6 General Procedure

General Procedure A

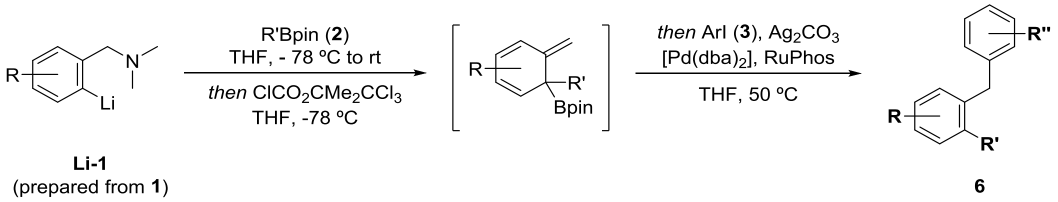

Note: Lithiated substrates **Li-1** were prepared by halogen exchange with *n*-BuLi on the corresponding aryl bromide **1**, with the exception of 1-(benzo[*b*]thiophen-3-yl)-*N*,*N*-dimethylmethanamine (**1h**), for which the direct lithiation on the benzylic ring was achieved under the same conditions.

n-BuLi (281 µL, 1.60 M in hexanes, 450 µmol, 1.50 equiv) was added to substrate **1** (450 µmol, 1.50 equiv) in THF (2 mL) at –78 °C and the solution was stirred at –78 °C for 1 h. Boronic ester **2** (450 µmol, 1.50 equiv) was added and the solution was stirred for 15 min at –78 °C and for 15 min at RT. 2,2,2-Trichloro-1,1-dimethylethyl chloroformate (108.0 mg, 450 µmol, 1.50 equiv) was added at -78 °C and the solution was stirred at –78 °C for 15 min and at RT for 10 min. Ag_2CO_3 (248.2 mg, 900 µmol, 3.00 equiv) was added and the solution was stirred at RT for 10 min. A solution of RuPhos (14.0 mg, 30.0 µmol, 10 mol%), [Pd(dba)$_2$] (8.6 mg, 15.0 µmol, 5 mol%), and the aryl iodide **3** (300 µmol, 1 equiv) in THF (1 mL) was added and the mixture was stirred at 50 °C for 5 h. The reaction mixture was diluted with 5 mL of Et_2O, filtered through a pad of silica gel with Et_2O (50 mL) and the solvent was removed under reduced pressure. Purification by flash column chromatography on silica gel afforded the pure product **6**.

7 Product Characterization

Note: For products **6** existing as a mixture of rotamers, the ratio was obtained by integration of the signals corresponding to the bisbenzylic protons (ArCH_2Ar) in each rotamer.

2-Benzyl-1-cyclohexylnaphthalene (6aaa)

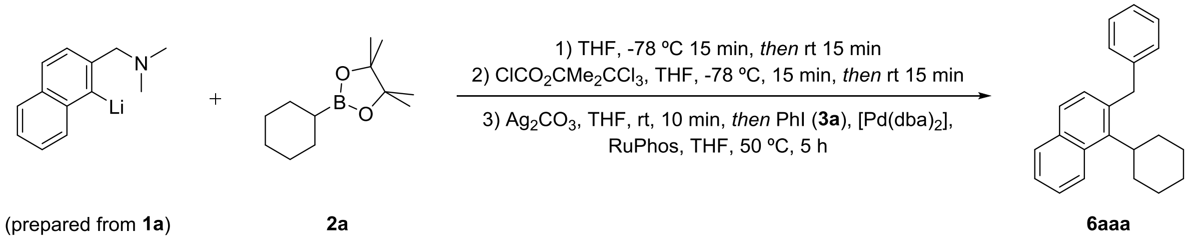

Prepared following general procedure A on a 300 µmol (34 µL) scale. Flash column chromatography on silica gel (hexane) afforded the product as a colorless oil. Yield: 79 mg (88%). ***R*f** (petrol ether:ethyl acetate = 91:9) = 0.69. **IR** (liquid film, cm^{-1}): 3051, 3025, 2924, 2850, 1598, 1494, 1448, 1029, 829, 800, 739, 720, 695. **^{1}H NMR** (400 MHz, $CDCl_3$) [*Note:* **6aaa** exists as a mixture of two rotamers observable by ^{1}H NMR, 87:13] *δ* **[Major rotamer]** 8.47 (*d*, $^3J_{H,H'}$ = 8.4 Hz, 1H, Ar–*H*), 7.89-7.79 (*m*, 1H, Ar–*H*), 7.70 (*d*, $^3J_{H,H'}$ = 8.3 Hz, 1H, Ar–*H*), 7.50-7.36 (*m*, 3H, Ar–*H*), 7.34-7.23 (*m*, 2H, Ar–*H*), 7.23-7.09 (*m*, 3H, Ar–*H*), 4.26 (*s*, 2H, ArCH_2Ar), 3.36 (*tt*, $^3J_{H,H'}$ = 12.6, 3.7 Hz, 1H, Cy C*H*), 2.29 (*qd*, $^2J_{H,H'}$ = 12.7 Hz, $^3J_{H,H'}$ = 12.7, 3.5 Hz, 2H, Cy CH_2), 2.14-1.72 (*m*, 3H, Cy CH_2), 1.57 (*m*, 2H, Cy CH_2), 1.47-1.18 (*m*, 3H, Cy CH_2); **[Minor Rotamer**, *observable signals***]** 8.31 (*br d*, $^3J_{H,H'}$ = 8.4 Hz, 1H, Ar–*H*), 7.66-7.59 (*br m*, 1H, Ar–*H*), 7.59-7.51 (*br m*, 1H, Ar–*H*), 4.48 (*bs*, 2H, ArCH_2Ar), 3.81 (*br t*, $^3J_{H,H'}$ = 13.6 Hz, 1H, Cy C*H*). **^{13}C{^{1}H} NMR** (101 MHz, $CDCl_3$): *δ* **[Major rotamer]** 141.9 (*C*, arom.), 141.1 (*C*, arom.), 135.7 (*C*, arom.), 134.3 (*C*, arom.), 132.7 (*C*, arom.), 130.2 (*C*H, arom.), 129.2 (*C*H, arom.), 128.7 (*C*H, arom.), 128.5 (*C*H, arom.), 126.8 (*C*, arom.), 126.6 (*C*H, arom.), 126.0 (*C*H, arom.), 124.7 (*C*H, arom.), 124.6 (*C*H, arom.), 41.9 (Cy *C*H), 41.6 (Ar*C*H_2Ar), 31.4 (Cy *C*H_2), 27.8 (Cy *C*H_2), 26.5 (Cy *C*H_2). **HRMS** (MALDI): Calcd. for $C_{23}H_{24}$ *m/z* 300.1873, found *m/z* 300.1881 $[M]^+$.

2-Benzyl-1-cyclopentylnaphthalene (6aba)

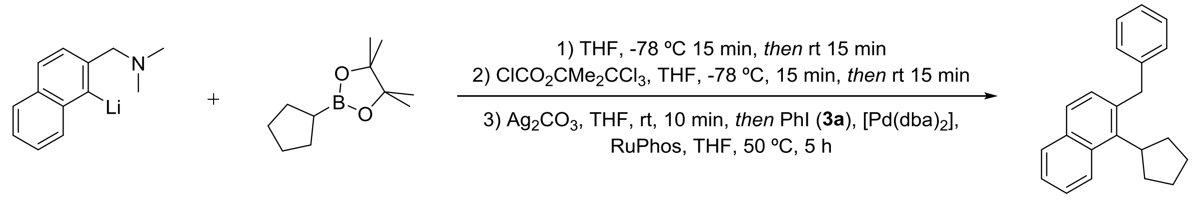

Prepared following general procedure A on a 300 μmol (34 μL) scale. Flash column chromatography on silica gel (pentane) afforded the product as a colorless oil. Yield: 81 mg (94%). ***R*f** (petrol ether:ethyl acetate = 91:9) = 0.60. **IR** (liquid film, cm^{-1}): 3050, 3025, 2950, 2866, 1599, 1509, 1493, 1451, 1364, 1029, 907, 828, 721, 695, 567, 434. **^{1}H NMR** (400 MHz, $CDCl_3$, 25 ºC): *δ* (ppm) 8.20-8.11 (m, 1H, Ar–*H*), 7.94-7.83 (*m*, 1H, Ar–*H*), 7.71 (*d*, $^3J_{H,H'}$ = 8.3 Hz, 1H, Ar–*H*), 7.53-7.41 (*m*, 2H, Ar–*H*), 7.36 (*d*, $^3J_{H,H'}$ = 8.3 Hz, 1H, Ar–*H*), 7.33-7.25 (*m*, 2H, Ar–*H*), 7.24-7.12 (*m*, 3H, Ar–*H*), 4.31 (*s*, 2H, ArC*H*2Ar), 3.87 (*p*, $^3J_{H,H'}$ = 9.7 Hz, 1H, *c*-pentyl C*H*), 2.30-2.14 (*m*, 2H, *c*-pentyl C*H*2), 2.14-2.01 (*m*, 2H, *c*-pentyl C*H*2), 1.95-1.71 (*m*, 4H, *c*-pentyl C*H*2). **^{13}C{^{1}H} NMR** (101 MHz, $CDCl_3$, 25 ºC): *δ* (ppm) 141.7 (*C*, arom.), 139.5 (*C*, arom.), 136.2 (*C*, arom.), 134.1 (*C*, arom.), 131.8 (*C*, arom.), 129.9 (*C*H, arom.), 129.5 (*C*H, arom.), 128.6 (*C*H, arom.), 128.5 (*C*H, arom.), 126.9 (*C*H, arom.), 126.0 (*C*H, arom.), 125.6 (*C*H, arom.), 124.9 (*C*H, arom.), 124.7 (*C*H, arom.), 41.2 (*c*-pentyl *C*H), 41.1 (Ar*C*H2Ar), 32.7 (*c*-pentyl *C*H2), 27.6 (*c*-pentyl *C*H2). **HRMS** (MALDI): Calcd. for $C_{22}H_{22}Na$ *m*/*z* 309.1614, found *m*/*z* 309.1621 $[M+Na]^+$.

2-Benzyl-1-cyclobutylnaphthalene (6aca)

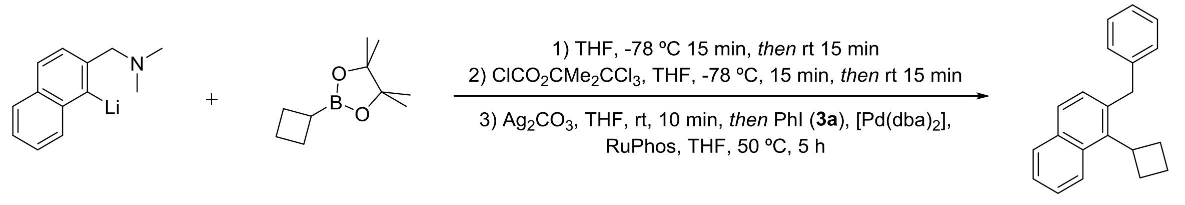

Prepared following general procedure A on a 300 μmol (34 μL) scale. Flash column chromatography on silica gel (pentane) afforded the product as a colorless oil. Yield: 72 mg (88%). ***R*f** (petrol ether:ethyl acetate = 91:9) = 0.60. **IR** (liquid film, cm^{-1}): 3050, 3025, 2939, 2866, 1599, 1565, 1508, 1493, 1452, 1029, 907. 828, 800, 756, 723, 695, 650, 456, 431. **^{1}H NMR** (400 MHz, $CDCl_3$, 25 ºC): *δ* (ppm) 8.41 (*d*, $^3J_{H,H'}$ = 8.9 Hz, 1H, Ar-*H*), 7.85 (*dd*, $^3J_{H,H'}$ = 7.9 Hz, $^4J_{H,H'}$ = 1.6 Hz, 1H, Ar–*H*), 7.66 (*d*, $^3J_{H,H'}$ = 8.3 Hz, 1H, Ar–*H*), 7.55-7.49 (*m*, 1H, Ar–*H*), 7.49-7.43 (*m*, 1H, Ar–*H*), 7.34-7.17 (*m*, 4H, Ar–*H*), 7.16-7.09 (*m*, 2H, Ar–*H*),

4.40 (*p*, $^3J_{H,H'}$ = 9.5 Hz, 1H, *c*-Bu C*H*), 4.30 (*s*, 2H, ArC*H*$_2$Ar), 2.78-2.62 (*pd*, $^2J_{H,H'}$ = 9.7 Hz, $^3J_{H,H'}$ = 9.7, 2.4 Hz, 2H, *c*-Bu C*H*$_2$), 2.58-2.44 (*m*, 2H, *c*-Bu C*H*$_2$), 2.22-1.99 (*m*, 2H, *c*-Bu C*H*$_2$). **^{13}C{^{1}H} NMR** (101 MHz, $CDCl_3$, 25 ºC): *δ* (ppm) 141.8 (*C*, arom.), 139.0 (*C*, arom.), 135.5 (*C*, arom.), 133.6 (*C*, arom.), 132.8 (*C*, arom.), 130.0 (*C*H, arom.), 129.1 (*C*H, arom.), 128.8 (*C*H, arom.), 128.5 (*C*H, arom.), 126.9 (*C*H, arom.), 126.0 (*C*H, arom.), 125.4 (*C*H, arom.), 125.1 (*C*H, arom.), 124.8 (*C*H, arom.), 40.4 (Ar*C*H$_2$Ar), 38.1 (c-Bu *C*H), 30.9 (*c*-Bu *C*H$_2$), 18.6 (*c*-Bu *C*H$_2$). **HRMS** (MALDI): Calcd. for $C_{21}H_{20}Na$ *m*/*z* 295.1457, found *m*/*z* 295.1465 $[M+Na]^+$.

2-Benzyl-1-cyclopropylnaphthalene (6ada)

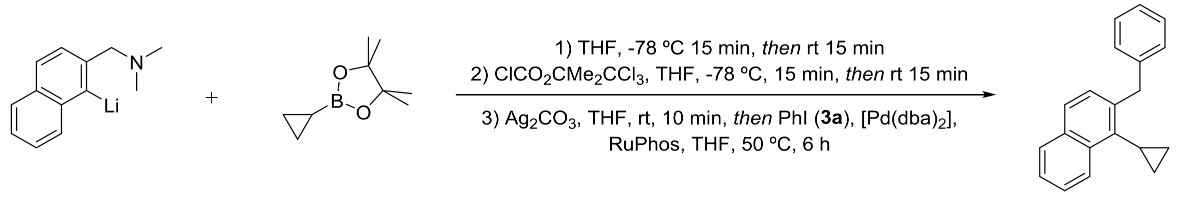

(prepared from **1a**) **2d** **6ada**

Prepared following general procedure A on a 300 µmol (34 µL) scale. Flash column chromatography on silica gel (hexane) afforded the product as a colorless oil. Yield: 64 mg (83%). ***R*$_f$** (petrol ether:ethyl acetate = 91:9) = 0.60. **IR** (liquid film, cm^{-1}): 3050, 3025, 3000, 1752, 1599, 1508, 1493, 1451, 1275, 1136, 1029, 906, 827, 802, 757, 741, 719, 697, 574, 546, 463, 423. **^{1}H NMR** (400 MHz, $CDCl_3$, 25 ºC): [*Note*: purified product contains a minor inseparable unidentified impurity as determined by ^{1}H NMR, e.g. 2.00 ppm (*s*).] *δ* (ppm) 8.63 (*dq*, $^3J_{H,H'}$ = 8.5 Hz, $^4J_{H,H'}$ = 0.9 Hz, 1H, Ar–*H*), 7.84 (*d*, $^3J_{H,H'}$ = 7.8 Hz, 1H, Ar–*H*), 7.71 (*d*, $^3J_{H,H'}$ = 8.4 Hz, 1H, Ar–*H*), 7.60-7.53 (*ddd*, $^3J_{H,H'}$ = 8.1, 6.7 Hz, $^4J_{H,H'}$ = 1.3 Hz, 1H, Ar–*H*), 7.51-7.45 (*ddd*, $^3J_{H,H'}$ = 8.5, 6.8 Hz, $^4J_{H,H'}$ = 1.5 Hz, 1H, Ar–*H*), 7.37-7.13 (*m*, 6H, Ar–*H*), 4.52 (*s*, 2H, ArC*H*$_2$Ar), 2.12-2.01 (*m*, 1H, *c*-Pr C*H*), 1.32-1.21 (*m*, 2H, *c*-Pr C*H*$_2$), 0.83-0.72 (*m*, 2H, *c*-Pr C*H*$_2$). **^{13}C{^{1}H} NMR** (101 MHz, $CDCl_3$, 25 ºC): *δ* (ppm) 141.9 (*C*, arom.), 138.8 (*C*, arom.), 136.2 (*C*, arom.), 134.3 (*C*, arom.), 132.8 (*C*, arom.), 129.04 (*C*H, arom.), 129.03 (*C*H, arom.), 128.51 (*C*H, arom.), 128.47 (*C*H, arom.), 127.2 (*C*H, arom.), 125.9 (*C*H, arom.), 125.7 (*C*H, arom.), 125.6 (*C*H, arom.), 125.1 (*C*H, arom.), 39.5 (Ar*C*H$_2$Ar), 11.2 (*c*-Pr *C*H), 9.0 (*c*-Pr *C*H$_2$). **HRMS** (MALDI): Calcd. for $C_{20}H_{18}Na$ *m*/*z* 281.1301, found *m*/*z* 281.1309 $[M+Na]^+$.

2-Benzyl-1-isopropylnaphthalene (6aea)

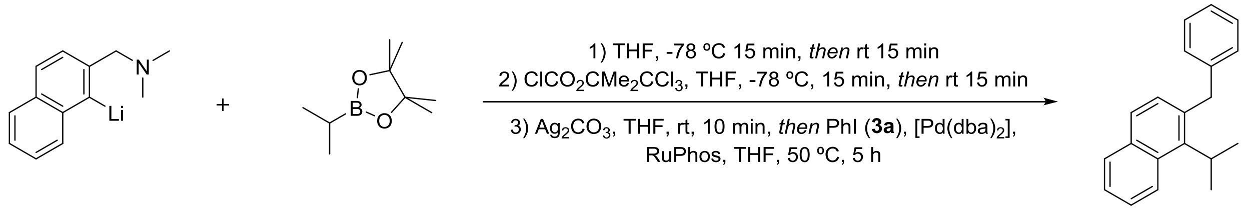

(prepared from **1a**) **2e** **6aea**

Prepared following general procedure A on a 300 μmol (34 μL) scale. Flash column chromatography on silica gel (hexane) afforded the product as a colorless oil. Yield: 67 mg (84%). ***R*f** (hexane) = 0.20. **IR** (liquid film, cm^{-1}): 3051, 3026, 2927, 1600, 1494, 1452, 1029, 829, 802, 758, 720, 696. **^{1}H NMR** (400 MHz, $CDCl_3$, 25 ºC): δ (ppm) 8.34 (*d*, $^3J_{H,H'}$ = 8.4 Hz, 1H, Ar–*H*), 7.87 (*d*, $^3J_{H,H'}$ = 7.5 Hz, 1H, Ar–*H*), 7.70 (*d*, $^3J_{H,H'}$ = 8.4 Hz, 1H, Ar–*H*), 7.51-7.42 (*m*, 2H, Ar–*H*), 7.34 (*br d*, $^3J_{H,H'}$ = 7.3 Hz, 1H, Ar–*H*), 7.29 (*t*, $^3J_{H,H'}$ = 7.2 Hz, 2H, Ar–*H*), 7.20 (*t*, $^3J_{H,H'}$ = 7.2 Hz, 1H, Ar–*H*), 7.15 (*d*, $^3J_{H,H'}$ = 7.6 Hz, 2H, Ar–*H*), 4.29 (*br s*, 2H, ArC*H*$_2$Ar), 3.79 (*br s*, 1H, ArC*H*(CH_3)$_2$), 1.5 (*d*, $^3J_{H,H'}$ = 7.3 Hz, 6H, C*H*$_3$). **^{13}C{^{1}H} NMR** (101 MHz, $CDCl_3$, 25 ºC): δ (ppm) δ 141.9 (*br*, *C*, arom.), 141.5 (*br*, *C*, arom.), 135.0 (*br*, *C*, arom.), 134.2 (*br*, *C*, arom.), 132.1 (*br*, *C*, arom.), 130.0 (*br*, *C*H, arom.), 129.3 (*C*H, arom.), 128.6 (*br*, *C*H, arom.), 128.4 (*C*H, arom.), 126.8 (*C*H, arom.), 126.2 (*br*, *C*H, arom.), 126.0 (*C*H, arom.), 124.9 (*br*, *C*H, arom.), 124.7 (*C*H, arom.), 40.8 (*br*, Ar*C*H_2Ar), 29.9 (*br*, Ar*C*H(CH_3)$_2$), 22.3 (*C*H_3). **HRMS** (MALDI): Calcd. for $C_{20}H_{20}Na$ *m/z* 283.1457, found *m/z* 283.1466 $[M+Na]^+$.

2-Benzyl-1-hexylnaphthalene (6afa)

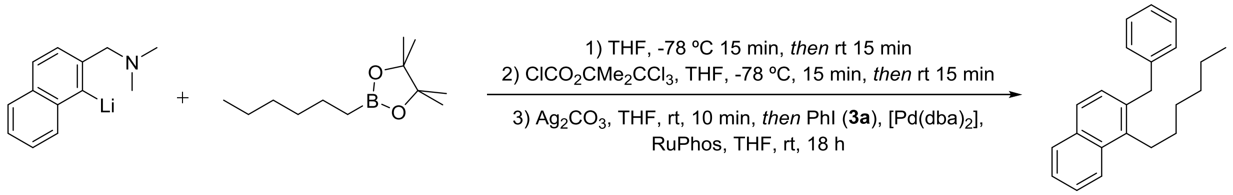

(prepared from **1a**) **2f** **6afa**

Prepared following general procedure A, at room temperature and during 18 h for the cross-coupling step, on a 300 μmol (34 μL) scale. Flash column chromatography on silica gel (hexane) afforded the product as a colorless oil. Yield: 60 mg (66%). ***R*f** (petrol ether:ethyl acetate = 91:9) = 0.70. **IR** (liquid film, cm^{-1}): 3026, 2954, 2923, 2854, 1599, 1511, 1494, 1452, 1180, 1029, 908, 861, 829, 799, 784, 758, 738, 722, 695, 555, 466, 426. **^{1}H NMR** (400 MHz, $CDCl_3$, 25 ºC): δ (ppm) 8.09 (*d*, $^3J_{H,H'}$ = 8.5 Hz, 1H, Ar–*H*), 7.86 (*dd*,

$^3J_{H,H'}$ = 8.3 Hz, $^4J_{H,H'}$ = 1.4 Hz, 1H, Ar–*H*), 7.54 (*ddd*, $^3J_{H,H'}$ = 8.5, 6.8 Hz, $^4J_{H,H'}$ = 1.5 Hz, 1H, Ar–*H*), 7.47 (*ddd*, $^3J_{H,H'}$ = 8.0, 6.8 Hz, $^4J_{H,H'}$ = 1.2 Hz, 1H, Ar–*H*), 7.36-7.27 (*m*, 3H, Ar–*H*), 7.25-7.15 (*m*, 3H, Ar–*H*), 4.26 (*s*, 2H, ArC*H*$_2$Ar), 3.15-3.06 (m, 2H, ArC*H*$_2$ (CH$_2$)$_4$CH$_3$), 1.66-1.55 (*m*, 2H, ArCH$_2$C*H*$_2$(CH$_2$)$_3$CH$_3$), 1.54-1.45 (*m*, 2H, Ar(CH$_2$)$_2$C*H*$_2$(CH$_2$)$_2$CH$_3$), 1.42-1.28 (*m*, 4H, Ar(CH$_2$)$_3$C*H*$_2$C*H*$_2$CH$_3$), 1.01-0.87 (*m*, 3H, Ar(CH$_2$)$_3$CH$_2$CH$_2$C*H*$_3$). **^{13}C{^{1}H} NMR** (101 MHz, CDCl$_3$, 25 ºC): δ (ppm) 141.4 (*C*, arom.), 136.9 (*C*, arom.), 135.3 (*C*, arom.), 133.0 (*C*, arom.), 132.6 (*C*, arom.), 129.3 (*C*H, arom.), 128.9 (*C*H, arom.), 128.8 (*C*H, arom.), 128.6 (*C*H, arom.), 126.4 (*C*H, arom.), 126.1 (*C*H, arom.), 126.0 (*C*H, arom.), 125.0 (*C*H, arom.), 124.3 (*C*H, arom.), 39.6 (Ar*C*H$_2$Ar), 31.9 (Ar(CH$_2$)$_3$*C*H$_2$*C*H$_2$CH$_3$), 30.9 (ArCH$_2$*C*H$_2$(CH$_2$)$_3$CH$_3$), 30.2 (Ar(CH$_2$)$_2$*C*H$_2$(CH$_2$)$_2$CH$_3$), 29.1 (Ar*C*H$_2$(CH$_2$)$_4$CH$_3$), 22.9 (Ar(CH$_2$)$_3$CH$_2$*C*H$_2$CH$_3$), 14.3 (Ar(CH$_2$)$_5$*C*H$_3$). **HRMS** (MALDI): Calcd. for $C_{23}H_{26}Na$ *m/z* 325.1927, found *m/z* 325.1939 [M+Na]$^+$.

1,2-bis(1-hexylnaphthalen-2-yl)ethane (S6af, homocoupling by-product obtained along with coupling product 6afa)

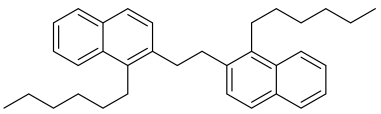

S6af

Homocoupling product obtained along with **6afa**. Flash column chromatography on silica gel (hexane) afforded the product as a colorless oil. Yield: 11 mg (8%). ***R*$_f$** (petrol ether:ethyl acetate = 91:9) = 0.72. **^{1}H NMR** (400 MHz, CDCl$_3$, 25 ºC): δ (ppm) 8.09 (*dd*, $^3J_{H,H'}$ = 8.5 Hz, $^4J_{H,H'}$ = 1.1 Hz, 2H, Ar–*H*), 7.82 (*dd*, $^3J_{H,H'}$ = 8.0 Hz, $^4J_{H,H'}$ = 1.4 Hz, 2H, Ar–*H*), 7.68 (*d*, $^3J_{H,H'}$ = 8.4 Hz, 2H, Ar–*H*), 7.51 (*ddd*, $^3J_{H,H'}$ = 8.4, 6.8 Hz, $^4J_{H,H'}$ = 1.5 Hz, 2H, Ar–*H*), 7.44 (*ddd*, $^3J_{H,H'}$ = 8.0, 6.8 Hz, $^4J_{H,H'}$ = 1.2 Hz, 2H, Ar–*H*), 7.38 (*d*, $^3J_{H,H'}$ = 8.4 Hz, 2H, Ar–*H*), 3.18-3.08 (m, 8H, 4 ArC*H*$_2$), 1.73-1.60 (*m*, 4H, 2 non-benzylic –C*H*$_2$–), 1.60-1.47 (*m*, 4H, 2 non-benzylic –C*H*$_2$–), 1.42-1.28 (*m*, 8H, 4 non-benzylic –C*H*$_2$–), 0.99-0.83 (*m*, 6H, 2 –C*H*$_3$). **^{13}C{^{1}H} NMR** (101 MHz, CDCl$_3$, 25 ºC): δ (ppm) 136.7 (2 *C*, arom.), 136.0 (2 *C*, arom.), 132.9 (2 *C*, arom.), 132.5 (2 *C*, arom.), 128.8 (2 *C*H, arom.), 128.4 (2 *C*H, arom.), 126.5 (2 *C*H, arom.), 126.0 (2 *C*H, arom.), 124.9 (2 *C*H, arom.), 124.2 (2 *C*H, arom.), 36.1 (2 Ar*C*H$_2$), 32.0 (2 non-benzylic –*C*H$_2$–), 31.5 (2 non-benzylic –*C*H$_2$–), 30.3 (2 non-benzylic –*C*H$_2$–), 28.8 (2 Ar*C*H$_2$), 22.9 (2 non-benzylic –*C*H$_2$–), 14.3 (2 –*C*H$_3$). **HRMS** (MALDI): Calcd. for $C_{34}H_{42}Na$ *m/z* 473.3179, found *m/z* 473.3188 [M+Na]$^+$.

1-(2-Benzylphenyl)adamantane (6bga)

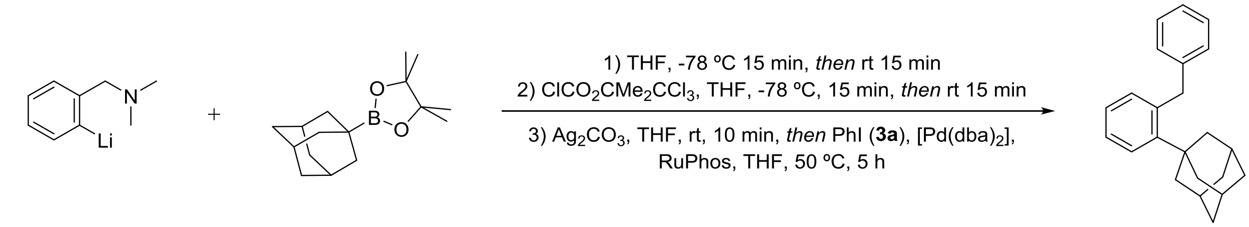

(prepared from **1b**) **2g** **6bga**

Prepared following general procedure A on a 300 µmol (34 µL) scale. Flash column chromatography on silica gel (hexane) afforded the product as a white solid. Yield: 80 mg (88%). **m.p.** = 124.9-126.3 ºC. ***R*f** (petrol ether:ethyl acetate = 91:9) = 0.70. **IR** (liquid film, cm^{-1}): 3021, 2903, 2884, 2849, 1602, 1494, 1451, 1367, 1346, 1315, 1096, 1072, 1027, 926, 756, 739, 727, 698, 622. **^{1}H NMR** (400 MHz, $CDCl_3$, 25 ºC): δ (ppm) 7.43 (*dd*, $^3J_{H,H'}$ = 8.1 Hz, $^4J_{H,H'}$ = 1.4 Hz, 1H, Ar–*H*), 7.34-7.27 (*m*, 2H, Ar–*H*). 7.26-7.18 (*m*, 2H, Ar–*H*), 7.14 (*dd*, $^3J_{H,H'}$ = 7.5 Hz, $^4J_{H,H'}$ = 1.4 Hz, 1H, Ar–*H*), 7.13-7.08 (*m*, 2H, Ar–*H*), 7.03 (*dd*, $^3J_{H,H'}$ = 7.6 Hz, $^4J_{H,H'}$ = 1.6 Hz, 1H, Ar–*H*), 4.46 (*s*, 2H, ArC*H*$_2$Ar), 2.22-2.07 (*m*, 9H, Ad C*H* and Ad C*H*$_2$), 1.80 (*t*, $^3J_{H,H'}$ = 3.1 Hz, 6H, Ad C*H*). **^{13}C{^{1}H} NMR** (101 MHz, $CDCl_3$, 25 ºC): δ (ppm) 148.4 (*C*, arom.), 142.9 (*C*, arom.), 139.1 (*C*, arom.), 133.7 (*C*H, arom.), 129.2 (*C*H, arom.), 128.4 (*C*H, arom.), 126.4 (*C*H, arom.), 126.2 (*C*H, arom.),126.0 (*C*H, arom.), 125.9 (*C*H, arom.), 42.5 (Ad *C*H_2), 40.1 (Ar*C*H_2Ar), 38.0 (Ad *C*), 29.4 (Ad *C*H). **HRMS** (MALDI): Calcd. for $C_{23}H_{26}Na$ *m*/*z* 325.1927, found *m*/*z* 325.1935 $[M+Na]^+$.

2-Benzyl-1-phenylnaphthalene (6aha)

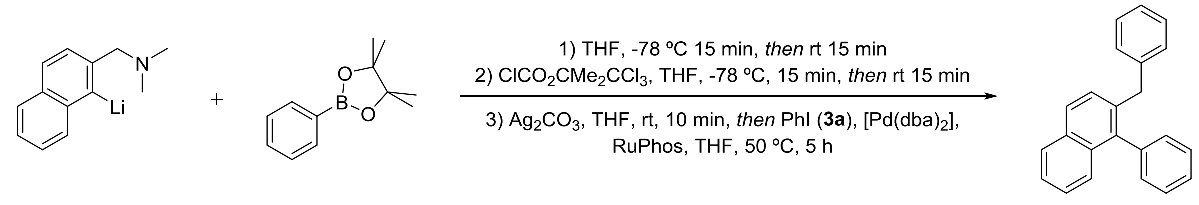

(prepared from **1a**) **2h** **6aha**

Prepared following general procedure A on a 300 µmol (34 µL) scale. Flash column chromatography on silica gel (gradient, toluene:hexane = 2.5:97.5 to 10: 90) afforded the product as a white solid. Yield: 67 mg (76%). ***R*f** (toluene:hexane = 5:95) = 0.21. **M.p.**: 79.7-81.2 °C. **IR** (liquid film, cm^{-1}): 3024, 2923, 1596, 1492, 1453, 1071, 1029, 807, 766, 744, 700. **^{1}H NMR** (400 MHz, $CDCl_3$, 25 ºC): δ (ppm) 7.84 (*d*, $^3J_{H,H'}$ = 7.3 Hz, 1H, Ar–*H*), 7.80 (*d*, $^3J_{H,H'}$ = 8.5 Hz, 1H, Ar–*H*), 7.48-7.40 (*m*, 5H, Ar–*H*), 7.38 (*d*, $^3J_{H,H'}$ = 8.5 Hz, 1H, Ar–*H*), 7.36-7.32 (*m*, 1H, Ar–*H*), 7.28-7.25 (*m*, 2H, Ar–*H*), 7.22-7.18 (*m*, 2H, Ar–*H*), 7.16-7.12 (*m*, 1H, Ar–*H*), 7.01 (*d*, $^3J_{H,H'}$ = 7.1 Hz, 2H, Ar–*H*), 3.94 (*s*, 2H, ArC*H*$_2$Ar). **^{13}C{^{1}H} NMR** (101 MHz, $CDCl_3$,

25 ºC): δ (ppm) 141.6 (*C*, arom.), 139.4 (*C*, arom.), 138.7 (*C*, arom.), 136.0 (*C*, arom.), 133.2 (*C*, arom.), 132.3 (*C*, arom.), 130.5 (*C*H, arom.), 129.0 (*C*H, arom.), 128.4 (*C*H, arom.), 128.34 (*C*H, arom.), 128.32 (*C*H, arom.), 127.9 (*C*H, arom.), 127.8 (*C*H, arom.), 127.3 (*C*H, arom.), 126.8 (*C*H, arom.), 126.0 (*C*H, arom.), 125.9 (*C*H, arom.), 125.3 (*C*H, arom.), 39.7 (Ar*C*H$_2$Ar). **HRMS** (MALDI): Calcd. for $C_{23}H_{18}$ *m/z* 294.1403, found *m/z* 294.1412 $[M]^+$.

tert-Butyl (_S_)-4-(2-benzylnaphthalen-1-yl)-6-phenylhexanoate ((_S_)-6aia)

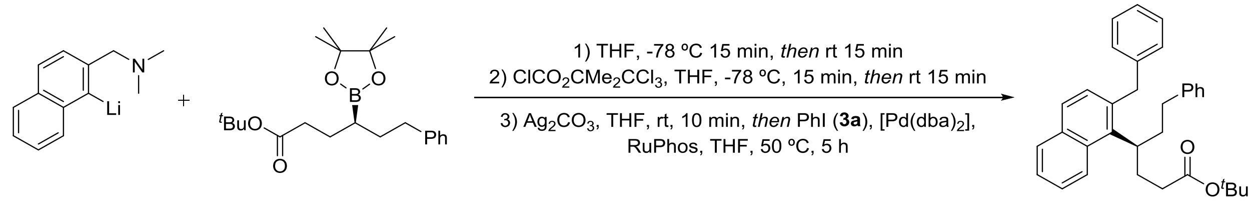

(prepared from **1a**) **(*S*)-2i** **(*S*)-6aia**

Prepared following general procedure A on a 300 µmol (34 µL) scale. Flash column chromatography on silica gel (gradient, diethyl ether:pentane = 1:99 to 10:90) afforded the product as a colorless oil. Yield: 103 mg (74%). $[\alpha]_D^{23}$ = +4.0 (*c* 1.0, CHCl$_3$). ***R*$_f$** (diethyl ether:pentane = 10:90) = 0.34. **IR** (liquid film, cm^{-1}): 2975, 2929, 1723, 1494, 1453, 1365, 1145, 721, 697. **^{1}H NMR** (400 MHz, CDCl$_3$, 25 ºC): [*Note:* product exists as a mixture of two rotamers observable by ^{1}H NMR, 88:12] δ (ppm) **[Major rotamer]** 8.34-8.30 (*m*, 1H, Ar–*H*), 7.94-7.90 (*m*, 1H, Ar–*H*), 7.78 (*d*, $^3J_{H,H'}$ = 8.3 Hz, 1H Ar–*H*), 7.52-7.47 (*m*, 2H, Ar–*H*), 7.36-7.32 (*m*, 2H, Ar–*H*), 7.30-7.25 (*m*, 3H, Ar–*H*), 7.22-7.16 (*m*, 3H, Ar–*H*), 7.03 (*d*, $^3J_{H,H'}$ = 7.2 Hz, Ar–*H*), 4.30 (q_{AB}, d_A, $^2J_{H,H'}$ = 15.9 Hz, 1H, ArC*H*$_A$H$_B$Ar), 4.15 (q_{AB}, d_B, $^2J_{H,H'}$ = 15.9 Hz, 1H, ArCH$_A$*H*$_B$Ar), 3.51 (app. *p*, $^3J_{H,H'}$ = 7.3 Hz, 1H, Cy C*H*), 2.47-2.29 (*m*, 4H, Cy C*H*$_2$), 2.26-1.92 (*m*, 4H, Cy C*H*$_2$), 1.47 (*s*, 9H, –O(C*H*$_3$)$_3$); **[Minor Rotamer**, *observable signals***]** 8.20-8.17 (*m*, 1H, Ar–*H*), 7.90-7.87 (*m*, 1H, Ar–*H*), 7.73 (*d*, $^3J_{H,H'}$ = 8.5 Hz, 1H, Ar–*H*), 7.07 (*d*, $^3J_{H,H'}$ = 7.5 Hz, 2H, Ar–*H*), 4.50 (s, 2H, ArC*H*$_2$Ar), 4.10 (app. *p*, $^3J_{H,H'}$ = 7.6 Hz, 1H, Cy C*H*), 2.74-2.66 (*m*, 2H, Cy C*H*$_2$), 2.61-2.53 (*m*, 2H, Cy C*H*$_2$), 1.45 (*s*, 9H, −O(C*H*$_3$)$_3$). **^{13}C{^{1}H} NMR** (101 MHz, CDCl$_3$, 25 ºC): δ (ppm) **[Major rotamer]** 173.1 (*C*=O), 142.5 (*C*, arom.), 141.6 (*C*, arom.), 138.6 (*C*, arom.), 136.9 (*C*, arom.), 134.2 (*C*, arom.), 132.3 (*C*, arom.), 130.0 (*C*H, arom.), 129.3 (*C*H, arom.), 128.9 (*C*H, arom.), 128.5 (*C*H, arom.), 128.4 (*C*H, arom.), 128.3 (*C*H, arom.), 127.2 (*C*H, arom.), 126.2 (*C*H, arom.), 125.9 (*C*H, arom.), 125.7 (*C*H, arom.), 125.2 (*C*H, arom.), 124.8 (*C*H, arom.), 80.1 (O(*C*H$_3$)$_3$), 41.2 (Ar*C*H$_2$Ar), 40.5 (Ar*C*H(CH$_2$R)$_2$), 37.9 (*C*H$_2$), 34.9 (*C*H$_2$), 34.2 (*C*H$_2$), 30.0 (*C*H$_2$), 28.2 (*C*H$_2$). **HRMS** (MALDI): Calcd. for $C_{33}H_{36}O_2Na$ *m/z* 487.2608, found *m/z* 487.2601 $[M+Na]^+$. **HPLC**: Chiralpak IB, *two in series* (hexane:2-propanol = 99.5:0.5, flow rate 0.3 mL/min, λ = 210 nm), retention times t_R(minor) = 74.5 min, t_R(major) = 78.3 min; 5:95 e.r.; 89.7%ee.

Figure S4: HPLC chromatograms and data tables for compounds **(±)-6aia** and **(*S*)-6aia**, respectively.

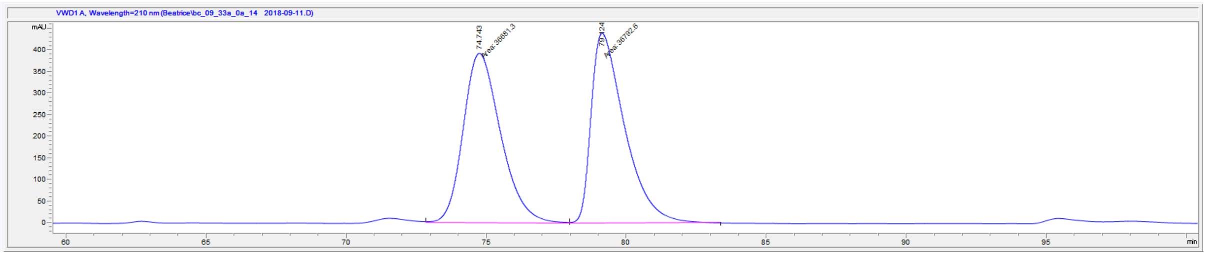

#	Time	Type	Area	Height	Width	Area%	Symmetry
1	74.743	MM	36681.3	393.3	1.5545	49.924	0.738
2	79.124	MM	36792.6	442	1.3873	50.076	0.474

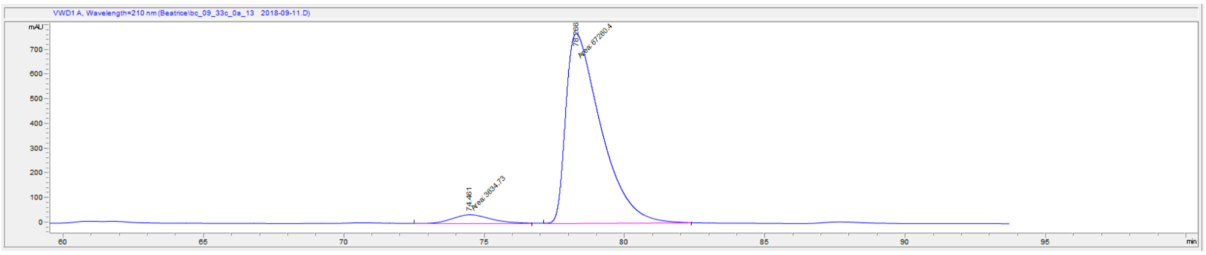

#	Time	Type	Area	Height	Width	Area%	Symmetry
1	74.461	MM	3634.7	36.1	1.6797	5.127	0.804
2	78.266	MM	67260.4	772.7	1.4508	94.873	0.409

(*S*)-1-(6-Azido-1-phenylhexan-3-yl)-2-benzylbenzene ((*S*)-6bja)

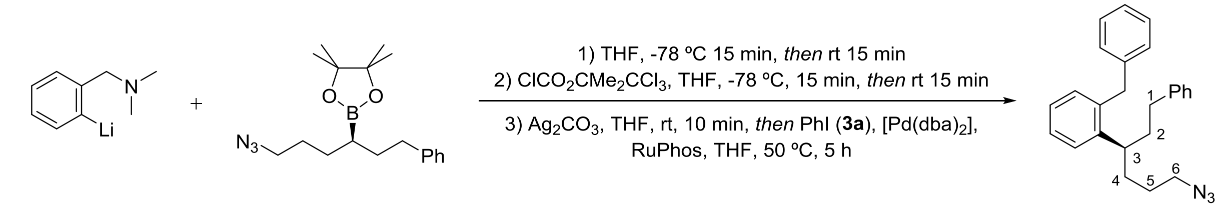

(prepared from **1b**) **(*S*)-2j** **(*S*)-6aja**

Prepared following general procedure A on a 300 μmol (34 μL) scale. Yield: 45% (by ^{1}H NMR using dibromomethane as internal standard). Purification of the product was not achieved by flash column chromatography (silica, hexane:toluene:diethyl ether = 88:9:3, $\boldsymbol{R}_\mathbf{f}$ (petrol ether:ethyl acetate = 91:9) = 0.64) because it co-elutes with unidentified impurities. 1 mg of pure material were obtained as a colourless oil

using preparative HPLC (Kromasil 60-5SIL, hexane:2-propanol = 99:1, flow rate 18.0 mL/min, λ = 254 nm, retention time t_R = 7 min). **$[\alpha]_D^{23}$** = −0.6 (*c* 0.052, $CHCl_3$). **IR** (liquid film, cm^{-1}): 3061, 3025, 2922, 2852, 2093, 1602, 1494, 1452, 1349, 1260, 1156, 1075, 1029, 802, 749, 733, 697, 625, 615, 557, 467. **^{1}H NMR** (400 MHz, $CDCl_3$, 25 °C): δ (ppm) 7.30-7.21 (*m*, 6H, Ar–*H*), 7.20-7.13 (*m*, 4H, Ar–*H*), 7.02 (*ddd*, $^3J_{H,H'}$ = 15.6, 8.0 Hz, $^4J_{H,H'}$ = 1.3 Hz, 4H, Ar–*H*), 3.94 (q_{AB}, d_A, $^2J_{H,H'}$ = 15.6 Hz, 1H, ArCH_2Ar), 3.88 (q_{AB}, d_B, $^2J_{H,H'}$ = 15.7 Hz, 1H, ArCH_2Ar), 2.94 (*t*, $^3J_{H,H'}$ = 6.9 Hz, 2H, Ph–C$^{(1)}H_2$–), 2.84 (*tt*, $^3J_{H,H'}$ = 8.5, 6.0 Hz, 1H, –ArC$^{(3)}H$–), 2.33 (*t*, $^3J_{H,H'}$ = 8.0 Hz, 2H, –C$^{(6)}H_2$–N_3), 1.92-1.83 (*m*, 1H, –C$^{(5)}H_2$–), 1.82-1.72 (*m*, 1H, –C$^{(5)}H_2$–), 1.70-1.61 (*m*, 1H, –C$^{(4)}H_2$–), 1.51-1.40 (*m*, 4H, –C$^{(4)}H_2$–), 1.22-1.10 (*m*, 1H, –C$^{(2)}H_2$–). **^{13}C{^{1}H} NMR** (101 MHz, $CDCl_3$, 25 °C): δ (ppm) 144.0 (*C*, arom.), 142.5 (*C*, arom.), 141.3 (*C*, arom.), 138.8 (*C*, arom.), 131.3 (*C*H, arom.), 128.9 (*C*H, arom.), 128.53 (*C*H, arom.), 128.50 (*C*H, arom.), 128.45 (2 *C*H, arom.), 127.3 (*C*H, arom.), 126.2 (*C*H, arom.), 126.1 (*C*H, arom.), 125.9 (*C*H, arom.), 51.6 ($C^{(1)}H_2$), 39.8 (ArCH_2Ar), 38.8 ($C^{(3)}$H and $C^{(5)}H_2$), 33.7 ($C^{(6)}H_2$), 33.4 ($C^{(4)}H_2$), 26.8 ($C^{(2)}H_2$). **HRMS** (MALDI): Calcd. for $C_{25}H_{27}N_3Na$ *m/z* 392.2097, found *m/z* 392.2088 $[M+Na]^+$. **HPLC**: Chiralpak IB (hexane:2-propanol = 99.5:0.5, flow rate 0.7 mL/min, λ = 210 nm), retention times t_R(minor) = 9.7 min, t_R(major) = 10.8 min; 2:98 e.r., 95.8%ee.

Figure S5: HPLC chromatograms and data tables for compounds **(±)-6bja** and **(*S*)-6bja**, respectively.

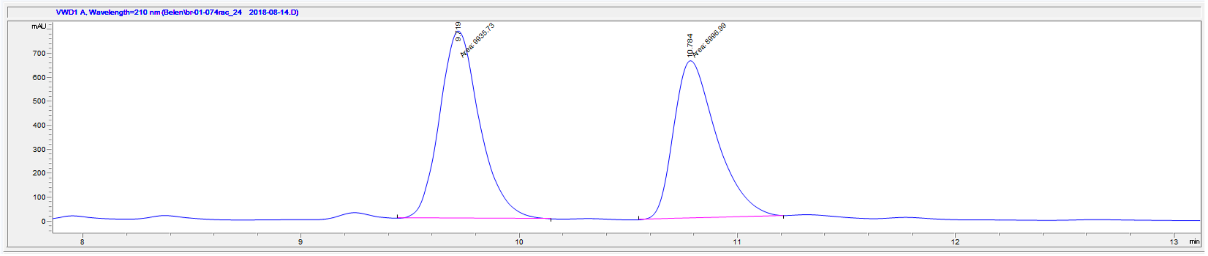

#	Time	Type	Area	Height	Width	Area%	Symmetry
1	9.719	MM	9935.7	777.1	0.2131	52.479	0.836
2	10.784	MM	8997	655.5	0.2288	47.521	0.622

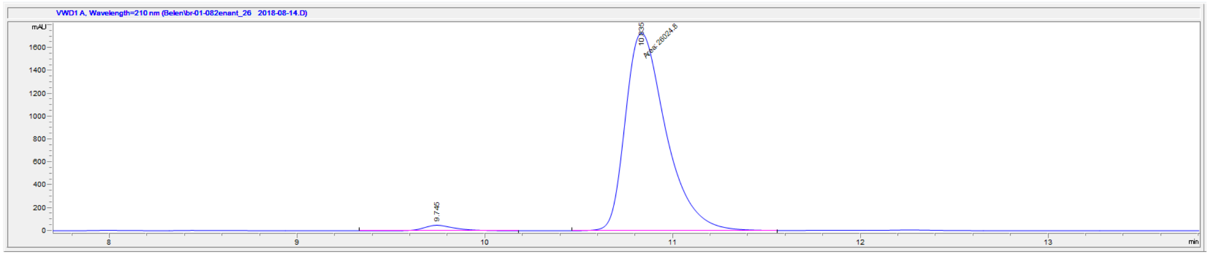

#	Time	Type	Area	Height	Width	Area%	Symmetry
1	9.745	BB	555.2	47.9	0.1724	2.089	0.687
2	10.835	MM	26024.8	1729.8	0.2507	97.911	0.636

(((2*S*,4*S*,6*S*)-6-(2-Benzylnaphthalen-1-yl)-2,4-dimethylheptyl)oxy)(*tert*-butyl)diphenylsilane (6aka)

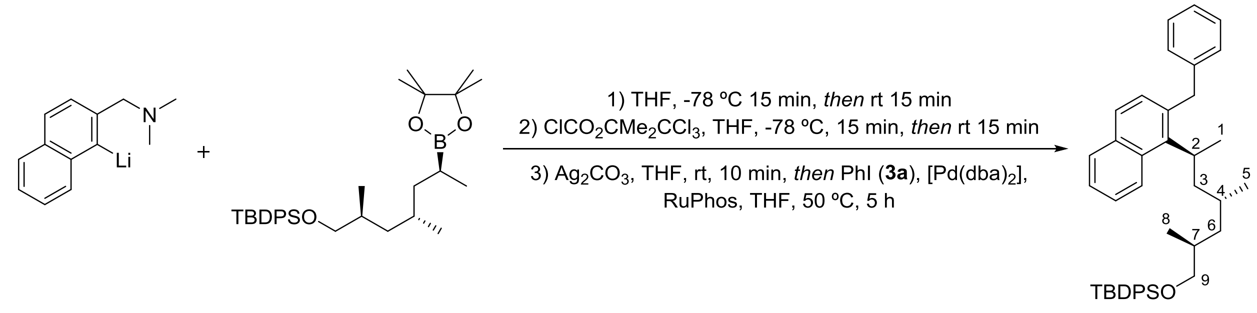

(prepared from **1a**) **2k** **6aka**

Prepared following general procedure A on a 300 μmol (34 μL) scale. Flash column chromatography on silica gel (petrol ether:ethyl acetate = 99:1) afforded the product as a colorless oil. Yield: 168 mg (93%). $\boldsymbol{R_f}$ (petrol ether: ethyl acetate = 97.5:2.5) = 0.38. **IR** (liquid film, cm^{-1}): 3070, 3049, 3026, 2955, 2928, 2856, 1601, 5190, 1567, 1509, 1494, 1471, 1454, 1388, 1378, 1361, 1110, 1086, 1029, 824, 801, 738, 699, 614, 568, 503, 488. **^{1}H NMR** (400 MHz, $CDCl_3$, 25 °C): [*Note:* **6aka** exists as a mixture of two rotamers observable by ^{1}H NMR, 81:19. The presence of a rotamer, and not a diasteromer, was confirmed by the corresponding EXSY NMR experiment] δ (ppm) **[Major rotamer]** 8.23 (*dt*, $^3J_{H,H'}$ = 6.2 Hz, $^4J_{H,H'}$ = 3.3 Hz, 1H, Ar–*H*), 7.82 (*dt*, $^3J_{H,H'}$ = 6.8 Hz, $^4J_{H,H'}$ = 3.2 Hz, 1H, Ar–*H*), 7.72-7.19 (*m*, 5H, Ar–*H*), 7.48-7.30 (*m*, 9H, Ar–H), 7.30-7.19 (*m*, 3H, Ar–H), 7.18-7.13 (*m*, 1H, Ar–H), 7.12-7.02 (*m*, 1H, Ar–H), 4.27 (q_{AB}, d_A, $^2J_{H,H'}$ = 16.0 Hz, 1H, ArC*H*$_2$Ar), 4.14 (q_{AB}, d_B, $^2J_{H,H'}$ = 16.0 Hz, 1H, ArC*H*$_2$Ar), 3.58-3.43 (*m*, 2H, Ar–C$^{(2)}$*H*Me– + –C$^{(9)}$*H*$_2$–), 3.37 (*dd*, $^3J_{H,H'}$ = 9.8, 6.8 Hz, 1H, –C$^{(9)}$*H*$_2$–), 2.13 (*ddd*, $^2J_{H,H'}$ = 13.5, $^3J_{H,H'}$ = 9.7, 3.7 Hz, 1H, –C$^{(6)}$*H*$_2$–), 1.80-1.66 (*m*, 1H, –C$^{(7)}$*H*Me–), 1.57-1.46 (*m*, 1H, –C$^{(4)}$*H*Me–), 1.45-1.35 (*m*, 1H, –C$^{(6)}$*H*$_2$–), 1.34-1.23 (*m*, 4H, –C$^{(1)}$*H*$_3$ + –C$^{(3)}$*H*$_2$–), 1.06 (*s*, 9H, –OSi(Ph)$_2$C(C*H*$_3$)$_3$), 0.96 (*d*, $^3J_{H,H'}$ = 6.7 Hz, 3H, –C$^{(8)}$*H*$_3$), 0.94-0.79 (*m*, 1H, –C$^{(3)}$*H*$_2$–), 0.68 (*d*, $^3J_{H,H'}$ = 6.4 Hz, 3H, –C$^{(5)}$*H*$_3$); **[Minor rotamer,** ***observable signals*****]** 7.76 (*br d*, $^3J_{H,H'}$ = 7.7 Hz, 1H, Ar–*H*), 7.56 (*br d*, $^3J_{H,H'}$ = 8.9, 1H, Ar–*H*), 4.39 (*d*, $^3J_{H,H'}$ = 16.3 Hz, 1H, ArC*H*$_2$Ar), 3.28-3.15 (*m*, 1H, –C$^{(9)}$*H*$_2$–), 1.03 (*br s*, 9H, –OSi(Ph)$_2$C(C*H*$_3$)$_3$). **^{13}C{^{1}H} NMR** (101 MHz, $CDCl_3$, 25 °C): δ (ppm) **[Major rotamer]** 142.2 (*C*, arom.), 141.5 (*C*, arom.), 135.8 (*C*H, arom.), 134.9 (*C*, arom.), 134.3 (*C*, arom.), 134.3 (*C*, arom.), 132.4 (*C*, arom.), 130.0 (*C*H, arom.), 129.7 (*C*H, arom.), 129.3 (*C*H, arom.), 128.7 (*C*H, arom.), 128.5 (*C*H, arom.), 127.8 (*C*H, arom.), 126.8 (*C*H, arom.), 126.6 (*C*H, arom.), 126.1 (*C*H, arom.), 124.8 (*C*H, arom.), 124.7 (*C*H, arom.), 69.2 (*C*$^{(9)}$H$_2$), 44.2 (*C*$^{(6)}$H$_2$), 42.1 (*C*$^{(3)}$H$_2$), 41.1 (Ar*C*H$_2$Ar), 33.4 (*C*$^{(7)}$H), 33.2 (*C*$^{(2)}$H), 29.4 (*C*$^{(4)}$H), 27.1 (–OSi(Ph)$_2$C(*C*H$_3$)), 20.1 (*C*$^{(5)}$H$_3$), 19.7 (*C*$^{(1)}$H$_3$), 19.5 (–OSi(Ph)$_2$*C*(CH$_3$)), 18.1 (*C*$^{(8)}$H$_3$). **HRMS** (MALDI): Calcd. for $C_{42}H_{50}OSiNa$ *m*/*z* 621.3523, found *m*/*z* 621.3530 $[M+Na]^+$.

(((2*S*,4*S*,6*R*)-6-(2-Benzylnaphthalen-1-yl)-2,4-dimethylheptyl)oxy)(*tert*-butyl)diphenylsilane (6ala)

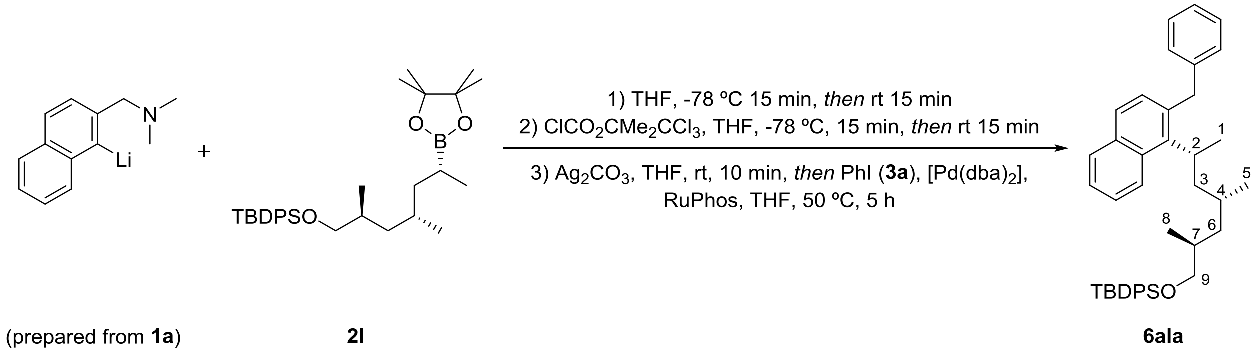

Prepared following general procedure A on a 300 μmol (34 μL) scale. Flash column chromatography on silica gel (petrol ether:ethyl acetate = 99:1) afforded parcial purification of 29 mg of product, which co-elutes with an unidentified impurity, as a colorless oil. Yield: 92% (by ^{1}H NMR, using dibromomethane as internal standard, based on the integration of the bisbenzylic protons (Nf–CH_2–Ph) for the major rotamer in the crude mixture and subsequent extrapolation regarding the observed ratio of major rotamer:minor rotamer = 86:14 obtained for the pure compound). $\boldsymbol{R_f}$ (petrol ether: ethyl acetate = 91:9) = 0.60. **IR** (liquid film, cm^{-1}): 3050, 2955, 2928, 2856, 1601, 1509, 1494, 1471, 1454, 1427, 1389, 1377, 1361, 1110, 1086, 1030, 1007, 998, 908, 824, 801, 788, 759, 738, 722, 699, 614, 570, 503, 488, 438. **^{1}H NMR** (400 MHz, $CDCl_3$, 25 °C): [*Note:* **6aia** exists as a mixture of two rotamers observable by ^{1}H NMR, 85:15. The presence of a rotamer, and not a diasteromer, was confirmed by the corresponding EXSY NMR experiment] δ (ppm) **[Major rotamer]** 8.29-8.19 (*m*, 1H, Ar–*H*), 7.87-7.76 (*m*, 1H, Ar–*H*), 7.69-7.56 (*m*, 5H, Ar–*H*), 7.45-7.32 (*m*, 8H, Ar–*H*), 7.30 (*d*, $^3J_{H,H'}$ = 8.3 Hz, 1H, Ar–*H*), 7.24-7.15 (*m*, 2H, Ar–*H*), 7.15-7.09 (*m*, 1H, Ar–*H*), 7.09-7.04 (*m*, 1H, Ar–*H*), 4.29 (q_{AB}, d_A, $^2J_{H,H'}$ = 15.9 Hz, 1H, ArCH_2Ar), 4.15 (q_{AB}, d_B, $^2J_{H,H'}$ = 15.9 Hz, 1H, ArCH_2Ar), 3.54 (*h*, $^3J_{H,H'}$ = 7.2 Hz, 1H, –C$^{(2)}$*H*Me–), 3.38 (*dd*, $^3J_{H,H'}$ = 9.8, 5.4 Hz, 1H, –C$^{(9)}H_2$–), 3.24 (*dd*, $^3J_{H,H'}$ = 9.8, 6.8 Hz, 1H, –C$^{(9)}H_2$–), 2.04 (*ddd*, $^2J_{H,H'}$ = 13.5, $^3J_{H,H'}$ = 7.1, 4.5 Hz, 1H, –C$^{(6)}H_2$–), 1.69-1.57 (*m*, 1H, –C$^{(6)}H_2$–), 1.57-1.49 (*m*, 1H, –C$^{(7)}$*H*Me–), 1.30 (*d*, $^3J_{H,H'}$ = 7.2 Hz, 3H, –C$^{(1)}H_3$), 1.25-1.10 (*m*, 2H, –C$^{(4)}$*H*Me– + –C$^{(3)}H_2$–), 1.03 (*s*, 9H, –OSi(Ph)$_2$C(CH_3)$_3$), 0.84 (*d*, $^3J_{H,H'}$ = 6.2 Hz, 3H, –C$^{(5)}H_3$), 0.74-0.64 (*m*, 1H, –C$^{(3)}$*H*Me–), 0.58 (*d*, $^3J_{H,H'}$ = 6.6 Hz, 3H, –C$^{(8)}H_3$); **[Minor rotamer,** *observable signals***]** 4.46 (*d*, $^3J_{H,H'}$ = 16.1 Hz, 1H, ArCH_2Ar), 4.22 (*d*, $^3J_{H,H'}$ = 16.2 Hz, 1H, ArCH_2Ar), 3.45 (*dd*, $^3J_{H,H'}$ = 10.2, 5.0 Hz, 1H, –C$^{(9)}H_2$–), 3.33 (*dd*, $^3J_{H,H'}$ = 9.8, 7.0 Hz, 1H, –C$^{(9)}H_2$–), 1.48-1.41 (*m*, 2H, aliphatic *H*). **^{13}C{^{1}H} NMR** (101 MHz, $CDCl_3$, 25 °C): δ (ppm) **[Major rotamer]** 141.6 (*C*, arom.), 135.8 (*C*H, arom.), 135.4 (*C*, arom.), 134.33 (*C*, arom.), 134.30 (*C*, arom.), 134.27 (*C*, arom.), 132.3 (*C*, arom.), 130.0 (*C*H, arom.), 129.6 (*C*H, arom.), 129.3 (*C*H, arom.), 128.7 (*C*H, arom.), 128.5 (*C*H, arom.), 127.7 (*C*H, arom.), 126.8 (*C*H, arom.), 126.5 (*C*H, arom.), 126.1 (*C*H, arom.), 124.8 (*C*H, arom.), 124.7 (*C*H, arom.), 69.2 (*C*$^{(9)}$H$_2$), 44.7 (*C*$^{(6)}$H$_2$),

41.5 ($C^{(3)}H_2$), 41.1 (ArCH$_2$Ar), 33.5 ($C^{(2)}$H), 33.3 ($C^{(7)}$H), 29.8 ($C^{(4)}$H), 27.1 (–OSi(Ph)$_2$C(CH$_3$)), 21.41 ($C^{(1)}H_3$), 21.39 ($C^{(5)}H_3$), 19.5 (–OSi(Ph)$_2$$C$(CH$_3$)), 17.6 ($C^{(8)}H_3$). **HRMS** (MALDI): Calcd. for $C_{42}H_{50}OSiNa$ *m/z* 621.3523, found *m/z* 621.3536 $[M+Na]^+$.

1-Cyclohexyl-2-(4-methoxybenzyl)naphthalene (6aab)

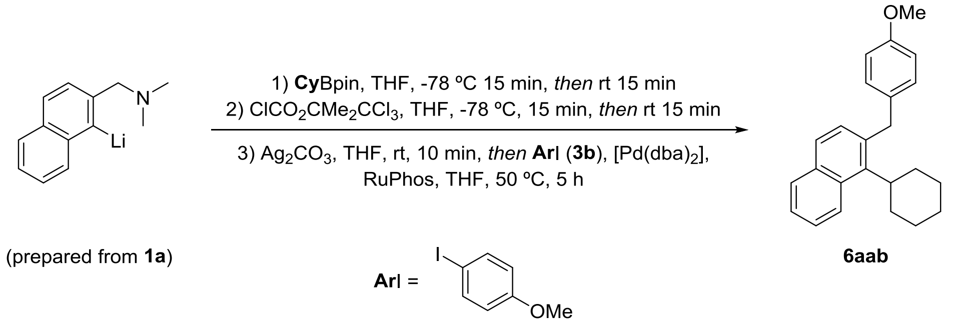

Prepared following general procedure A on a 300 μmol (34 μL) scale. Flash column chromatography on silica gel (hexane:toluene = 91:9) afforded the product as a colorless oil. Yield: 66 mg (66%). ***R*f** (hexane: toluene = 91:9) = 0.31. **IR** (liquid film, cm^{-1}): 2924, 2850, 1611, 1583, 1508, 1448, 1367, 1349, 1300, 1244, 1175, 1116, 1104, 1034, 993, 894, 810, 757, 735, 669, 561, 538, 513, 433. **^{1}H NMR** (400 MHz, CDCl$_3$, 25 ºC): [*Note:* **6aab** exists as a mixture of two rotamers observable by ^{1}H NMR, 85:15] δ (ppm) **[Major rotamer]** 8.46 (*d*, $^3J_{H,H'}$ = 8.3 Hz, 1H, Ar–*H*), 7.83 (*dd*, $^3J_{H,H'}$ = 7.1 Hz, $^4J_{H,H'}$ = 2.2 Hz, 1H, Ar–*H*), 7.68 (*d*, $^3J_{H,H'}$ = 8.3 Hz, 1H, Ar–*H*), 7.49-7.31 (*m*, 3H, Ar–*H*), 7.06 (*d*, $^3J_{H,H'}$ = 8.3 Hz, 2H, Ar–*H*), 6.81 (*d*, $^3J_{H,H'}$ = 8.7 Hz, 2H, Ar–*H*), 4.18 (*s*, 2H, ArC*H$_2$*Ar), 3.78 (*s*, 3H, –OC*H$_3$*), 3.36 (*tt*, $^3J_{H,H'}$ = 12.7, 3.8 Hz, 1H, Cy C*H*), 2.29 (*qd*, $^2J_{H,H'}$ = 12.6 Hz, $^3J_{H,H'}$ = 12.6, 3.4 Hz, 2H, Cy C*H$_2$*), 1.95-1.72 (*br m*, 3H, Cy C*H$_2$*), 1.48-1.18 (*m*, 3H, Cy C*H$_2$*); **[Minor Rotamer**, *observable signals***]** 8.29 (*br d*, $^3J_{H,H'}$ = 8.9 Hz, 1H, Ar–*H*), 7.64-7.58 (*br m*, 1H, Ar–*H*), 7.57-7.50 (*br m*, 1H, Ar–*H*), 7.18-7.10 (*br m*, 1H, Ar–*H*), 4.40 (*br s*, 2H, ArC*H$_2$*Ar), 2.03 (*br d*, $^3J_{H,H'}$ = 12.4 Hz, 2H, Cy C*H$_2$*). **^{13}C{^{1}H} NMR** (101 MHz, CDCl$_3$, 25 ºC): δ (ppm) **[Major rotamer]** 157.9 (*C*, arom.), 140.9 (*C*, arom.), 136.1 (*C*, arom.), 134.2 (*C*, arom.), 133.9 (*C*, arom.), 132.7 (*C*, arom.), 130.1 (*C*H, arom.), 129.5 (*C*H, arom.), 129.2 (*C*H, arom.), 126.8 (*C*H, arom.), 126.64 (*C*H, arom.), 124.63 (*C*H, arom.), 124.58 (*C*H, arom.), 113.9 (*C*H, arom.), 55.4 (–O*C*H$_3$), 41.8 (Cy *C*H), 40.6 (Ar*C*H$_2$Ar), 31.5 (Cy *C*H$_2$), 27.9 (Cy *C*H$_2$), 26.5(Cy *C*H$_2$). **HRMS** (MALDI): Calcd. for $C_{24}H_{26}ONa$ *m/z* 353.1876. found *m/z* 353.1868 $[M+Na]^+$.

Methyl 4-((1-cyclohexylnaphthalen-2-yl)methyl)benzoate (6aac)

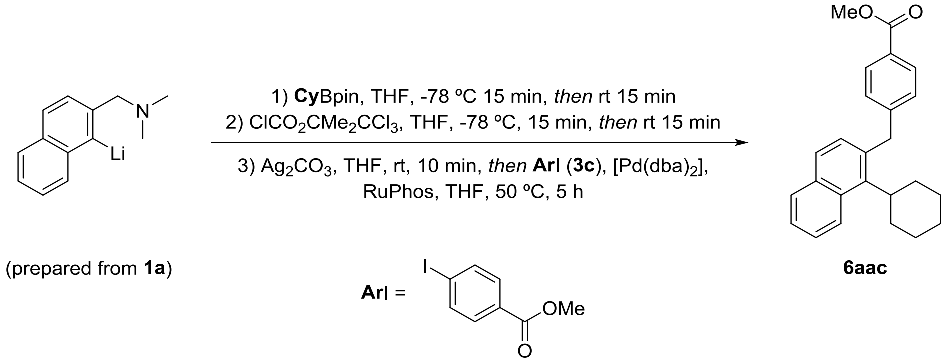

Prepared following general procedure A on a 300 µmol (34 µL) scale. Flash column chromatography on silica gel (petrol ether:ethyl acetate = 98.5:1.5) afforded the product as a pale yellow oil. Yield: 80 mg (74%). ***R*f** (petrol ether:ethyl acetate = 91:9) = 0.32. **IR** (liquid film, cm^{-1}): 2925, 2851, 1719, 1610, 1574, 1508, 1447, 1434, 1414, 1310, 1276, 1177, 1107, 1019, 820, 799, 785, 763, 747, 732, 433. **^{1}H NMR** (400 MHz, $CDCl_3$, 25 ºC): [*Note:* **6aac** exists as a mixture of two rotamers observable by ^{1}H NMR, 85:15] δ (ppm) **[Major rotamer]** 8.50-8.37 (*m*, 1H, Ar–*H*), 7.94 (*d*, $^3J_{H,H'}$ = 8.4 Hz, 2H, Ar–*H*), 7.88-7.78 (*m*, 1H, Ar–*H*), 7.70 (*d*, $^3J_{H,H'}$ = 8.3 Hz, 1H, Ar–*H*), 7.49-7.39 (*m*, 2H, Ar–*H*), 7.36 (*d*, $^3J_{H,H'}$ = 8.3 Hz, 1H, Ar–*H*), 7.21 (*d*, $^3J_{H,H'}$ = 8.0 Hz, 2H, Ar–*H*), 4.28 (*s*, 2H, ArCH_2Ar), 3.90 (*s*, 3H, −OCH_3), 3.24 (*tt*, $^3J_{H,H'}$ = 12.7, 3.6 Hz, 1H, Cy C*H*), 2.27 (*dq*, $^2J_{H,H'}$ = 12.9 Hz, $^3J_{H,H'}$ = 12.9, 3.5 Hz, 2H, Cy CH_2), 1.85-1.70 (*m*, 3H, Cy CH_2), 1.57-1.47 (*m*, 2H, Cy CH_2), 1.44-1.10 (*m*, 3H, Cy CH_2); **[Minor Rotamer**, *observable signals***]** 8.33-8.22 (*m*, 1H, Ar–*H*), 7.66-7.59 (*m*, 1H, Ar–*H*), 7.58-7.50 (*m*, 1H, Ar–*H*), 7.10-7.02 (*m*, 1H, Ar–*H*), 4.50 (*bs*, 2H, ArCH_2Ar), 3.84-3.73 (*m*, 1H, Cy C*H*), 2.02-1.86 (*bm*, 6H, Cy CH_2), 1.67-1.57 (*bm*, 2H, Cy CH_2). **^{13}C{^{1}H} NMR** (101 MHz, $CDCl_3$, 25 ºC): δ (ppm) **[Major rotamer]** 167.2 (*C*, ester), 147.4 (*C*, arom.), 141.2 (*C*, arom.), 134.7 (*C*, arom.), 134.4 (*C*, arom.), 132.7 (*C*, arom.), 130.0 (*C*H, arom.), 129.8 (*C*H, arom.), 129.3 (*C*H, arom.), 128.6 (*C*H, arom.), 128.1 (*C*, arom.), 127.1 (*C*H, arom.), 126.6 (*C*H, arom.), 124.8 (*C*H, arom.), 52.2 (–O*C*H_3), 42.1 (Cy *C*H), 41.7 (Ar*C*H_2Ar), 31.4 (Cy *C*H_2), 27.8 (Cy *C*H_2), 26.4 (Cy *C*H_2). **HRMS** (MALDI): Calcd. for $C_{25}H_{27}O_2$ *m/z* 359.2006. found *m/z* 359.2000 $[M+H]^+$.

2-(4-Chlorobenzyl)-1-cyclohexylnaphthalene (6aad)

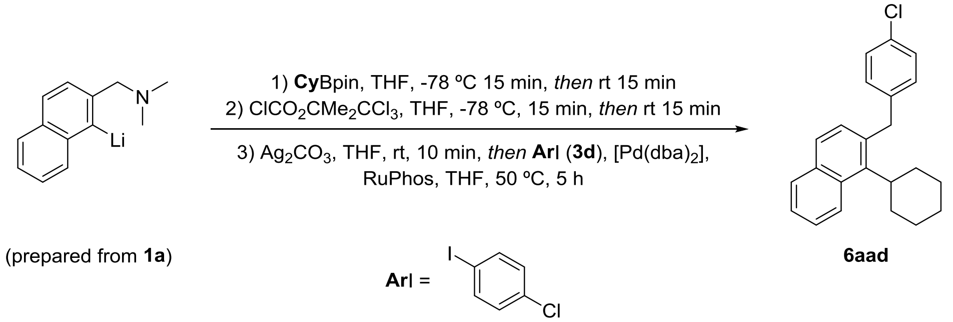

Prepared following general procedure A on a 300 µmol (34 µL) scale. Flash column chromatography on silica gel (hexane) afforded the product as a white solid. Yield: 91 mg (91%). **m.p.** = 69.7-70.9 ºC. ***R*f** (petrol ether:ethyl acetate = 91:9) = 0.54. **IR** (liquid film, cm^{-1}): 3050, 2925, 2851, 1599, 1509, 1489, 1448, 1406, 1177, 1090, 1014, 994, 906, 799, 753, 732, 645, 550, 492, 433, 403. **^{1}H NMR** (400 MHz, $CDCl_3$, 25 ºC [*Note:* **6aad** exists as a mixture of two rotamers observable by ^{1}H NMR, 84:16] *δ* (ppm) **[Major rotamer]** 8.52-8.38 (*m*, 1H, Ar–*H*), 7.90-7.76 (*m*, 1H, Ar–*H*), 7.68 (*d*, $^{3}J_{H,H'}$ = 8.3 Hz, 1H, Ar–*H*), 7.48-7.38 (*m*, 2H, Ar–*H*), 7.33 (*d*, $^{3}J_{H,H'}$ = 8.3 Hz, 1H, Ar–*H*), 7.22 (*d*, $^{3}J_{H,H'}$ = 8.4 Hz, 2H, Ar–*H*), 7.06 (*d*, $^{3}J_{H,H'}$ = 8.2 Hz, 2H, Ar–*H*), 4.19 (*s*, 2H, ArC*H₂*Ar), 3.25 (*tt*, $^{3}J_{H,H'}$ = 12.7, 3.8 Hz, 1H, Cy C*H*), 2.28 (*qd*, $^{2}J_{H,H'}$ = 12.8 Hz, $^{3}J_{H,H'}$ = 11.3, 6.3 Hz, 2H, Cy CH_2), 2.04-1.71 (*m*, 4H, Cy CH_2), 1.62-1.46 (*m*, 2H, Cy CH_2), 1.46-1.15 (*m*, 2H, Cy CH_2); **[Minor Rotamer**, *observable signals***]** 8.28 (*d*, $^{3}J_{H,H'}$ = 8.7 Hz, 1H, Ar–*H*), 7.65-7.58 (*br m*, 1H, Ar–*H*), 7.57-7.49 (*br m*, 1H, Ar–*H*), 4.41 (*br s*, 2H, ArCH_2Ar), 3.85-3.72 (*br m*, 1H, Cy C*H*). **^{13}C{^{1}H} NMR** (101 MHz, $CDCl_3$, 25 ºC): *δ* (ppm) **[Major rotamer]** 141.1 (*C*, arom.), 140.3 (*C*, arom.), 135.0 (*C*, arom.), 134.3 (*C*, arom.), 132.7 (*C*, arom.), 131.8 (*C*, arom.), 130.0 (*C*H, arom.), 129.9 (*C*H, arom.), 129.3 (*C*H, arom.), 128.6 (2*C*H, arom.), 127.0 (*C*H, arom.), 126.6 (*C*H, arom.), 124.8 (*C*H, arom.), 42.0 (Cy *C*H), 40.9 (Ar*C*H_2Ar), 31.5 (Cy *C*H_2), 27.8 (Cy *C*H_2), 26.4 (Cy *C*H_2). **HRMS** (MALDI): Calcd. For $C_{23}H_{23}ClNa$ *m/z* 357.1380. found *m/z* 357.1389 $[M+Na]^+$.

1-Cyclohexyl-2-(4-fluorobenzyl)naphthalene (6aae)

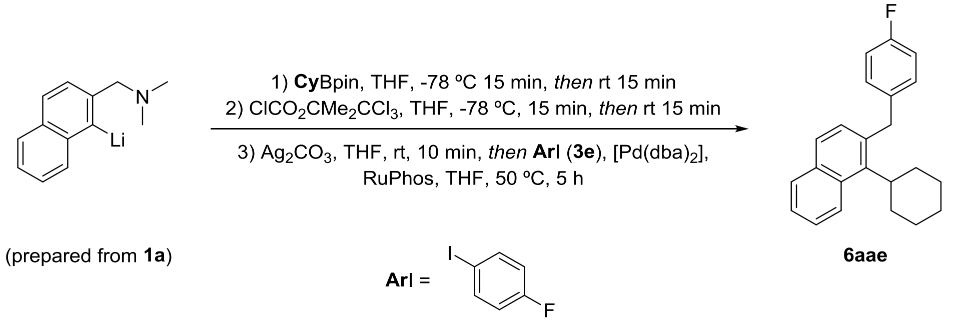

Prepared following general procedure A on a 300 µmol (34 µL) scale. Flash column chromatography on silica gel (hexane:toluene= 97.5:2.5) afforded the product as a white solid. Yield: 88 mg (92%). **m.p.** = 83.4-85.5 ºC. ***R*f** (petrol ether:ethyl acetate = 91:9) = 0.72. **IR** (liquid film, cm^{-1}): 3050, 2940, 2848, 1598, 1506, 1450, 1365, 1219, 1158, 1093, 1018, 997, 962, 908, 893, 814, 753, 739, 670, 656, 501. **^{1}H NMR** (400 MHz, $CDCl_3$, 25 ºC): [*Note:* **6aae** exists as a mixture of two rotamers observable by ^{1}H NMR, 85:15] δ (ppm) **[Major rotamer]** 8.47 (*d*, $^{3}J_{H,H'}$ = 7.9 Hz, 1H, Ar–*H*), 7.92-7.79 (*m*, 1H, Ar–*H*), 7.71 (*d*, $^{3}J_{H,H'}$ = 8.3 Hz, 1H, Ar–*H*), 7.45 (tt, $^{3}J_{H,H'}$ = 6.8, 5.1 Hz, 2H, Ar–*H*), 7.37 (*d*, $^{3}J_{H,H'}$ = 8.3 Hz, 1H, Ar–*H*), 7.16-7.04 (*m*, 2H, Ar–*H*), 6.96 (t, $^{3}J_{H,H'}$ = 8.7 Hz, 2H, Ar–*H*), 4.22 (*s*, 2H, ArC*H*$_2$Ar), 3.30 (*ddt*, $^{3}J_{H,H'}$ = 12.6, 7.4, 3.7 Hz, 1H, Cy C*H*), 2.30 (*qd*, $^{2}J_{H,H'}$ = 12.7 Hz, $^{3}J_{H,H'}$ = 12.7, 3.6 Hz, 2H, Cy C*H*$_2$), 2.10-1.72 (*m*, 4H, Cy C*H*$_2$), 1.64-1.50 (*m*, 2H, Cy C*H*$_2$), 1.47-1.17 (*m*, 2H, Cy C*H*$_2$); **[Minor Rotamer**, *observable signals***]** 8.31 (*br d*, $^{3}J_{H,H'}$ = 8.8 Hz, 1H, Ar–*H*), 7.67-7.60 (*m*, 1H), 7.59-7.52 (*m*, 1H), 4.44 (*bs*, 0.4H, ArC*H*$_2$Ar), 3.82 (*bt*, $^{3}J_{H,H'}$ = 11.6 Hz, 0.2H, Cy C*H*). **^{13}C{^{1}H} NMR** (101 MHz, $CDCl_3$, 25 ºC): δ (ppm) **[Major rotamer]** 161.4 (*d*, $^{1}J_{C,F}$ = 243.8 Hz, *C*, arom.), 141.0 (*C*, arom.), 137.52 (*d*, $^{4}J_{C,F}$ = 3.2 Hz, *C*, arom.), 137.45 (*C*, arom.), 135.5 (*C*, arom.), 134.3 (*C*, arom.), 130.0 (*C*H, arom.), 129.9 (*d*, $^{3}J_{C,F}$ = 7.7 Hz, *C*H, arom.), 129.2 (*C*H, arom.), 127.0 (*C*H, arom.), 126.7 (*C*H, arom.), 124.77 (*C*H, arom.), 124.74 (*C*H, arom.), 115.2 (*d*, $^{2}J_{C,F}$ = 21.2 Hz, *C*H, arom.), 42.0 (Cy *C*H), 40.8 (Ar*C*H$_2$Ar), 31.4 (Cy *C*H$_2$), 27.8 (Cy *C*H$_2$), 26.4 (Cy *C*H$_2$). **^{19}F{^{1}H}-NMR** (377 MHz, $CDCl_3$, 25 ºC): δ (ppm) –117.5 (*s*, 1F); **[Minor Rotamer**, *observable signals***]** –117.5 (*bs*, 1F, Ar–*F*). **HRMS** (MALDI): Calcd. For $C_{23}H_{23}F$ *m/z* 318.1778. found *m/z* 318.1787 $[M]^{+}$.

1-Cyclohexyl-2-(3-(trifluoromethyl)benzyl)naphthalene (6aaf)

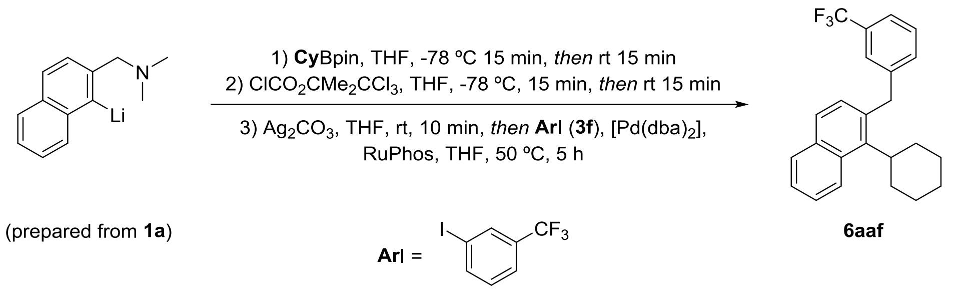

Prepared following general procedure A on a 300 µmol (34 µL) scale. Flash column chromatography on silica gel (gradient, toluene:hexane = 0:100 to 5: 95) afforded the product as a white solid. Yield: 79 mg (71%). **m.p.** = 83.4-85.5 ºC. ***R*f** (toluene:hexane = 5:95) = 0.49. **IR** (liquid film, cm^{-1}): 2927, 2853, 1446, 1328, 1161, 1120, 1074, 909, 734, 701. **^{1}H NMR** (400 MHz, $CDCl_3$, 25 ºC): [*Note*: purified product contains a minor inseparable unidentified impurity as determined by ^{1}H NMR, e.g. 4.84 ppm (*br s*). Product **6aaf** exists as a mixture of two rotamers observable by ^{1}H NMR, 87:13] δ (ppm) **[Major Rotamer]** 8.49 (*d*, $^{3}J_{H,H'}$ = 8.1 Hz, 1H, Ar–*H*), 7.87 (*d*, $^{3}J_{H,H'}$ = 7.6 Hz, 1H, Ar–*H*), 7.73 (*d*, $^{3}J_{H,H'}$ = 8.3 Hz, 1H, Ar–*H*), 7.53-7.43 (*m*, 4H, Ar–*H*), 7.41-7.35 (*m*, 2H, Ar–*H*), 7.35-7.31 (*m*, 1H, Ar–*H*), 4.30 (*br s*, 2H, ArC*H*2Ar), 3.28 (*tt*, $^{3}J_{H,H'}$ = 12.7, 3.8 Hz, 1H, Cy C*H*), 2.32 (*qd*, $^{2}J_{H,H'}$ = 12.8 Hz, $^{3}J_{H,H'}$ = 12.8, 3.6 Hz, 2H, Cy C*H2*), 2.04-1.76 (*m*, 4H, Cy C*H2*), 1.62-1.50 (*m*, 2H, Cy C*H2*), 1.47-1.35 (*m*, 1H, Cy C*H2*), 1.31-1.19 (*m*, 2H, Cy C*H2*); **[Minor Rotamer**, *observable signals***]** 8.33 (*br d*, $^{3}J_{H,H'}$ = 8.6 Hz, 1H, Ar–*H*), 7.09 (*br d*, $^{3}J_{H,H'}$ = 7.6 Hz, 1H, Ar–*H*), 4.54 (*br s*, 2H, ArC*H2*Ar), 3.89-3.79 (*m*, 1H, Cy C*H*). **^{13}C{^{1}H} NMR** (101 MHz, $CDCl_3$, 25 ºC): δ (ppm) **[Major Rotamer]** 142.8 (*C*, arom.), 141.0 (*C*, arom.), 134.5 (*C*, arom.), 134.4 (*C*, arom.), 132.7 (*C*, arom.), 132.0 (*C*H, arom.), 130.8 (*q*, $^{2}J_{C,F}$ = 32.0 Hz, *C*, arom.), 129.9 (*C*H, arom.), 129.3 (*C*H, arom.), 128.9 (*C*H, arom.), 127.2 (*C*H, arom.), 126.6 (*C*H, arom.), 125.2 (*q*, $^{3}J_{C,F}$ = 3.8 Hz, *C*H, arom.), 124.9 (*C*H, arom.), 124.8 (*C*H, arom.), 124.3 (*q*, $^{1}J_{C,F}$ = 272.8 Hz, *C*F3), 123.0 (*q*, $^{3}J_{C,F}$ = 3.9 Hz, *C*H, arom.), 42.2 (Cy *C*H), 41.4 (Ar*C*H2Ar), 31.4 (Cy *C*H2), 27.7 (Cy *C*H2), 26.4 (Cy *C*H2); *signals corresponding to the minor rotamer are clearly evident as broad signals on the baseline*. **^{19}F{^{1}H}-NMR** (377 MHz, $CDCl_3$, 25 ºC): δ (ppm) **[Major Rotamer]** – 62.58 (*s*, 3F); **[Minor Rotamer]** – 62.49 (*s*, 3F). **HRMS** (MALDI): Calcd. for $C_{24}H_{23}F_3Na$ *m/z* 391.1644, found *m/z* 391.1655 $[M+Na]^+$.

1-Cyclohexyl-2-(2-methylbenzyl)naphthalene (6aag)

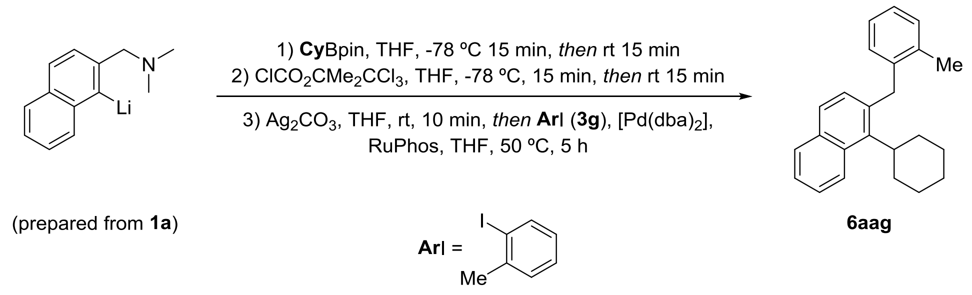

Prepared following general procedure A on a 300 µmol (34 µL) scale. Flash column chromatography on silica gel (hexane) afforded the product as a colorless oil. Yield: 78 mg (83%). ***R*f** (petrol ether:ethyl acetate = 91:9) = 0.77. **IR** (liquid film, cm^{-1}): 3049, 3016, 2924, 2850, 1600, 1509, 1490, 1448, 1378, 1364, 1175, 1051, 1034, 994, 907, 830, 817, 766, 733, 674, 572, 433. **^{1}H NMR** (400 MHz, $CDCl_3$, 25 ºC): [*Note:* **6aag** exists as a mixture of two rotamers observable by ^{1}H NMR, 84:16] δ (ppm) **[Major rotamer]** 8.48 (*d*, $^{3}J_{H,H'}$ = 8.3 Hz, 1H, Ar–*H*), 7.85 (*dd*, $^{3}J_{H,H'}$ = 7.3 Hz, $^{4}J_{H,H'}$ = 2.1 Hz, 1H, Ar–*H*), 7.67 (*dd*, $^{3}J_{H,H'}$ = 8.3 Hz, 1H, Ar–*H*), 7.50-7.38 (*m*, 2H, Ar–*H*), 7.32-7.19 (*m*, 2H, Ar–*H*), 7.14 (*t*, $^{3}J_{H,H'}$ = 7.3 Hz, 1H, Ar–*H*), 7.05 (*t*, $^{3}J_{H,H'}$ = 7.6 Hz, 1H, Ar–*H*), 6.80 (*d*, $^{3}J_{H,H'}$ = 7.7 Hz, 1H, Ar–*H*), 4.17 (s, 2H, ArC*H*2Ar), 3.16 (*tt*, $^{3}J_{H,H'}$ = 12.3, 3.5 Hz, 1H, Cy C*H*), 2.48-2.23 (*m*, 3H for Ar–C*H*3 plus 2H for Cy C*H*2), 1.94-1.72 (*m*, 4H, Cy C*H*2), 1.64 (*d*, $^{3}J_{H,H'}$ = 13.2 Hz, 2H, Cy C*H*2), 1.46-1.12 (*m*, 2H, Cy C*H*2) ; **[Minor Rotamer**, *observable signals***]** 8.29 (*bs*, 1H, Ar–*H*), 7.59-7.51 (*m*, 1H, Ar–*H*), 4.37 (*bs*, 2H, ArC*H*2Ar), 3.80 (*bs*, 1H, Cy C*H*), 2.08-1.95 (*m*, 2H, Cy C*H*2). **^{13}C{^{1}H} NMR** (101 MHz, $CDCl_3$, 25 ºC): δ (ppm) **[Major rotamer]** 141.0 (*C*, arom.), 139.8 (*C*, arom.), 135.9 (*C*, arom.), 135.4 (*C*, arom.), 134.2 (*C*, arom.), 132.6 (*C*, arom.), 130.1 (*C*H, arom.), 129.8 (*C*H, arom.), 129.2 (*C*H, arom.), 129.0 (*C*H, arom.), 126.9 (*C*H, arom.), 126.6 (*C*H, arom.), 126.2 (*C*H, arom.), 126.1 (*C*H, arom.), 124.7 (*C*H, arom.), 124.6 (*C*H, arom.), 42.0 (Ar*C*H), 38.5 (Ar*C*H2Ar), 31.5 (Cy *C*H2), 27.9 (Cy *C*H2), 26.5 (Cy *C*H2), 20.2 (*C*H3). **HRMS** (MALDI): Calcd. For $C_{24}H_{26}$ *m/z* 314.2029. found *m/z* 314.2035 $[M]^{+}$.

4-((1-Cyclohexylnaphthalen-2-yl)methyl)pyridine (6aah)

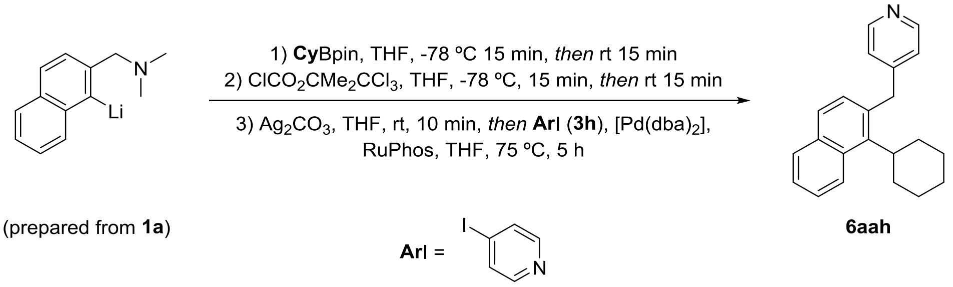

Prepared following general procedure A, at 75 ºC for the coupling step, on a 300 μmol (34 μL) scale. Flash column chromatography on silica gel (hexane) afforded the product as a pale orange oil. Yield: 32 mg (36%). ***R*f** (petrol ether:ethyl acetate = 67:33) = 0.15. **IR** (liquid film, cm^{-1}): 3050, 2924, 2850, 1597, 1558, 1509, 1494, 1448, 1414, 1371, 1349, 1218, 1179, 1116, 1069, 1034, 993, 908, 894, 864, 836, 807, 790, 756, 732, 679, 597, 566, 541, 501, 482, 433. **^{1}H NMR** (400 MHz, $CDCl_3$, 25 ºC): [*Note:* **6aah** exists as a mixture of two rotamers observable by ^{1}H NMR, 83:17] *δ* (ppm) **[Major rotamer]** 8.54-8.37 (*m*, 3H, Ar–*H*), 7.89-7.78 (*m*, 1H, Ar–*H*), 7.70 (*d*, $^3J_{H,H'}$ = 8.3 Hz, 1H, Ar–*H*), 7.50-7.38 (*m*, 2H, Ar–*H*), 7.33 (*d*, $^3J_{H,H'}$ = 8.3 Hz, 1H, Ar–*H*), 7.12-6.96 (*m*, 2H, Ar–*H*), 4.21 (*s*, 2H, ArC*H₂*Ar), 3.15 (*ddt*, $^3J_{H,H'}$ = 16.3, 12.6, 3.8 Hz, 1H, Cy C*H*), 2.27 (*dq*, $^2J_{H,H'}$ = 12.7 Hz, $^3J_{H,H'}$ = 12.7, 3.6 Hz, 2H, Cy CH_2), 2.07-1.69 (*m*, 3H, Cy CH_2), 1.52 (*m*, 2H, Cy CH_2), 1.46-1.07 (*m*, 3H, Cy CH_2); **[Minor Rotamer**, *observable signals***]** 8.34-8.21 (*br m*, 1H, Ar–*H*), 7.67-7.60 (*br m*, 1H, Ar–*H*), 7.59-7.51 (*br m*, 1H, Ar–*H*), 4.43 (*br s*, 2H, ArCH_2Ar), 3.86-3.70 (*br m*, 1H, Cy C*H*). **^{13}C{^{1}H} NMR** (101 MHz, $CDCl_3$, 25 ºC): *δ* (ppm) **[Major rotamer]** 150.9 (*C*, arom.), 149.9 (*C*H, arom.), 141.3 (*C*, arom.), 134.5 (*C*, arom.), 133.5 (*C*, arom.), 132.6 (*C*, arom.), 129.9 (*C*H, arom.), 129.3 (*C*H, arom.), 127.2 (*C*H, arom.), 126.6 (*C*H, arom.), 125.02 (*C*H, arom.), 124.97 (*C*H, arom.), 123.9 (*C*H, arom.), 42.3 (Cy *C*H), 41.0 (Ar*C*H₂Ar), 31.4 (Cy CH_2), 27.8 (Cy CH_2), 26.3 (Cy CH_2). **HRMS** (MALDI): Calcd. For $C_{22}H_{24}N$ *m/z* 302.1903. found *m/z* 302.1910 $[M+H]^+$.

1-Benzyl-2-cyclohexylbenzene (6baa)

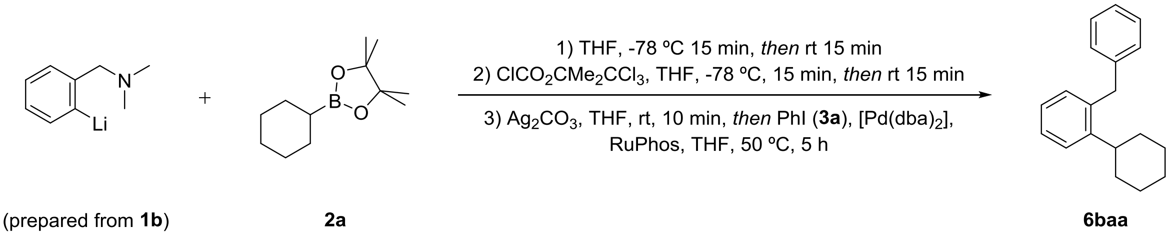

Prepared following general procedure A on a 300 μmol (34 μL) scale. Flash column chromatography on silica gel (hexane) afforded the product as a colorless oil. Yield: 51 mg (67%). ***R*f** (hexane) = 0.26. **IR** (liquid film, cm^{-1}): 3061, 3025, 2923, 2850, 1601, 1494, 1448, 1074, 1050, 1030, 998, 752, 728, 696. **^{1}H NMR** (400 MHz, $CDCl_3$, 25 ºC): δ (ppm) 7.33-7.09 (*m*, 9H, Ar–*H*), 4.07 (*s*, 2H, ArCH_2Ar), 2.76 (*tt*, $^{3}J_{H,H'}$ = 11.7, 3.3 Hz, 1H, Cy C*H*), 1.86-1.68 (*m*, 3H, Cy CH_2), 1.67-1.58 (*m*, 2H, Cy CH_2), 1.48-1.17 (*m*, 5H, Cy CH_2). **^{13}C{^{1}H} NMR** (101 MHz, $CDCl_3$, 25 ºC): δ (ppm) 146.4 (*C*, arom.), 141.6 (*C*, arom.), 137.8 (*C*, arom.), 130.7 (*C*H, arom.), 128.9 (*C*H, arom.), 128.5 (*C*H, arom.), 126.9 (*C*H, arom.), 126.4 (*C*H, arom.), 126.0 (*C*H, arom.), 125.8 (*C*H, arom.), 40.1 (Cy *C*H), 39.2 (Ar*C*H$_2$Ar), 34.3 (Cy *C*H$_2$), 27.3 (Cy *C*H$_2$), 26.4 (Cy *C*H$_2$). **HRMS** (MALDI): Calcd. For $C_{19}H_{22}Na$ *m/z* 273.1614. found *m/z* 273.16.18 $[M+Na]^+$.

1-Benzyl-2-cyclohexyl-4-methylbenzene (6caa)

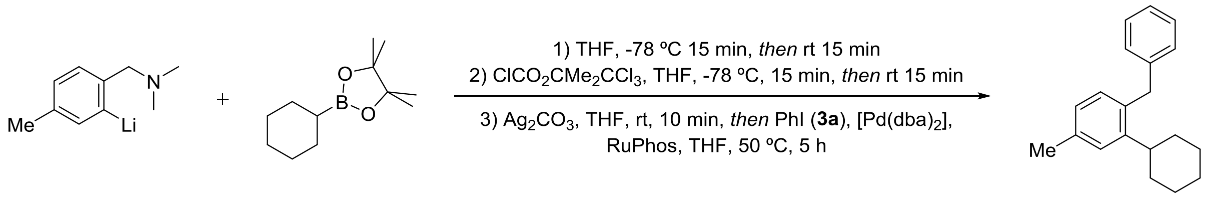

(prepared from **1c**) **2a** **6caa**

Prepared following general procedure A on a 300 μmol (34 μL) scale. Flash column chromatography on silica gel (hexane) afforded the product as a white solid. Yield: 51 mg (64%). **m.p.** = 46.1-47.8 ºC. ***R*f** (petrol ether:ethyl acetate = 91:9) = 0.71. **IR** (liquid film, cm^{-1}): 3055, 3029, 3001, 2941, 2921, 2845, 1598, 1582, 1503, 1492, 1450, 1226, 1073, 1030, 949, 873, 853, 836, 794, 730, 717, 695, 632, 600, 575, 517, 502, 467, 450. **^{1}H NMR** (400 MHz, $CDCl_3$, 25 ºC): δ (ppm) 7.30-7.22 (*m*, 2H, Ar–*H*), 7.21-7.11 (*m*, 2H, Ar–*H*), 7.10-7.07 (*br m*, 1H, Ar–*H*), 7.02 (*d*, $^{3}J_{H,H'}$ = 7.7 Hz, 1H, Ar–*H*), 6.95 (*ddd*, $^{3}J_{H,H'}$ = 7.7 Hz, $^{4}J_{H,H'}$ = 1.9 Hz, $^{5}J_{H,H'}$ = 0.7 Hz, 1H, Ar–*H*), 4.02 (*s*, 2H, ArCH_2Ar), 2.72 (*tt*, $^{3}J_{H,H'}$ = 11.7, 3.3 Hz, 1H, Cy C*H*), 2.34 (*s*, 3H, Ar–CH_3), 1.84-1.66 (*m*, 3H, Cy CH_2), 1.66-1.56 (*m*, 2H, Cy CH_2), 1.48-1.15 (*m*, 5H, Cy CH_2). **^{13}C{^{1}H} NMR** (101 MHz, $CDCl_3$, 25 ºC): δ (ppm) 146.2 (*C*, arom.), 141.8 (*C*, arom.), 136.2 (*C*, arom.), 134.8 (*C* arom.), 130.6 (*C*H, arom.), 128.8 (*C*H, arom.), 128.5 (*C*H, arom.), 127.2 (*C*H, arom.), 126.5 (*C*H, arom.), 125.9 (*C*H, arom.), 40.0 (Cy *C*H), 38.9 (Ar*C*H$_2$Ar), 34.3 (Cy *C*H$_2$), 27.3 (Cy *C*H$_2$), 26.5 (Cy *C*H$_2$), 21.5 (Ar*C*H$_3$). **HRMS** (MALDI): Calcd. for $C_{20}H_{24}Na$ *m/z* 287.1770 found *m/z* 287.1778 $[M+Na]^+$.

1-Cyclohexyl-2-(3-methoxybenzyl)-3-methylbenzene (6dai)

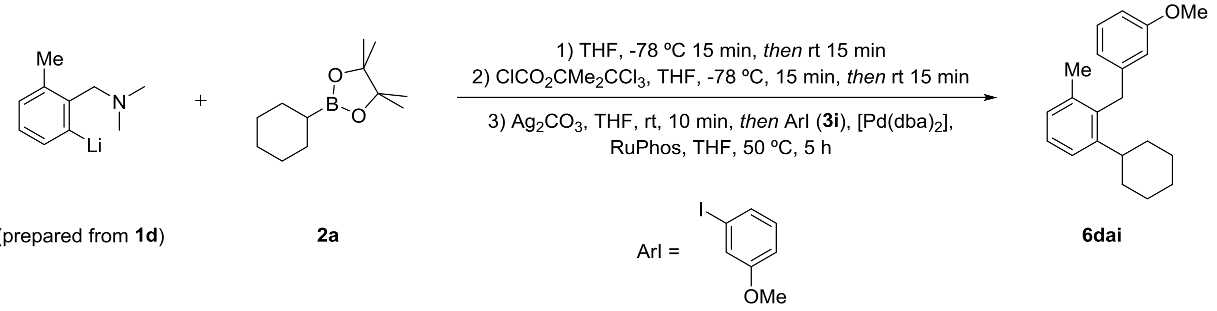

(prepared from **1d**) **2a** **6dai**

Prepared following general procedure A on a 300 µmol (34 µL) scale. Yield: 49% (by ^{1}H NMR using dibromomethane as internal standard). Purification of the product was not achieved by flash column chromatography (silica, hexane:diethyl ether = 99:1, $\boldsymbol{R_f}$ (hexane:diethyl ether = 99:1) = 0.41) because it co-elutes with unidentified impurities. 7 mg of pure material were obtained as a colourless oil using preparative HPLC (Kromasil 60-5SIL, hexane, flow rate 18.9 mL/min, λ = 214 nm, retention time t_R = 4.2 min). **IR** (liquid film, cm^{-1}): 2924, 2850, 1746, 1608, 1600, 1583, 1488, 1464, 1448, 1379, 1313, 1270, 1258, 1228, 1155, 1142, 1092, 1051, 960, 873, 775, 764, 734, 713, 691, 638, 444. **^{1}H NMR** (400 MHz, $CDCl_3$, 25 ºC): δ (ppm) 7.19-7.11 (*m*, 3H, Ar–*H*), 7.07-6.97 (*m*, 1H, Ar–*H*), 6.70 (*ddd*, $^3J_{H,H'}$ = 8.2 Hz, $^4J_{H,H'}$ = 2.7 Hz, $^5J_{H,H'}$ = 0.9 Hz, 1H, Ar–*H*), 6.61 (*ddd*, $^3J_{H,H'}$ = 7.6 Hz, $^4J_{H,H'}$ = 1.6 Hz, $^5J_{H,H'}$ = 0.9 Hz, 1H, Ar–*H*), 6.59-6.55 (*m*, 1H, Ar–*H*), 4.07 (*s*, 2H, ArC*H$_2$*Ar), 3.74 (*s*, 3H, Ar–OC*H$_3$*), 2.70 (*tt*, $^3J_{H,H'}$ = 11.8, 3.2 Hz, 1H, Cy C*H*), 2.23 (*s*, 3H, Ar–C*H$_3$*), 1.83-1.60 (*m*, 5H, Cy C*H$_2$*), 1.48-1.15 (*m*, 5H, Cy C*H$_2$*). **^{13}C{^{1}H} NMR** (101 MHz, $CDCl_3$, 25 ºC): δ (ppm) 159.9 (*C*, arom.), 146.9 (*C*, arom.), 142.6 (*C*, arom.), 137.4 (*C* arom.), 135.5 (*C* arom.), 129.4 (*C*H, arom.), 128.0 (*C*H, arom.), 126.7 (*C*H, arom.), 124.1 (*C*H, arom.), 120.7 (*C*H, arom.), 114.1 (*C*H, arom.), 111.0 (*C*H, arom.), 55.3 (Ar–O*C*H$_3$), 40.5 (Cy *C*H), 34.7 (Cy *C*H$_2$), 34.4 (Ar*C*H$_2$Ar), 27.3 (Cy *C*H$_2$), 26.4 (Cy *C*H$_2$), 20.9 (Ar–*C*H$_3$). **HRMS** (MALDI): Calcd. for $C_{21}H_{26}ONa$ *m/z* 317.1876 found *m/z* 317.1870 $[M+Na]^+$.

2-Cyclohexyl-4-methoxy-1-(3-methoxybenzyl)benzene (6eai)

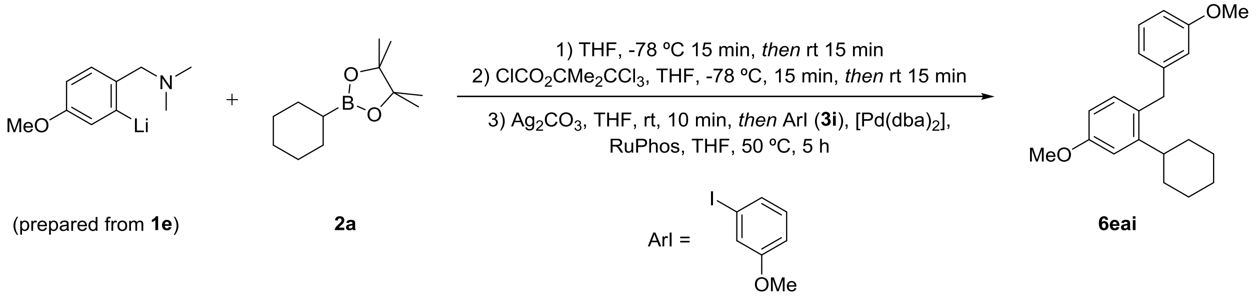

(prepared from **1e**) **2a** **6eai**

Prepared following general procedure A on a 300 μmol (34 μL) scale. Yield: 65% (by ^{1}H NMR using dibromomethane as internal standard). Purification of the product was not achieved by flash column chromatography (silica, hexane:toluene:diethyl ether = 92:5:3, $\boldsymbol{R_f}$ (hexane:toluene:diethyl ether = 92:5:3) = 0.18) because it co-elutes with unidentified impurities. 10 mg of pure material were obtained as a colourless oil using preparative HPLC (Kromasil 60-5SIL, hexane, flow rate 10.0 mL/min, λ = 214 nm, retention time t_R = 17 min). **IR** (liquid film, cm^{-1}): 2997, 2923, 2849, 1599, 1582, 1499, 1488, 1464, 1449, 1294, 1252, 1204, 1158, 1145, 1091, 1043, 997, 945, 872, 849, 808, 784, 766, 733, 718, 692, 593, 577, 465, 442. **^{1}H NMR** (400 MHz, $CDCl_3$, 25 ºC): δ (ppm) 7.21-7.14 (*m*, 2H, Ar–*H*), 6.77 (*dd*, $^3J_{H,H'}$ = 8.6 Hz, $^4J_{H,H'}$ = 2.8 Hz, 1H, Ar–*H*), 6.75-6.70 (*m*, 2H, Ar–*H*), 6.70-6.64 (*m*, 2H, Ar–*H*), 3.98 (*s*, 2H, ArCH_2Ar), 3.76 (*s*, 3H, Ar–OCH_3), 3.75 (*s*, 3H, Ar–OCH_3), 2.67 (*tt*, $^3J_{H,H'}$ = 11.5, 3.2 Hz, 1H, Cy C*H*), 1.83-1.66 (*m*, 3H, Cy CH_2), 1.66-1.58 (*m*, 2H, Cy CH_2), 1.42-1.17 (*m*, 5H, Cy CH_2). **^{13}C{^{1}H} NMR** (101 MHz, $CDCl_3$, 25 ºC): δ (ppm) 159.8 (*C*, arom.), 157.5 (*C*, arom.), 142.9 (*C*, arom.), 138.9 (*C* arom.), 138.8 (*C* arom.), 129.5 (*C*H, arom.), 127.3 (*C*H, arom.), 121.4 (*C*H, arom.), 116.3 (*C*H, arom.), 114.7 (*C*H, arom.), 111.9 (*C*H, arom.), 111.4 (*C*H, arom.), 55.3 (2 –O*C*H_3), 39.4 (Cy *C*H), 39.3 (Ar*C*H_2Ar), 34.6 (Cy *C*H_2), 27.4 (Cy *C*H_2), 26.4 (Cy *C*H_2). **HRMS** (MALDI): Calcd. for $C_{21}H_{26}O_2Na$ *m/z* 333.1825 found *m/z* 333.1836 $[M+Na]^+$.

1-Cyclohexyl-4-fluoro-2-(3-methoxybenzyl)benzene (6fai)

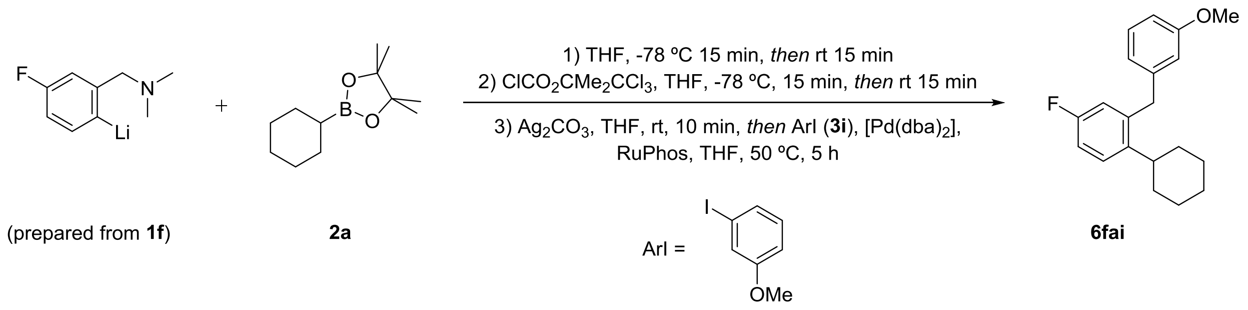

Prepared following general procedure A on a 300 μmol (34 μL) scale. Yield: 65% (by ^{1}H NMR using dibromomethane as internal standard). Flash column chromatography on silica gel (gradient, hexane:toluene = 95: 5 to 83:17), followed by preparative TLC (twice in hexane:toluene = 95:5) afforded 9 mg of the product as a colorless oil, which co-eluted with a minor inseparable unidentified impurity. $\boldsymbol{R_f}$ (petrol ether:toluene = 95:5) = 0.26. **IR** (liquid film, cm^{-1}): 2924, 2850, 1748, 1599, 1583, 1489, 1449, 1437, 1417, 1380, 1372, 1346, 1314, 1191, 1257, 1248, 1227, 1191, 1146, 1098, 1050, 998, 962, 872, 860, 815, 785, 766, 734, 716, 692, 658, 573, 558, 536, 476, 466, 440. **^{1}H NMR** (400 MHz, $CDCl_3$, 25 ºC): [*Note*: purified product contains a minor inseparable unidentified impurity as determined by ^{1}H NMR, e.g. 7.75 ppm (*dd*, $^3J_{H,H'}$ = 8.3, 6.8 Hz), 2.75 (*d*, $^3J_{H,H'}$ = 7.0 Hz), 1.34 (*s*).] δ (ppm) 7.23-7.15 (*m*, 2H, Ar–*H*), 6.89 (*td*, $^3J_{H,H'}$ =

8.4 Hz, $^4J_{H,H'}$ = 2.8 Hz, 1H, Ar–*H*), 6.78 (*dd*, $^3J_{H,H'}$ = 10.0 Hz, $^4J_{H,H'}$ = 2.8 Hz, 1H, Ar–*H*), 6.76-6.69 (*m*, 2H, Ar–*H*), 6.68-6.63 (*m*, 1H, Ar–*H*), 3.98 (*s*, 2H, ArC*H*$_2$Ar), 3.77 (*s*, 3H, Ar–OC*H*$_3$), 2.70 (*tt*, $^3J_{H,H'}$ = 11.5, 3.3 Hz, 1H, Cy C*H*), 1.83-1.58 (m, 6H, Cy C*H*$_2$), 1.41-1.20 (m, 4H, Cy C*H*$_2$). **^{13}C{^{1}H} NMR** (101 MHz, CDCl$_3$, 25 ºC): [*Note*: purified product contains a minor inseparable unidentified impurity as determined by ^{13}C NMR, e.g. 33.1, 29.7, 26.8, 26.6, 25.0 ppm.] δ (ppm) 160.9 (*d*, $^1J_{C,F}$ = 243.2 Hz, *C*, arom.), 159.9 (*C*, arom.), 142.3 (*C*, arom.), 142.0 (*d*, $^4J_{C,F}$ = 3.1 Hz, *C*, arom.), 139.8 (*d*, $^3J_{C,F}$ = 6.7 Hz, *C*, arom.), 129.6 (*C*H, arom.), 127.7 (*d*, $^3J_{C,F}$ = 7.9 Hz, *C*H, arom.), 121.4 (*C*H, arom.), 116.9 (*d*, $^2J_{C,F}$ = 20.9 Hz, *C*H, arom.), 114.8 (*C*H, arom.), 113.5 (*d*, $^2J_{C,F}$ = 20.6 Hz, *C*H, arom.), 111.5 (*C*H, arom.), 55.34 (Ar–O*C*H$_3$), 39.5 (Cy *C*H), 39.0 (*d*, $^4J_{C,F}$ = 1.5 Hz, Ar*C*H$_2$Ar), 34.5 (Cy *C*H$_2$), 27.3 (Cy *C*H$_2$), 26.4 (Cy *C*H$_2$). **^{19}F{^{1}H}-NMR** (377 MHz, CDCl$_3$, 25 ºC): [*Note*: purified product contains a minor inseparable unidentified impurity as determined by ^{19}F NMR, e.g. –110.61 ppm (*s*).] δ (ppm) – 118.15 (*s*, 1F). **HRMS** (APCI): Calcd. For $C_{20}H_{24}OF$ *m/z* 299.1806, found *m/z* 299.1808 [M+H]$^+$.

2-Cyclohexyl-1-(3-methoxybenzyl)-4-(trifluoromethyl)benzene (6gai)

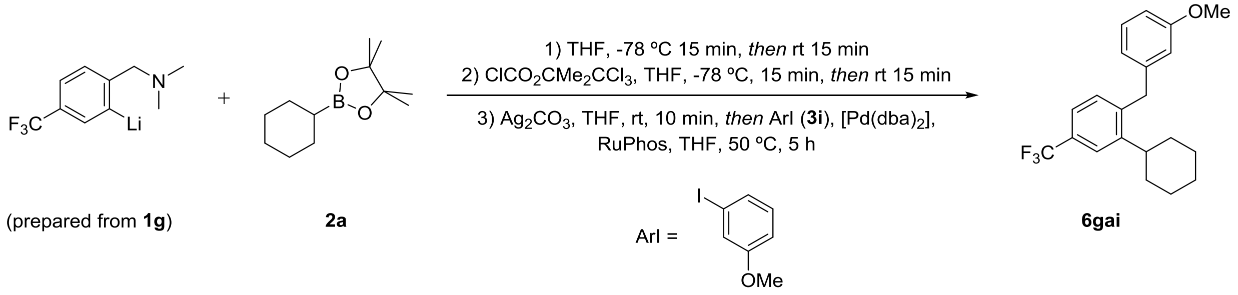

Prepared following general procedure A on a 300 µmol (34 µL) scale. Flash column chromatography on silica gel (gradient, toluene:hexane = 10:90 to 20:80) afforded the product as a colorless oil. Yield: 51 mg (49%). ***R*$_f$** (toluene:hexane = 30:70) = 0.60. **IR** (liquid film, cm^{-1}): 2927, 2852, 1599, 1585, 1489, 1450. 1328, 1274, 1157, 1117, 1098, 1049, 771, 693. **^{1}H NMR** (400 MHz, CDCl$_3$, 25 ºC): δ (ppm) 7.50 (*d*, $^3J_{H,H'}$ = 1.3 Hz, 1H, Ar–*H*), 7.37 (*dd*, $^3J_{H,H'}$ = 8.1 Hz, $^4J_{H,H'}$ = 1.3 Hz, 1H, Ar–*H*), 7.21 (app. *t*, $^3J_{H,H'}$ = 7.6 Hz, 2H, Ar–*H*), 6.76 (*dd*, $^3J_{H,H'}$ = 8.2 Hz, $^4J_{H,H'}$ = 2.5 Hz, 1H, Ar–*H*), 6.71 (*d*, $^3J_{H,H'}$ = 7.5 Hz, 1H, Ar–*H*), 6.66 (app. *t*, $^3J_{H,H'}$ = 2.0 Hz, 1H, Ar–*H*), 4.06 (*s*, 2H, C*H*$_2$), 3.77 (*s*, 3H, OC*H*$_3$), 2.81 (*tt*, $^3J_{H,H'}$ = 11.7, 3.2 Hz, C*H*), 1.84-1.71 (*m*, 3H, C*H*$_2$), 1.69-1.62 (*m*, 2H, C*H*$_2$), 1.47-1.22 (*m*, 5H, C*H*$_2$). **^{13}C{^{1}H} NMR** (101 MHz, CDCl$_3$, 25 ºC): δ (ppm) 159.9 (*C*, arom.), 147.1 (*C*, arom.), 142.0 (*C*, arom.), 141.6 (*C*, arom.), 130.9 (*C*H, arom.), 129.6 (*C*H, arom.), 129.1 (*q*, $^2J_{C,F}$ = 31.8 Hz, CF$_3$–*C*, arom.), 124.7 (*q*, $^1J_{C,F}$ = 269.2 Hz, *C*F$_3$), 123.2 (*q*, $^3J_{C,F}$ = 4.5 Hz, *C*H, arom.), 122.6 (*q*, $^3J_{C,F}$ = 3.8 Hz, *C*H, arom.), 121.3 (*C*H, arom.), 114.8 (*C*H, arom.), 111.5

(*C*H, arom.), 55.3 (O*C*H$_3$), 40.0 (Cy *C*H), 39.0 (Ar*C*H$_2$Ar), 34.1 (Cy *C*H$_2$), 27.1 (Cy *C*H$_2$), 26.2 (Cy *C*H$_2$). **^{19}F{^{1}H}-NMR** (377 MHz, CDCl$_3$, 25 ºC): δ (ppm) –62.28 (*s*, 3F). **HRMS** (MALDI): Calcd. For $C_{21}H_{23}OF_3Na$ *m/z* 371.1593 found *m/z* 371.1581 [M+Na]$^+$.

2-Cyclohexyl-3-(3-methoxybenzyl)benzo[b]thiophene (6hai)

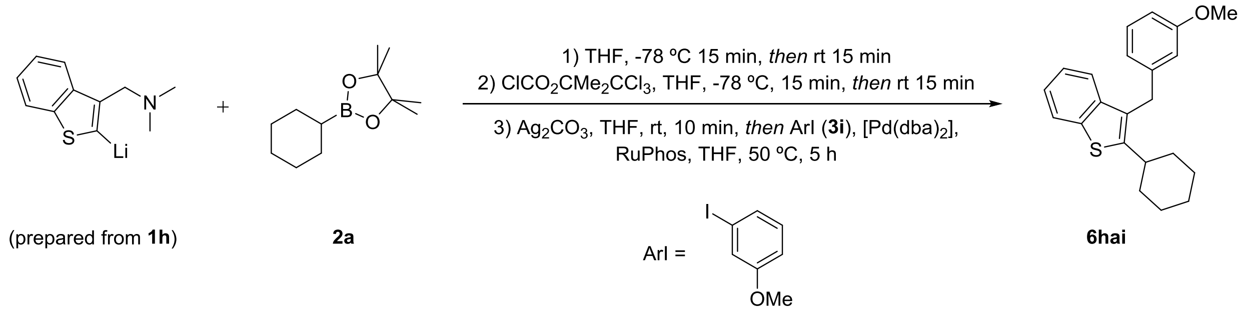

Prepared following general procedure A on a 300 µmol (34 µL) scale. Flash column chromatography on silica gel (toluene:hexane = 90:10) afforded the product as a colorless oil. Yield: 50 mg (49%). ***R*$_f$** (toluene:hexane = 91:9) = 0.10. **IR** (liquid film, cm^{-1}): 3056, 2998, 2923, 2849, 1745, 1608, 1599, 1583, 1488, 1448, 1436, 1348, 1311, 1278, 1256, 1144, 1072, 1050, 1021, 975, 888, 874, 846, 764, 739, 726, 692, 613, 574, 555, 443, 433. **^{1}H NMR** (400 MHz, CDCl$_3$, 25 ºC): δ (ppm) 7.82-7.75 (*m*, 1H, Ar–*H*), 7.54-7.46 (*m*, 1H, Ar–*H*), 7.28-7.20 (*m*, 2H, Ar–*H*), 7.19-7.13 (*m*, 1H, Ar–*H*), 6.79-6.69 (*m*, 3H, Ar–*H*), 4.18 (*s*, 2H, ArC*H$_2$*Ar), 3.74 (*s*, 3H, OC*H$_3$*), 3.08 (*tt*, $^3J_{H,H'}$ = 11.8, 3.5 Hz, 1H, Cy C*H*), 2.01-1.91 (*m*, 2H, Cy C*H$_2$*), 1.89-1.80 (*m*, 2H, Cy C*H$_2$*), 1.80-1.71 (*m*, 1H, Cy C*H$_2$*), 1.55 (*qd*, $^2J_{H,H'}$ = 12.3Hz, $^3J_{H,H'}$ = 12.3, 3.1 Hz, 2H, Cy C*H$_2$*), 1.44-1.10 (*m*, 3H, Cy C*H$_2$*). **^{13}C{^{1}H} NMR** (101 MHz, CDCl$_3$, 25 ºC): δ (ppm) 159.9 (*C*, arom.), 148.9 (*C*, arom.), 141.9 (*C*, arom.), 140.5 (*C*, arom.), 138.3 (*C*, arom.), 129.5 (*C*H, arom.), 127.5 (*C*, arom.), 124.0 (*C*H, arom.), 123.5 (*C*H, arom.), 122.4 (*C*H, arom.), 122.0 (*C*, arom.), 120.7 (*C*H, arom.), 114.3 (*C*H, arom.), 111.3 (*C*H, arom.), 55.3 (–O*C*H$_3$), 38.8 (Cy *C*H), 35.6 (Cy *C*H$_2$), 32.3 (Ar*C*H$_2$Ar), 26.9 (Cy *C*H$_2$), 26.0 (Cy *C*H$_2$). **HRMS** (MALDI): Calcd. For $C_{22}H_{24}OSNa$ *m/z* 359.1440. found *m/z* 359.1449 [M+Na]$^+$.

(*S*)-1-Cyclohexyl-2-(1-phenylethyl)benzene ((*S*)-6iaa)

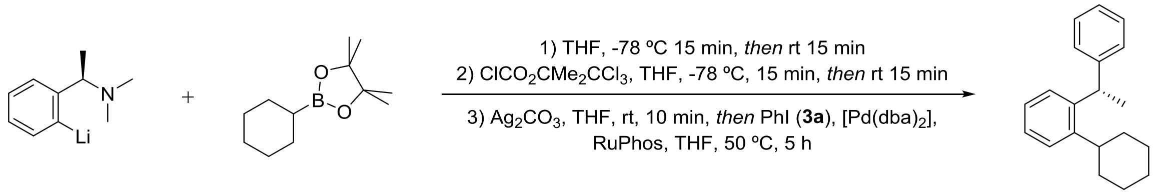

(prepared from **(*R*)-1i**) **2a** **6iaa**

Prepared following general procedure A on a 300 μmol (34 μL) scale. Yield: 50% (by ^{1}H NMR using 1,3,5-trimethoxybenzene as internal standard). Flash column chromatography on silica gel (gradient, hexane:toluene = 100:0 to 99: 1), afforded the product as a colorless oil, which co-eluted with minor inseparable unidentified impurities. 10 mg of the cleaner column fraction were used for analysis. **$[\alpha]_D^{23}$** = −0.15 (*c* 0.4, $CHCl_3$). ***R*f** (hexane) = 0.27. **IR** (liquid film, cm^{-1}): 2924, 2850, 1486, 1447, 1026, 752, 727, 698. **^{1}H NMR** (400 MHz, $CDCl_3$, 25 ºC; *Note*: despite purification, aliphatic region contains two additional protons by integration, reported as observed in ^{1}H NMR spectrum): δ (ppm) 7.28-7.14 (*m*, 9H, Ar–*H*), 4.47 (*q*, $^3J_{H,H'}$ = 7.2 Hz, 1H, ArC(CH_3)*H*Ar), 2.83 (*tt*, $^3J_{H,H'}$ = 11.4, 3.6 Hz, 1H, Cy C*H*), 1.87-1.67 (*m*, 4H, Cy C*H2*), 1.63 (*d*, $^3J_{H,H'}$ = 7.2 Hz, 3H, C*H*3), 1.51-1.15 (*m*, 8H, Cy C*H2*). **^{13}C{^{1}H} NMR** (101 MHz, $CDCl_3$, 25 ºC): δ (ppm) 147.1 (*C*, arom.), 145.8 (*C*H, arom.), 142.9 (*C*, arom.), 128.4 (2 *C*H, arom.), 127.8 (*C*H, arom.), 127.3 (2 *C*H, arom.), 126.44 (*C*H, arom.), 126.42 (*C*H, arom.), 125.9 (*C*H, arom.), 125.8 (*C*H, arom.), 40.3 (Ar*C*(CH_3)HAr), 39.5 (Cy *C*H), 34.5 (Cy *C*H_2), 34.4 (Cy *C*H_2), 27.4 (Cy *C*H_2), 27.2 (Cy *C*H_2), 26.4 (Cy *C*H_2), 22.7 (*C*H_3). **HRMS** (MALDI): Calcd. For $C_{20}H_{24}Na$ *m/z* 287.1770. found *m/z* 287.1777 [M+Na]$^+$. **HPLC**: Chiralpak IB, *two in series* (hexane:2-propanol = 100:0, flow rate 0.5 mL/min, λ = 210 nm), retention times t_R(minor) = 22.8 min, t_R(major) = 24.7 min; 9:91 e.r.; 82.5% ee.

Figure S6: HPLC chromatograms and data tables for compounds **(±)-6iaa** and **(*S*)-6iaa**, respectively.

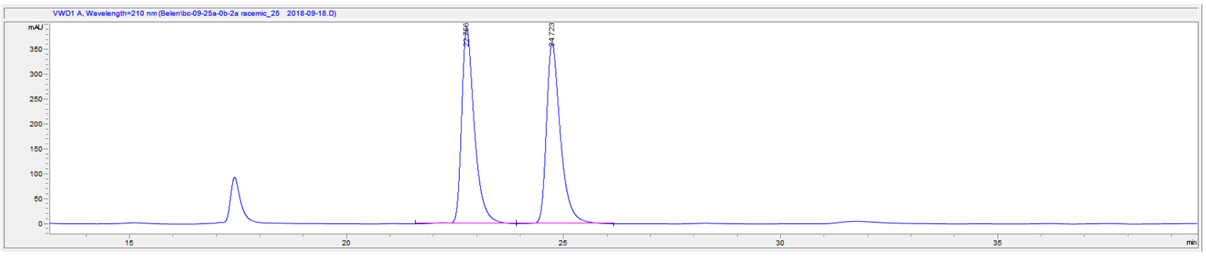

#	Time	Type	Area	Height	Width	Area%	Symmetry
1	22.756	VB R	8068.6	390.3	0.3085	50.002	0.627
2	24.723	BB	8067.9	361.5	0.3383	49.998	0.626

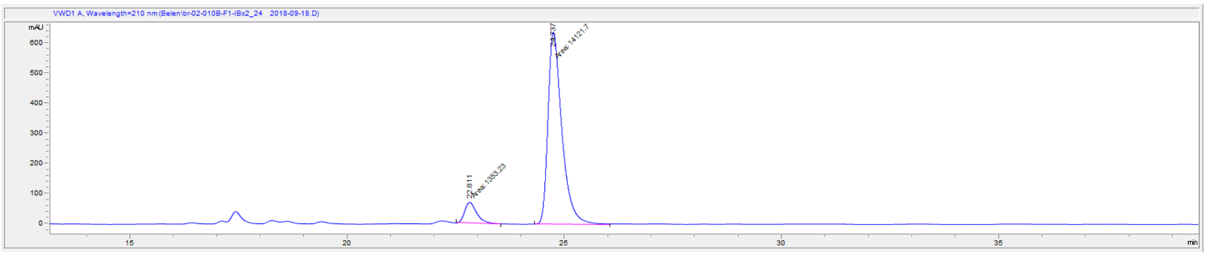

#	Time	Type	Area	Height	Width	Area%	Symmetry
1	22.811	MM	1353.2	69.2	0.3258	8.745	0.672
2	24.737	MM	14121.7	634.8	0.3708	91.255	0.605

1-((1*R*,2*S*,5*R*)-2-Isopropyl-5-methylcyclohexyl)-2-((*S*)-1-phenylethyl)benzene (6ima)

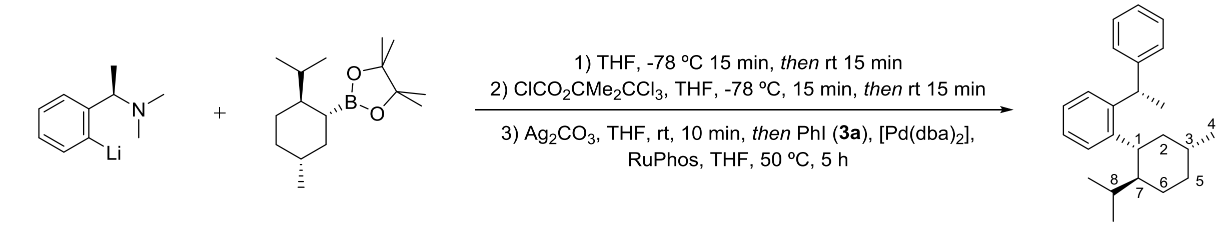

(prepared from **(*R*)-1i**) **2m** **6ima**

Prepared following general procedure A on a 300 μmol (34 μL) scale. Flash column chromatography on silica gel (hexane) afforded the product as a colorless oil. Yield: 39 mg (40%). ***R*f** (petrol ether:ethyl acetate = 91:9) = 0.74. **IR** (liquid film, cm^{-1}): 3061, 3025, 2952, 2923, 2868, 1600, 1494, 1486, 1385, 1368, 1046, 1027, 936, 907, 779, 753, 727, 697, 632, 617, 595, 581, 550, 467. **^{1}H NMR** (400 MHz, $CDCl_3$, 25 °C): δ (ppm) 7.33-7.05 (*m*, 9H, Ar–*H*), 4.50 (*q*, $^3J_{H,H'}$ = 7.2 Hz, 1H, ArC*H*(CH_3)Ar), 2.84 (*td*, $^3J_{H,H'}$ = 11.4 Hz, $^4J_{H,H'}$ = 3.3 Hz, 1H, Ar–C$^{(1)}$*H*), 1.88-1.78 (*br m*, 2H, –C$^{(2)}H_2$– and –C$^{(5)}H_2$–), 1.77-1.68 (*m*, 1H, –C$^{(6)}H_2$), 1.66-1.36 (*m*, 3H, –C$^{(7)}$*H*–, –C$^{(3)}$*H*– and –C$^{(8)}$*H*–), 1.59 (*d*, $^3J_{H,H'}$ = 7.2 Hz, 3H, ArCH(CH_3)Ar), 1.16-0.95 (*m*, 3H,

–C⁽²⁾H₂–, –C⁽⁵⁾H₂– and –C⁽⁶⁾H₂–), 0.91 (*d*, $^3J_{H,H'}$ = 6.5 Hz, 3H, –C⁽⁴⁾H₃), 0.77 (*d*, $^3J_{H,H'}$ = 7.0 Hz, 3H, isopropyl –C*H*₃), 0.22 (*d*, $^3J_{H,H'}$ = 7.2 Hz, 3H, isopropyl –C*H*₃). **^{13}C{^{1}H} NMR** (101 MHz, $CDCl_3$, 25 ºC): δ (ppm) 146.9 (*C*, arom.), 144.4 (*C*, arom.), 143.8 (*C*, arom.), 128.5 (*C*H, arom.), 128.1 (*C*H, arom.), 127.9 (*C*H, arom.), 126.4 (*C*H, arom.), 126.3 (*C*H, arom.), 126.0 (*C*H, arom.), 125.5 (*C*H, arom.), 46.8 ($C^{(7)}H$), 46.6 ($C^{(2)}H_2$), 41.8 ($C^{(1)}H$), 39.8 (Ar*C*H(CH_3)Ar), 35.6 ($C^{(5)}H_2$), 33.7 ($C^{(3)}H$), 27.3 `($C^{(8)}H$), 25.1 ($C^{(6)}H_2$), 23.3 (ArCH(*C*H_3)Ar), 22.7 ($C^{(4)}H_3$), 22.2 (isopropyl CH_3), 15.8 (isopropyl CH_3). **HRMS** (MALDI): Calcd. for $C_{24}H_{32}Na$ *m/z* 343.2396 found *m/z* 343.2384 $[M+Na]^+$.

1-((1*R*,2*S*,5*R*)-2-isopropyl-5-methylcyclohexyl)-2-((*R*)-1-phenylethyl)benzene (6ima')

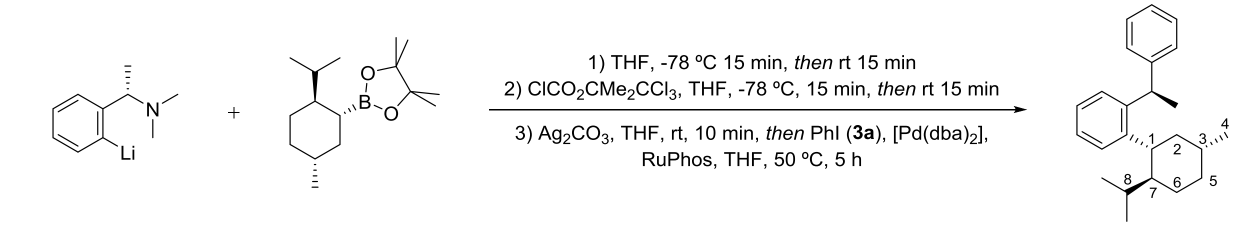

Prepared following general procedure A on a 300 μmol (34 μL) scale. Flash column chromatography on silica gel (hexane) afforded the product as a white solid. Yield: 42.6 mg (44%). **m.p.** = 67.0-68.1 ºC. ***R*f** (petrol ether:ethyl acetate = 91:9) = 0.73. **IR** (liquid film, cm^{-1}): 3061, 3020, 2955, 2940, 2921, 2871, 2860, 1600, 1583, 1493, 1445, 1383, 1363, 1336, 1178, 1078, 1045, 1026, 937, 908, 786, 753, 727, 701, 631, 616, 579, 548, 457. **^{1}H NMR** (400 MHz, $CDCl_3$, 25 ºC): δ (ppm) 7.32-7.23 (*m*, 1H, Ar–*H*), 7.22-7.00 (*m*, 8H, Ar–*H*), 4.42 (*q*, $^3J_{H,H'}$ = 7.1 Hz, 1H, ArC*H*(CH_3)Ar), 2.75 (*td*, $^3J_{H,H'}$ = 11.3, 3.4 Hz, 1H, Ar–$C^{(1)}H$), 1.76-1.62 (*m*, 2H, –$C^{(5)}H_2$– and –$C^{(6)}H_2$–), 1.57 (*d*, $^3J_{H,H'}$ = 7.1 Hz, 3H, ArCH(C*H*₃)Ar), 1.54-1.39 (*m*, 2H, –$C^{(7)}H$– and –$C^{(8)}H$–), 1.15-1.00 (*m*, 2H, –$C^{(3)}H$– and –$C^{(6)}H_2$–), 0.94-0.79 (*m*, 2H, –$C^{(2)}H_2$– and –$C^{(5)}H_2$–), 0.76 (*d*, $^3J_{H,H'}$ = 6.9 Hz, 3H, isopropyl –C*H*₃), 0.73-0.63 (*m*, 1H, –$C^{(2)}H_2$–), 0.67 (*d*, $^3J_{H,H'}$ = 6.8 Hz, 3H, isopropyl –C*H*₃), 0.58 (*d*, $^3J_{H,H'}$ = 6.5 Hz, 3H, –$C^{(4)}H_3$). **^{13}C{^{1}H} NMR** (101 MHz, $CDCl_3$, 25 ºC): δ (ppm) 147.6 (*C*, arom.), 144.5 (*C*, arom.), 143.3 (*C*, arom.), 128.4 (*C*H, arom.), 127.9 (*C*H, arom.), 126.8 (*C*H, arom.), 126.6 (*C*H, arom.), 126.5 (*C*H, arom.), 125.8 (*C*H, arom.), 125.5 (*C*H, arom.), 47.2 ($C^{(7)}H$), 45.4 ($C^{(2)}H_2$), 41.9 ($C^{(1)}H$), 40.6 (Ar*C*H(CH_3)Ar), 35.5 ($C^{(5)}H_2$), 33.6 ($C^{(3)}H$), 27.6 ($C^{(8)}H$), 25.1 ($C^{(6)}H_2$), 23.0 (ArCH(*C*H_3)Ar), 22.5 (isopropyl CH_3), 22.0 (isopropyl CH_3), 16.6 ($C^{(4)}H_3$). **HRMS** (MALDI): Calcd. for $C_{24}H_{32}Na$ *m/z* 343.2396 found *m/z* 343.2388 $[M+Na]^+$.

X-ray structure of **6ima'**: Clear crystals of **6ima'** were obtained from methanol/hexanes. See Section 8 for crystallography data.

1-((*S*)-4-phenylbutan-2-yl)-2-((*S*)-1-phenylethyl)benzene (6ina)

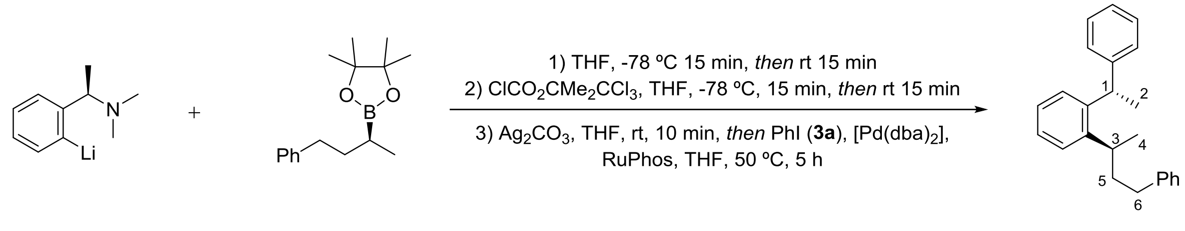

(prepared from (***R***)-**1i**) **2n** **6ina**

Prepared following general procedure A on a 150 μmol (17 μL) scale. Flash column chromatography on silica gel (9% toluene in hexane) afforded the product as a colourless oil. Yield: 15.8 mg (33%). ***R*f** (petrol ether:ethyl acetate = 91:9) = 0.61. **IR** (liquid film, cm^{-1}): 3062, 3023, 2964, 2928, 1600, 1493, 1451, 1374, 1026, 912, 756, 697, 631, 553, 541. **^{1}H NMR** (400 MHz, $CDCl_3$, 25 ºC): δ (ppm) 7.36-7.16 (*m*, 9H, Ar-*H*), 7.16-7.09 (*m*, 3H, Ar-*H*), 7.01 (*d*, $^3J_{H,H'}$ = 7.0 Hz, 2H, Ar-*H*), 4.22 (*q*, $^3J_{H,H'}$ = 7.1 Hz, 1H, –C$^{(1)}$*H*Me–), 3.04 (*h*, $^3J_{H,H'}$ = 7.0 Hz, 1H, –C$^{(3)}$*H*Me–), 2.64-2.43 (*m*, 2H, –C$^{(6)}H_2$–), 1.99-1.83 (*m*, 2H, –C$^{(5)}H_2$–), 1.55 (*d*, $^3J_{H,H'}$ = 7.2 Hz, 3H, –C$^{(2)}H_3$), 0.94 (*d*, $^3J_{H,H'}$ = 6.8 Hz, 3H, –C$^{(4)}H_3$). **^{13}C{^{1}H} NMR** (101 MHz, $CDCl_3$, 25 ºC): δ (ppm) 147.2 (*C*, arom.), 145.5 (*C*, arom.), 143.1 (*C*, arom.), 142.6 (*C*, arom.), 128.6 (*C*H, arom.), 128.5 (*C*H, arom.), 128.4 (*C*H, arom.), 127.8 (*C*H, arom.), 127.2 (*C*H, arom.), 126.7 (*C*H, arom.), 126.1 (*C*H, arom.), 125.92 (*C*H, arom.), 125.85 (*C*H, arom.), 125.83 (*C*H, arom.), 40.3 (C$^{(1)}$H), 40.0 (C$^{(5)}H_2$), 34.1 (C$^{(6)}H_2$), 33.0 (C$^{(3)}$H), 22.8 (C$^{(2)}H_3$), 22.1 (C$^{(4)}H_3$). **HRMS** (MALDI): Calcd. for $C_{24}H_{26}Na$ *m/z* 337.1927 found *m/z* 337.1931 $[M+Na]^+$.

1-((*S*)-4-phenylbutan-2-yl)-2-((*R*)-1-phenylethyl)benzene (6ina')

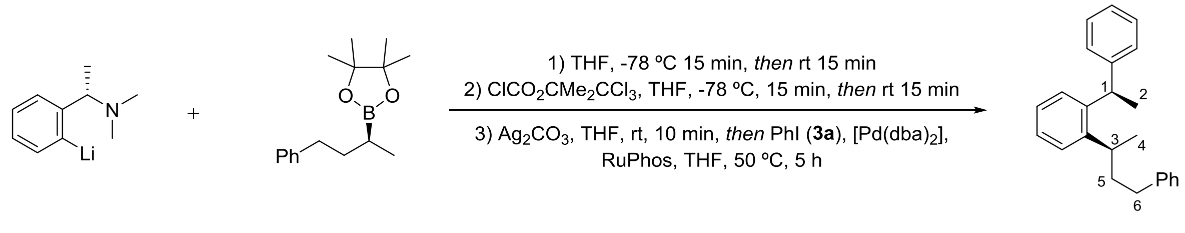

(prepared from (***S***)-**1i**) **2n** **6ina'**

Prepared following general procedure A on a 150 μmol (17 μL) scale. Flash column chromatography on silica gel (9% toluene in hexane) afforded the product as a colourless oil. Yield: 19.2 mg (41%). ***R*f** (petrol ether:ethyl acetate = 91:9) = 0.67. **IR** (liquid film, cm^{-1}): 3067, 3027, 2963, 2928, 2862, 1605, 1493, 1450, 1374, 1026, 755, 744, 697, 629, 553, 542. **^{1}H NMR** (400 MHz, $CDCl_3$, 25 ºC): δ (ppm) 7.39-7.06 (*m*, 12H, Ar-*H*), 7.02-6.92 (*m*, 2H, Ar-*H*), 4.44 (*q*, $^3J_{H,H'}$ = 7.2 Hz, 1H, –C$^{(1)}$*H*Me–), 3.15 (*h*, $^3J_{H,H'}$ = 7.0 Hz, 1H, –

$C^{(3)}H$Me–), 2.24-2.11 (*m*, 2H, –$C^{(6)}H_2$–), 1.80-1.66 (*m*, 2H, –$C^{(5)}H_2$–), 1.61 (*d*, $^3J_{H,H'}$ = 7.1 Hz, 3H, –$C^{(2)}H_3$), 1.26 (*d*, $^3J_{H,H'}$ = 6.9 Hz, 3H, –$C^{(4)}H_3$). **^{13}C{^{1}H} NMR** (101 MHz, $CDCl_3$, 25 ºC): δ (ppm) 147.1 (*C*, arom.), 145.5 (*C*, arom.), 143.0 (*C*, arom.), 142.9 (*C*, arom.), 128.5 (*C*H, arom.), 128.4 (2 *C*H, arom.), 128.0 (*C*H, arom.), 127.4 (*C*H, arom.), 126.7 (*C*H, arom.), 126.02 (*C*H, arom.), 125.97 (*C*H, arom.), 125.91 (*C*H, arom.), 125.7 (*C*H, arom.), 40.3 ($C^{(1)}H$), 40.1 ($C^{(5)}H_2$), 34.2 ($C^{(6)}H_2$), 33.7 ($C^{(3)}H$), 22.9 ($C^{(2)}H_3$), 22.7 ($C^{(4)}H_3$). **HRMS** (MALDI): Calcd. for $C_{24}H_{26}Na$ *m/z* 337.1927 found *m/z* 337.1935 $[M+Na]^+$.

8 Crystallographic data for product 6ima’

X-ray diffraction experiments on **6ima’** was carried out at 100(2) K on a Bruker APEX II CCD diffractometer using Mo-K_α radiation (λ = 0.71073 Å). Intensities were integrated in SAINT[S9] and absorption corrections based on equivalent reflections were applied using SADABS.[S10] Structures **6ima’** was solved using ShelXT[S11] and refined by full matrix least squares against F^2 in ShelXL[S12,S13] using Olex2.[S14] All of the non-hydrogen atoms were refined anisotropically. While all of the hydrogen atoms were located geometrically and refined using a riding model. The absolute structure was not determined. Crystal structure and refinement data are given in Table 1. Crystallographic data for **6ima’** have been deposited with the Cambridge Crystallographic Data Centre as supplementary publication CCDC 1869423. Copies of the data can be obtained free of charge on application to CCDC, 12 Union Road, Cambridge CB2 1EZ, UK [e-mail: deposit@ccdc.cam.ac.uk].

Figure S7: Illustration of the structure of **6ima** with the atomic numbering scheme depicted. Anisotropic displacement parameters depicted at the 50% probability level. Only one of the two unique molecules in the asymmetric unit is shown as both had the same conformation. Only hydrogen atoms at stereocenters are shown.

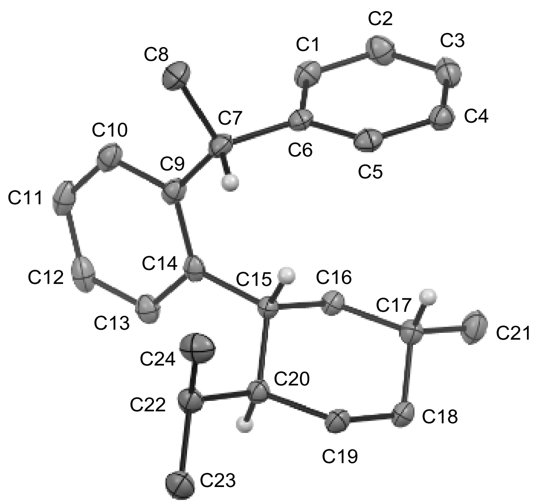

Table S7: Crystal data and structure refinement for **6ima'**.

Identification code	**6ima**
Empirical formula	$C_{24}H_{32}$
Formula weight	320.49
Temperature/K	100(2)
Crystal system	monoclinic
Space group	*C*2
a/Å	19.0802(5)
b/Å	8.4059(2)
c/Å	26.0029(9)
α/°	90
β/°	110.7770(10)
γ/°	90
Volume/Å^3	3899.29(19)
Z	8
ρ_{calc}g/cm^3	1.092
μ/mm^{-1}	0.061
F(000)	1408.0
Crystal size/mm^3	0.391 × 0.253 × 0.228
Radiation	MoKα (λ = 0.71073)
2θ range for data collection/°	4.27 to 55.886
Index ranges	–25 ≤ h ≤ 25, –11 ≤ k ≤ 10, –33 ≤ l ≤ 34
Reflections collected	17339
R_{int} / R_{sigma}	0.0355 / 0.0608
Data/restraints/parameters	9266/1/441
Goodness-of-fit on F^2	1.017
Final R indexes [I>=2σ (I)]	R_1 = 0.0540, wR_2 = 0.1152
Final R indexes [all data]	R_1 = 0.0830, wR_2 = 0.1281
Largest diff. peak/hole / e Å^{-3}	0.24/–0.22

9 Characterization of rotamers 6aaa-R_A and 6aaa-R_B

The identification and characterization of rotameric species **6aaa-*R*$_A$** and **6aaa-*R*$_B$** was addressed following two pathways. On one hand, EXSY/NOESY NMR experiments helped to unmistakably identify the two species present in the ^{1}H NMR spectrum as rotamers, as well as infer the structure of both. On the other hand, variable temperature (VT) ^{1}H NMR experiments allowed us to again confirm the existence of chemical exchange by observing coalescence, as well as find the coalescence temperature, and the rotation rate and barrier to rotation values.

An NMR sample of **6aaa** was prepared in $CDCl_3$ and an EXSY/NOESY experiment was carried out using 400 ms as mixing time to assure the transference of magnetization between the species that were, presumably, presenting chemical exchange.

Figure S8: EXSY/NOESY experiment for compound **6aaa**, existing as a mixture of two rotamers, **6aaa-*R*$_A$** and **6aaa-*R*$_B$**.

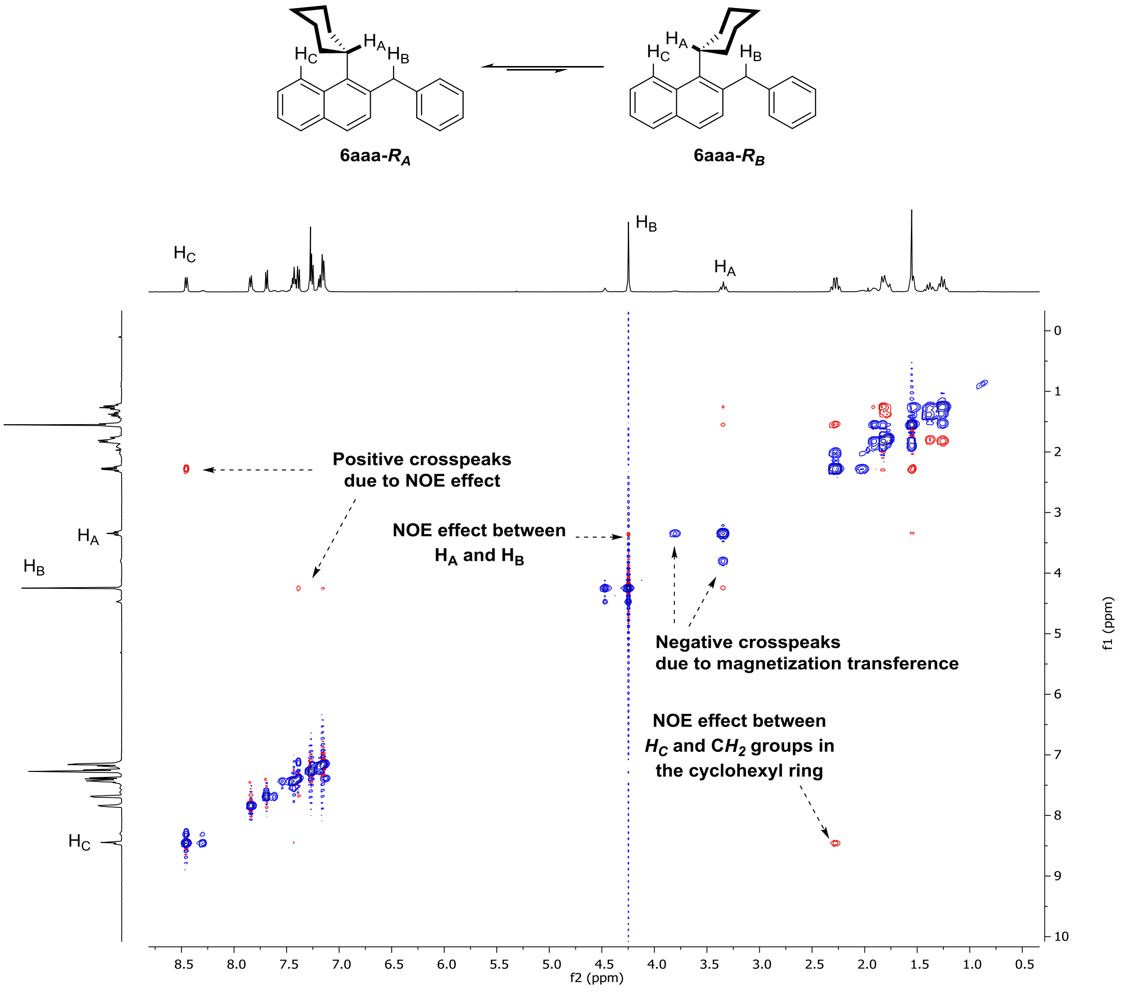

As shown in Figure S8, negative cross peaks (blue, in the same phase as the diagonal peaks) indicate the presence of two species presenting chemical exchange. Moreover, considering the nature of the hydrogen atoms in **6aaa**, this exchange can only be due to a rotational barrier in some of the bonds within the molecule. Positive cross peaks (red, in the opposite phase to the diagonal peaks) corresponding to NOE enhancement effects, show a major species in which proton A is close to proton B, and the *peri* hydrogen (proton C) is close to the methylene groups of the cyclohexyl ring. The minor rotamer NOE peaks are not visible due to the low concentration of this species with respect to the major one. These results allowed us to assign the major and minor rotamer structures as **6aaa-*R*$_A$** and **6aaa-*R*$_B$**, respectively (see Figure S8), where the interconversion occurs through rotation about the naphthyl-cyclohexyl bond.

Figure S9: ^{1}H NMR of **6aaa** at room temperature, 50, 55, 60, 65, 70, and 75 °C (500 MHz, d_6-DMSO).

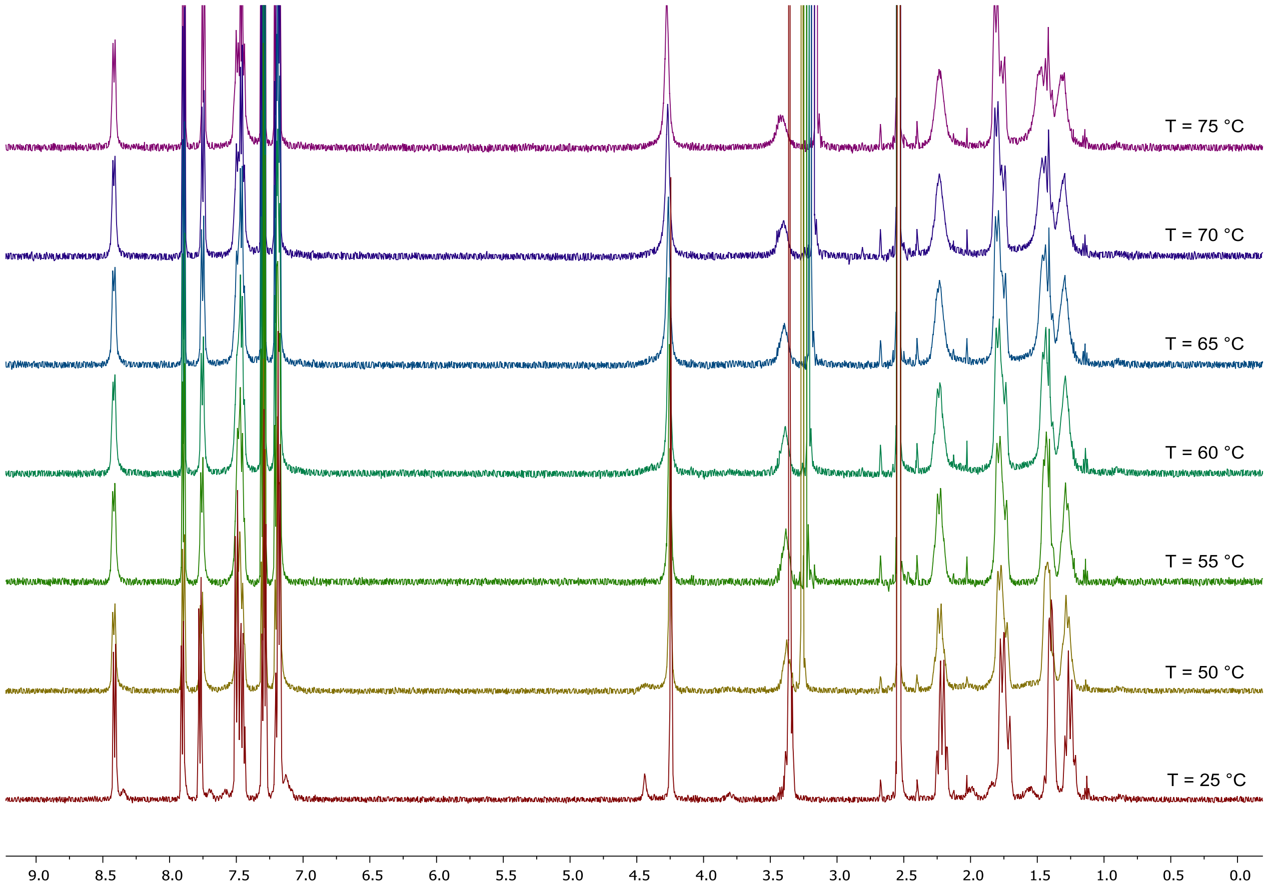

Then, compound **6aaa** was solved in d_6-DMSO and variable temperature ^{1}H NMR experiments were carried out heating in 25 °C intervals, starting at 25 °C and increasing up to 50, 75, 100 and 125 °C, respectively. Another ^{1}H NMR experiment back to 25 °C confirmed no decomposition of the sample. It was observed that the coalescence temperature for the rotation exchange was between 50 and 75 °C. Subsequently, a series of ^{1}H NMR were performed between 50 and 75 °C, heating in 5 °C intervals. Stacked spectra for these VT ^{1}H NMR experiments can be seen in Figure S9, and show that the coalescence temperature, defined as the lowest temperature at which the minimum between the two resonances became a saddle point,[S15] can be established at, approximately, 55 °C.

For the case of two unequaly populated sites, at the coalescence temperature, Shanan-Atidi and Bar-Eli have developed a convenient graphical method to obtain the free energies of activation of both pathways $A_{(major)} \rightleftharpoons B_{(minor)}$ or $B_{(minor)} \rightleftharpoons A_{(major)}$, as well as their respective exchange rates.[S16]

In our case, **6aaa-*R*$_A$** and **6aaa-*R*$_B$** are our major and minor species (A and B), presenting fractional populations $P_A = 0.87$ and $P_B = 0.13$, respectively, and a difference in shift $\Delta v_{(d_6-DMSO)} = |v_A - v_B| = |2219.61 - 2122.03| = 97.58$ Hz. The free energy for both rotation pathways is given by Eyring's expressions (equations (1) and (2)), in cal/mol, where $\Delta P = P_A - P_B$ and $X = 2\pi\tau\Delta v$, and τ is defined by the relation $(1/\tau) = (1/\tau_A) + (1/\tau_B)$, where τ_A and τ_B are the lifetimes of species A and B, respectively.

$$\Delta G^{\ddagger}_{A\to B} = 4.57T_C\left[10.62 + \log\left(\frac{X}{2\pi(1-\Delta P)}\right) + log\left(\frac{T_C}{\Delta v}\right)\right] \quad (1)$$

$$\Delta G^{\ddagger}_{B\to A} = 4.57T_C\left[10.62 + \log\left(\frac{X}{2\pi(1+\Delta P)}\right) + log\left(\frac{T_C}{\Delta v}\right)\right] \quad (2)$$

Using Shanan-Atidi and Bar-Eli's plot for calculated values of $\tau\Delta v$ vs. ΔP,[S16] a value of $\tau\Delta v = 0.41$ s for $\Delta P = 0.83 - 0.17 = 0.74$ is obtained. Therefore, $X = 2.58$. Alternatively, Shanan-Atidi and Bar-Eli's plot for $\log(X/2\pi(1 \pm \Delta P))$ vs. ΔP can be used in the same manner[S16] to obtain values of $\log(X/2\pi(1 + \Delta P)) = -0.63$ and $\log(X/2\pi(1 - \Delta P)) = 0.20$. Both options give the same outcome when substituting in equations (1) and (2) to obtain $\Delta G^{\ddagger}_{A\to B} = 17.0$ kcal/mol and $\Delta G^{\ddagger}_{B\to A} = 15.8$ kcal/mol. This means a difference in free energy between species A and B of 1.2 kcal/mol.

Moreover, the rate constant of both pathways $A_{(major)} \rightleftharpoons B_{(minor)}$ and $B_{(minor)} \rightleftharpoons A_{(major)}$ can be obtained from equations (3) and (4):

$$k_{A\rightarrow B} = \frac{1}{\tau}(1 - \Delta P) \quad (3)$$

$$k_{B\rightarrow A} = \frac{1}{\tau}(1 + \Delta P) \quad (4)$$

Having obtained from Shanan-Atidi and Bar-Eli's plot for $\tau\Delta\nu$ vs. ΔP the value $\tau\Delta\nu = 0.41$ s, it is possible to infer $\tau = 4.2 \cdot 10^{-3}$s. Substitution of every value in equations (3) and (4) leads to $k_{A\rightarrow B} = 30.9$ Hz and $k_{B\rightarrow A} = 207.1$ Hz as rotation rates.

10 Analysis of the enantiospecificity of the process in its application to the synthesis of 1,1-diarylethanes

10A Procedure for the obtention of product **6iaa** *(this work):*

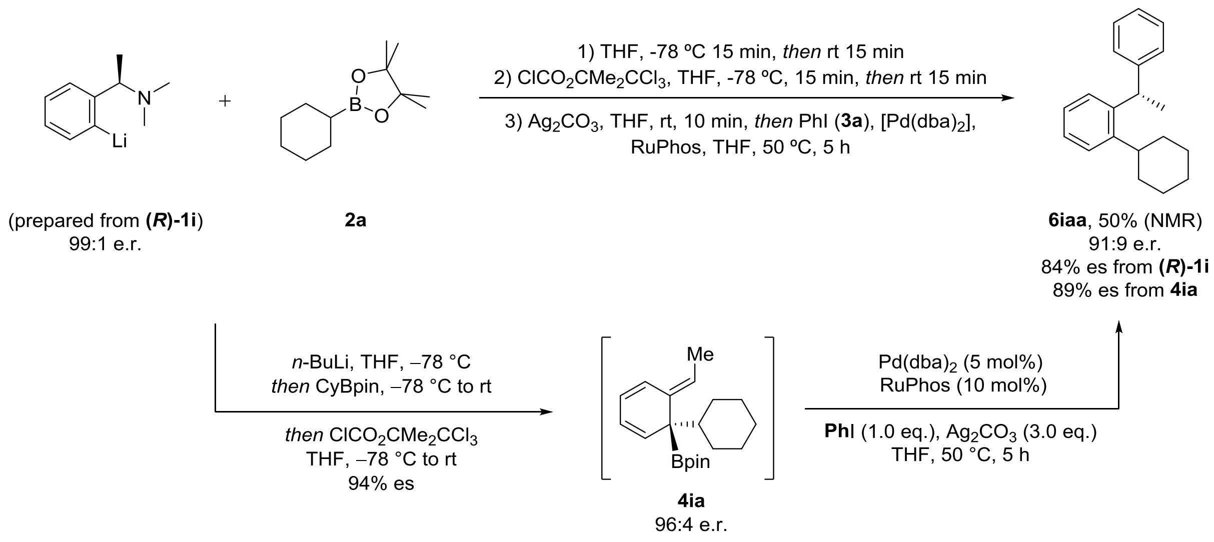

Following general procedure A, on a 300 µmol scale, and using enantioenriched α-methyl benzylamine **(*R*)-1i** (99:1 e.r.), cyclohexylboronic ester **2a** and phenyl iodide **3a** as substrates, product **6iaa** was afforded in 50% yield as determined by ^{1}H NMR and 91:9 e.r. (**HPLC**: Chiralpak IB, *two in series*, (hexane:2-propanol = 100:0, flow rate 0.5 mL/min, λ = 210 nm), retention times t_R(minor) = 22.8 min, t_R(major) = 24.7 min; 9:91 e.r., 82.5% ee, see Product Characterization for full details, page S20), corresponding to an enantioespecificity of 84% es from **(*R*)-1i** (Figure S10).

Previous studies in our group have shown that a the 1,2-migration/anti-S_N2' elimination of secondary alkyl groups en route to intermediates similar to **4ia** proceed in 94% es.[S3] Therefore, the enantiospecifity for the last step of the sequence, the palladium catalyzed rearomatizing γ-selective allylic Suzuki-Miyaura cross-coupling, is 89% es.

Figure S10: HPLC chromatograms and data tables for compounds **(±)-6iaa** and **(*R*)-6iaa**, respectively, obtained through our reaction conditions.

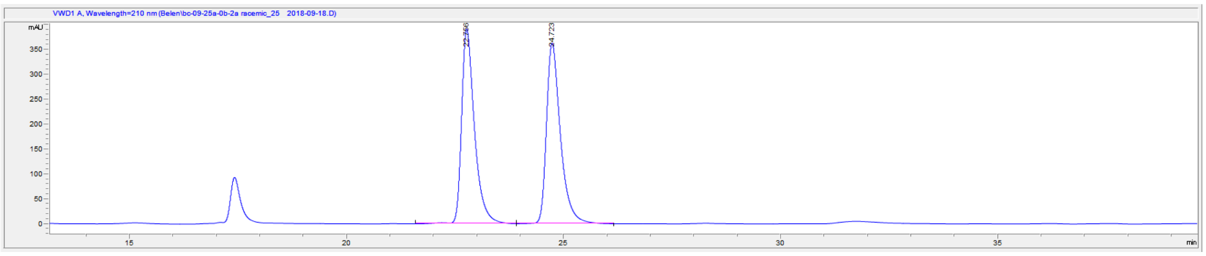

#	Time	Type	Area	Height	Width	Area%	Symmetry
1	22.756	VB R	8068.6	390.3	0.3085	50.002	0.627
2	24.723	BB	8067.9	361.5	0.3383	49.998	0.626

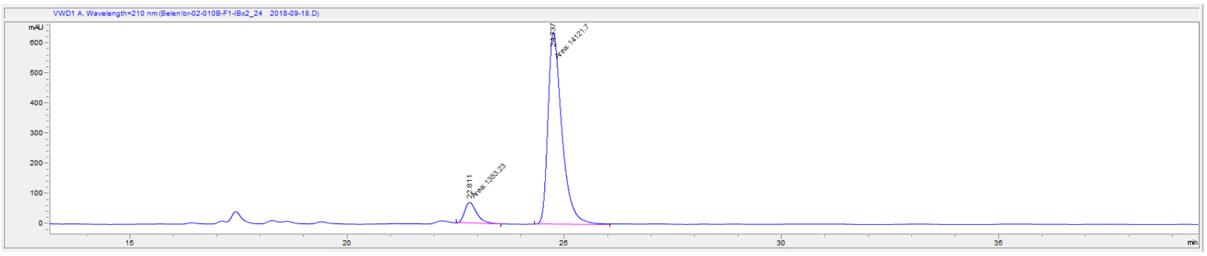

#	Time	Type	Area	Height	Width	Area%	Symmetry
1	22.811	MM	1353.2	69.2	0.3258	8.745	0.672
2	24.737	MM	14121.7	634.8	0.3708	91.255	0.605

10B Procedure for obtaining product 6iaa *via Crudden's conditions:*

(*R*)-2-(1-(2-cyclohexylphenyl)ethyl)-4,4,5,5-tetramethyl-1,3,2-dioxaborolane (5ia)

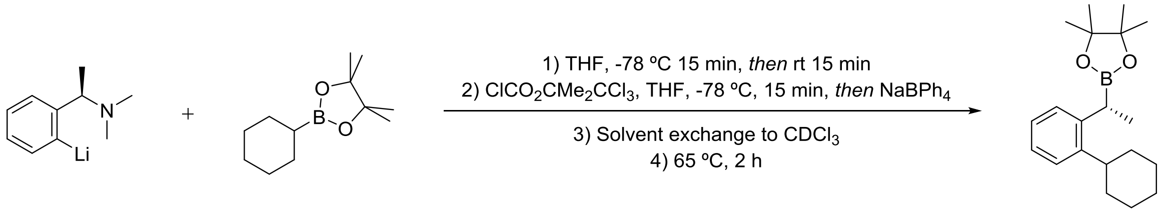

(prepared from **(*R*)-1i**) 99:1 e.r. — **2a** — **5ia**, 50%

Prepared according to a literature procedure in 50% yield on a 1.5 mmol scale.[S3]

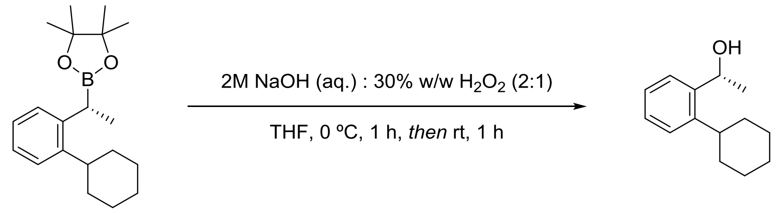

5ia

9ia, 83%
94:6 e.r.

The enantiomeric excess was determined after oxidation to the corresponding alcohol, as previously reported by our group.[S3] **HPLC**: Chiralpak IC (hexane:2-propanol = 99:1, flow rate 1.0 mL/min, λ = 210 nm), retention times t_R(minor) = 8.7 min, t_R(major) = 9.8 min; 6:94 e.r., 87.8%ee.

Figure S11: HPLC chromatograms and data tables for compounds **(±)-9ia** and **(*R*)-9ia**, respectively.

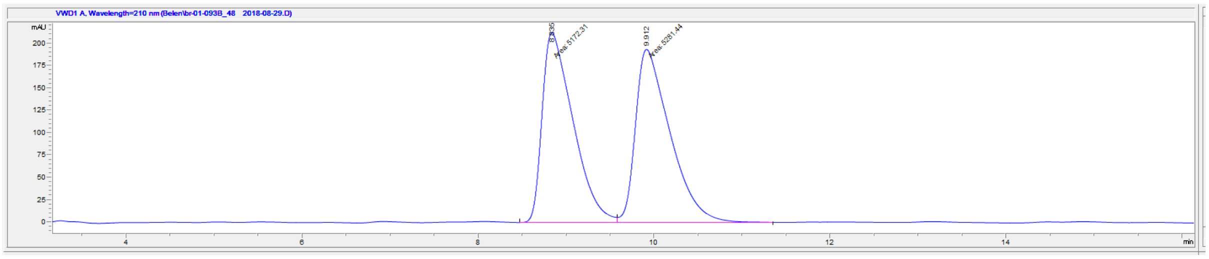

#	Time	Type	Area	Height	Width	Area%	Symmetry
1	8.835	MF	5172.3	212	0.4066	49.478	0
2	9.912	FM	5281.4	193.4	0.4552	50.522	0.463

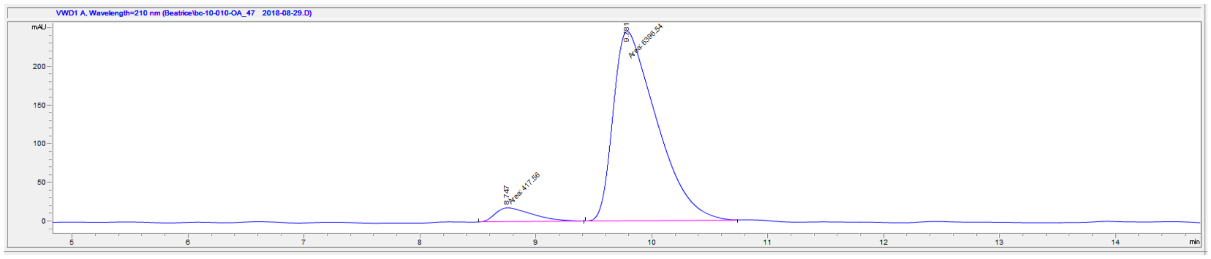

#	Time	Type	Area	Height	Width	Area%	Symmetry
1	8.747	MM	417.6	18.3	0.3805	6.128	0.461
2	9.781	MM	6396.5	245	0.4352	93.872	0.46

For comparison purposes, α-methyl benzylic boronic ester **5ia** was subjected to Crudden's optimized conditions for his palladium-catalyzed Suzuki-Miyaura coupling of benzylic boronic esters[S17] on a 300μmol scale.

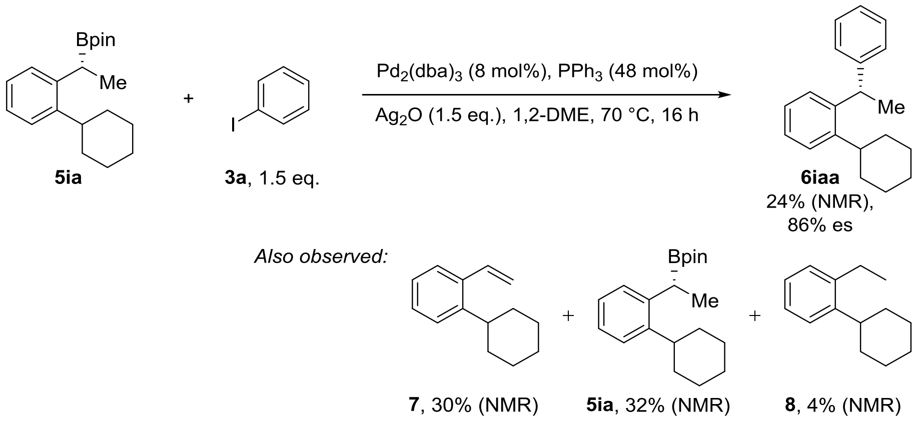

For this conditions, **6iaa** was obtained in 24% yield (as determined by ^{1}H NMR using 1,3,5-trimethoxybenzene as internal standard). It was also observed β-hydride elimination product **7** (30% yield), returning starting boronic ester **5ia** (32% yield) and protodeboronation product **8** (4% yield) in the crude reaction mixture. The identity of **7** and **8** was confirmed by GCMS analysis. **HPLC**: Chiralpak IB (hexane:2-propanol = 100:0, flow rate 0.7 mL/min, λ = 210 nm), retention times t_R(minor) = 8.3 min, t_R(major) = 9.1 min; 88:12 e.r.. Therefore, the enatioespecificity for this process is 86% es.

Figure S12: HPLC chromatograms and data tables for compounds **(±)-6iaa** and **(*R*)-6iaa**, respectively, obtained through Crudden’s cross coupling conditions.

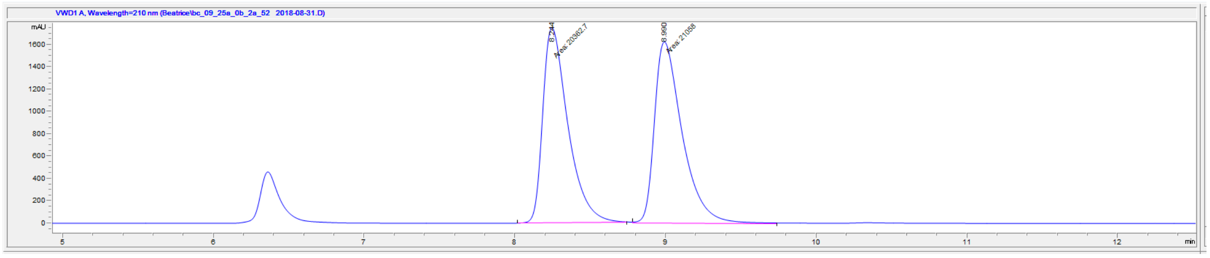

#	Time	Type	Area	Height	Width	Area%	Symmetry
1	8.244	MM	20362.7	1745	0.1945	49.161	0.549
2	8.99	MM	21058	1623.3	0.2162	50.839	0.53

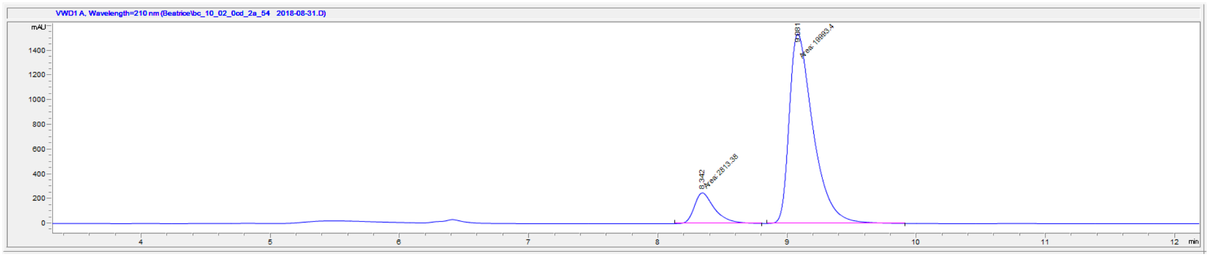

#	Time	Type	Area	Height	Width	Area%	Symmetry
1	8.342	MM	2813.4	251	0.1868	12.336	0.618
2	9.081	MM	19993.4	1545.7	0.2156	87.664	0.532

11 Mechanistic investigations on the γ-selective allylic transmetallation vs. 1,3-borotropic shift/direct cross-coupling pathways

For this transformation, we propose an indirect coupling via a γ-selective allylic transmetallation, already reported in the literature (Ref. [11] on the main article), rather than a 1,3-borotropic shift[S3] followed by a direct cross-coupling on the benzylic boronic ester. This is based on the experiments implemented in order to gain further insight into the reaction pathway.

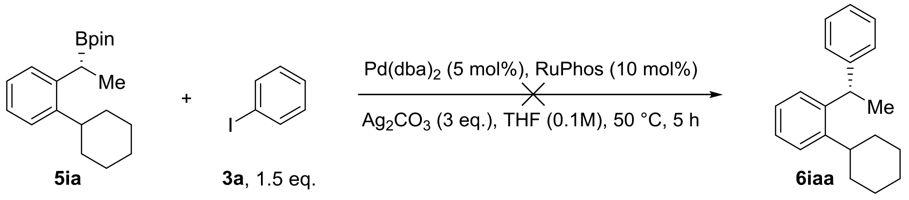

In first place, benzylic boronic ester **5ia**, which was synthesized as already mentioned (section 10B, page S53), was subjected to our coupling conditions. Product **6iaa** was not observed and the starting boronic ester **5ia** was recovered in 84% yield (as determined by ^{1}H NMR using 1,3,5-trimethoxybenzene as internal standard). This suggests that a direct coupling on the benzylic boronic ester is not possible under our reaction conditions.

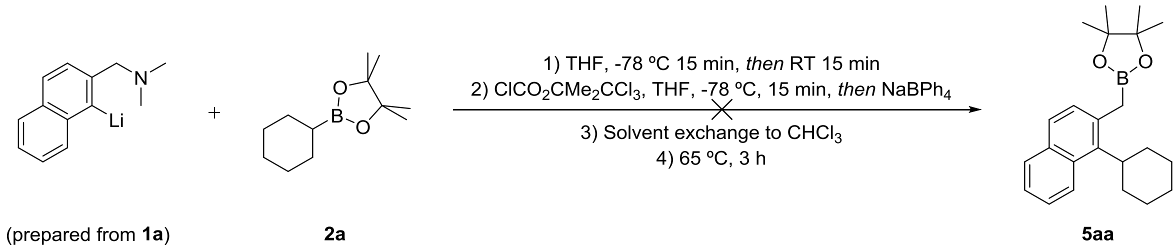

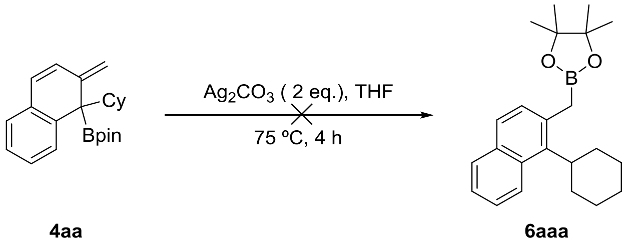

In second place, naphthylic amine **1a** is stable with respect to 1,3-borotropic shift, as it has been unsuccessfully subjected to our previously reported conditions for the synthesis of benzylic boronic esters.[S3] No compound **5aa** was observed and 75% of intermediate **4aa** was recovered (as determined by ^{1}H NMR using 1,3,5-trimethoxybenzene as internal standard). Moreover, having synthesized intermediate **4aa** as

previously indicated (see section 5, page S14), it was heated at 75 ºC for 4 h in the presence of silver carbonate, in analogy to our optimized allylic cross-coupling conditions, observing no evidence of 1,3-borotropic shift product. Also 75% of intermediate **4aa** was recovered (as determined by ^{1}H NMR using the same internal standard).

According to these results, we can exclude a direct coupling on the benzylic boronic ester for naphthylic of secondary benzylamine substrates.

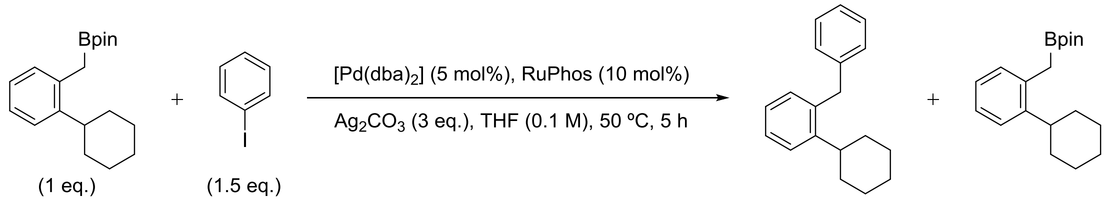

5ba **3a** **6baa** 21% (NMR) (67% isolated yield from **1b**, under our standard one-pot conditions) Returning **5ba** 21% (NMR)

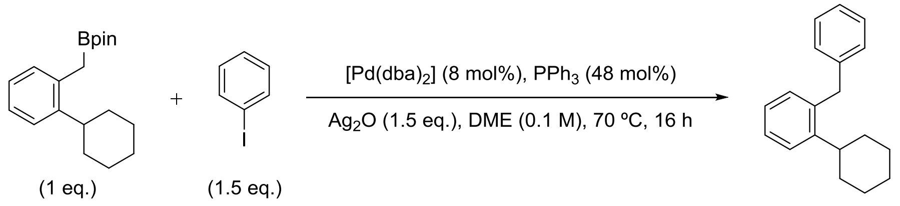

5ba **3a** **6baa** 83% (NMR)

Regarding primary benzylamines, when primary boronic ester **5ba**, accessed from amine **1b** and boronic ester **2a** via our 1,3-borotropic shift methodology,[S3] was subjected to our standard coupling conditions, the corresponding cross-coupling product **6baa** was obtained in 21% yield, along with 21% of returning starting boronic ester **5ba** (determined by ^{1}H NMR using 1,3,5-trimethoxybenzene as internal standard). Using the conditions reported by Crudden,[S17] **6baa** was access in 83% NMR yield. Therefore, we cannot rule out a minor pathway via a 1,3-borotropic shift plus a direct Suzuki-Miyaura cross-coupling for primary benzylamines like **1b**.

12 References

(S1) Staniland, S.; Adams, R. W.; McDouall, J. J. W.; Maffucci, I.; Contini, A.; Grainger, D. M.; Turner, N. J.; Clayden, J. *Angew. Chem. Int. Ed.* **2016**, *55*, 10755-10759.

(S2) Ruzziconi, R.; Lepri, S.; Buonerba, F.; Schlosser, M.; Mancinelli, M.; Ranieri, S.; Prati, L.; Mazzanti, A. *Org. Lett.* **2015**, *17*, 2740-2743.

(S3) Aichhorn, S.; Bigler, R.; Myers, E.; Aggarwal, V. K. *J. Am. Chem. Soc.* **2017**, *139*, 9519-9522.

(S4) Hajime, I.; Kubota, K. *Org. Lett.* **2012**, *14*, 890-893.

(S5) Zhou, X.-F.; Wu, Y.-D.; Dai, J.-J.; Li, Y.-J.; Huand, Y.; Xu, J. *RSC Adv.* **2015**, *5*, 46672-46676.

(S6) Fawcett, A.; Pradeilles, J.; Wang, Y.; Mutsuga, T.; Myers, E. L.; Aggarwal, V. K. *Science* **2017**, *357*, 283-286.

(S7) Hintermann, L.; Wong, K. M. *Eur. J. Org. Chem.* **2017**, 5527-5536.

(S8) For the lithiation-borylation procedure see: (a) S. Balieu, G. E. Hallett, M. Burns, T. Bootwicha, J. Studley, V. K. Aggarwal, *J. Am. Chem. Soc.* **2015**, *137*, 4398–4403. For the synthesis of the enantioenriched stannane see: (b) M. Burns, S. Essafi, J. R. Bame, S. P. Bull, M. P. Webster, S. Balieu, J. W. Dale, C. P. Butts, J. N. Harvey, V. K. Aggarwal, *Nature* **2014**, *513*, 183–188. For the synthesis of the starting primary boronic ester see: (c) G. Casoni, E. L. Myers, V. K. Aggarwal, *Synthesis* **2016**, *48*, 3241–3253. For analytical data see: (d) R. Larouche-Gauthier, C. J. Fletcher, I. Couto, V. K. Aggarwal, *Chem. Commun.* **2011**, *47*, 12592–12594.

(S9) Bruker, *SAINT+ v8.38A Integration Engine, Data Reduction Software, Bruker Analytical X-ray Instruments Inc., Madison, WI, USA*, 2015.

(S10) Bruker, *SADABS 2014/5, Bruker AXS area detector scaling and absorption correction, Bruker Analytical X-ray Instruments Inc., Madison, Wisconsin, USA*, 2014/5.

(S11) G. M. Sheldrick, *Acta Crystallographica a-Foundation and Advances*, 2015, **71**, 3-8.

(S12) G. M. Sheldrick, *Acta Crystallogr., Sect. A: Found. Crystallogr.*, 2008, **64**, 112-122.

(S13) G. M. Sheldrick, *Acta Crystallogr. C*, 2015, **71**, 3-8.

(S14) O. V. Dolomanov, L. J. Bourhis, R. J. Gildea, J. A. K. Howard and H. Puschmann, *J. Appl. Crystallogr.*, 2009, **42**, 339-341.

(S15) R. R. Fraser, J.-L. A. Roustan, J. R. Mahajan, *Can. J. Chem.* **1979**, *57,* 2239–2244.

(S16) H. Shanan-Atidi, K. H. Bar-Eli, *J. Phys. Chem.* **1970**, *74*, 961–963.

(S17) D. Imao, B. W. Glasspoole, V. S. Laberge, C. M. Crudden, *J. Am. Chem. Soc.* **2009**, *131*, 5024-5025.

13 NMR spectra

Figure S13: ^{1}H NMR of precursor to **1a** (400 MHz, $CDCl_3$)

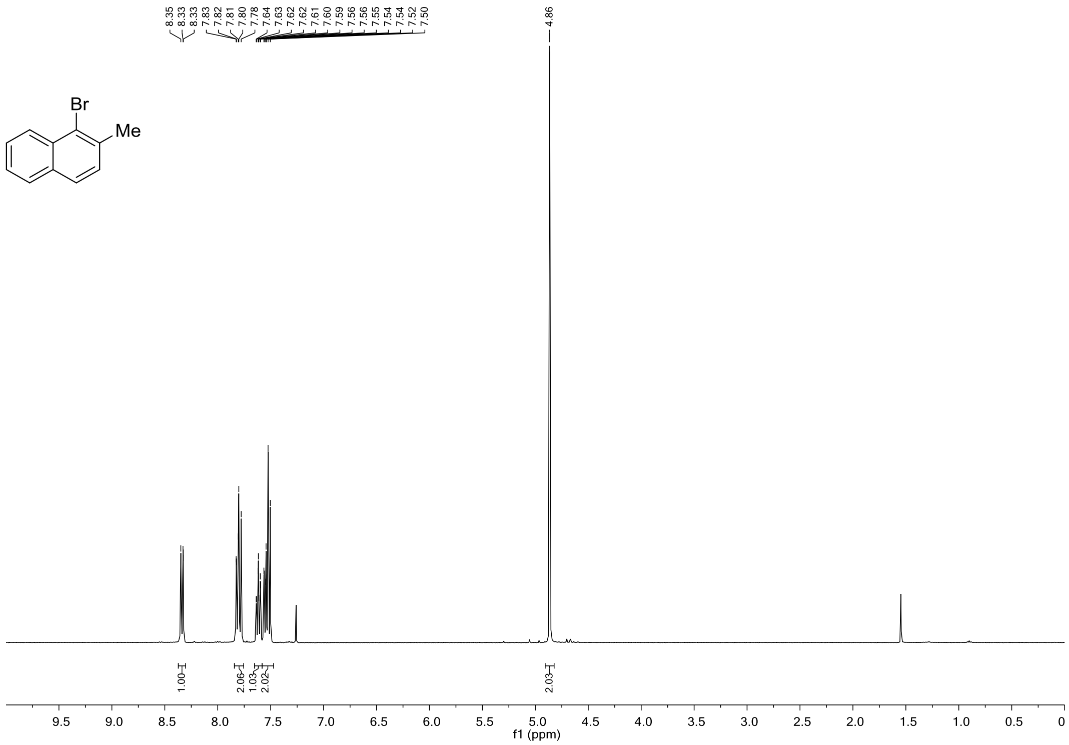

Figure S14: $^{13}C\{^{1}H\}$ NMR of precursor to **1a** (101 MHz, $CDCl_3$)

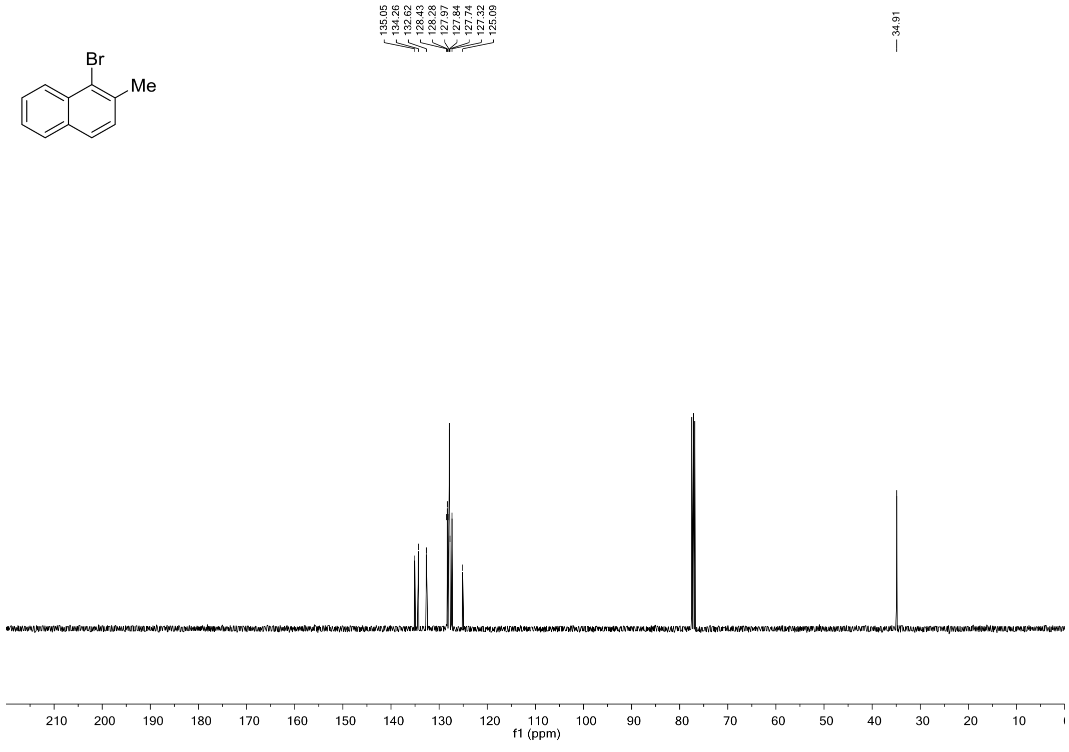

Figure S15: ^{1}H NMR of **1a** (400 MHz, $CDCl_3$)

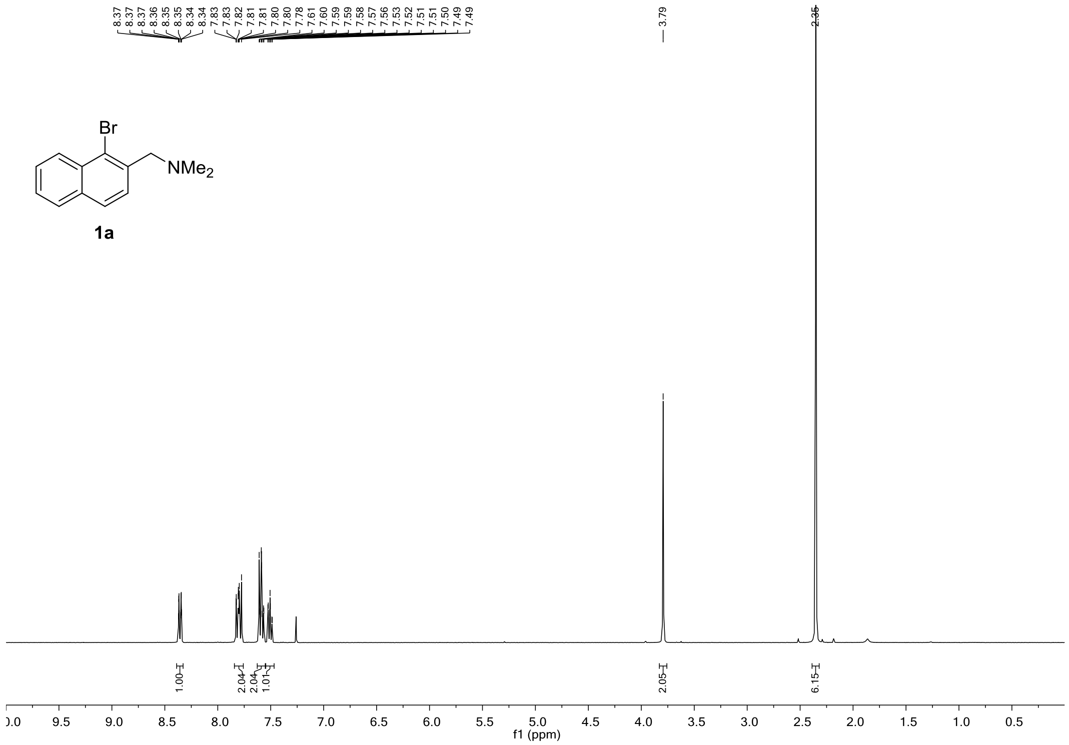

Figure S16: ^{13}C{^{1}H} NMR of **1a** (101 MHz, $CDCl_3$)

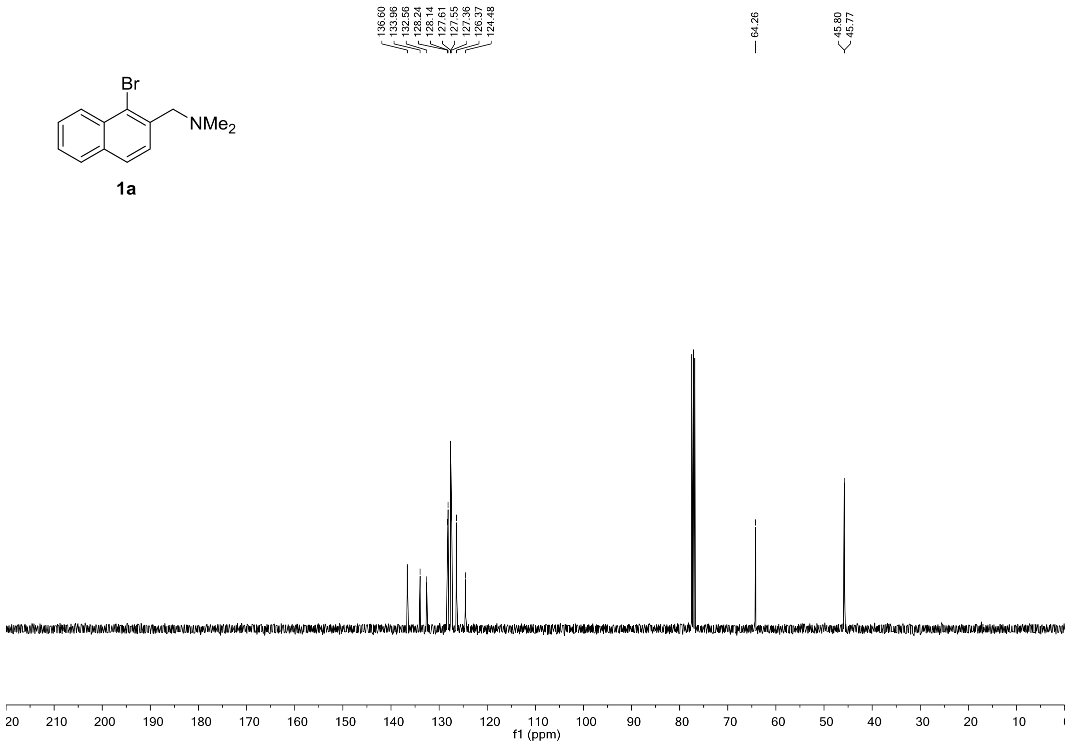

Figure S17: ^{1}H NMR of **1c** (400 MHz, $CDCl_3$)

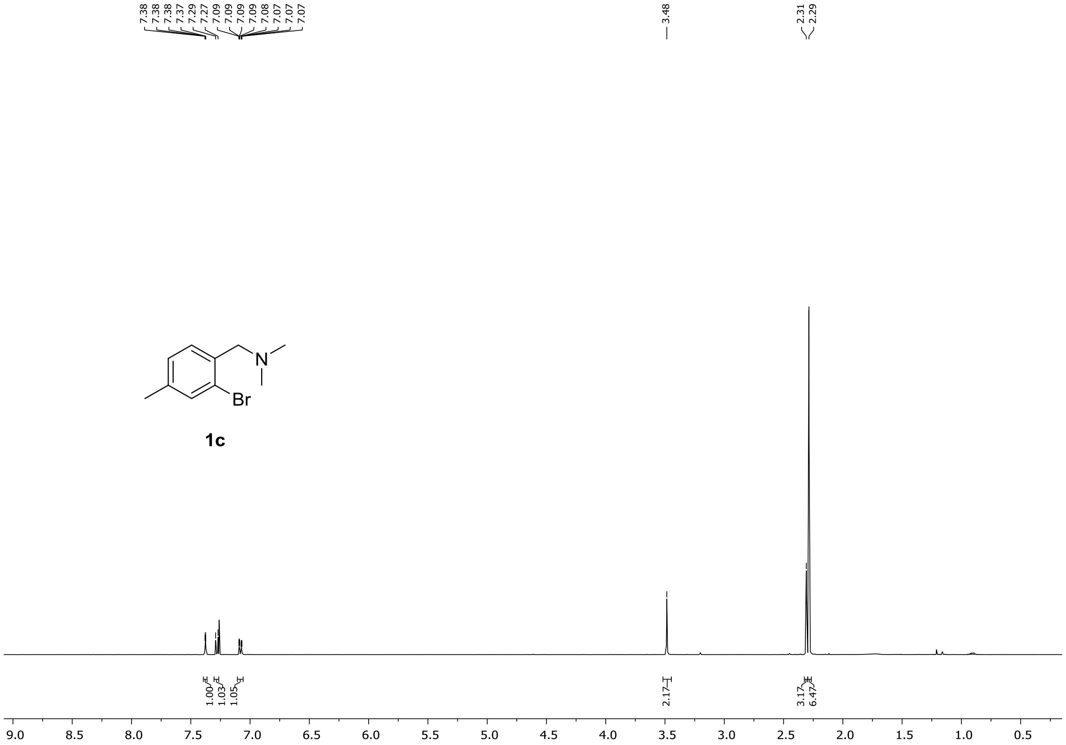

Figure S18: $^{13}C\{^{1}H\}$ NMR of **1c** (101 MHz, $CDCl_3$)

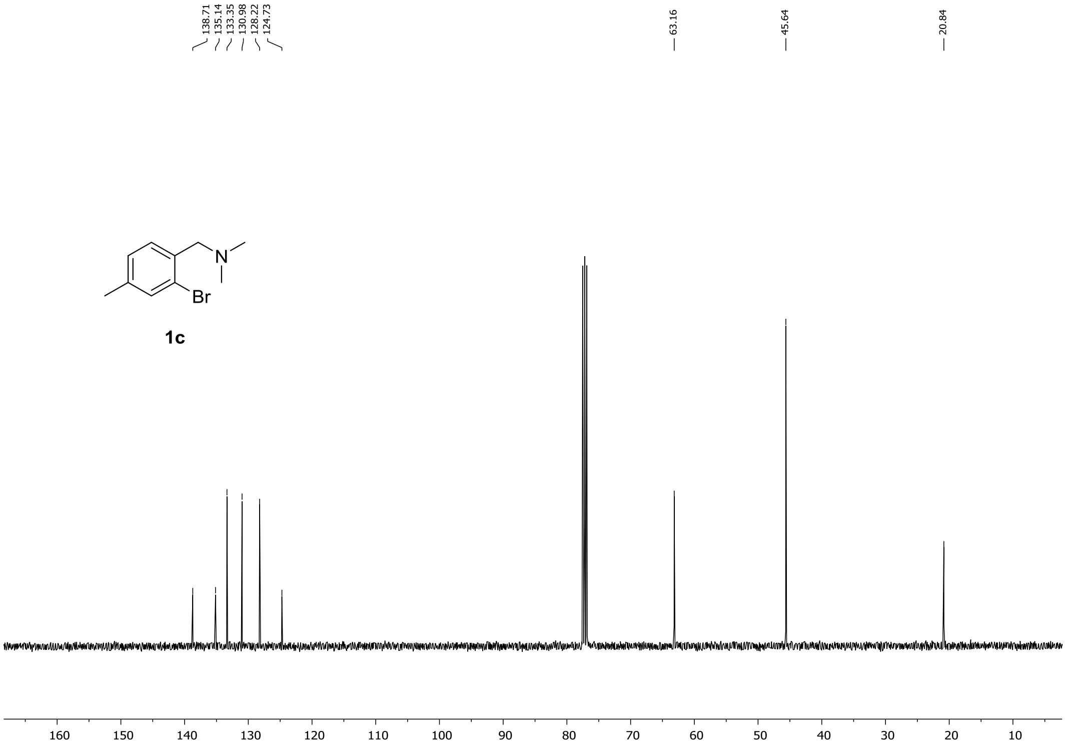

Figure S19: ^{1}H NMR of **2b** (400 MHz, $CDCl_3$)

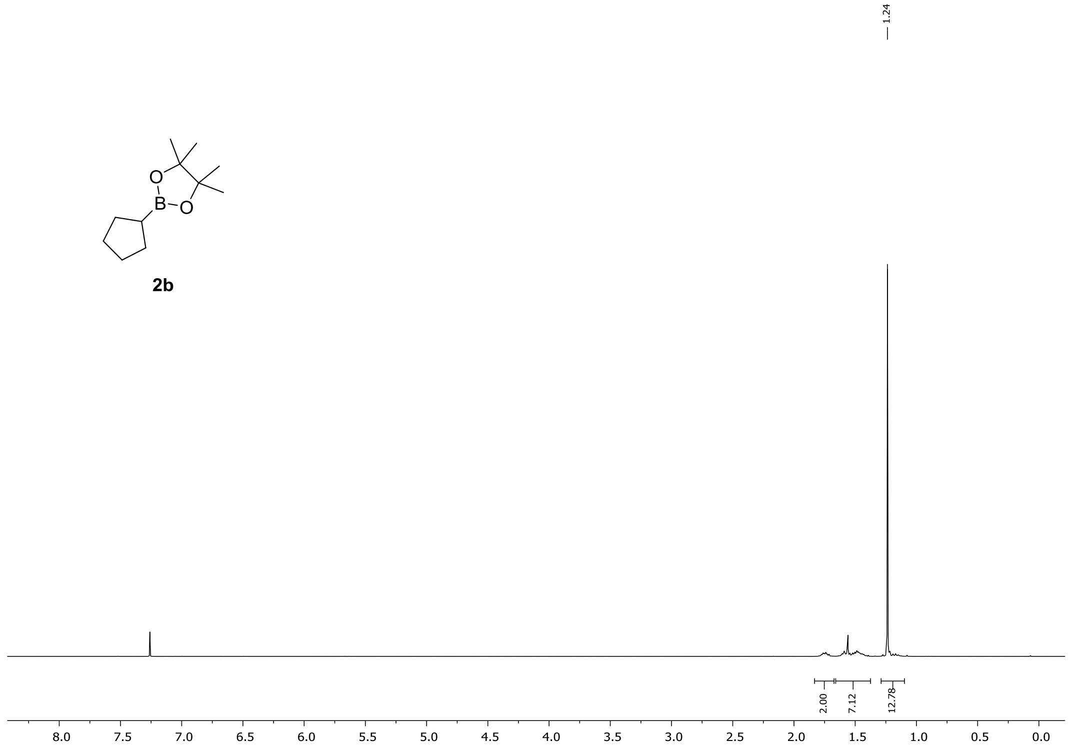

Figure S20: ^{13}C{^{1}H} NMR of **2b** (101 MHz, $CDCl_3$)

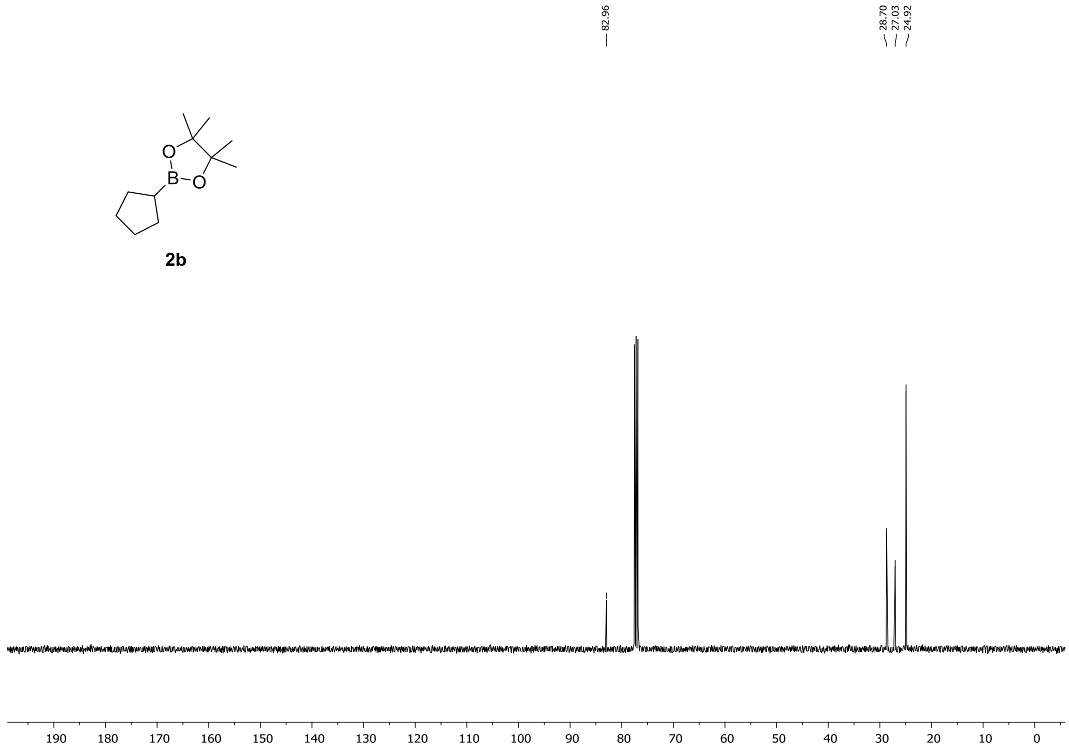

Figure S21: ^{1}H NMR of **2c** (400 MHz, $CDCl_3$)

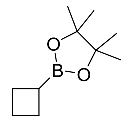

2c

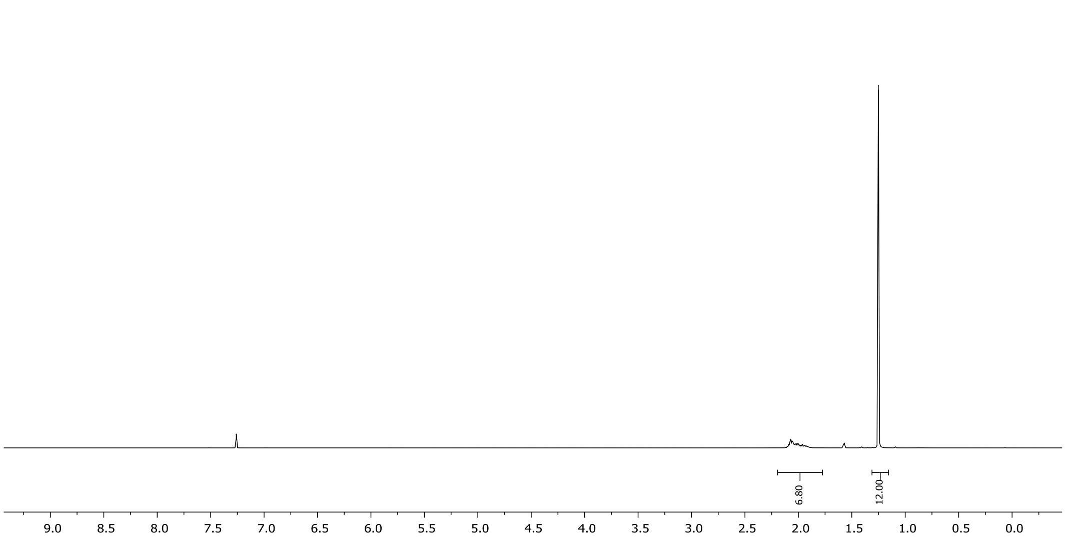

Figure S22: $^{13}C\{^{1}H\}$ NMR of **2c** (101 MHz, $CDCl_3$)

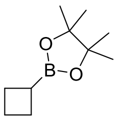

2c

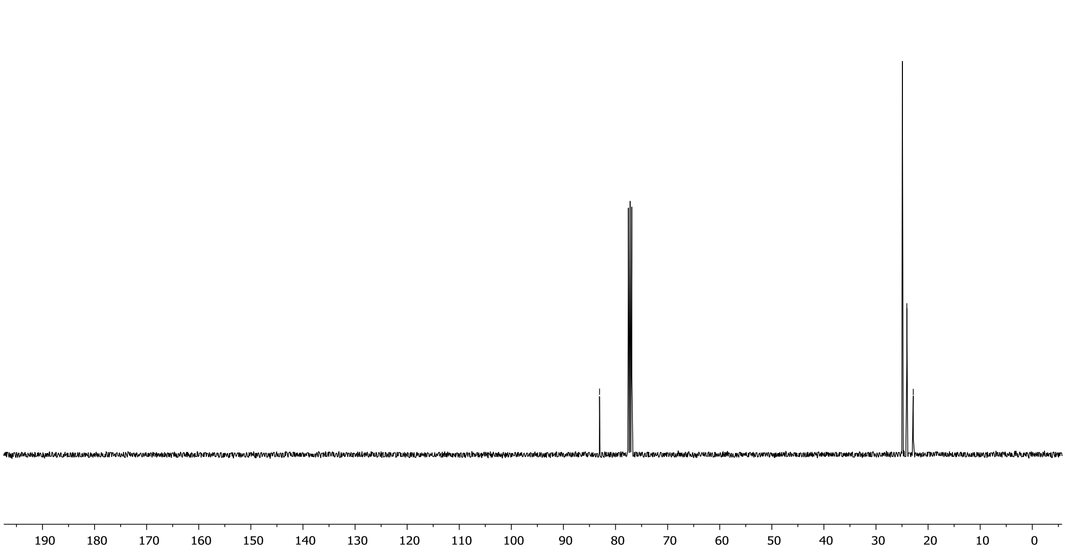

Figure S23: ^{1}H NMR of precursor to **2m** (400 MHz, $CDCl_3$)

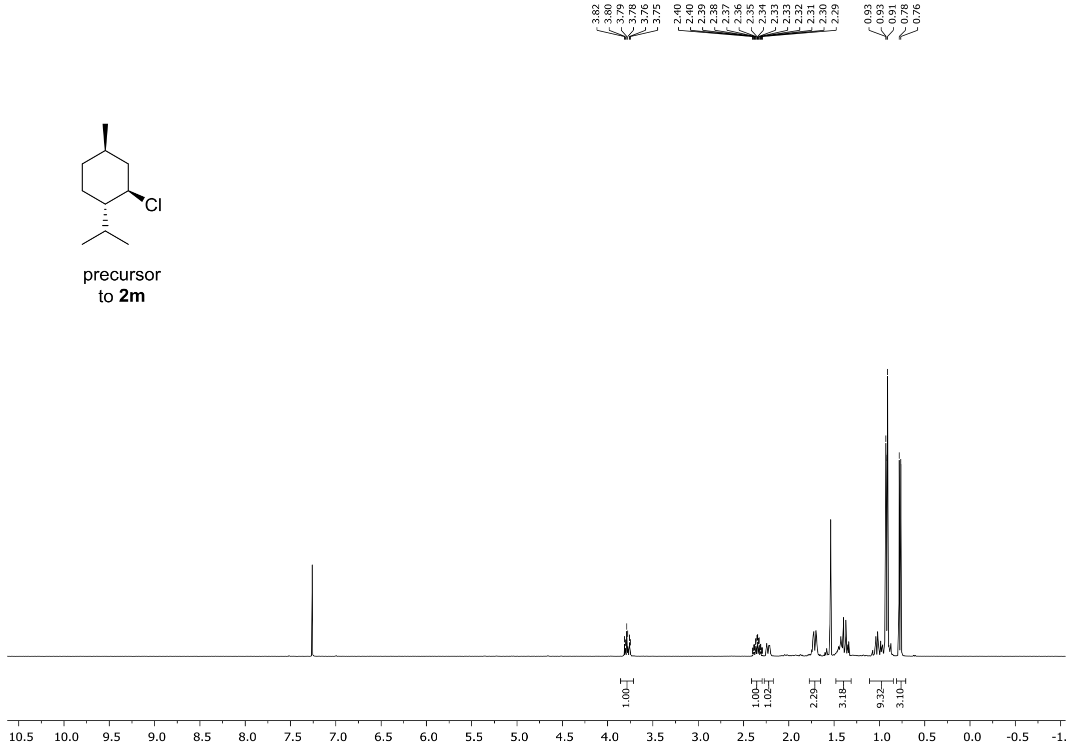

Figure S24: $^{13}C\{^{1}H\}$ NMR of precursor to **2m** (101 MHz, $CDCl_3$)

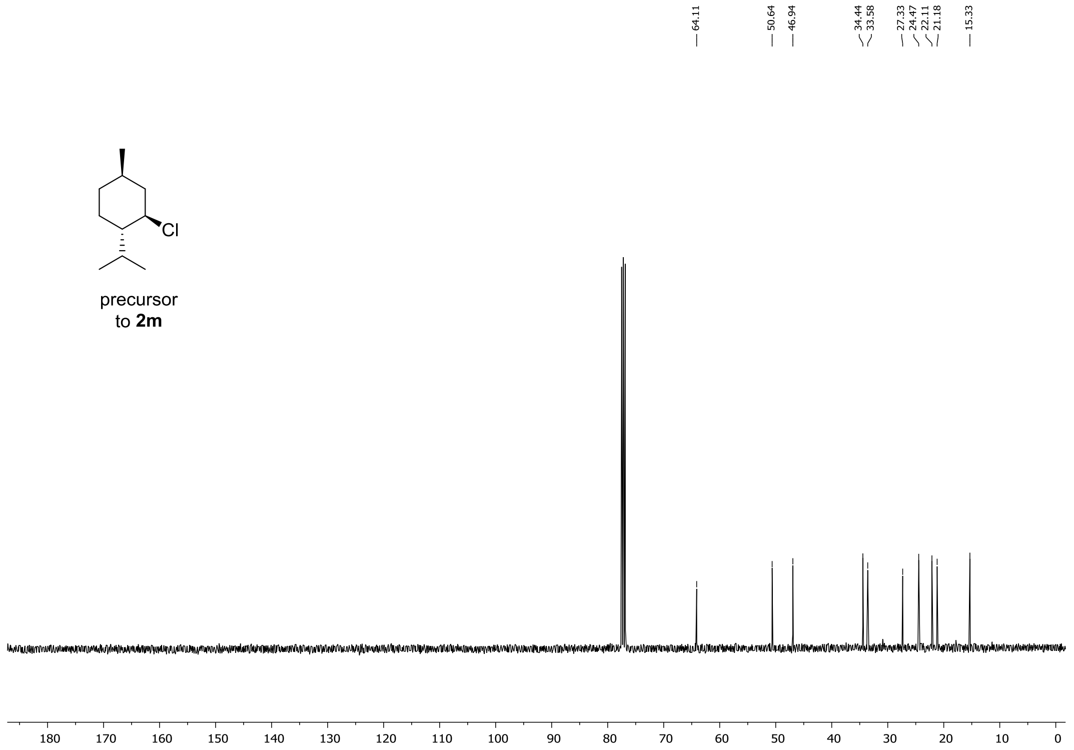

Figure S25: ^{1}H NMR of **2m** (400 MHz, $CDCl_3$)

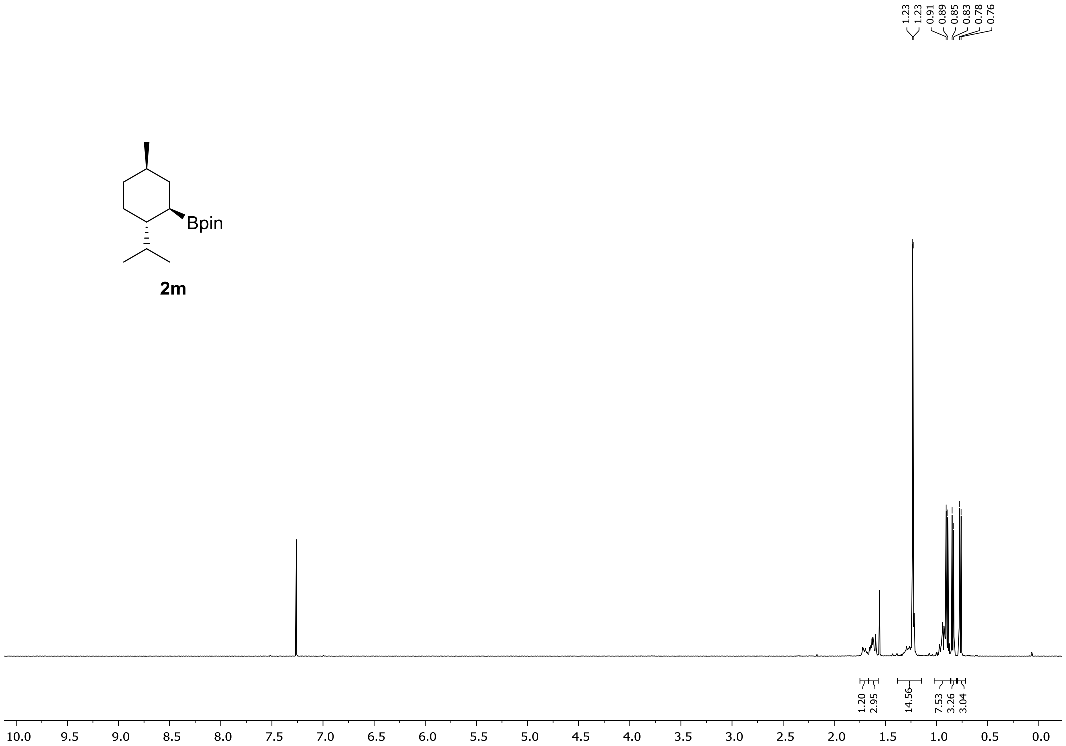

Figure S26: ^{13}C{^{1}H} NMR of **2m** (101 MHz, $CDCl_3$)

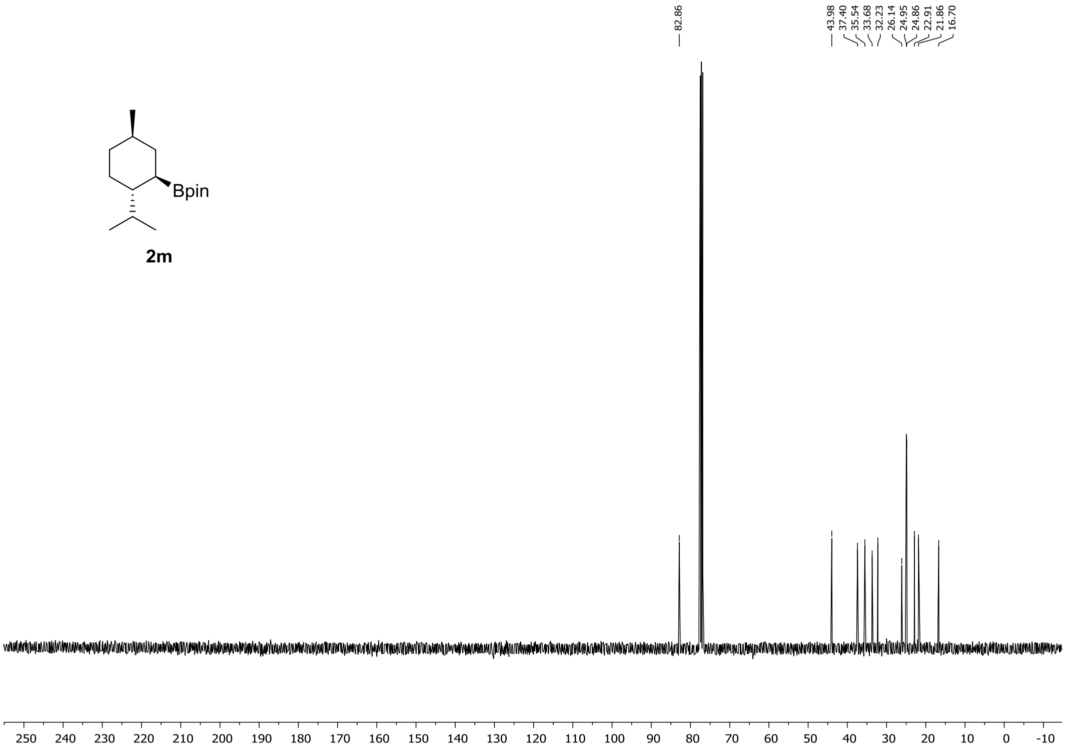

Figure S27: ^{1}H NMR of **4aa** (400 MHz, $CDCl_3$)

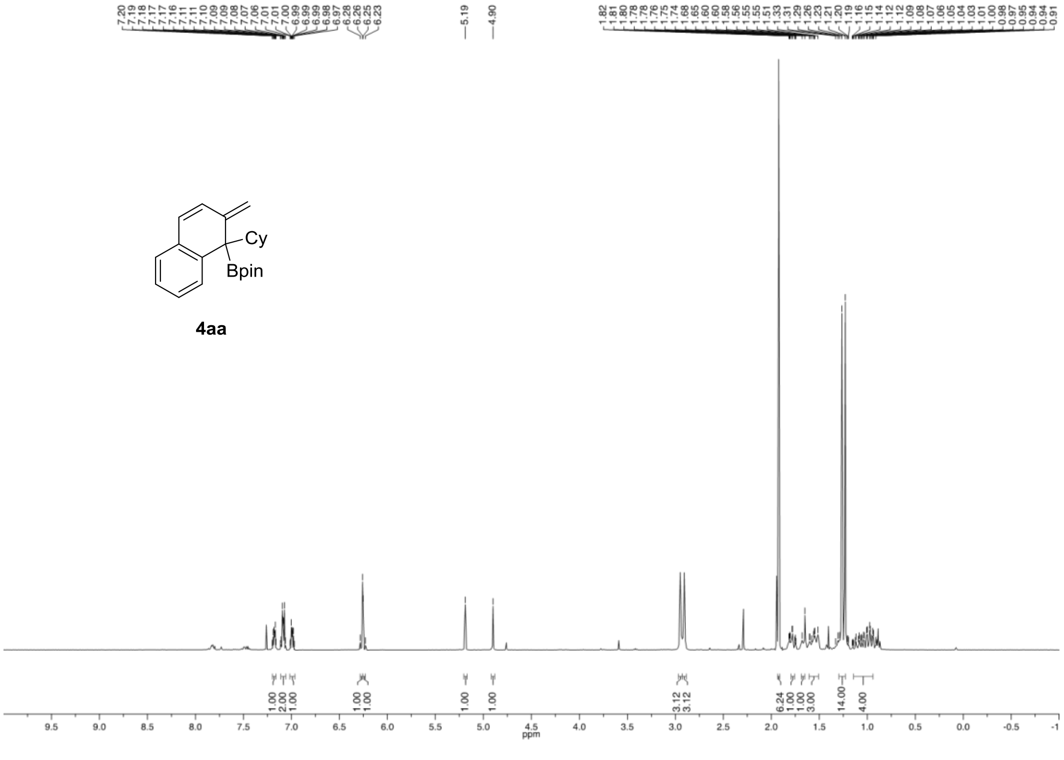

Figure S28: $^{13}C\{^{1}H\}$ NMR of **4aa** (101 MHz, $CDCl_3$)

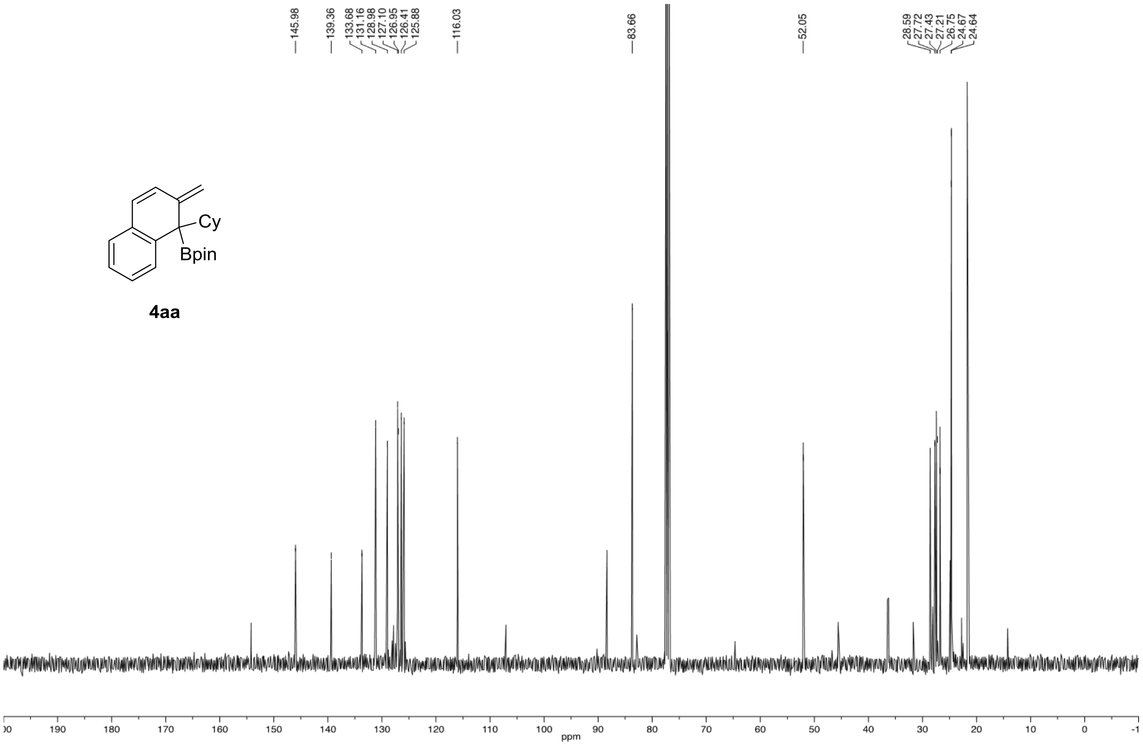

Figure S29: ^{1}H NMR of **6aaa** (400 MHz, $CDCl_3$)

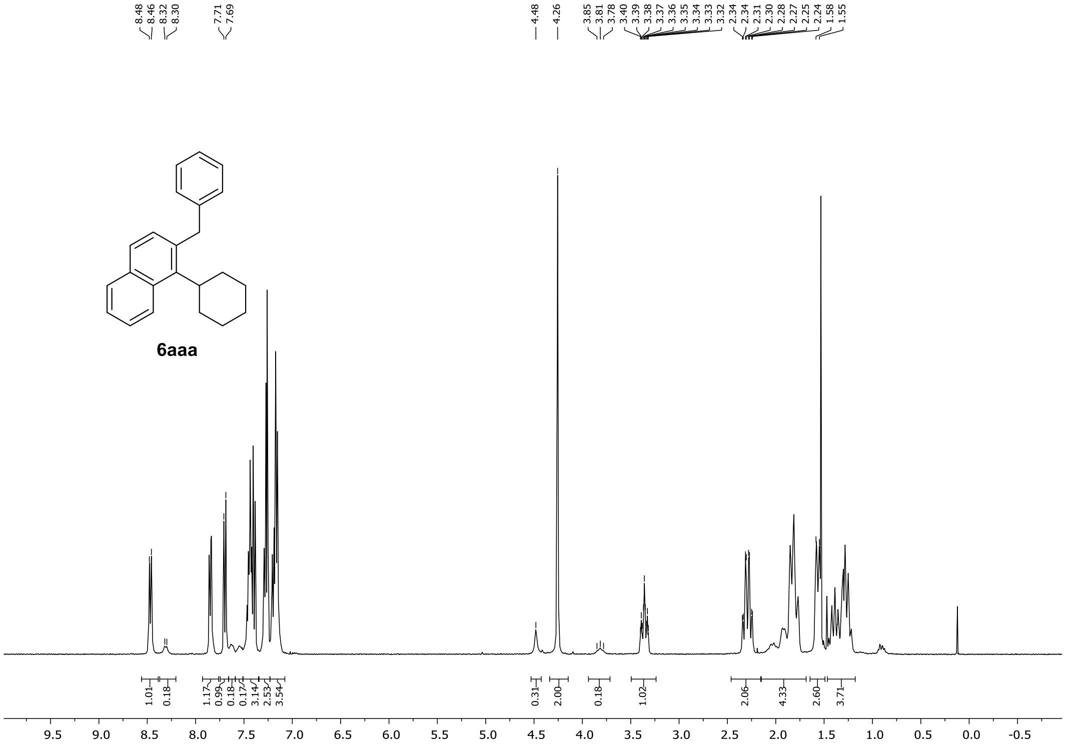

Figure S30: ^{13}C{^{1}H} NMR of **6aaa** (101 MHz, $CDCl_3$)

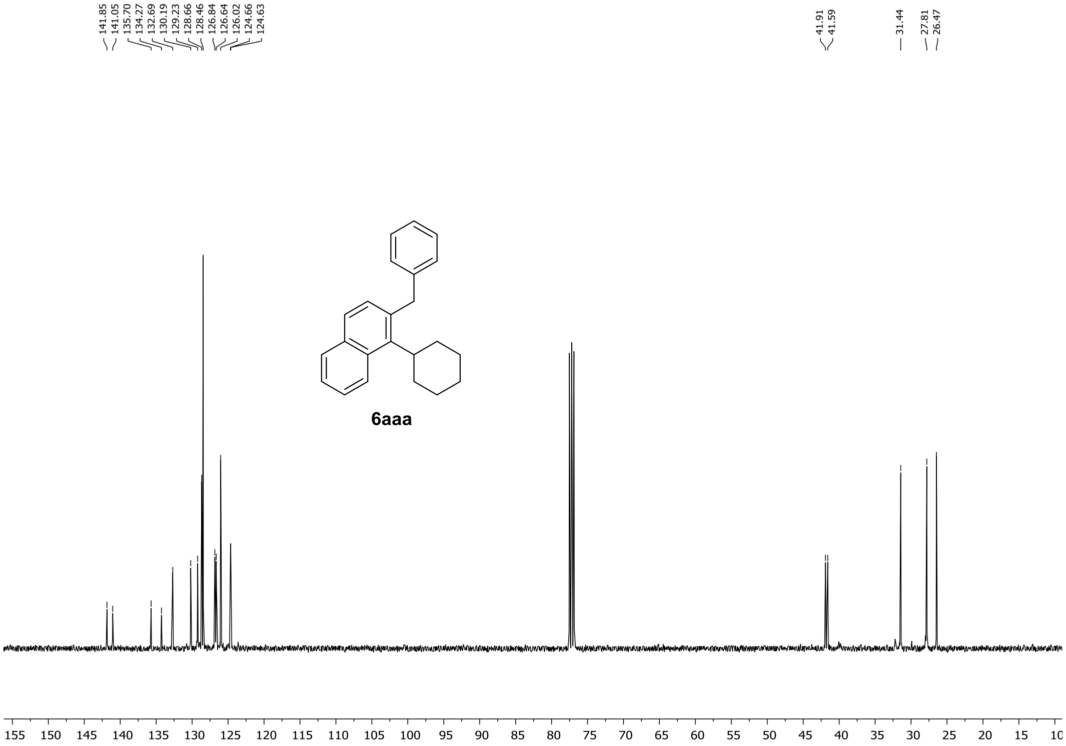

Figure S31: ^{1}H NMR of **6aba** (400 MHz, $CDCl_3$)

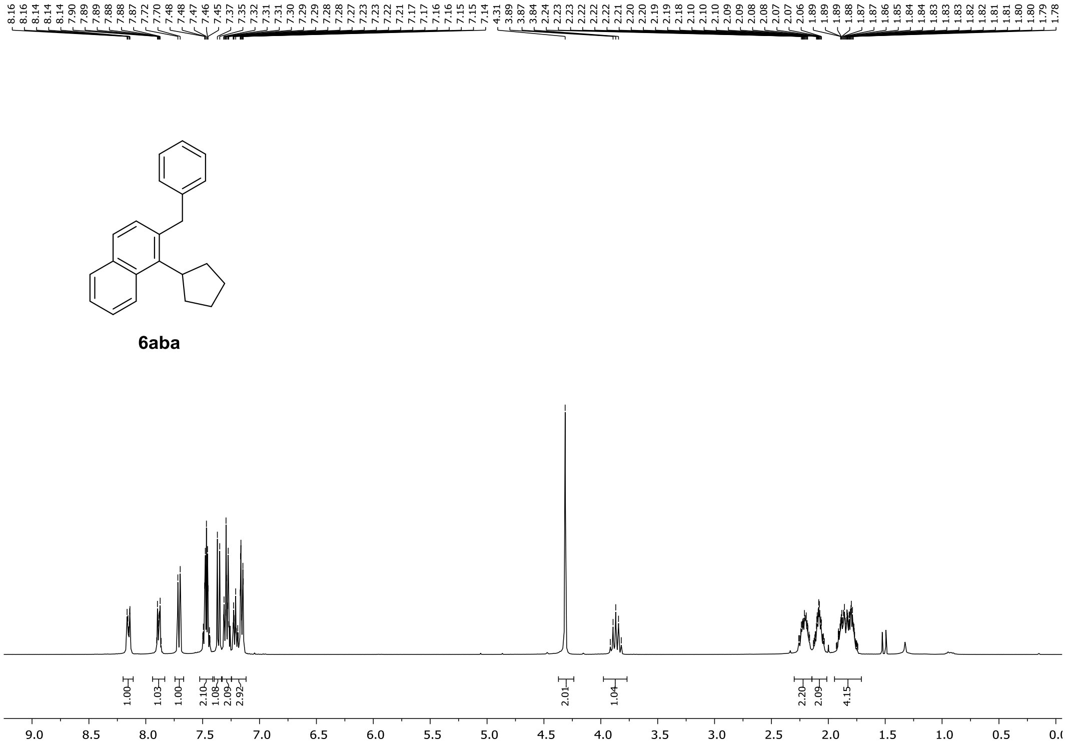

Figure S32: ^{13}C{^{1}H} NMR of **6aba** (101 MHz, $CDCl_3$)

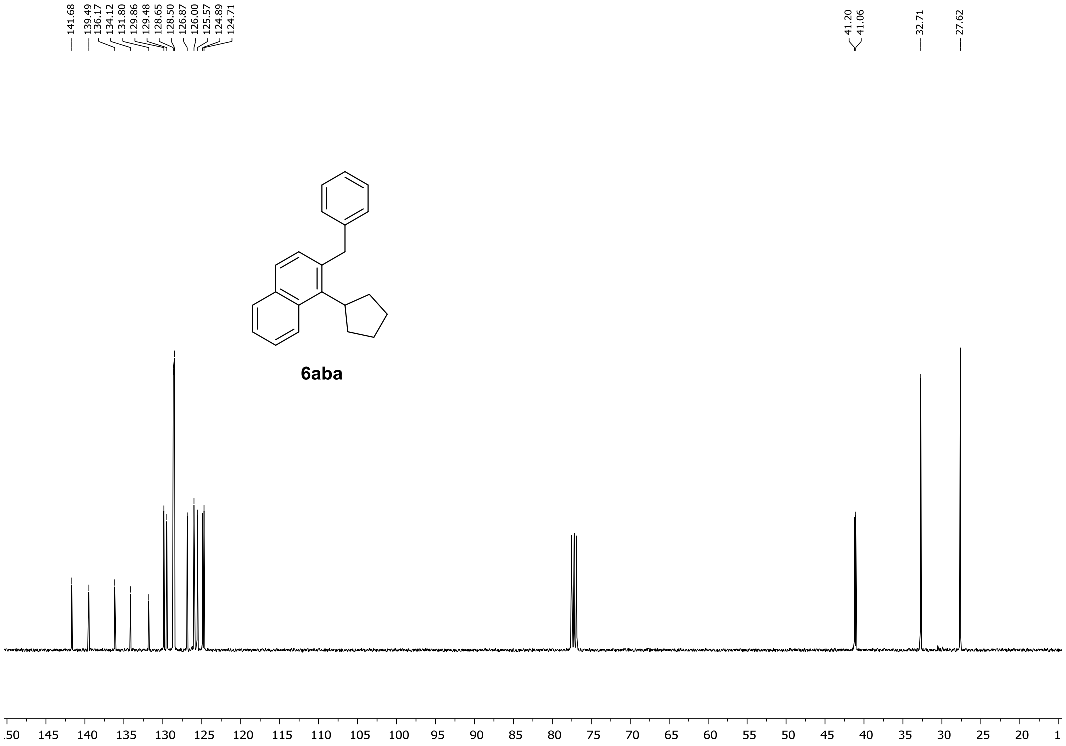

Figure S33: ^{1}H NMR of **6aca** (400 MHz, $CDCl_3$)

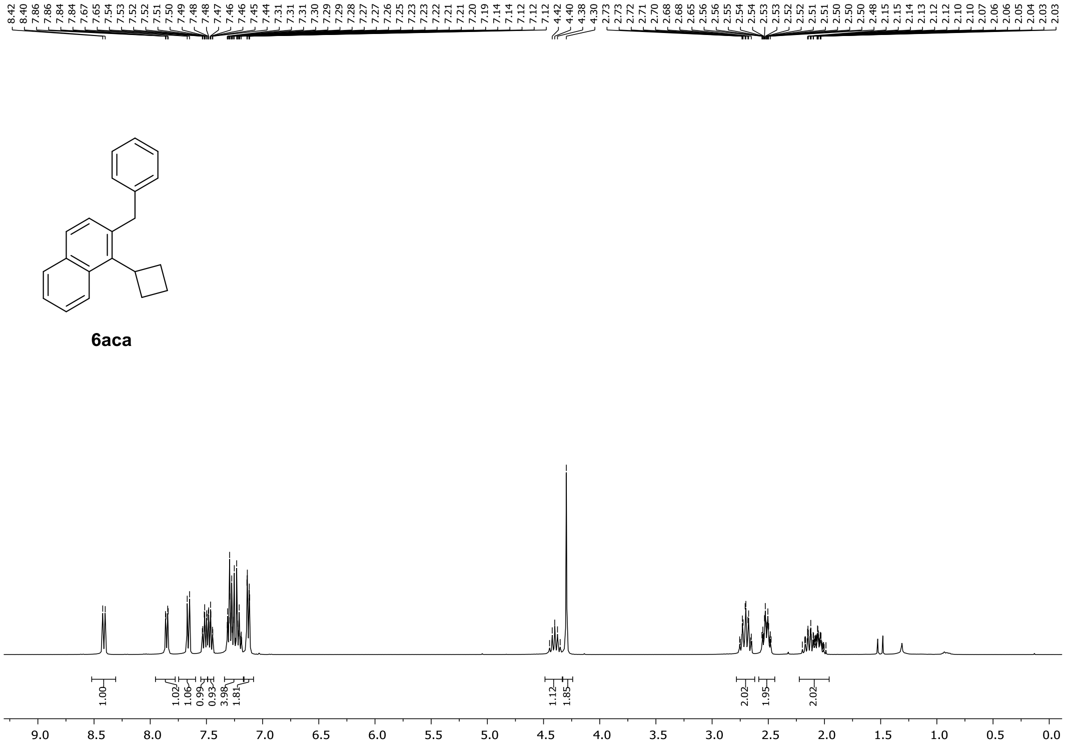

Figure S34: $^{13}C\{^{1}H\}$ NMR of **6aca** (101 MHz, $CDCl_3$)

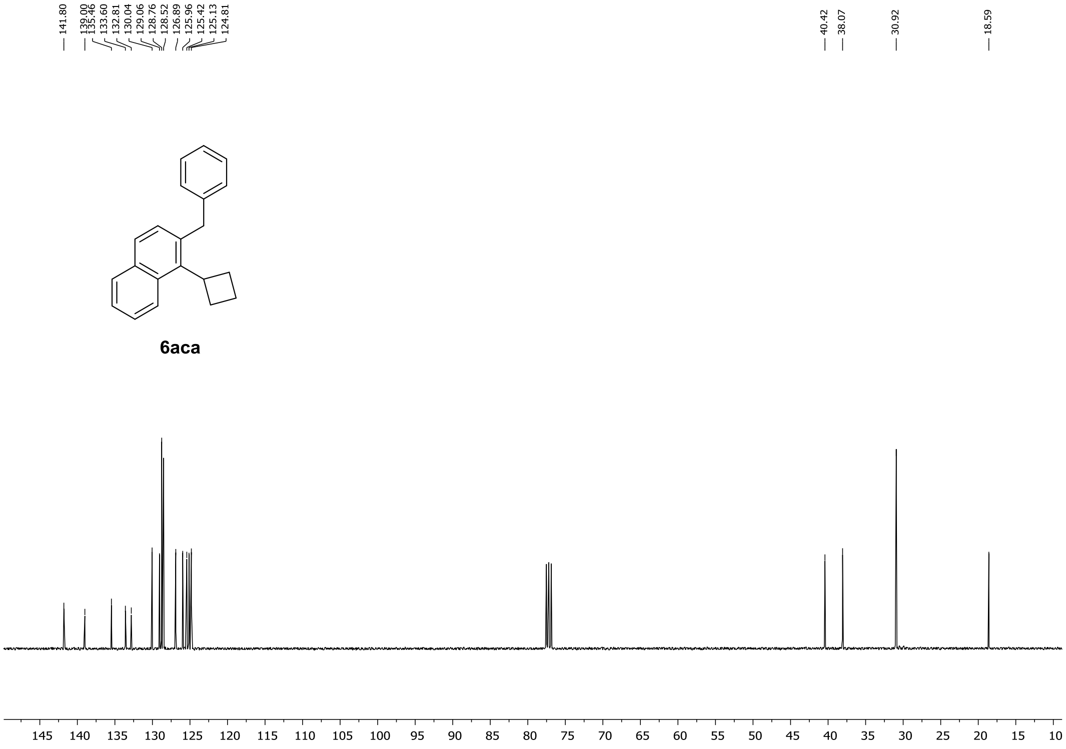

Figure S35: ^{1}H NMR of **6ada** (400 MHz, $CDCl_3$)

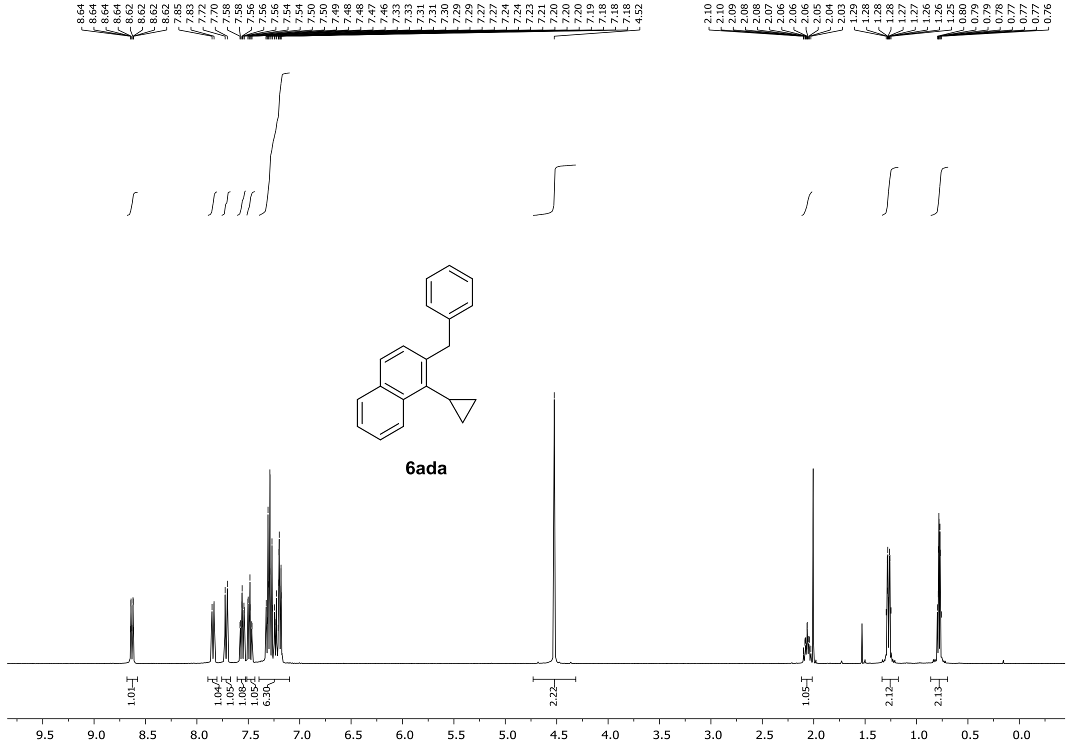

Figure S36: $^{13}C\{^1H\}$ NMR of **6ada** (101 MHz, $CDCl_3$)

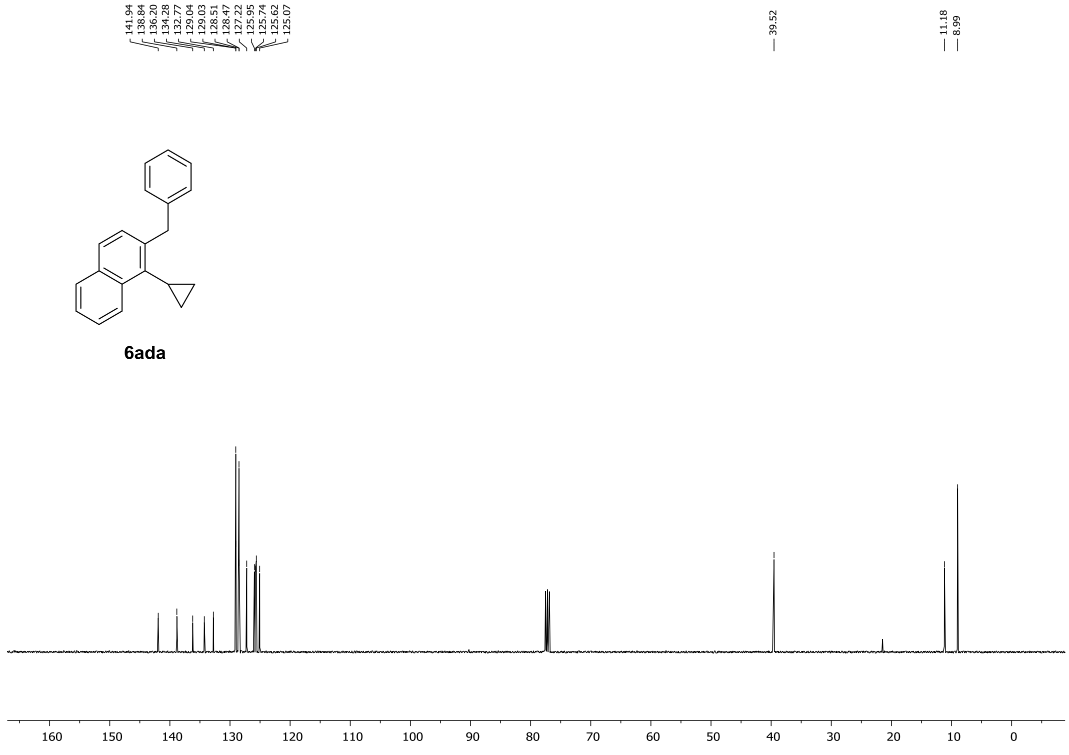

Figure S37: ^{1}H NMR of **6aea** (400 MHz, $CDCl_3$)

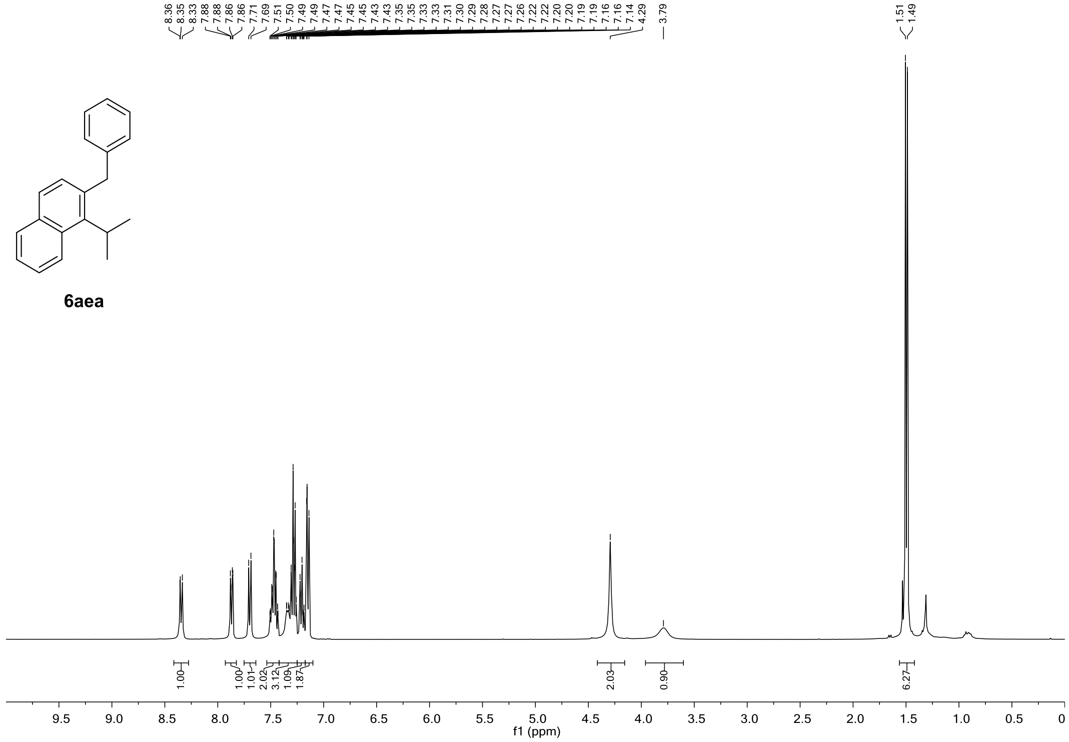

Figure S38: ^{13}C{^{1}H} NMR of **6aea** (101 MHz, $CDCl_3$)

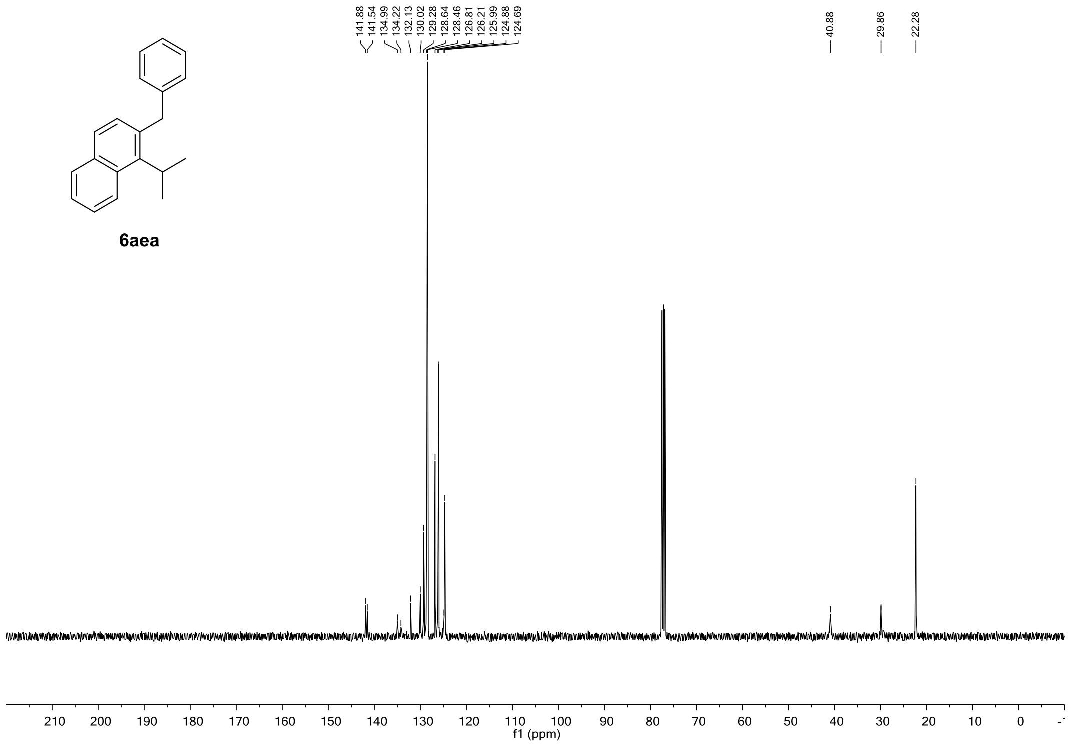

Figure S39: ^{1}H NMR of **6afa** (400 MHz, $CDCl_3$)

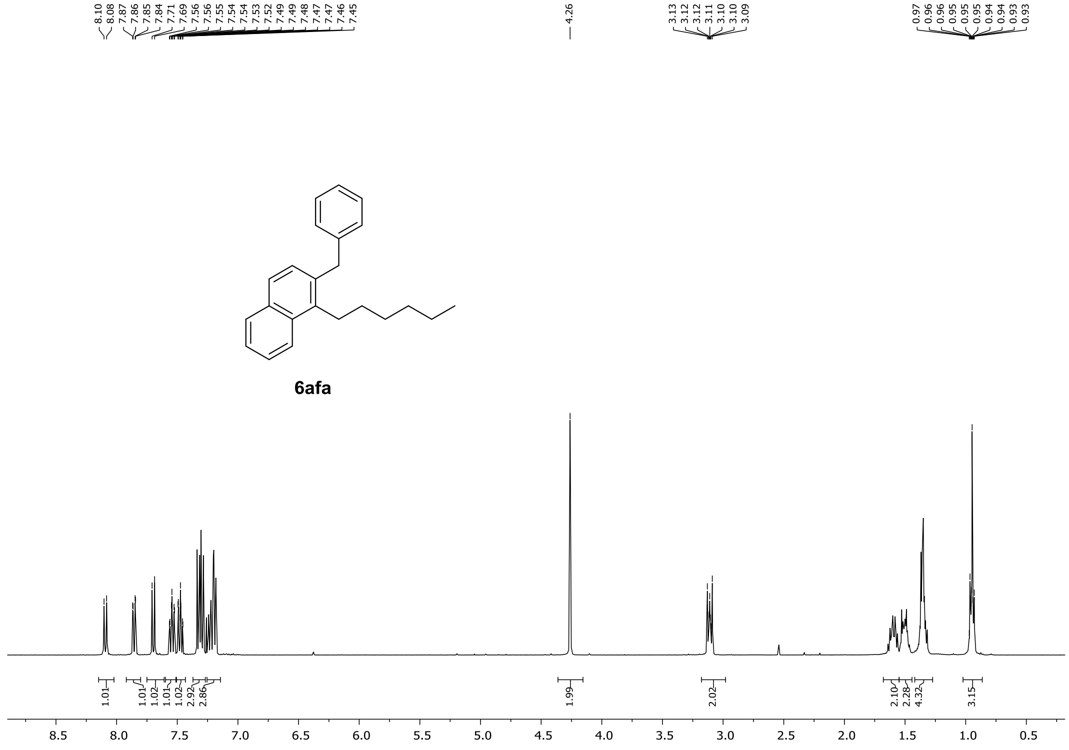

Figure S40: $^{13}C\{^{1}H\}$ NMR of **6afa** (101 MHz, $CDCl_3$)

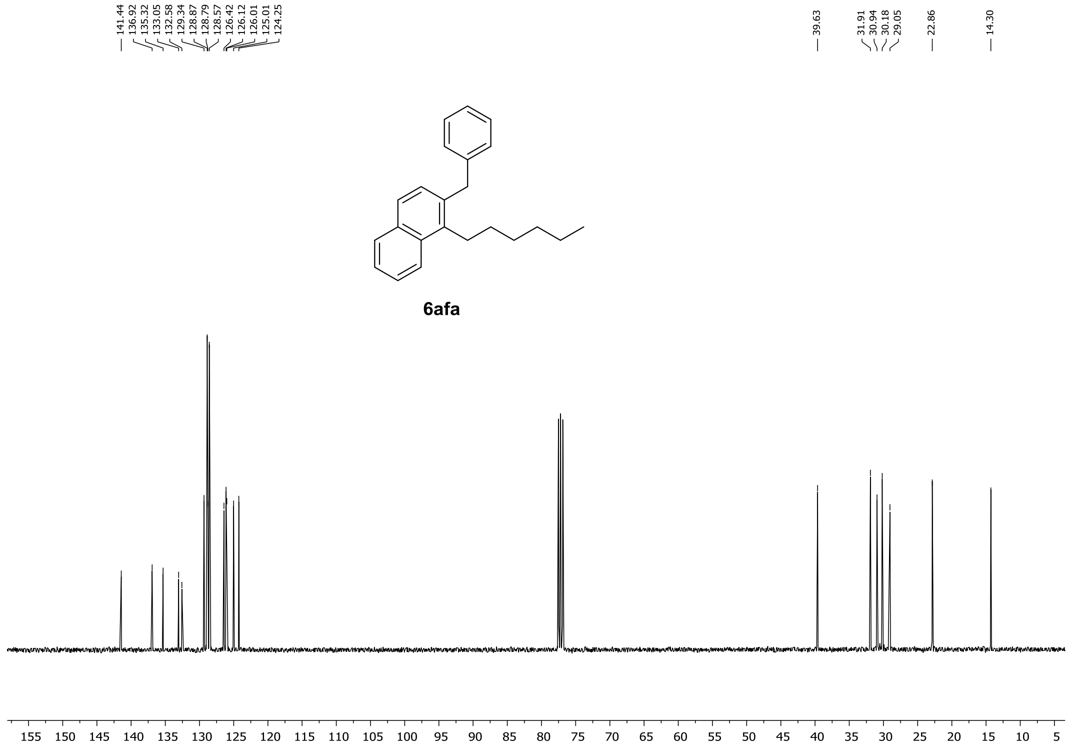

Figure S41: ^{1}H NMR of **S6af** (400 MHz, $CDCl_3$)

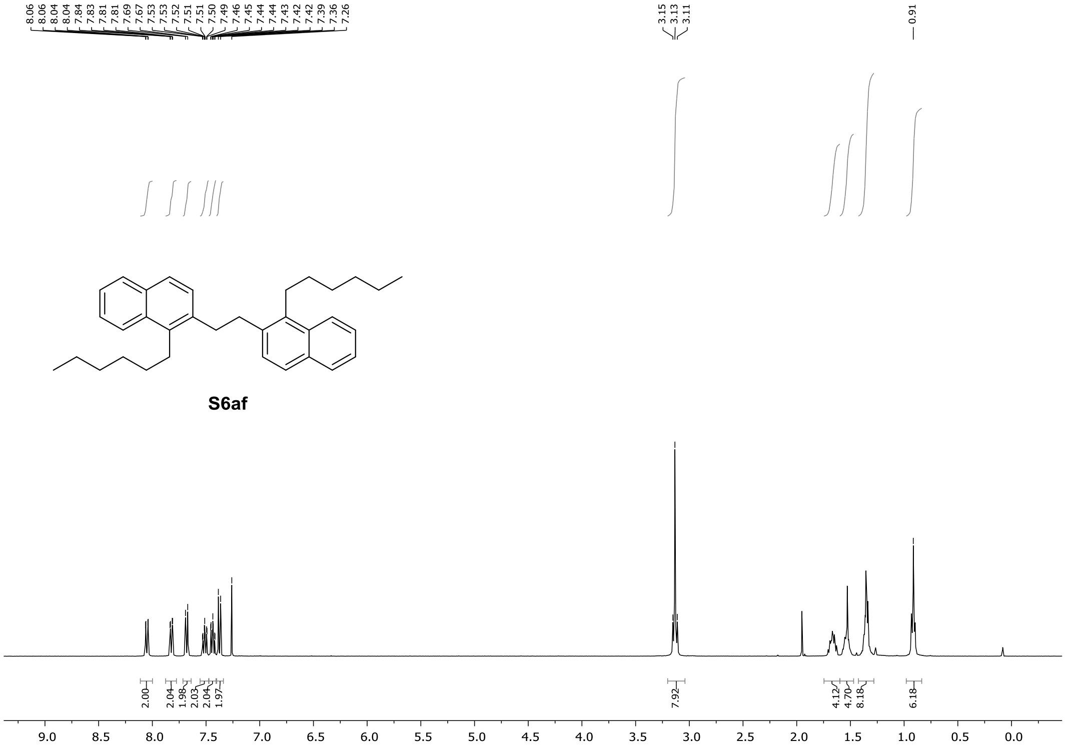

Figure S42: ^{13}C{^{1}H} NMR of **S6af** (101 MHz, $CDCl_3$)

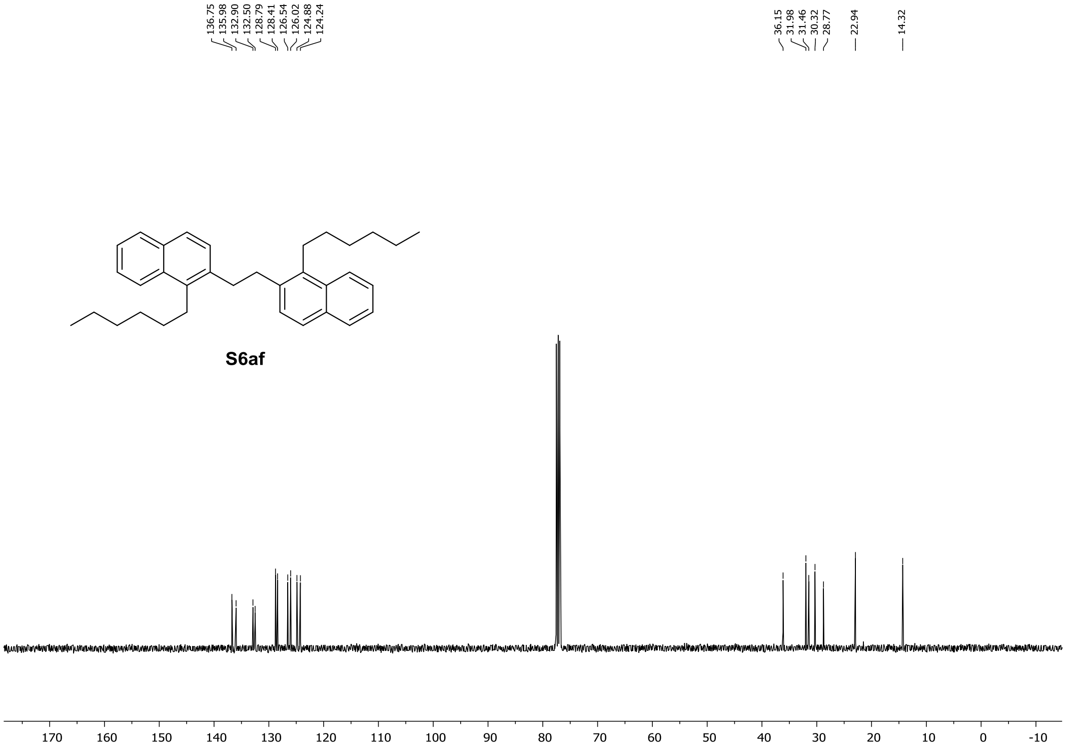

Figure S43: ^{1}H NMR of **6bga** (400 MHz, $CDCl_3$)

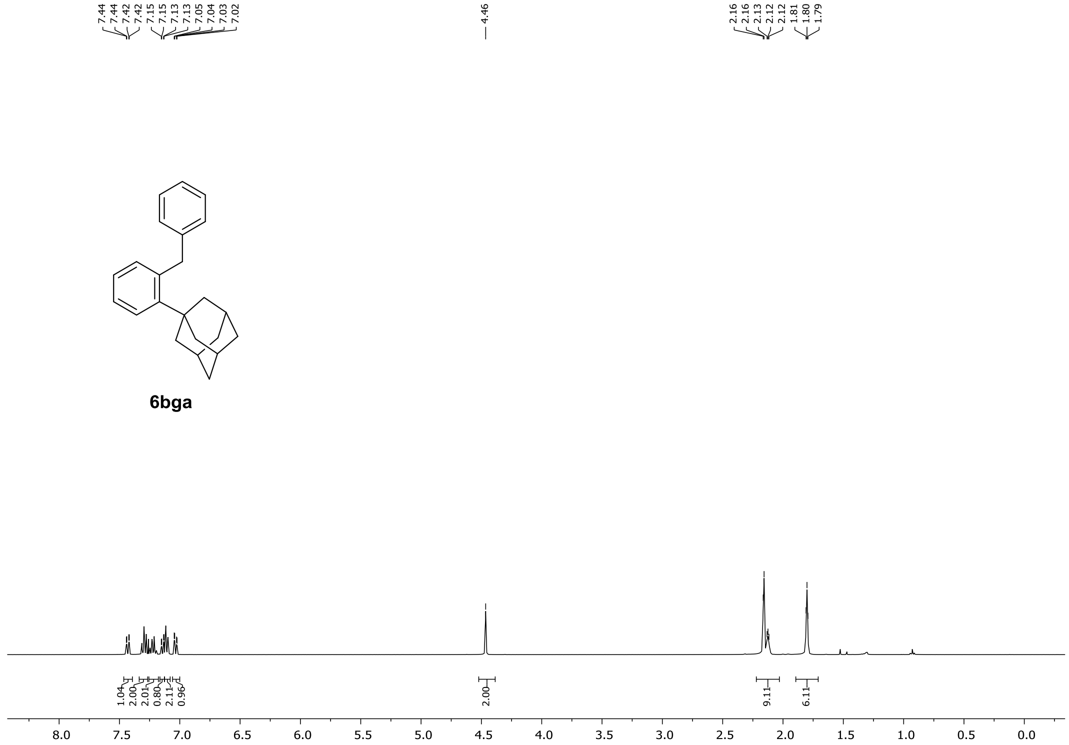

Figure S44: ^{13}C{^{1}H} NMR of **6bga** (101 MHz, $CDCl_3$)

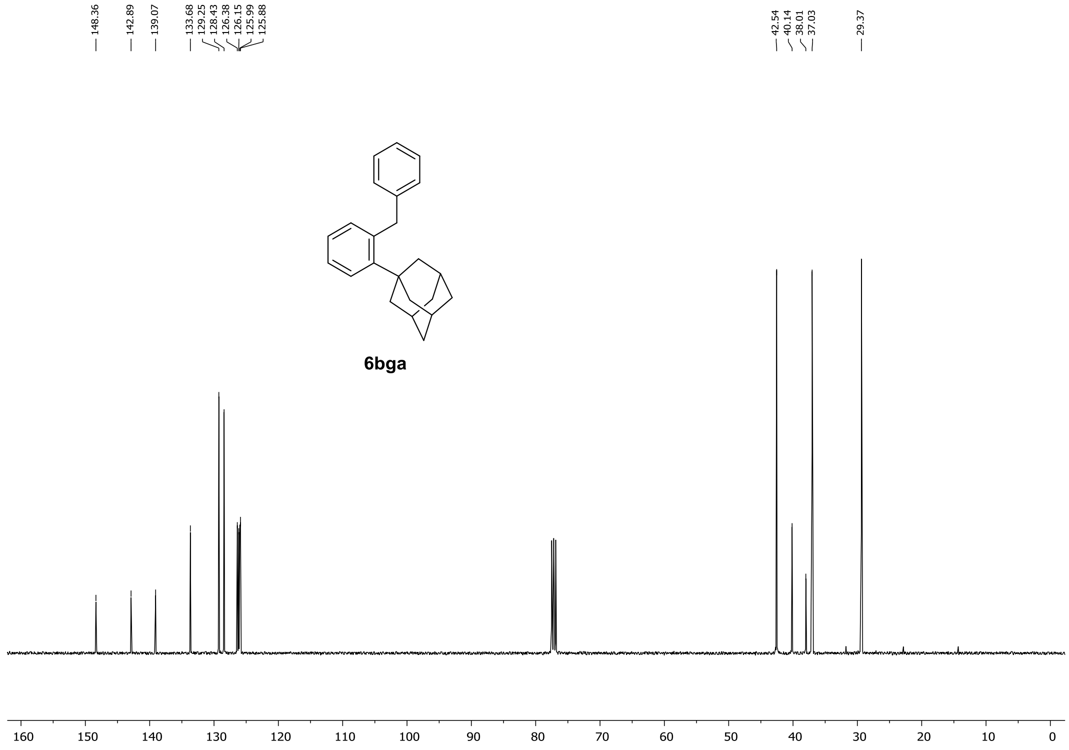

Figure S45: ^{1}H NMR of **6aha** (400 MHz, $CDCl_3$)

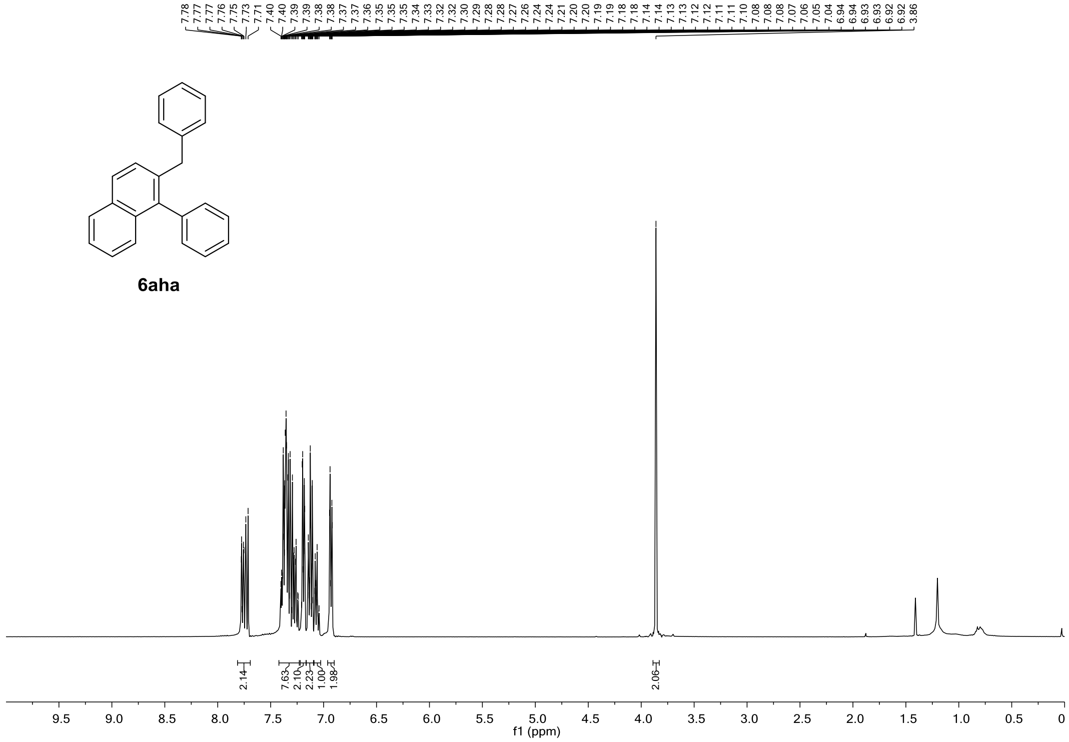

Figure S46: $^{13}C\{^{1}H\}$ NMR of **6aha** (101 MHz, $CDCl_3$)

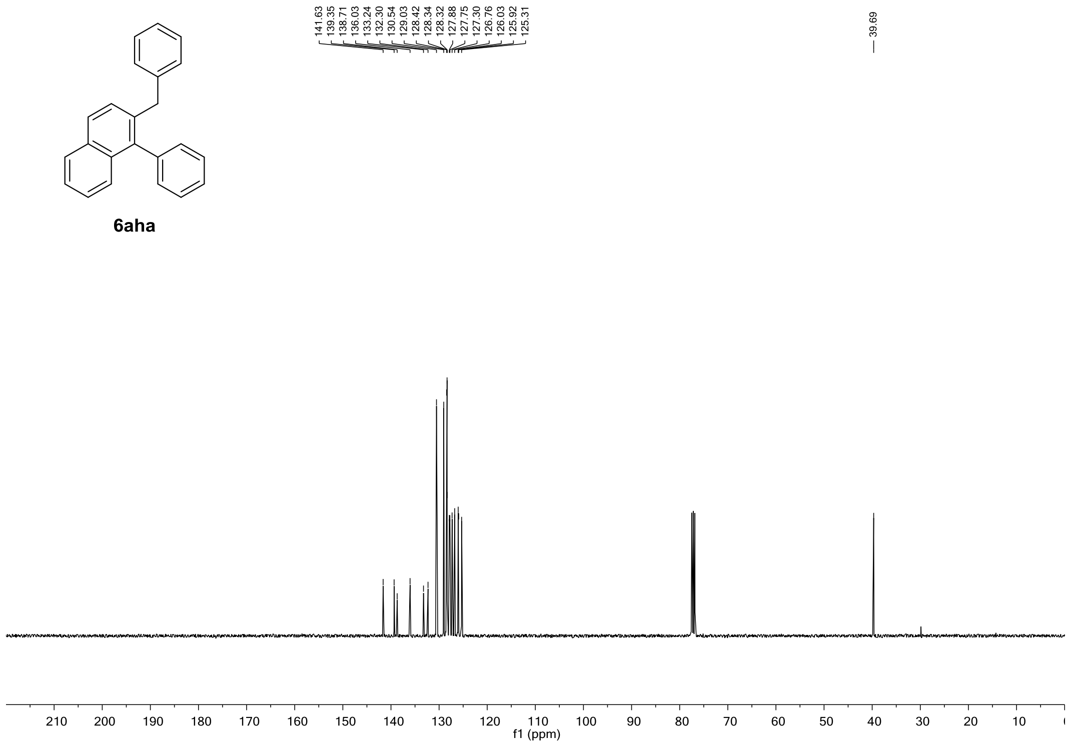

Figure S47: ^{1}H NMR of **(*S*)-6aia** (400 MHz, $CDCl_3$)

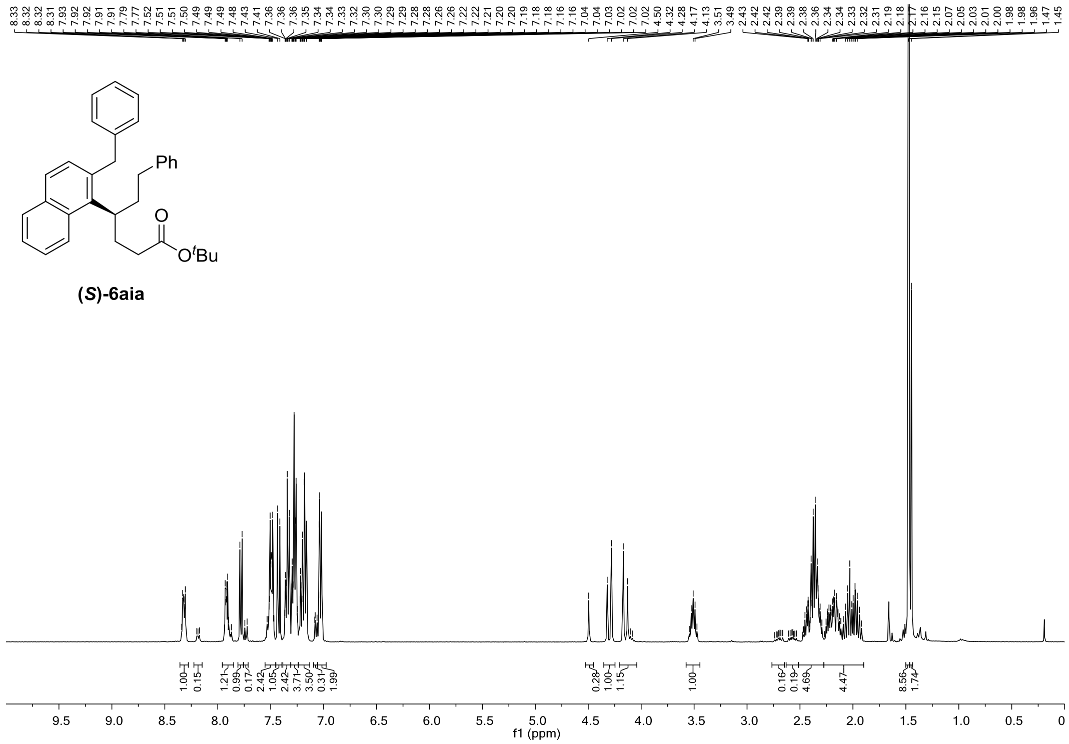

Figure S48: ^{13}C{^{1}H} NMR of **(*S*)-6aia** (101 MHz, $CDCl_3$)

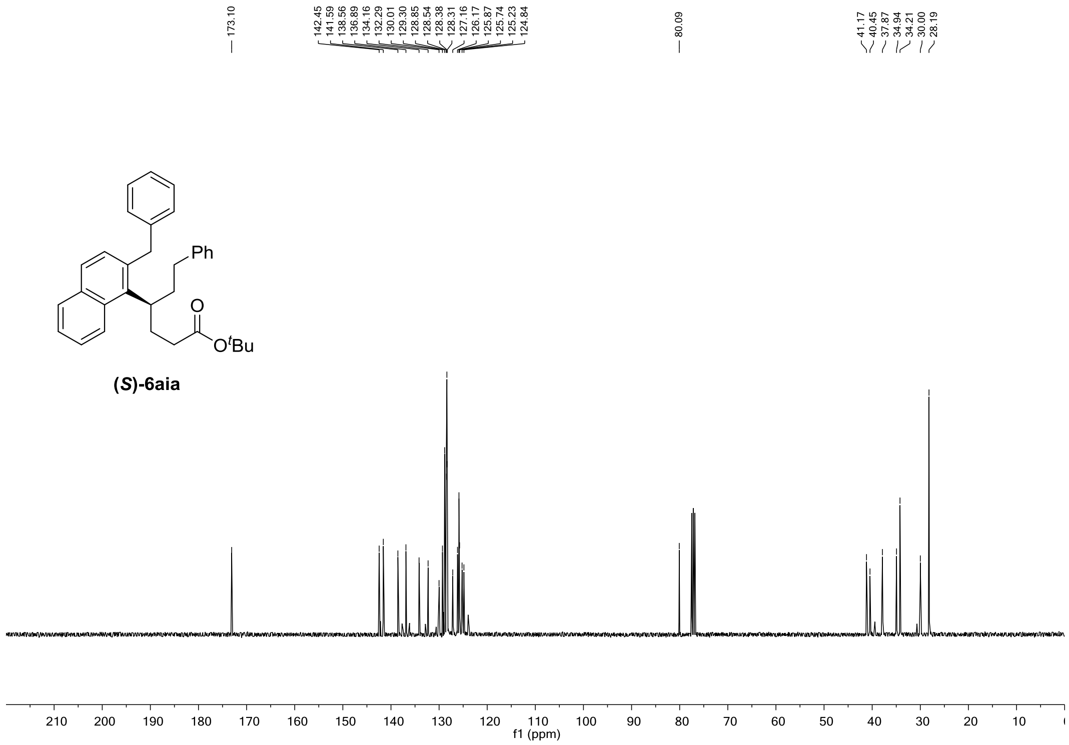

Figure S49: ^{1}H NMR of the crude reaction mixture for product **6bja** (400 MHz, $CDCl_3$).

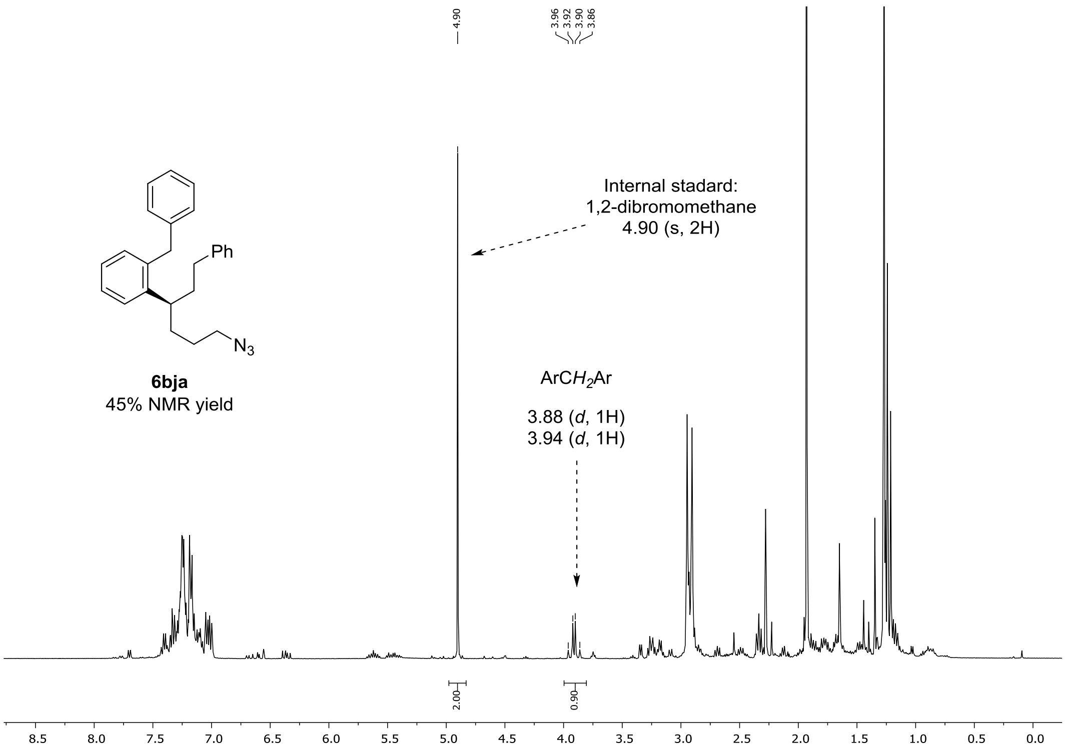

Figure S50: ^{1}H NMR of **6bja** (400 MHz, $CDCl_3$)

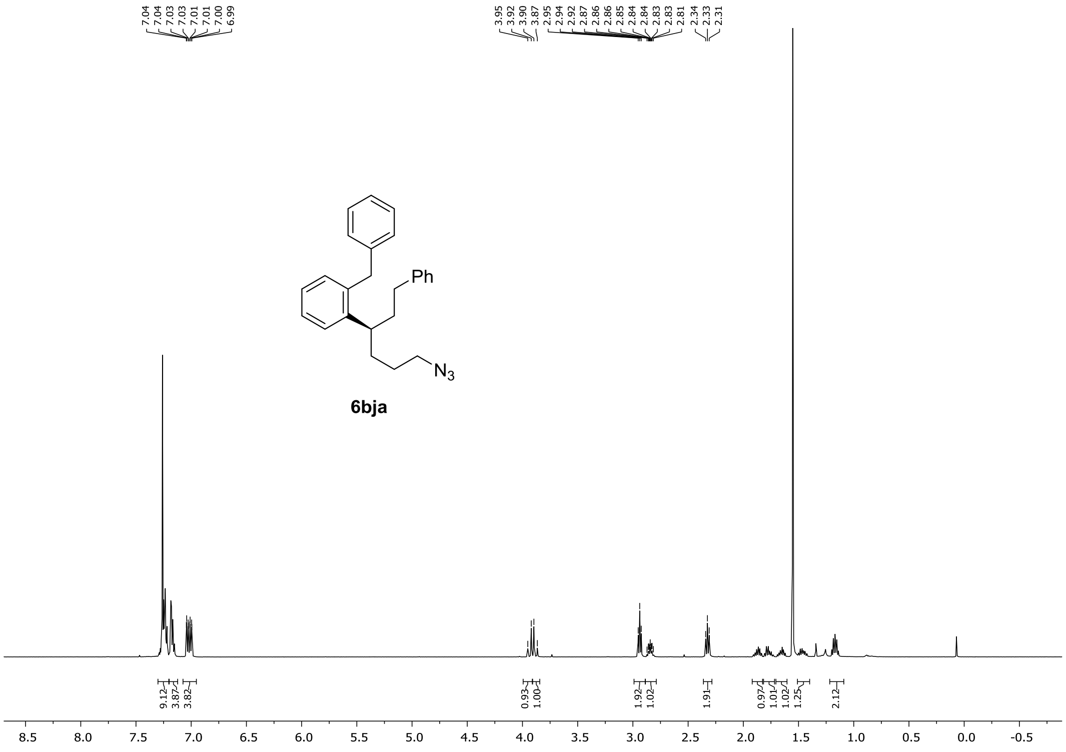

Figure S51: $^{13}C\{^{1}H\}$ NMR of **6bja** (126 MHz, $CDCl_3$)

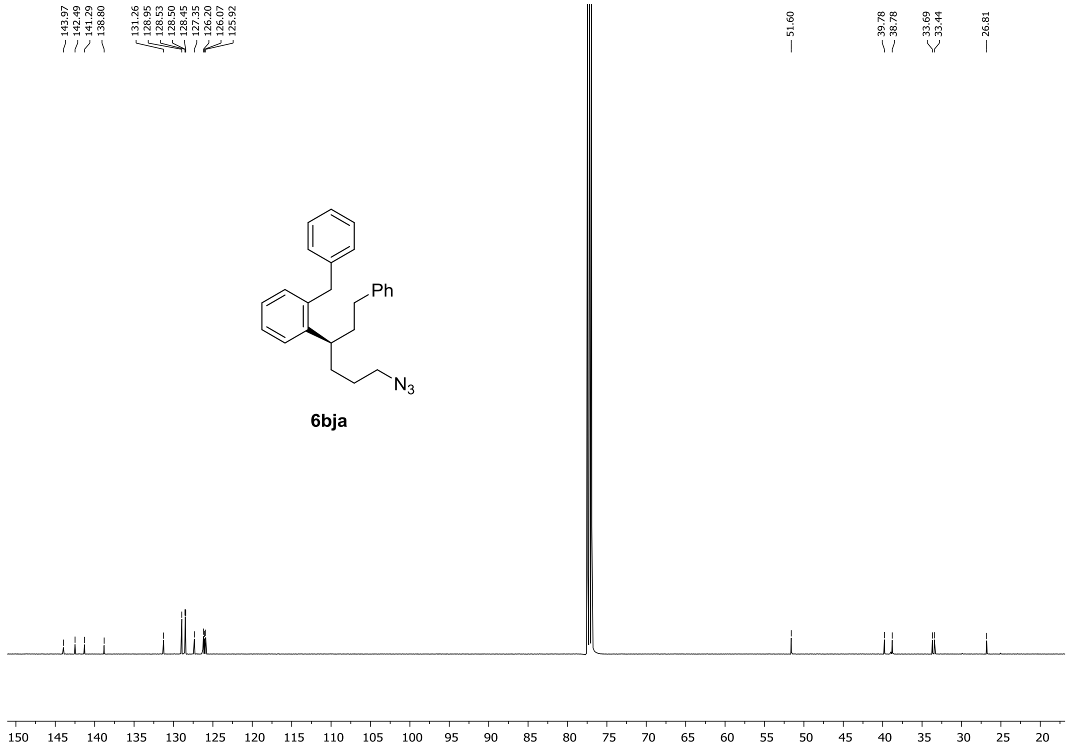

Figure S52: ^{1}H NMR of **(2*S*,4*S*,6*S*)-6aka** (400 MHz, $CDCl_3$)

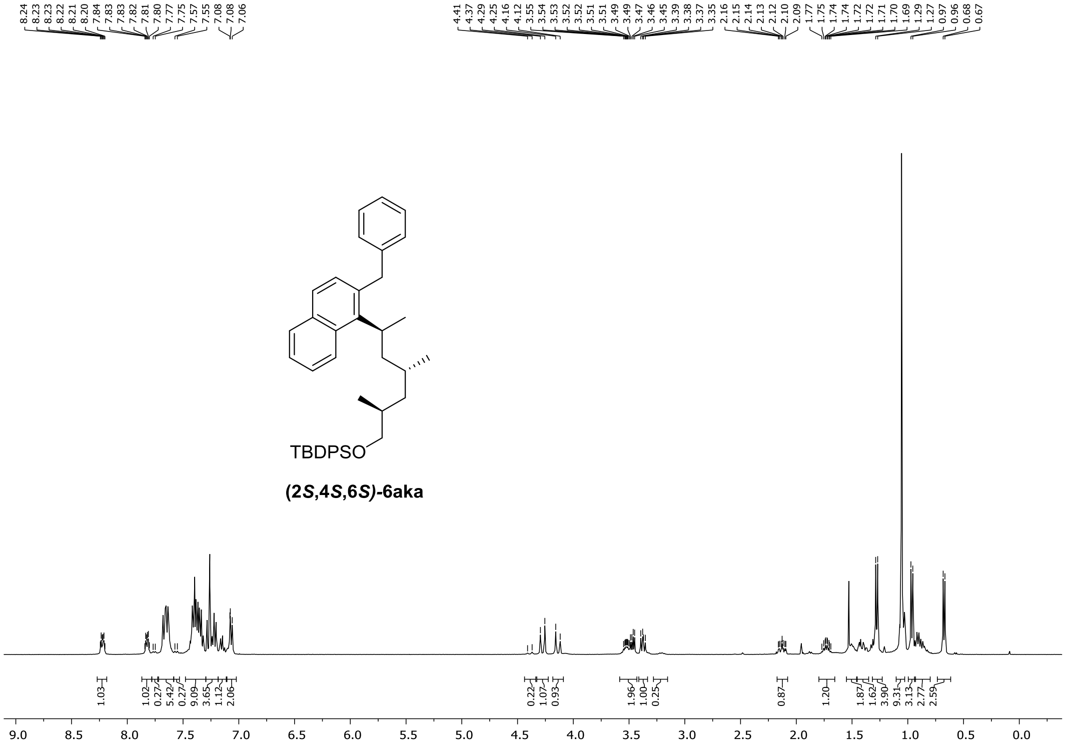

Figure S53: ^{13}C{^{1}H} NMR of **(2*S*,4*S*,6*S*)-6aka** (101 MHz, $CDCl_3$)

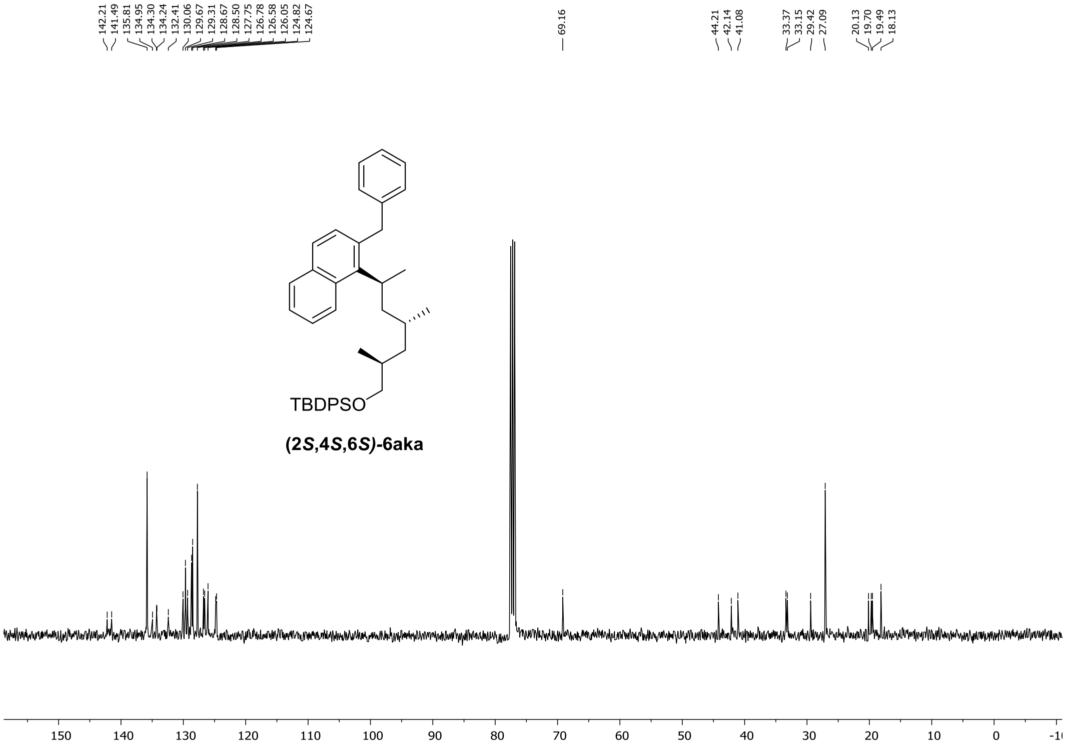

Figure S54: ^{1}H NMR of the crude reaction mixture for product **(2*S*,4*S*,6*R*)-6ala** (400 MHz, $CDCl_3$).

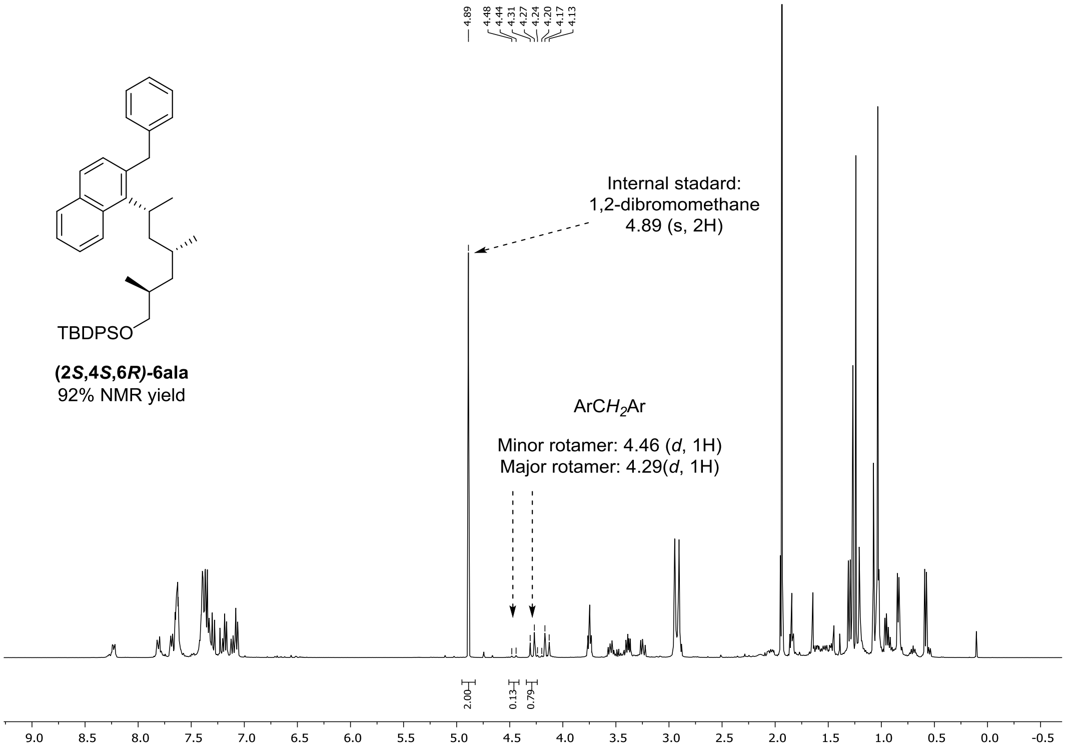

Figure S55: ^{1}H NMR of **(2*S*,4*S*,6*R*)-6ala** (400 MHz, $CDCl_3$)

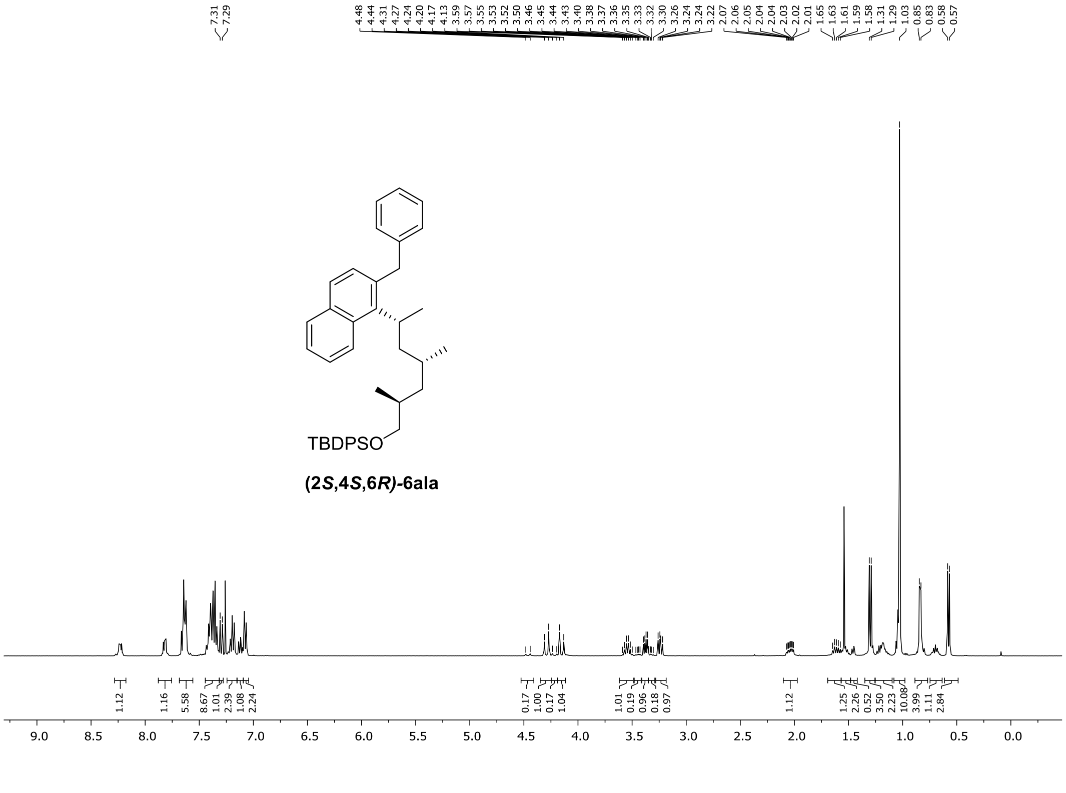

Figure S56: $^{13}C\{^{1}H\}$ NMR of **(2*S*,4*S*,6*R*)-6ala** (101 MHz, $CDCl_3$)

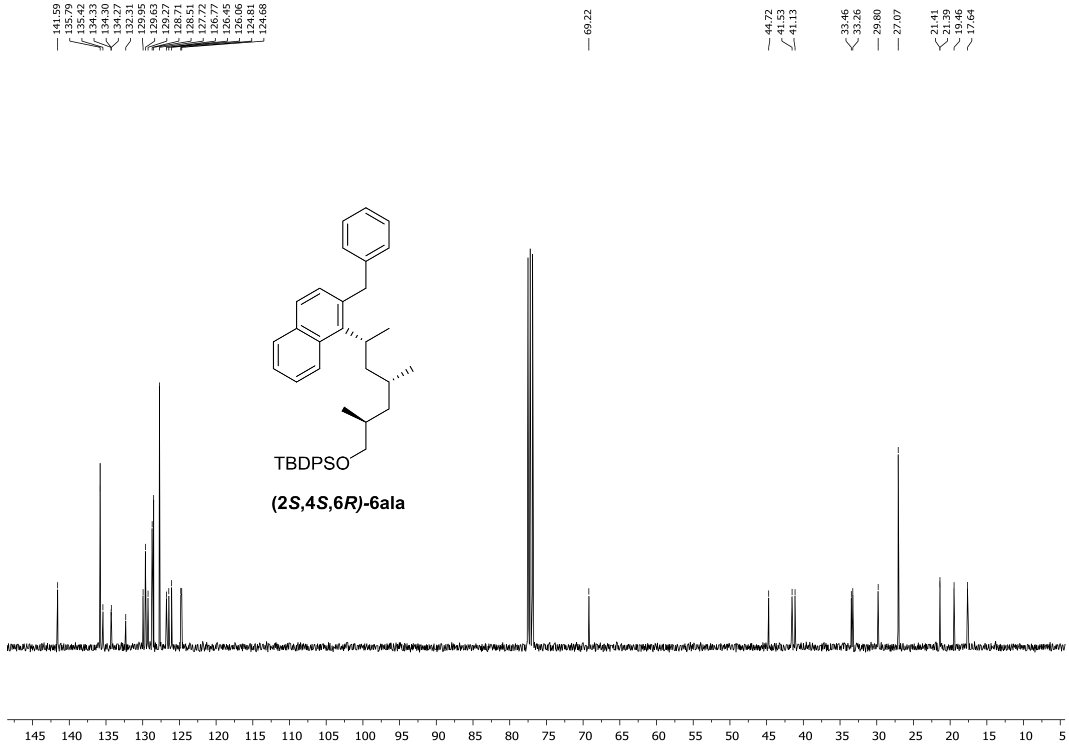

Figure S57: ^{1}H NMR of **6aab** (400 MHz, $CDCl_3$)

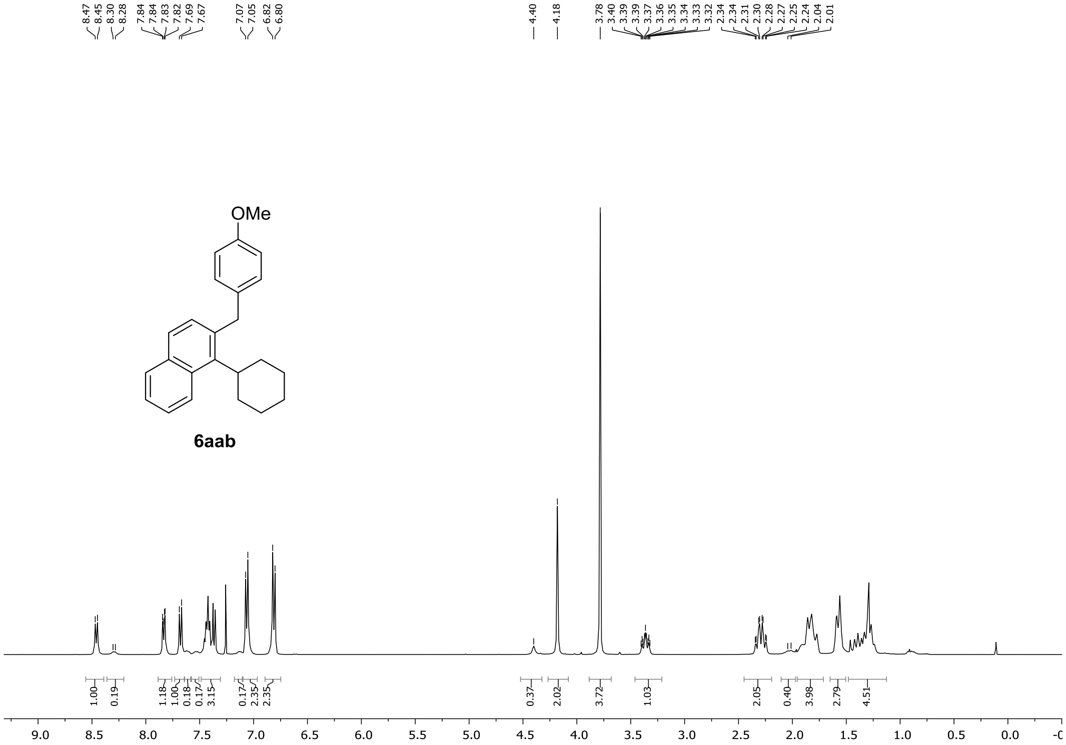

Figure S58: ^{13}C{^{1}H} NMR of **6aab** (101 MHz, $CDCl_3$)

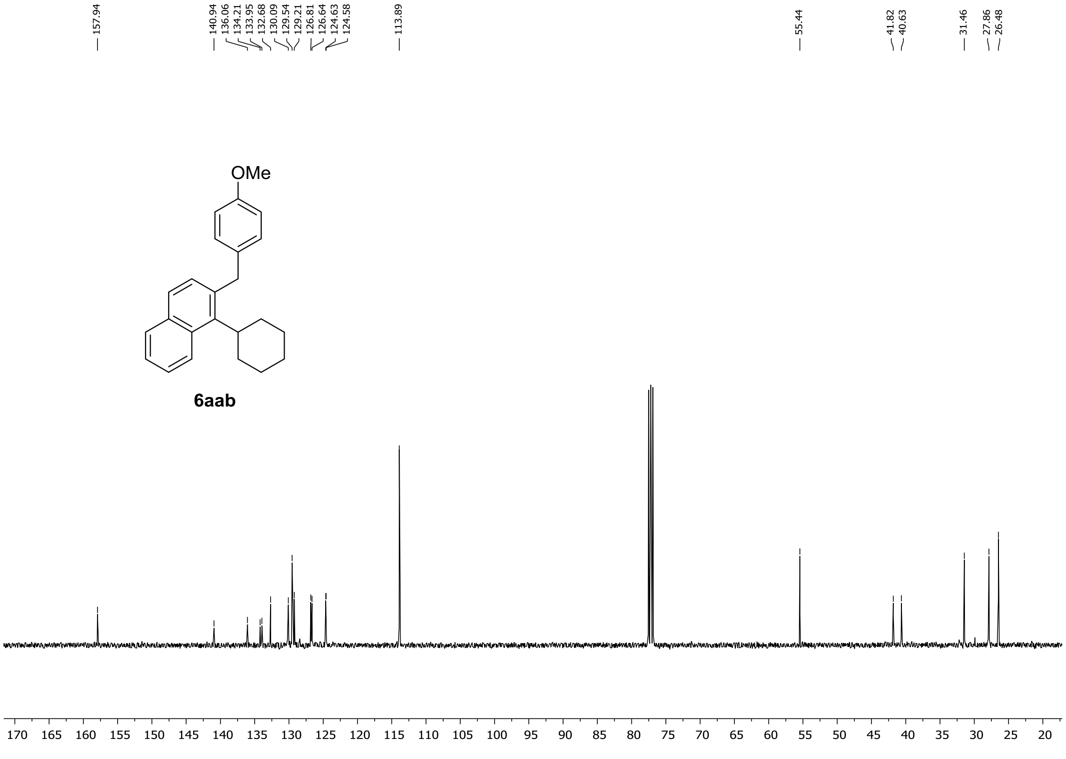

Figure S59: ^{1}H NMR of **6aac** (400 MHz, $CDCl_3$)

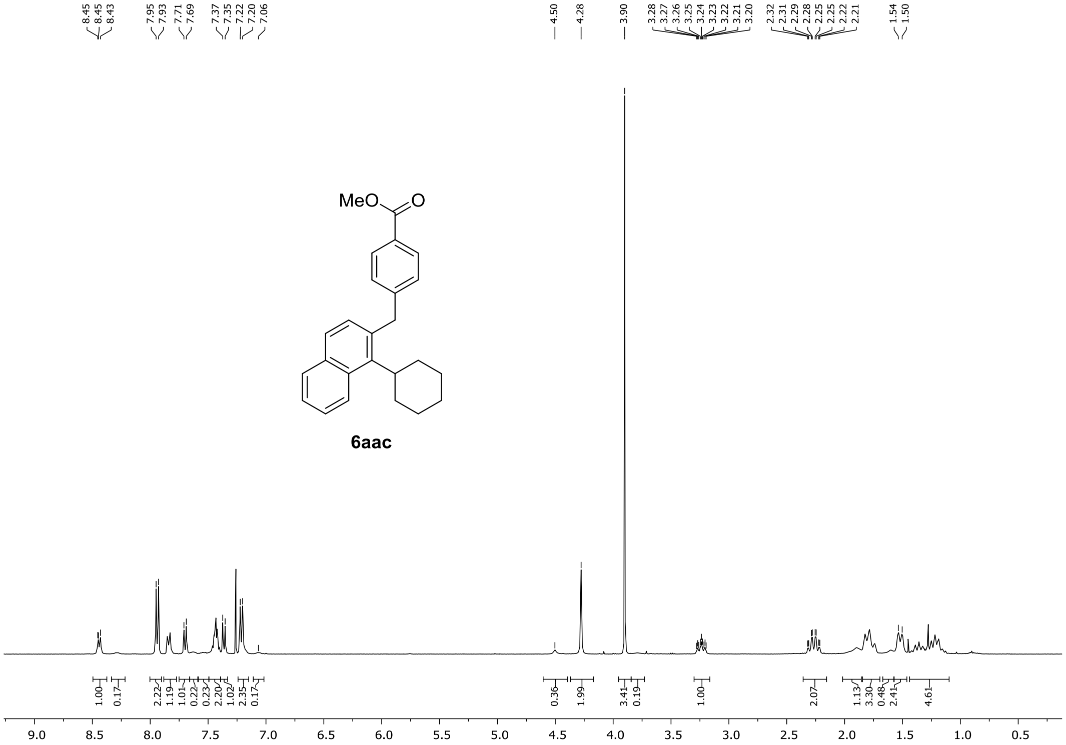

Figure S60: ^{13}C{^{1}H} NMR of **6aac** (101 MHz, $CDCl_3$)

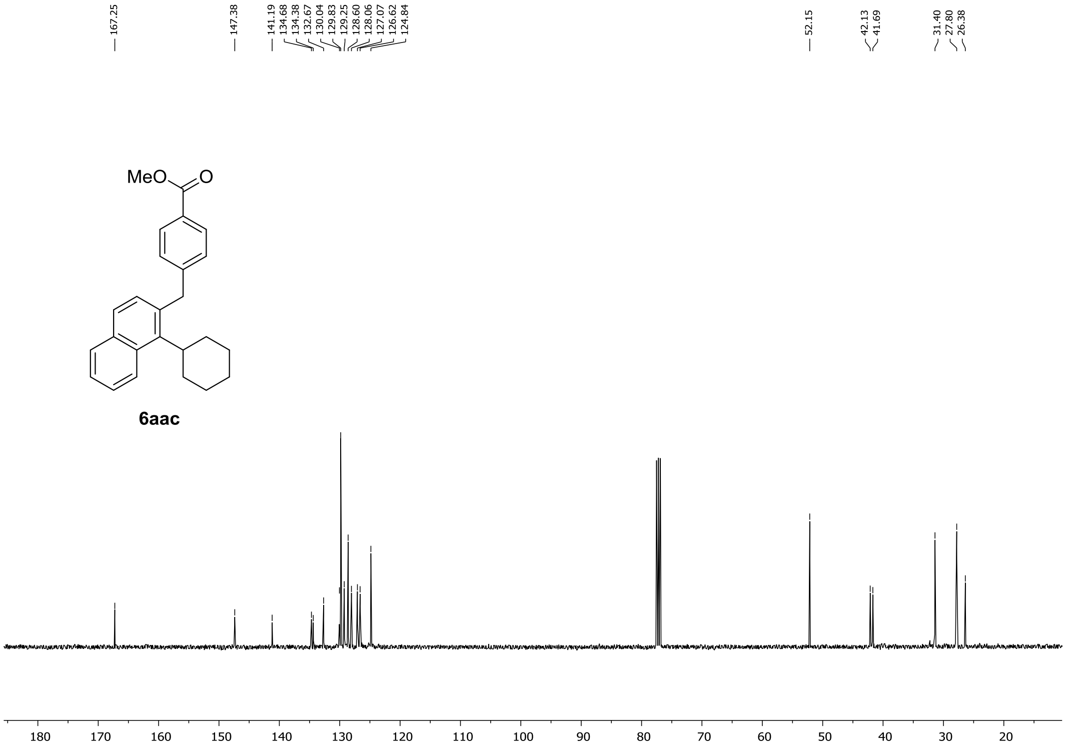

Figure S61: ^{1}H NMR of **6aad** (400 MHz, $CDCl_3$)

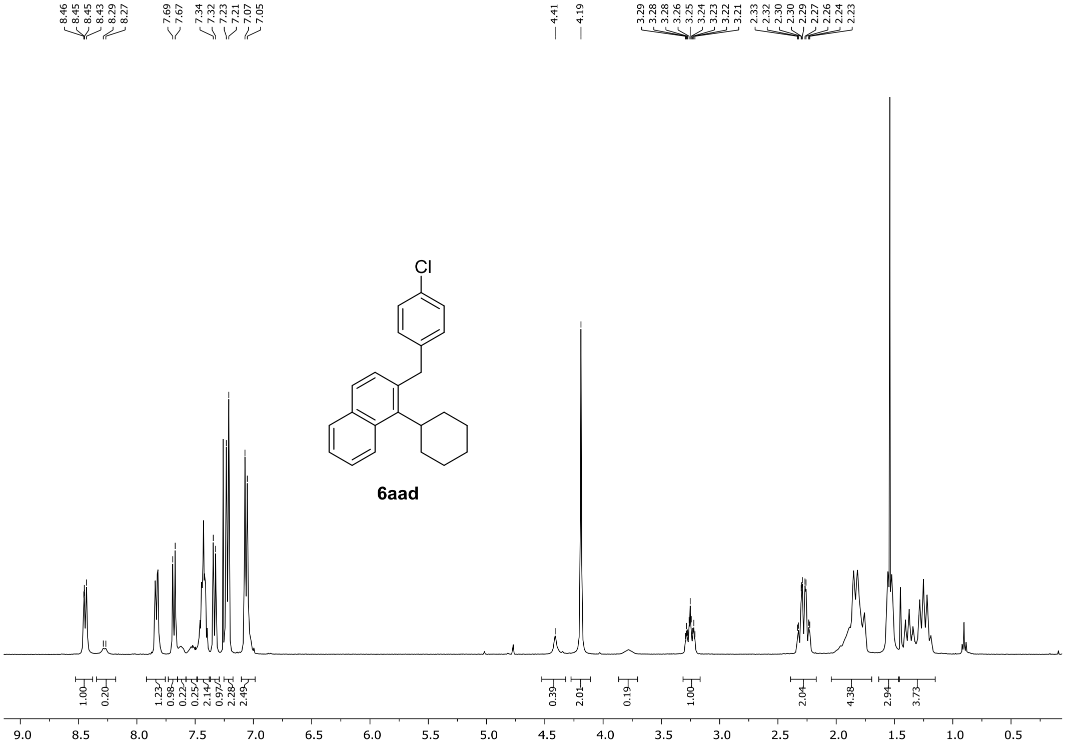

Figure S62: $^{13}C\{^{1}H\}$ NMR of **6aad** (101 MHz, $CDCl_3$)

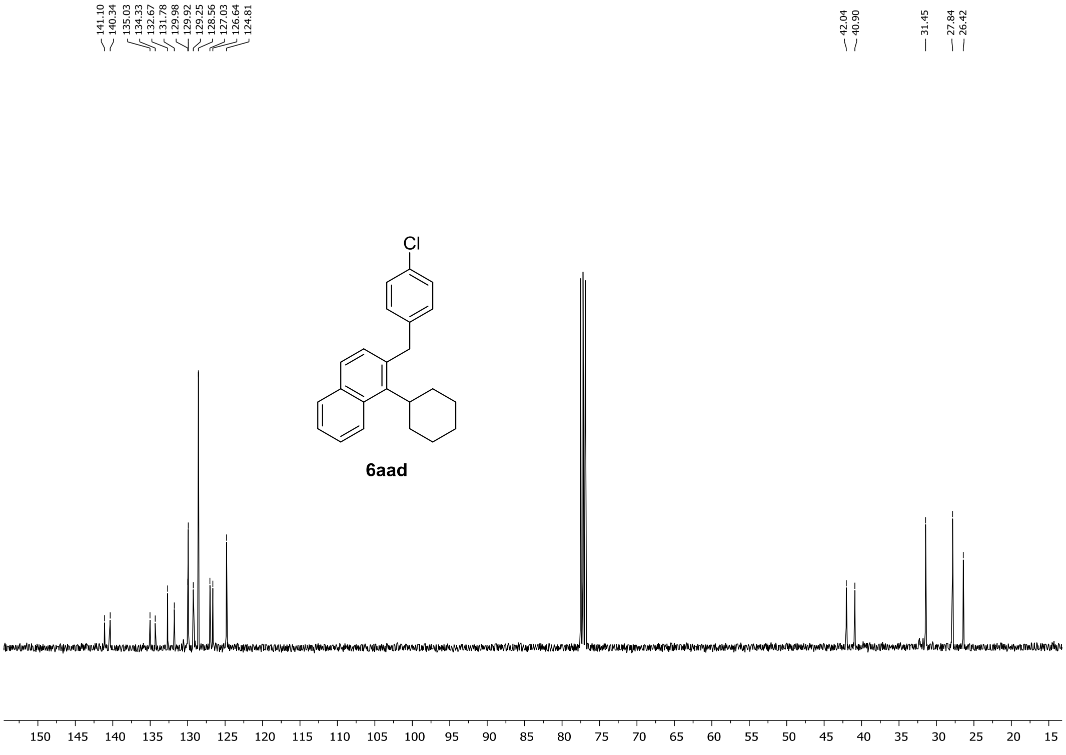

Figure S63: ^{1}H NMR of **6aae** (400 MHz, $CDCl_3$)

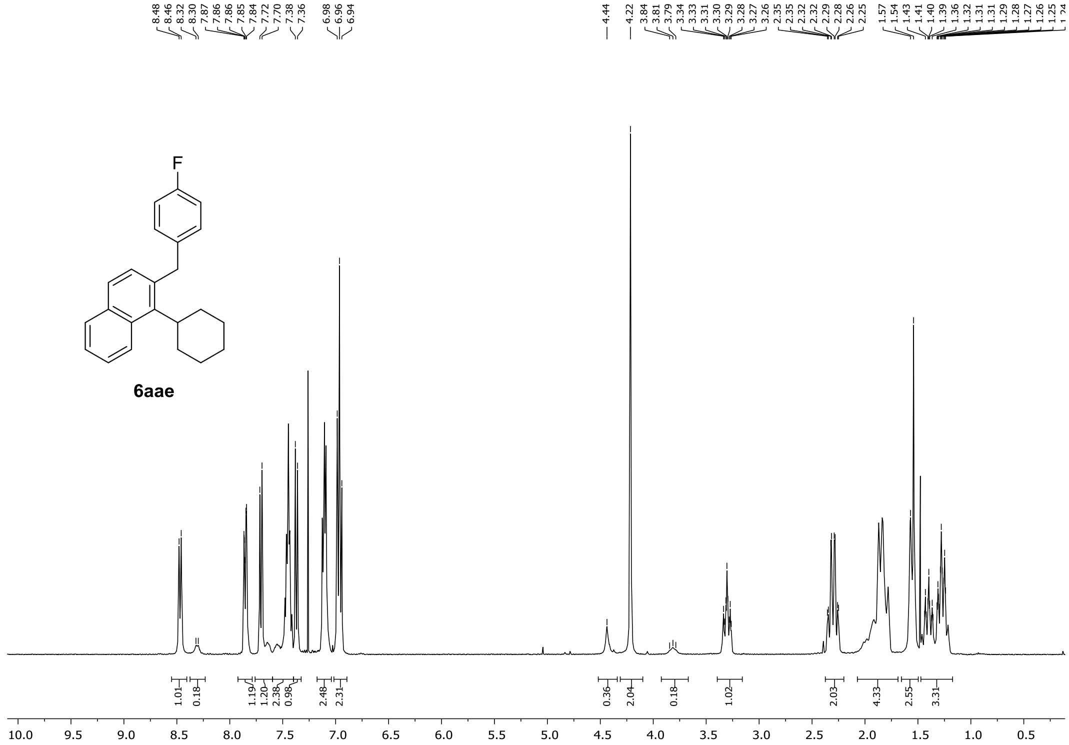

Figure S64: $^{13}C\{^{1}H\}$ NMR of **6aae** (101 MHz, $CDCl_3$)

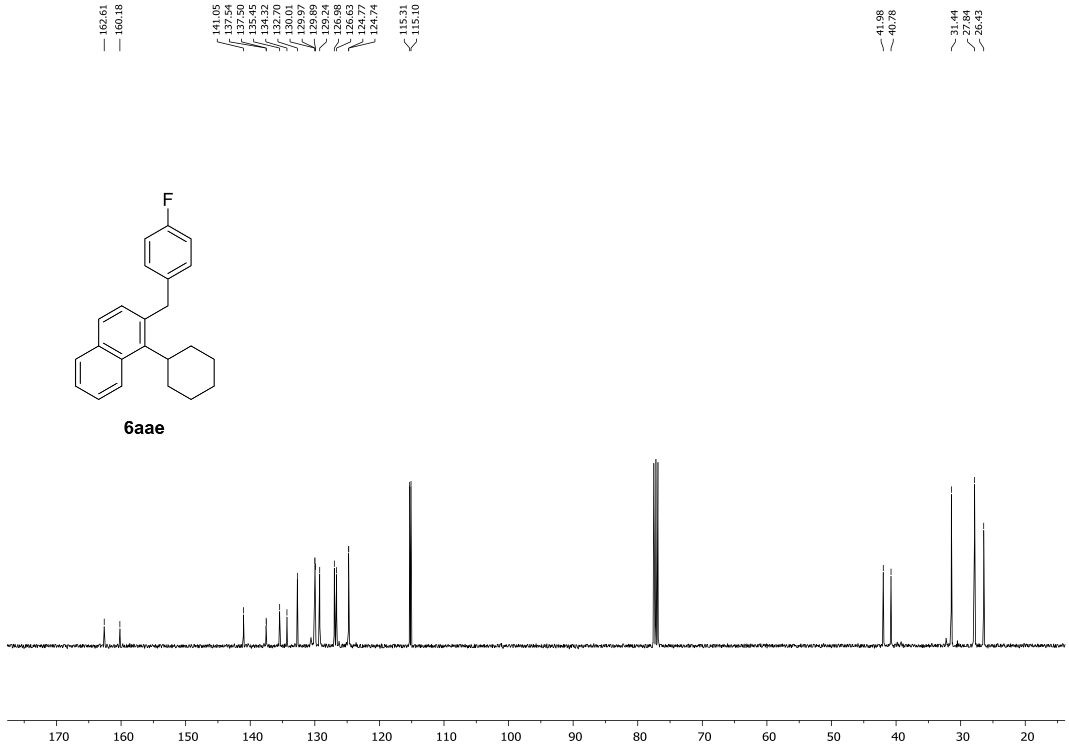

Figure S65: ^{19}F NMR of **6aae** (376 MHz, $CDCl_3$)

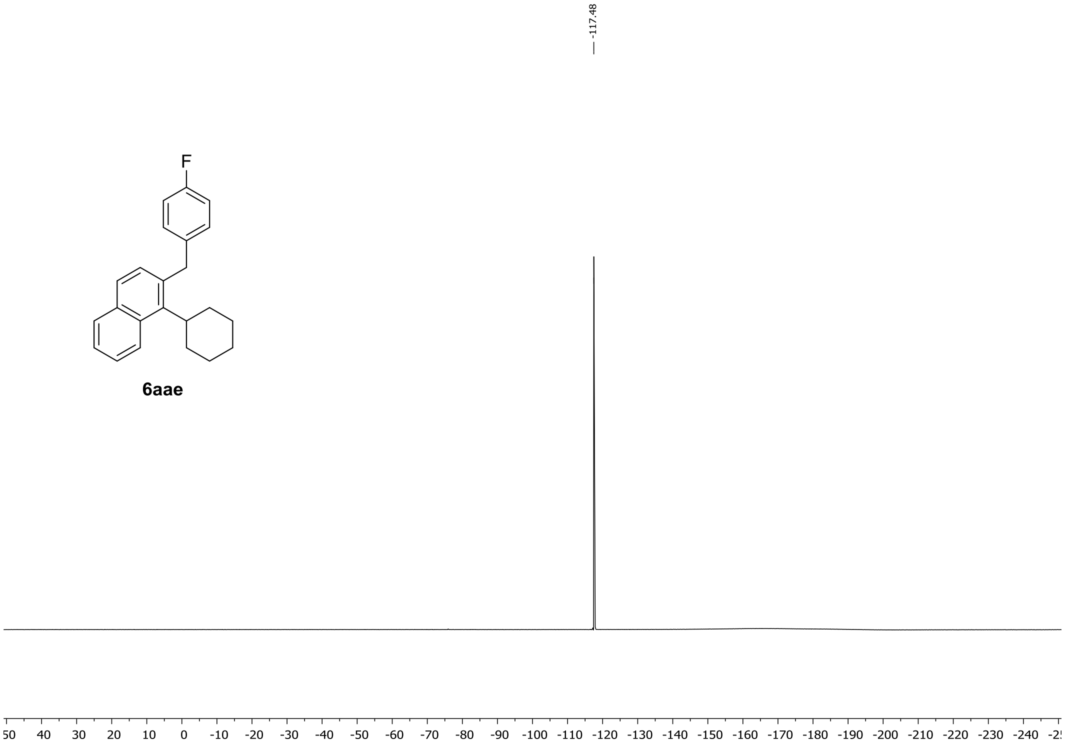

Figure S66: ^{1}H NMR of **6aaf** (400 MHz, $CDCl_3$)

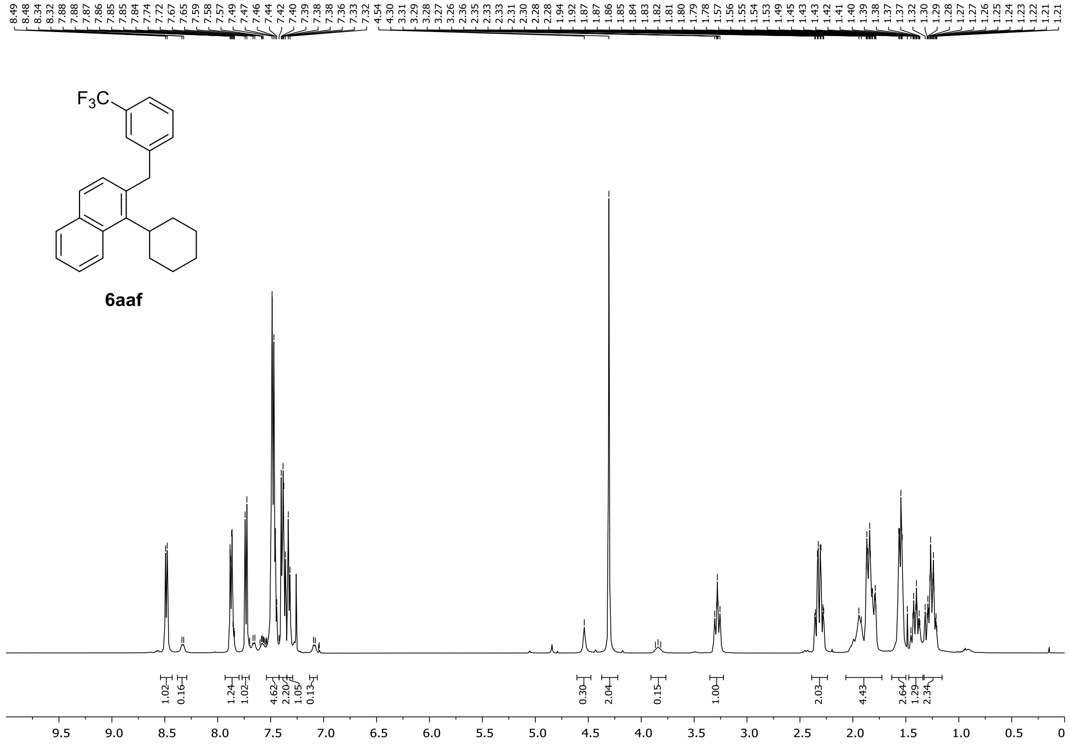

Figure S67: ^{13}C{^{1}H} NMR of **6aaf** (126 MHz, $CDCl_3$)

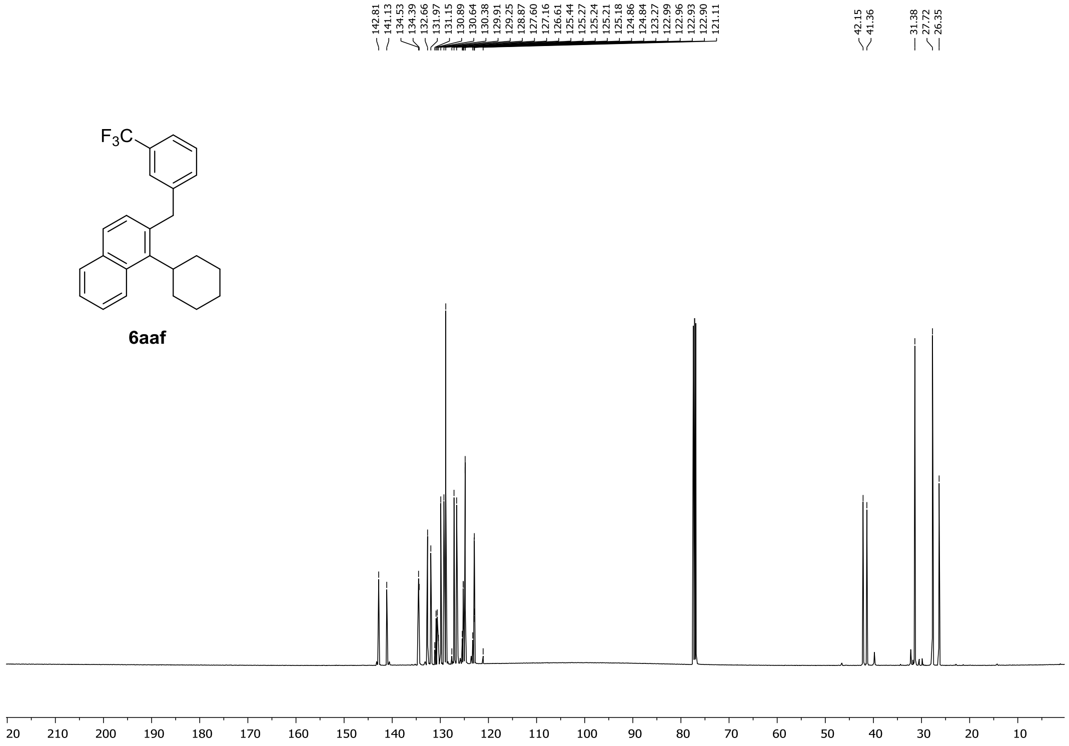

Figure S68: ^{19}F NMR of **6aaf** (376 MHz, $CDCl_3$)

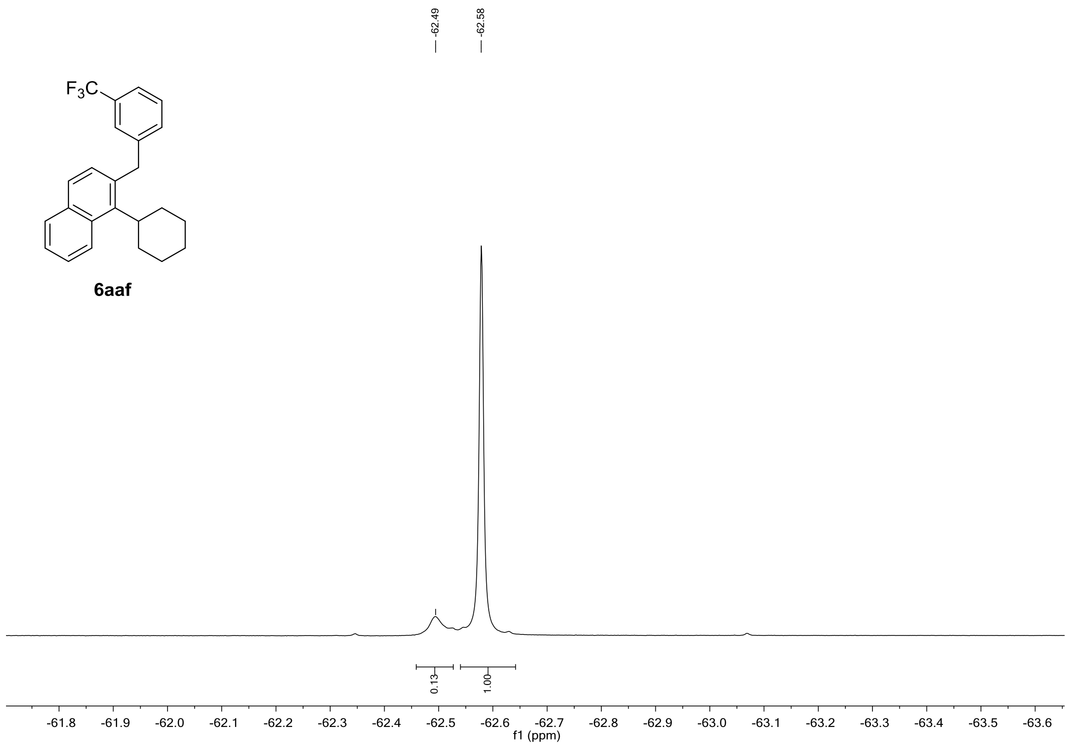

Figure S69: ^{1}H NMR of **6aag** (400 MHz, $CDCl_3$)

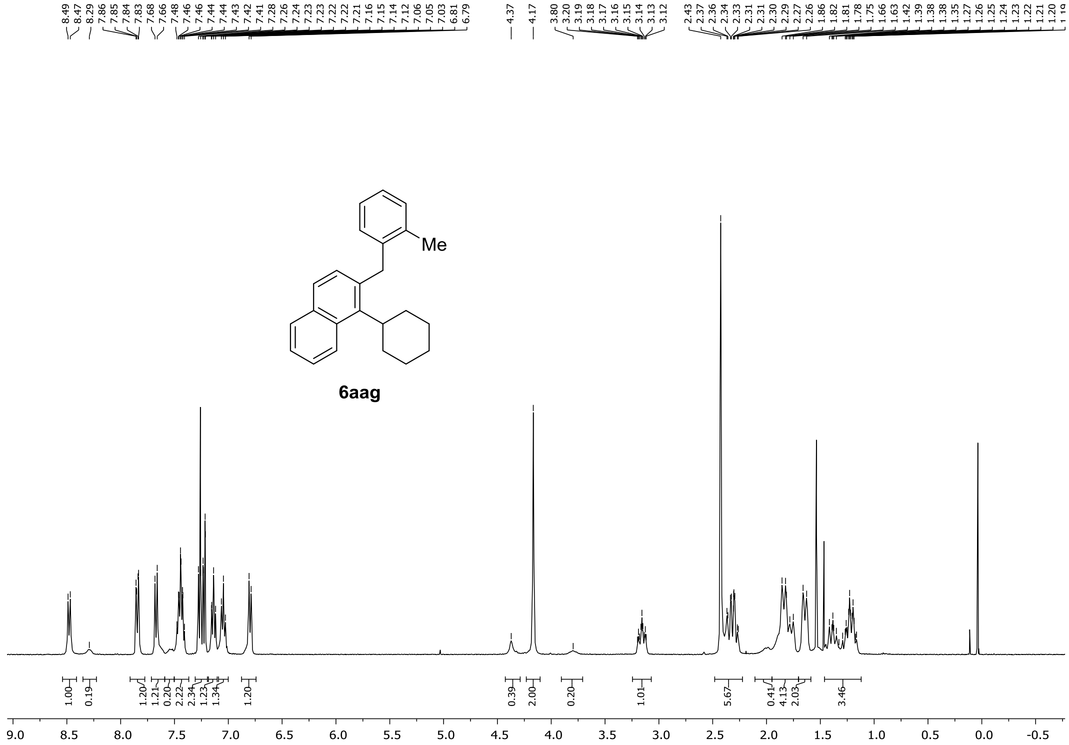

Figure S70: ^{13}C{^{1}H} NMR of **6aag** (101 MHz, $CDCl_3$)

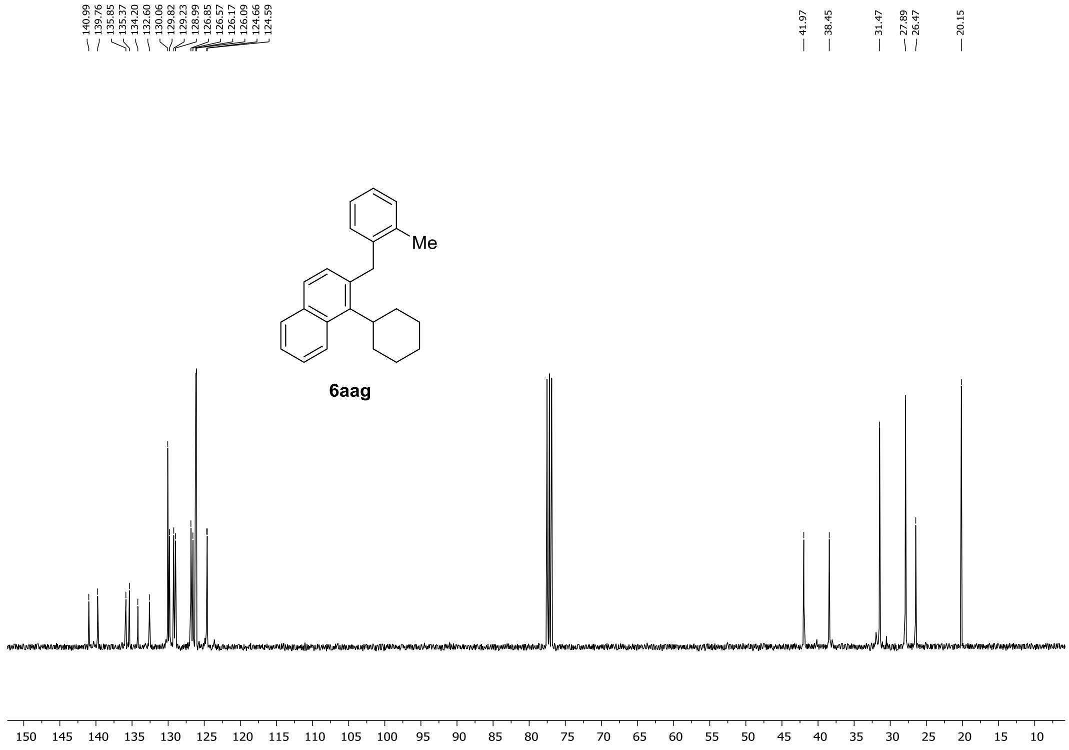

Figure S71: ^{1}H NMR of **6aah** (400 MHz, $CDCl_3$)

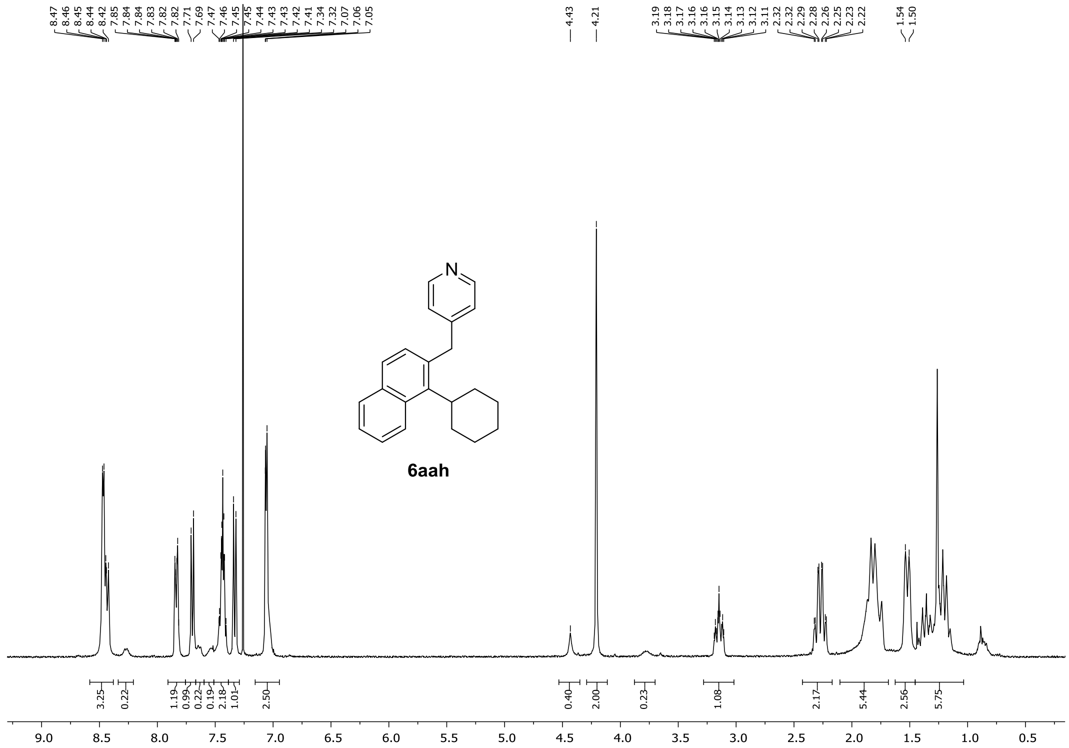

Figure S72: ^{13}C{^{1}H} NMR of **6aah** (101 MHz, $CDCl_3$)

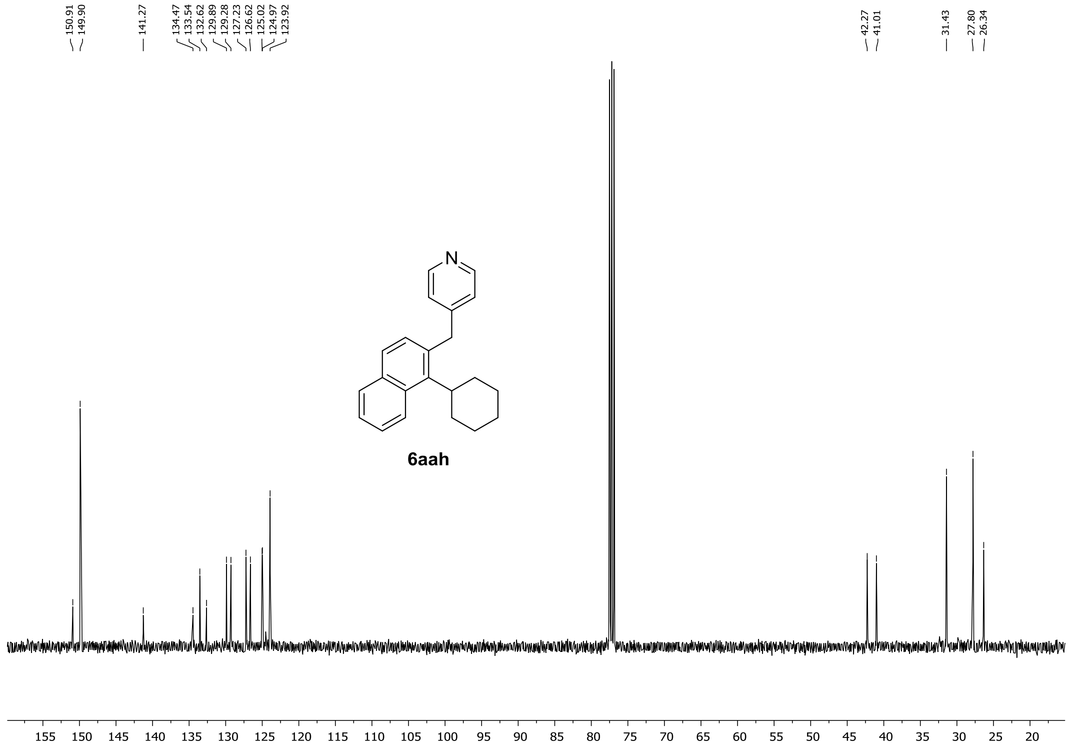

Figure S73: ^{1}H NMR of **6baa** (400 MHz, $CDCl_3$)

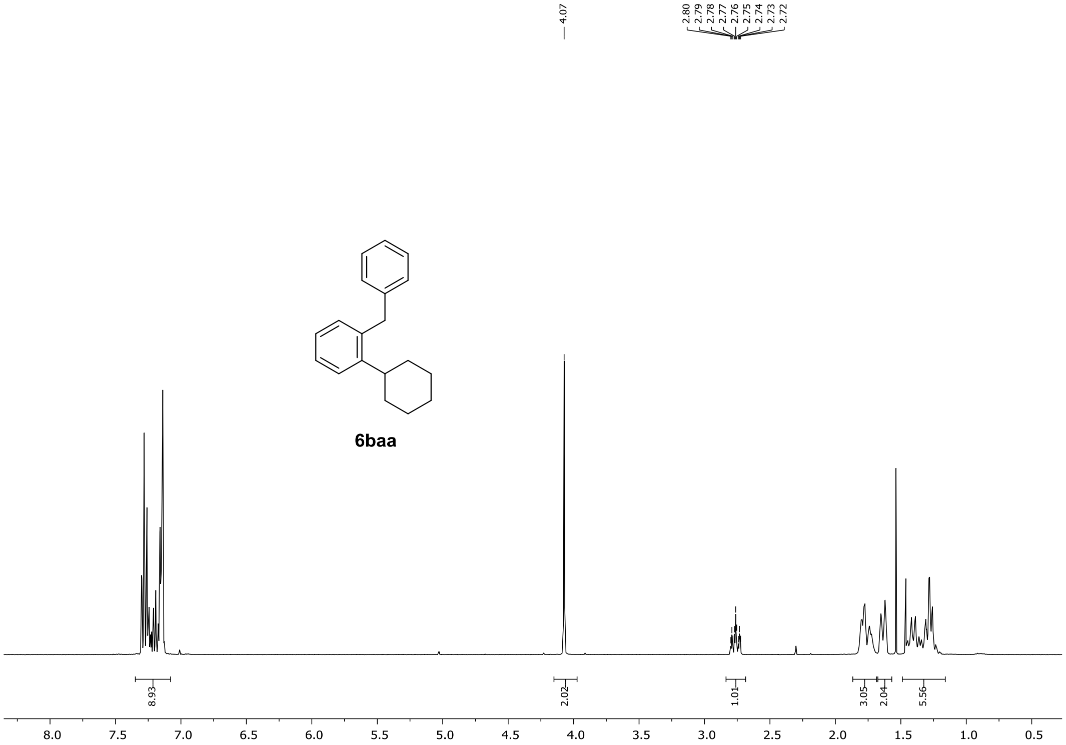

Figure S74: $^{13}C\{^{1}H\}$ NMR of **6baa** (101 MHz, $CDCl_3$)

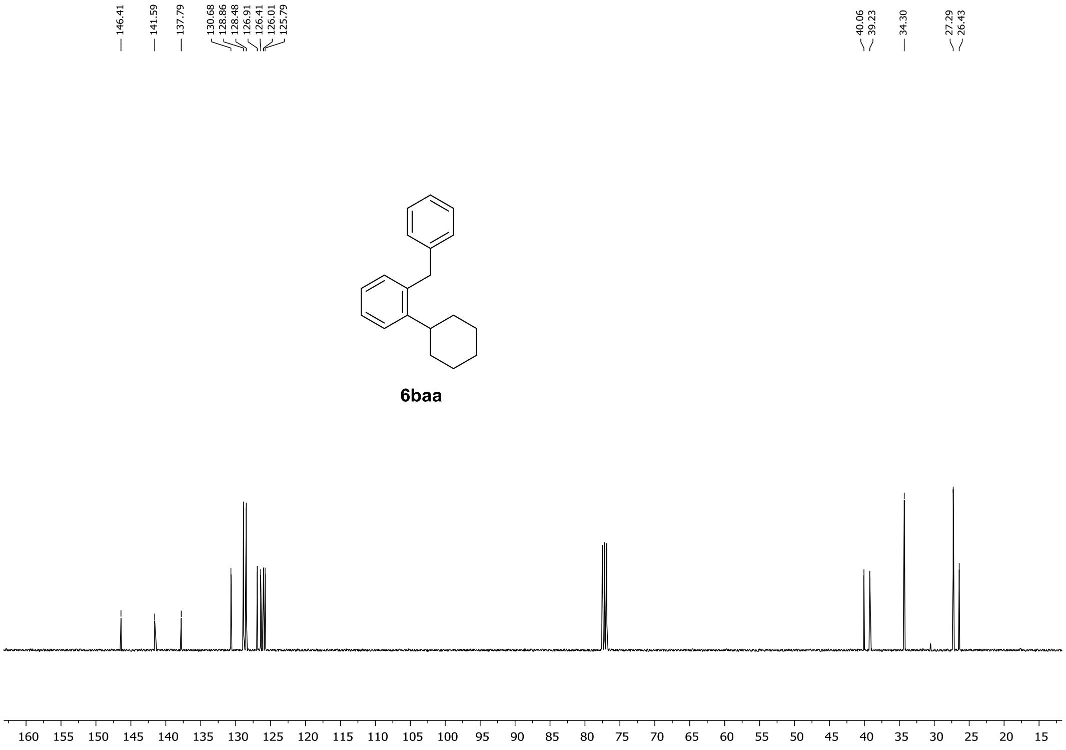

Figure S75: ^{1}H NMR of **6caa** (400 MHz, $CDCl_3$)

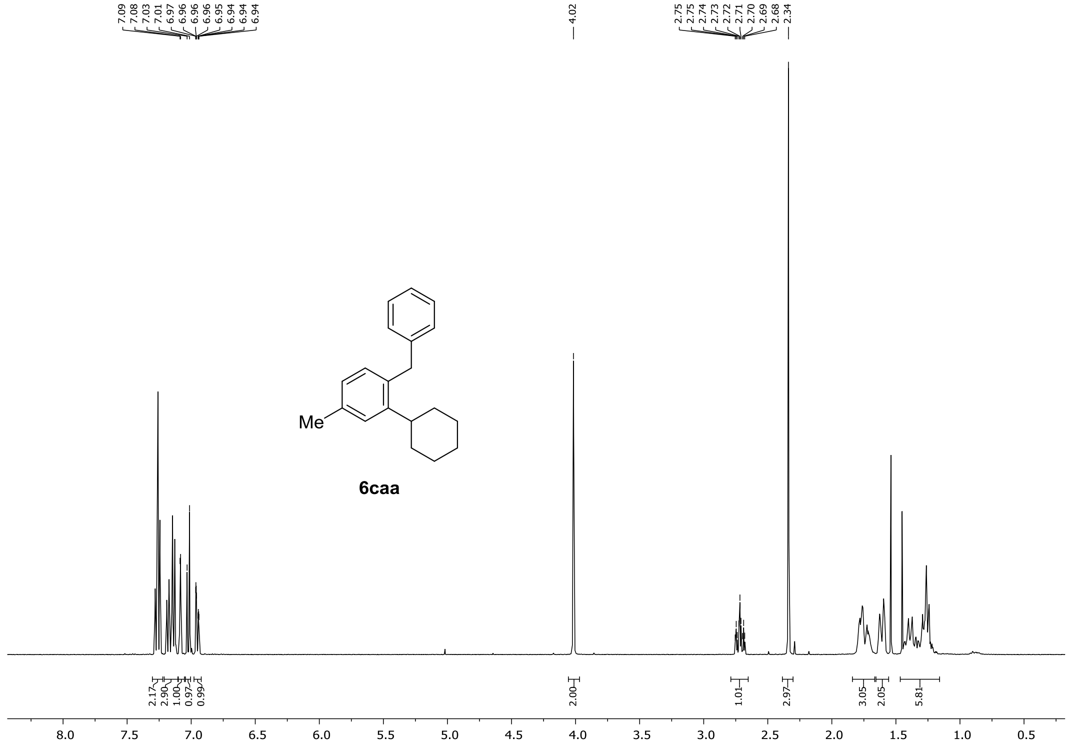

Figure S76: ^{13}C{^{1}H} NMR of **6caa** (101 MHz, $CDCl_3$)

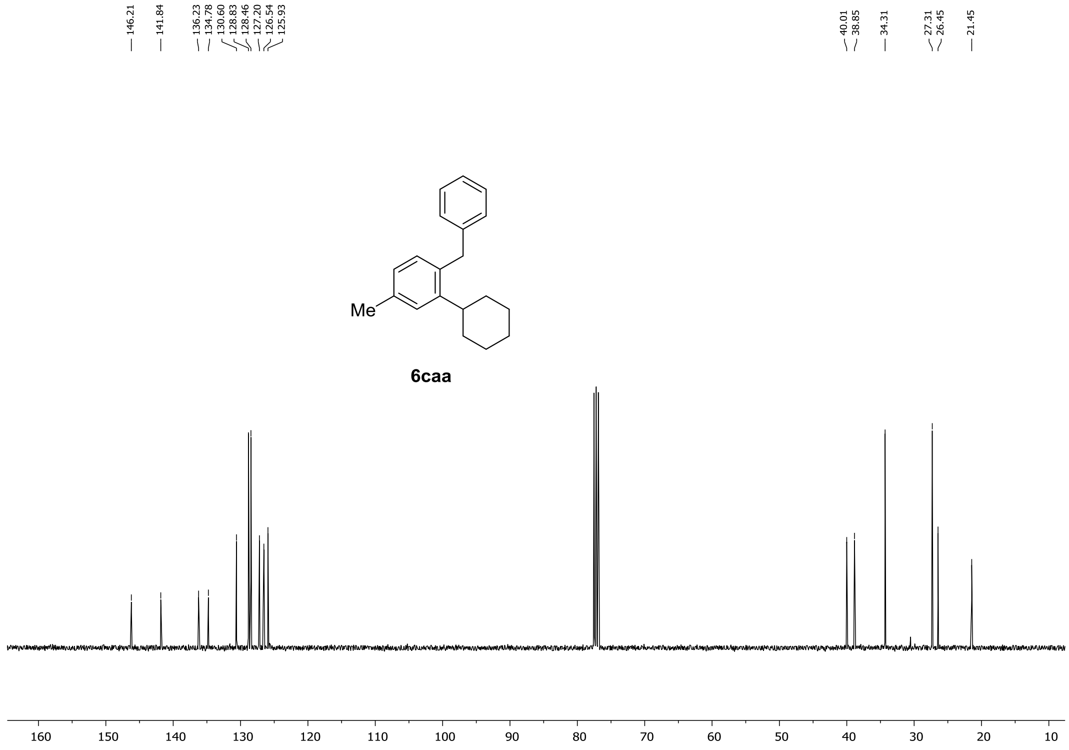

Figure S77: ^{1}H NMR of the crude reaction mixture for product **6dai** (400 MHz, $CDCl_3$).

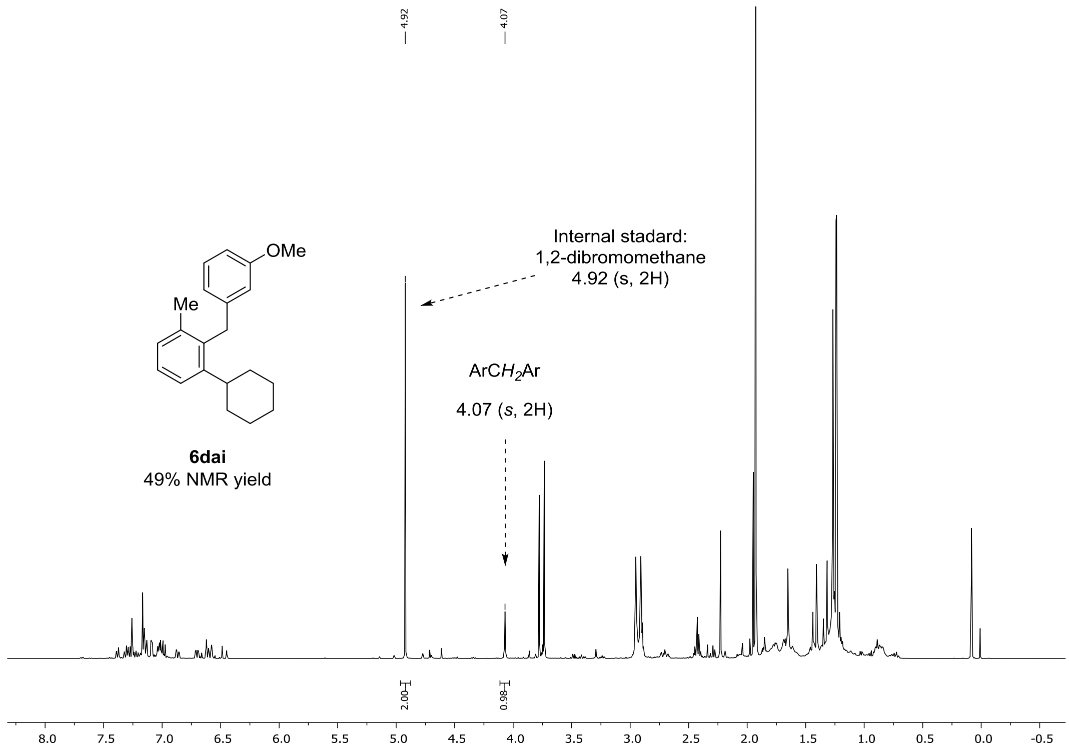

Figure S78: ^{1}H NMR of **6dai** (400 MHz, $CDCl_3$)

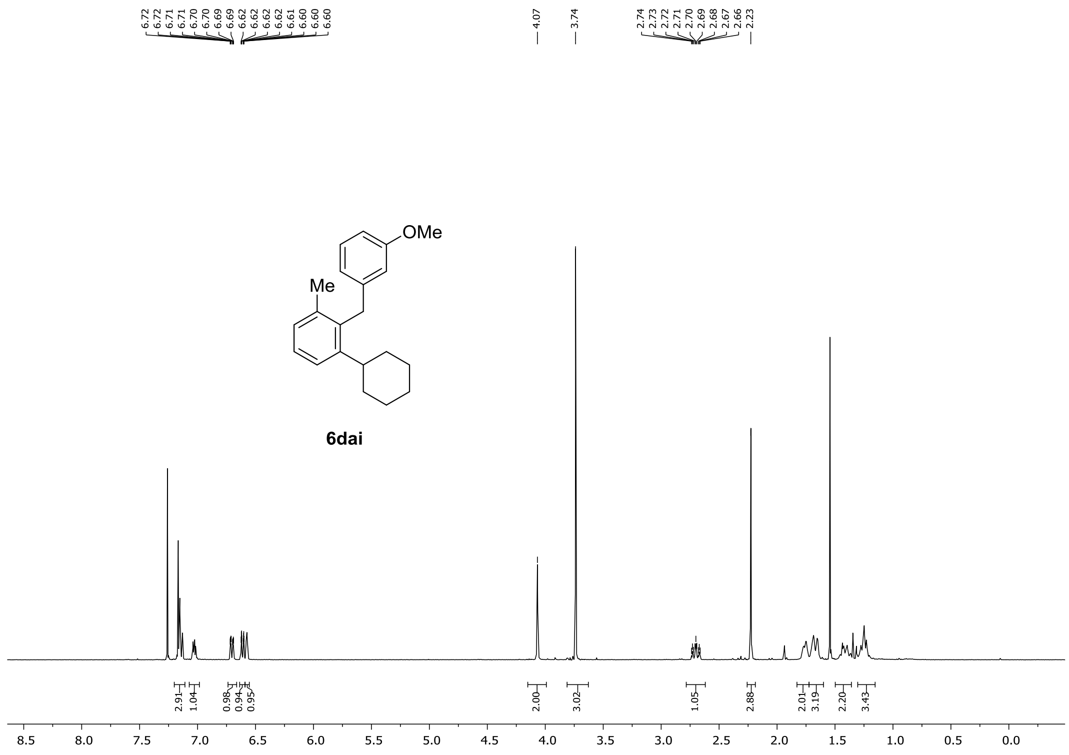

Figure S79: $^{13}C\{^{1}H\}$ NMR of **6dai** (126 MHz, $CDCl_3$)

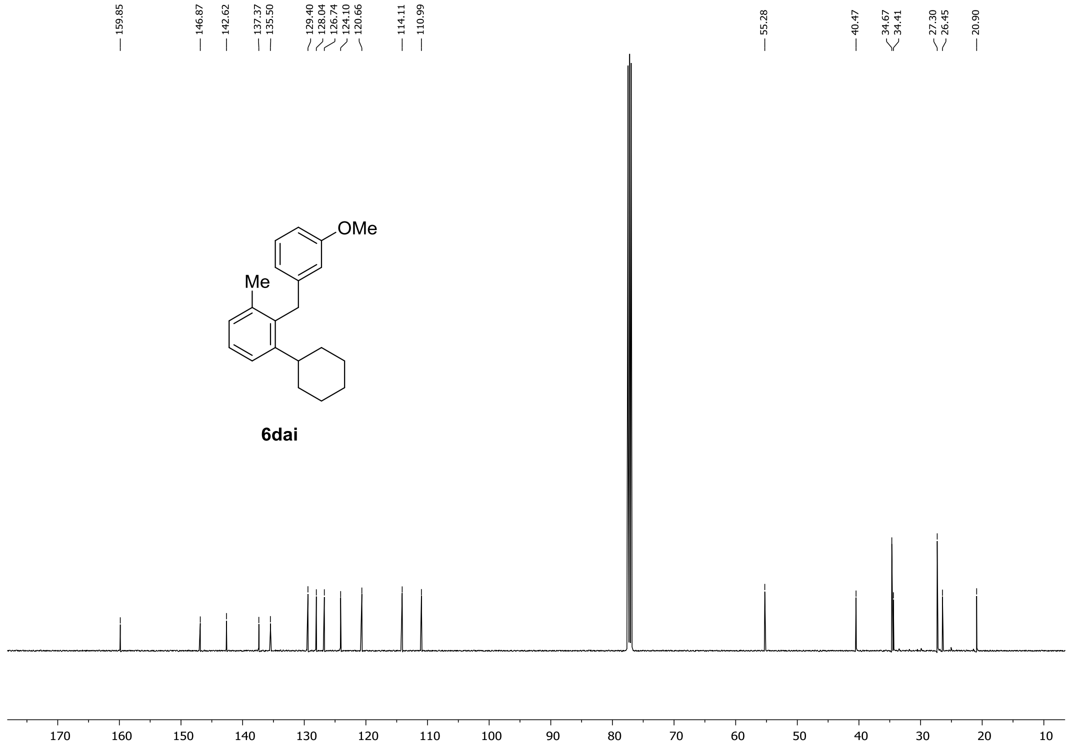

Figure S80: ^{1}H NMR of the crude reaction mixture for product **6eai** (400 MHz, $CDCl_3$).

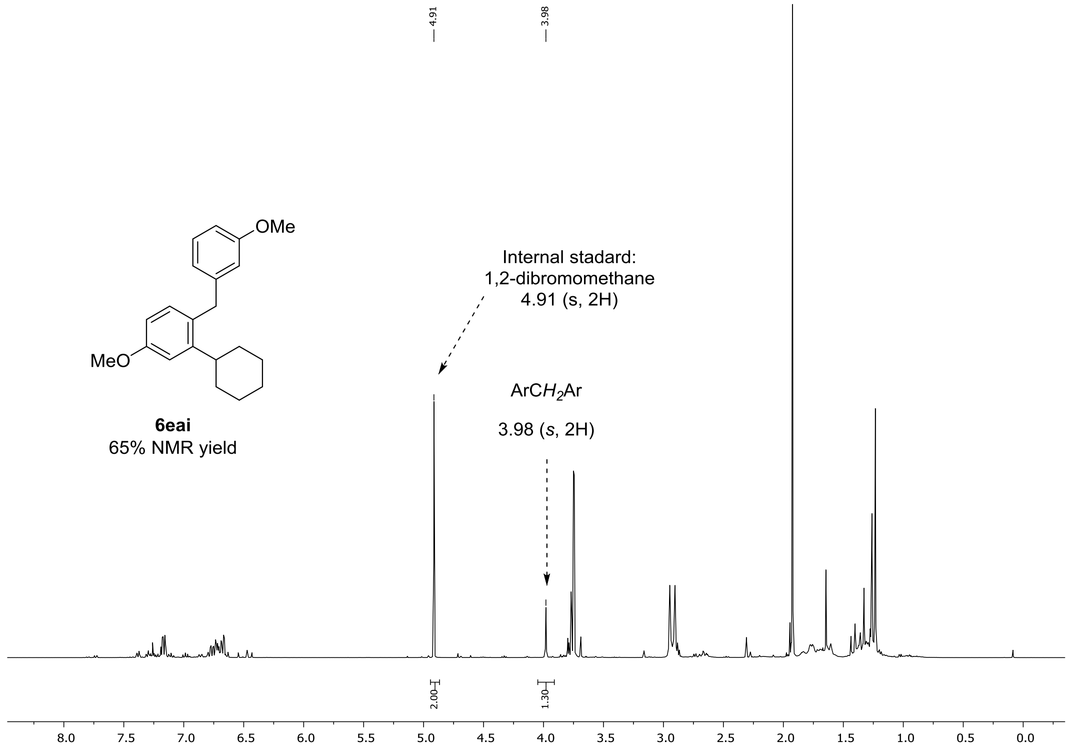

Figure S81: ^{1}H NMR of **6eai** (400 MHz, $CDCl_3$)

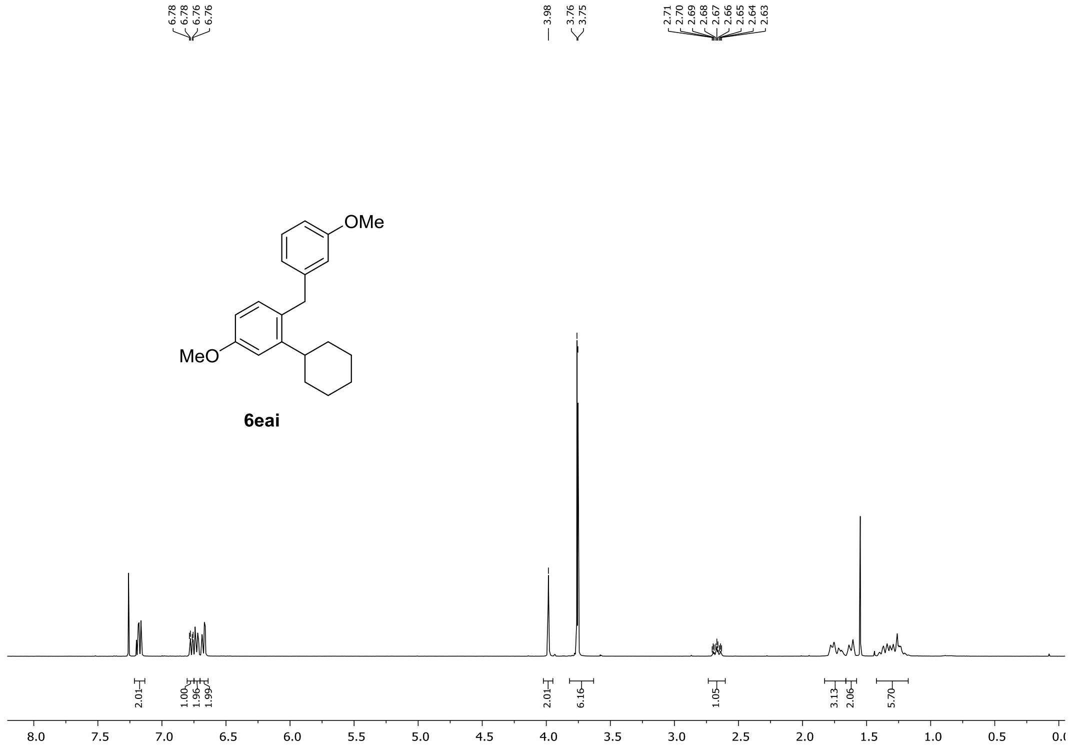

Figure S82: $^{13}C\{^1H\}$ NMR of **6eai** (101 MHz, $CDCl_3$)

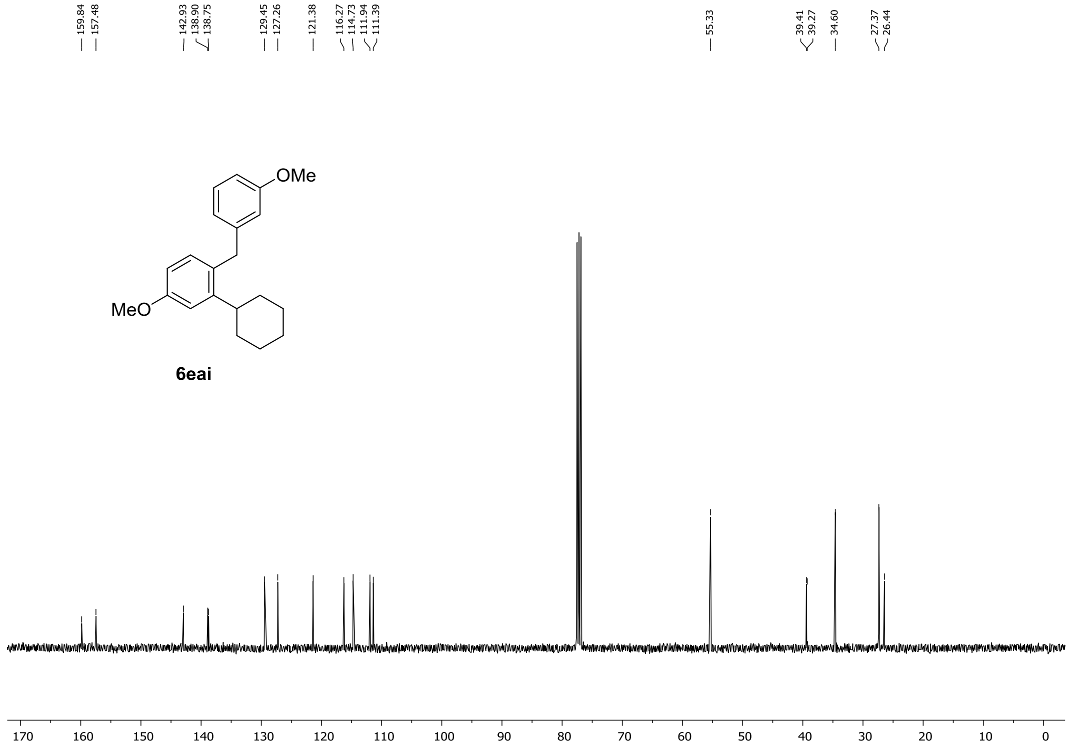

Figure S83: ^{1}H NMR of the crude reaction mixture for product **6fai** (400 MHz, $CDCl_3$).

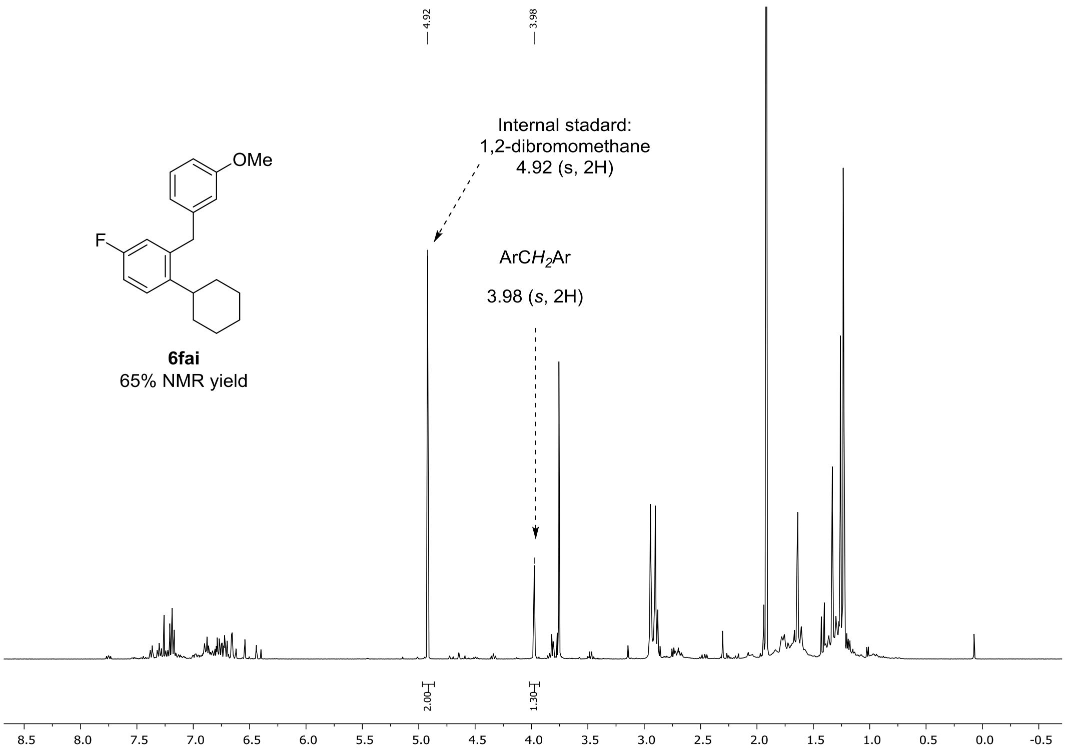

Figure S84: ^{1}H NMR of **6fai** (400 MHz, $CDCl_3$)

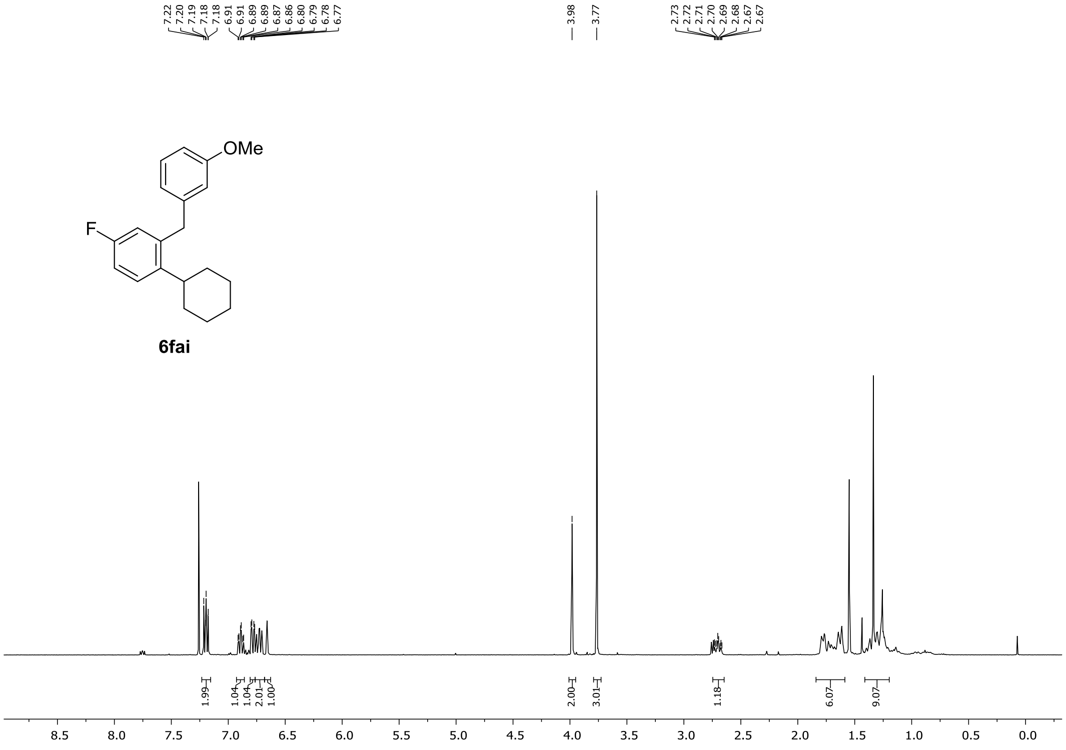

Figure S85: $^{13}C\{^{1}H\}$ NMR of **6fai** (126 MHz, $CDCl_3$)

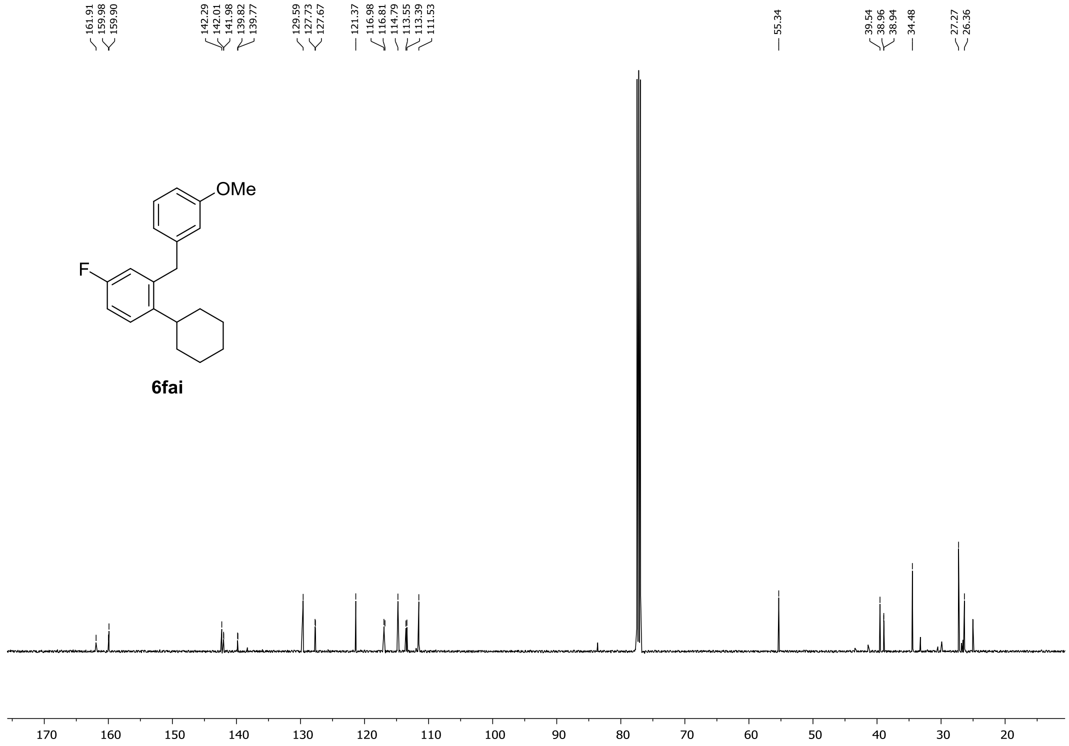

Figure S86: ^{19}F NMR of **6fai** (376 MHz, $CDCl_3$)

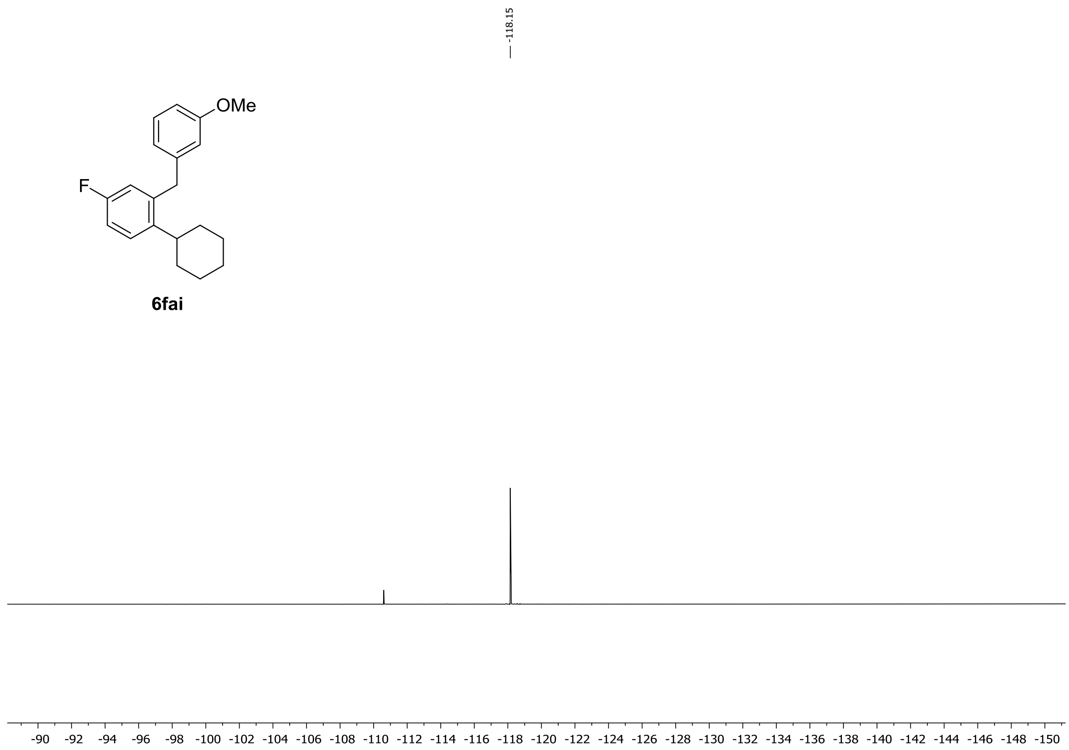

Figure S87: ^{1}H NMR of **6gai** (400 MHz, $CDCl_3$)

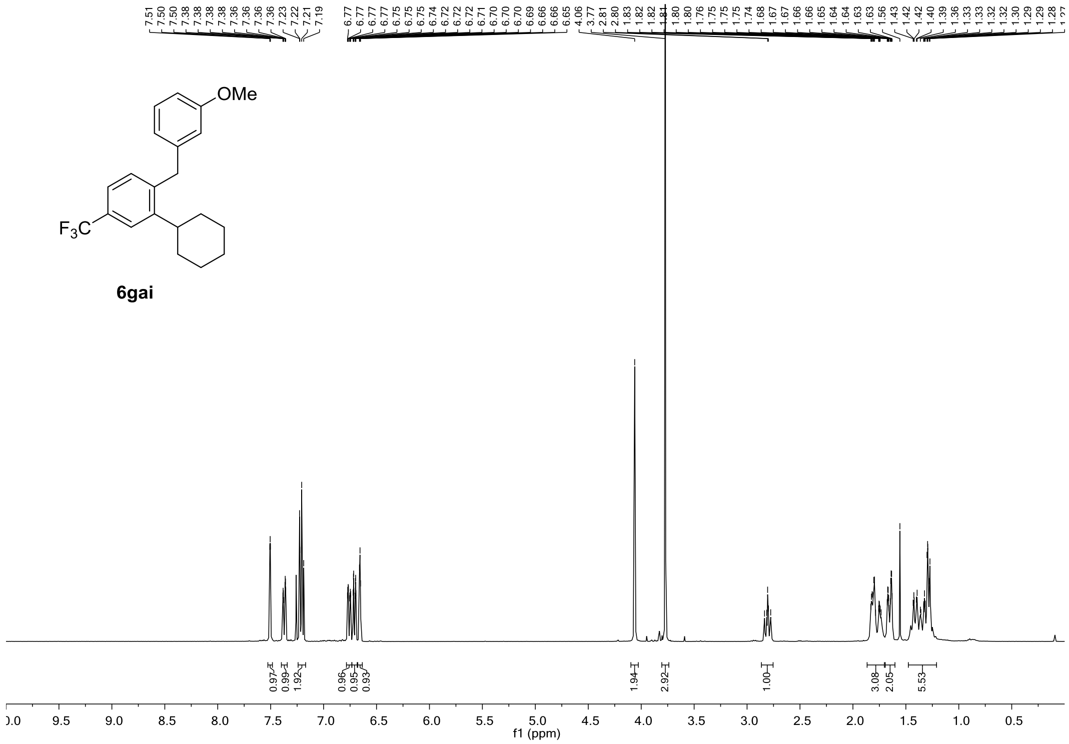

Figure S88: ^{13}C{^{1}H} NMR of **6gai** (125 MHz, $CDCl_3$)

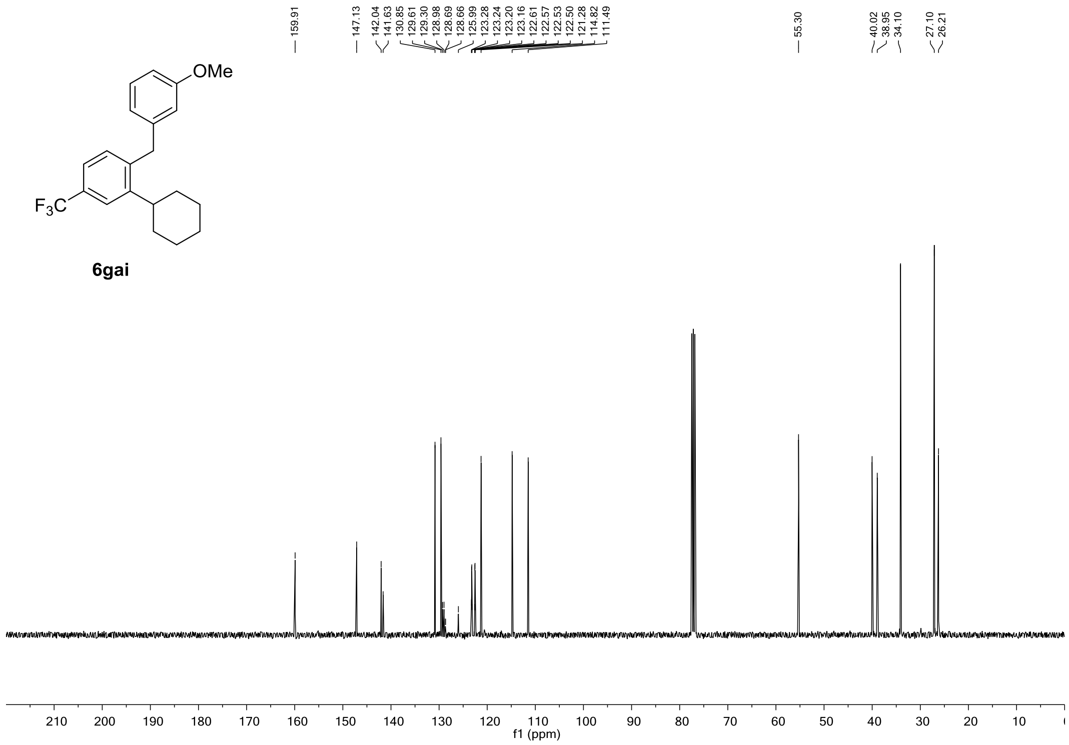

Figure S89: ^{19}F NMR of **6gai** (376 MHz, $CDCl_3$)

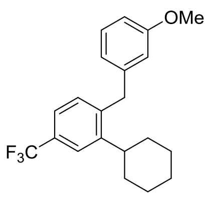

6gai

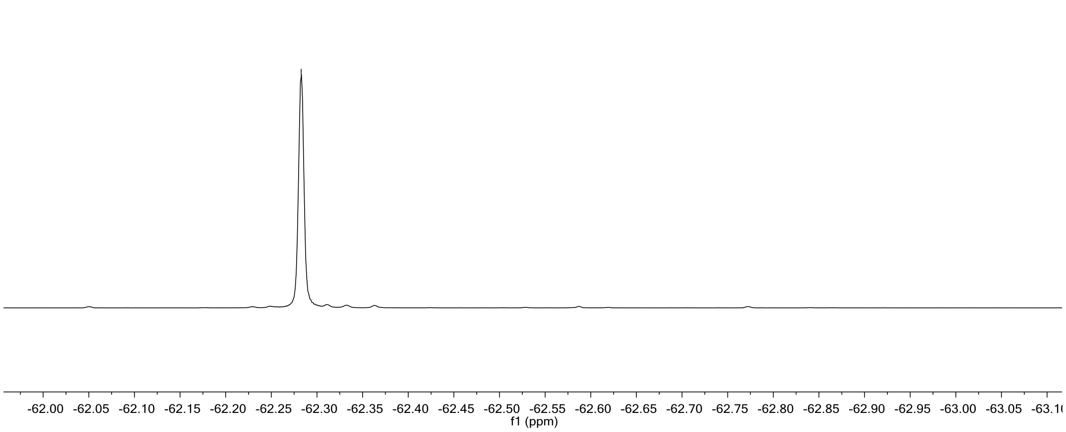

Figure S90: ^{1}H NMR of **6hai** (400 MHz, $CDCl_3$)

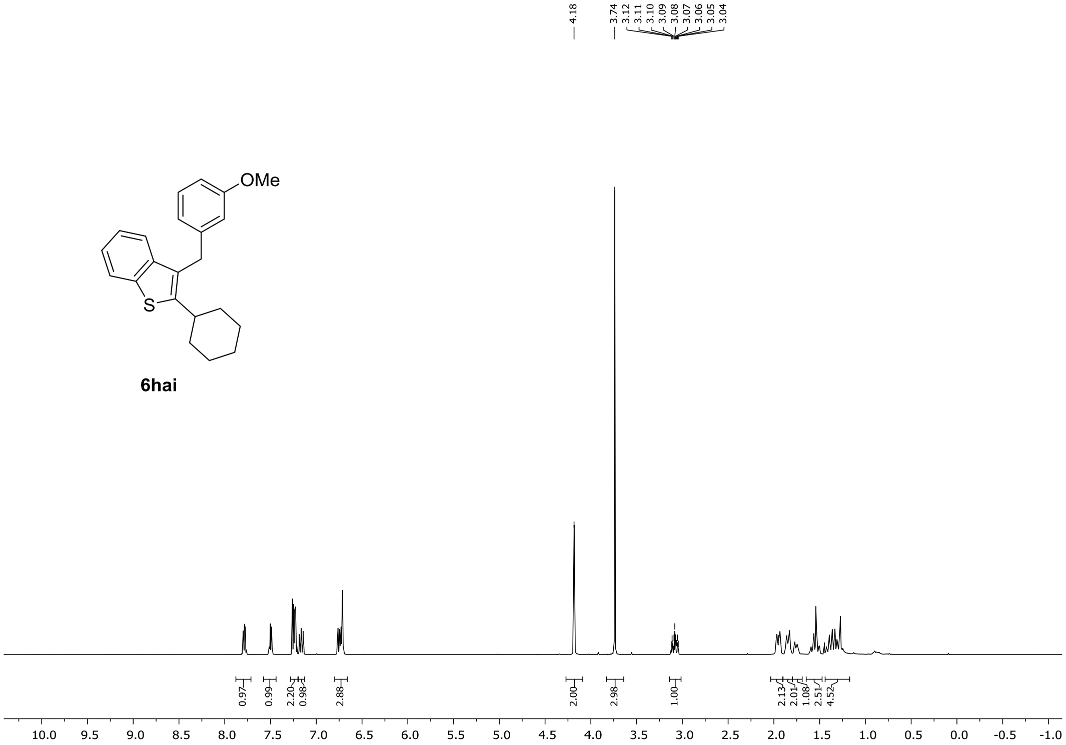

Figure S91: ^{13}C{^{1}H} NMR of **6hai** (101 MHz, $CDCl_3$)

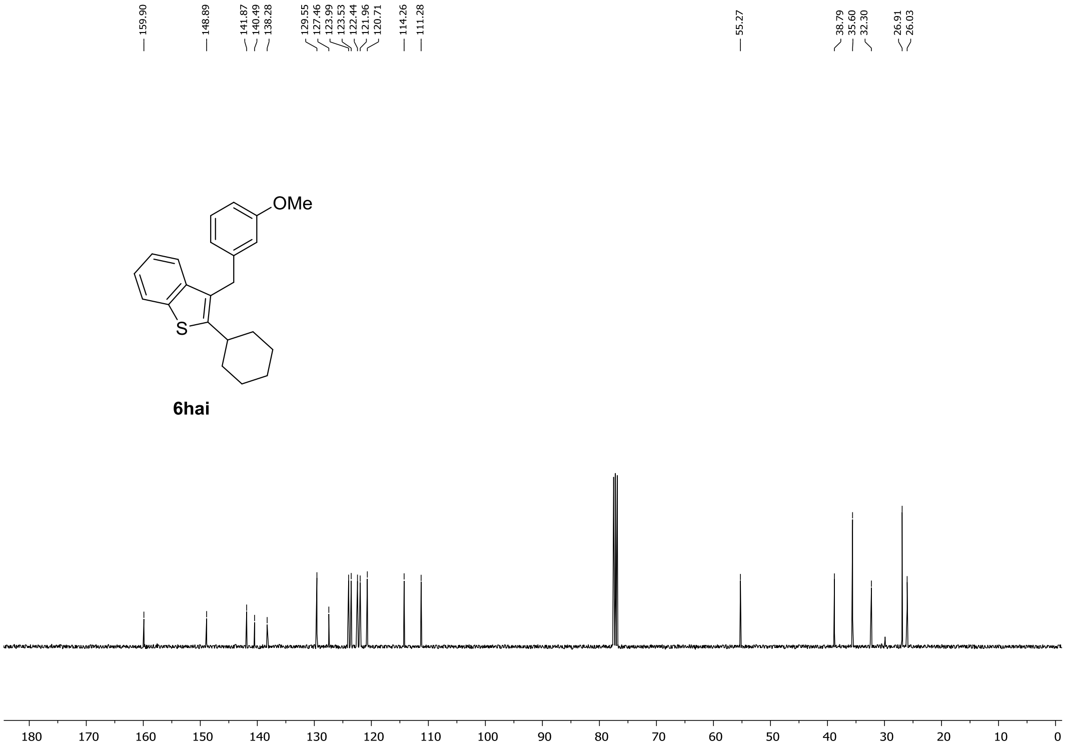

Figure S92: ^{1}H NMR of **(*S*)-6iaa)** (400 MHz, $CDCl_3$)

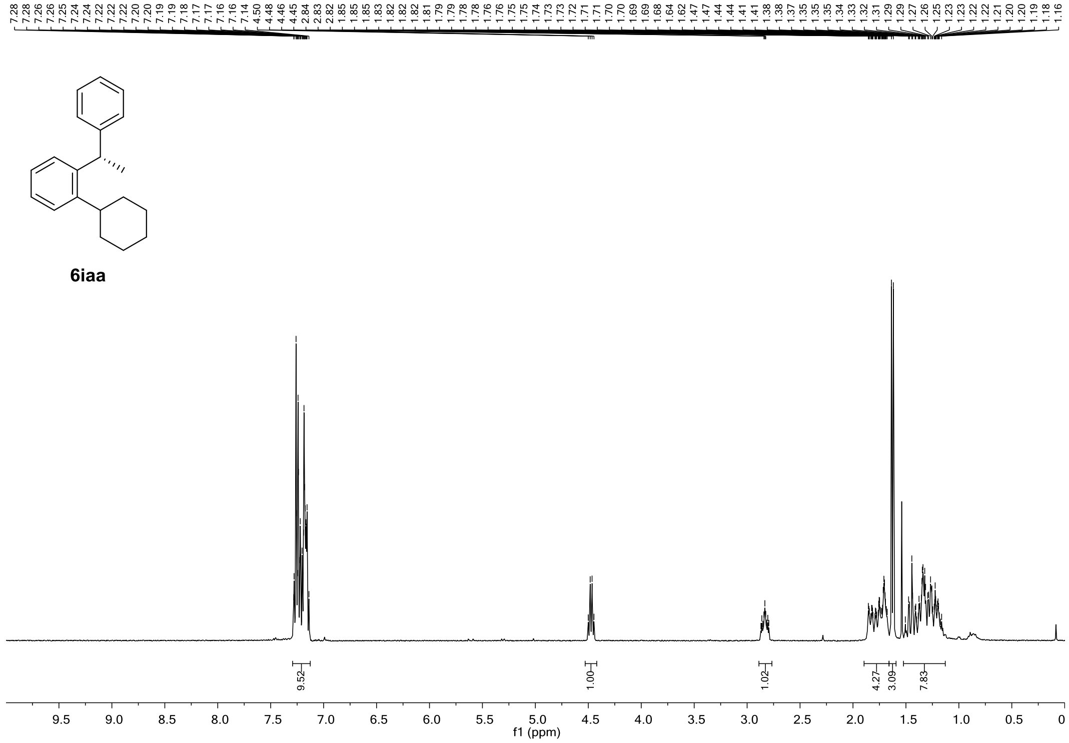

Figure S93: ^{13}C{^{1}H} NMR of **(*S*)-6iaa)** (125 MHz, $CDCl_3$)

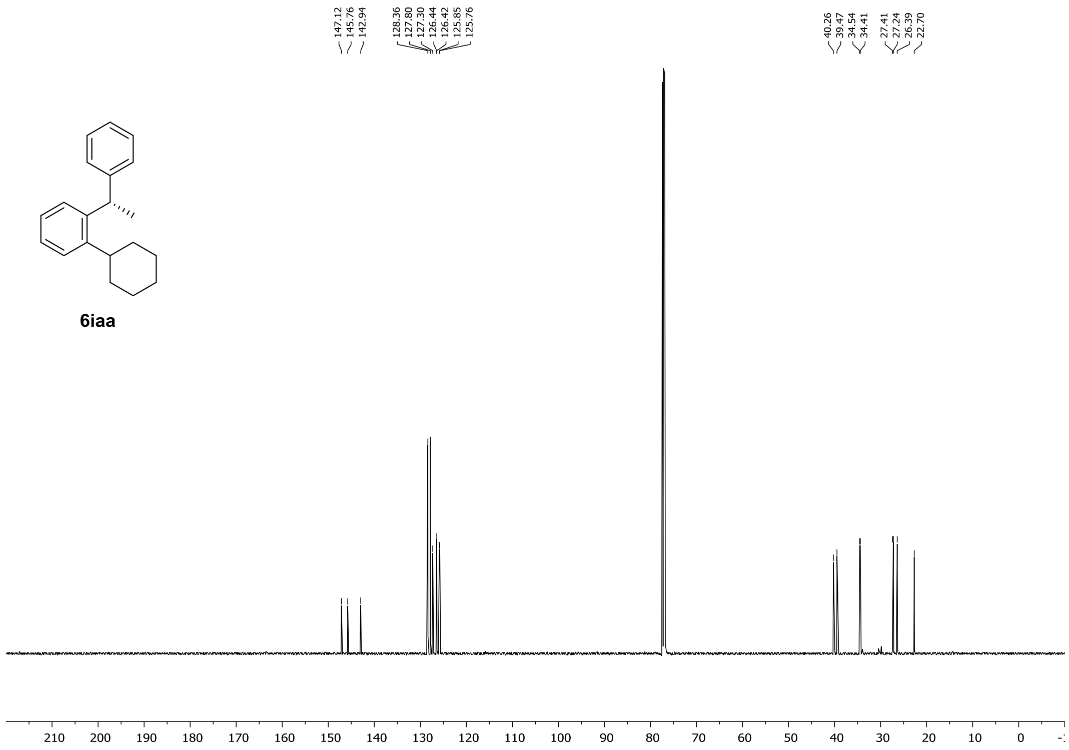

Figure S94: ^{1}H NMR of **6ima** (400 MHz, $CDCl_3$)

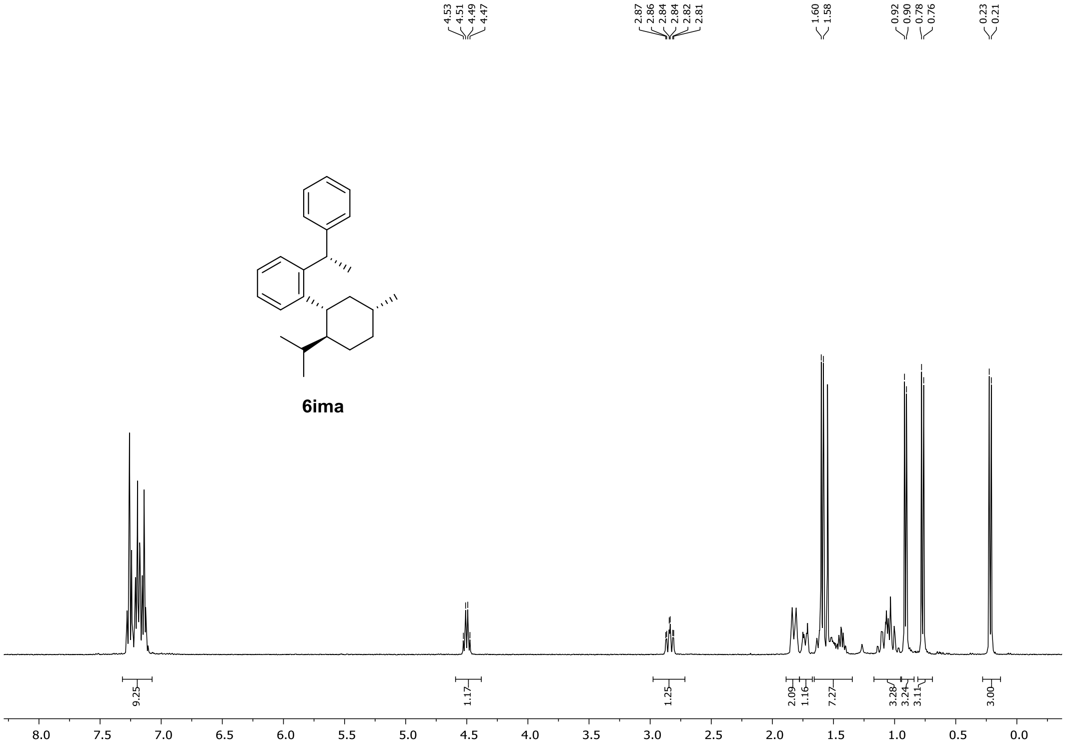

Figure S95: ^{13}C{^{1}H} NMR of **6ima** (101 MHz, $CDCl_3$)

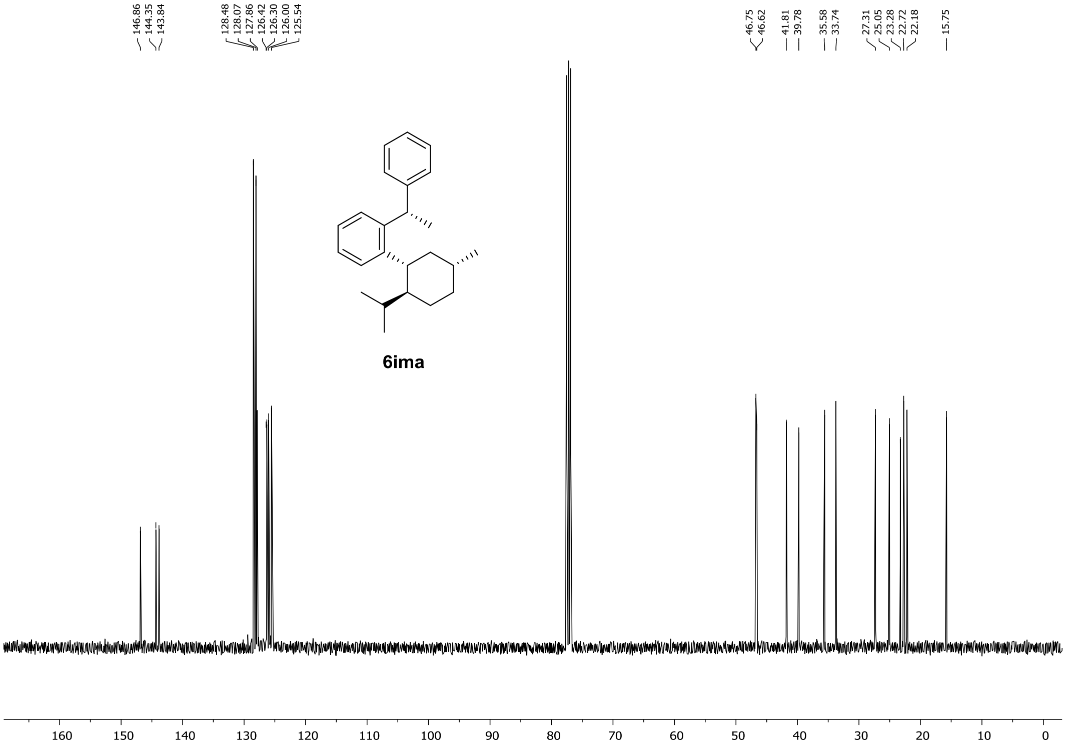

Figure S96: ^{1}H NMR of **6ima’** (400 MHz, $CDCl_3$)

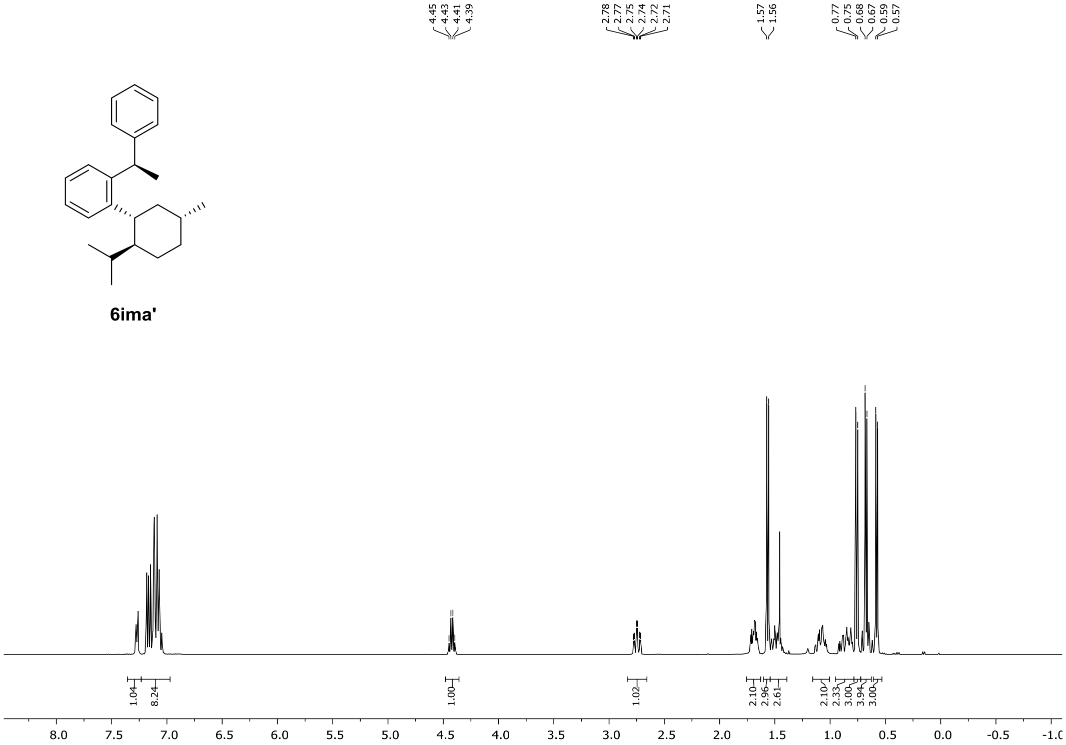

Figure S97: $^{13}C\{^{1}H\}$ NMR of **6ima’** (101 MHz, $CDCl_3$)

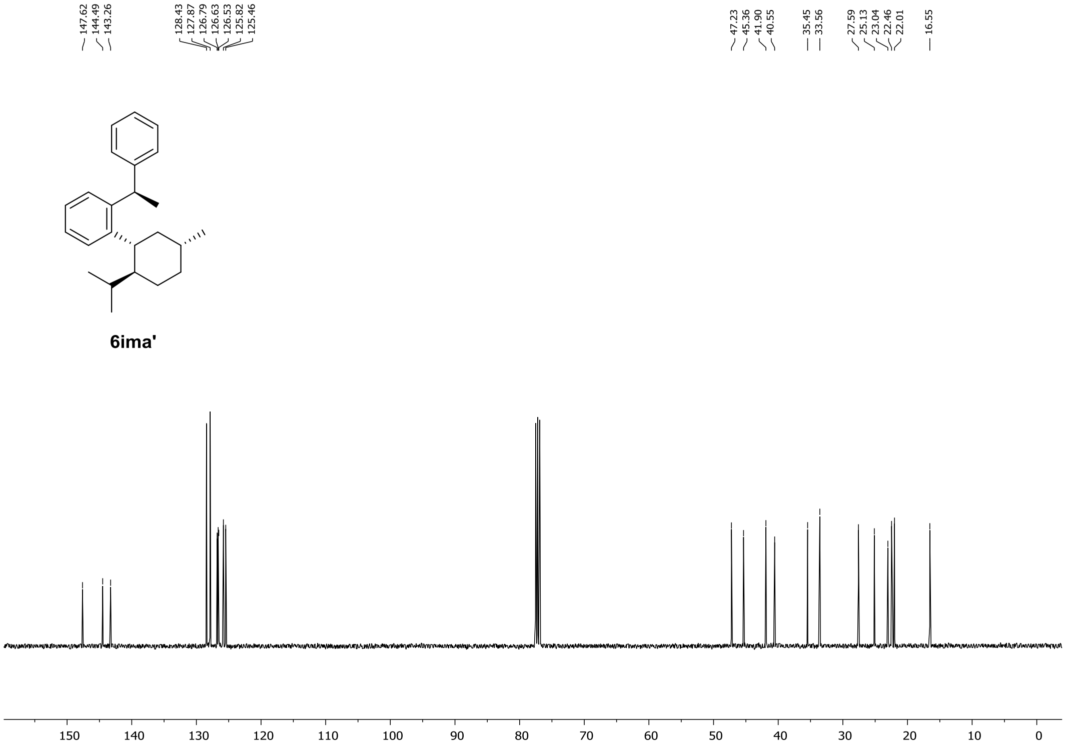

Figure S98: ^{1}H NMR of **6ina** (400 MHz, $CDCl_3$)

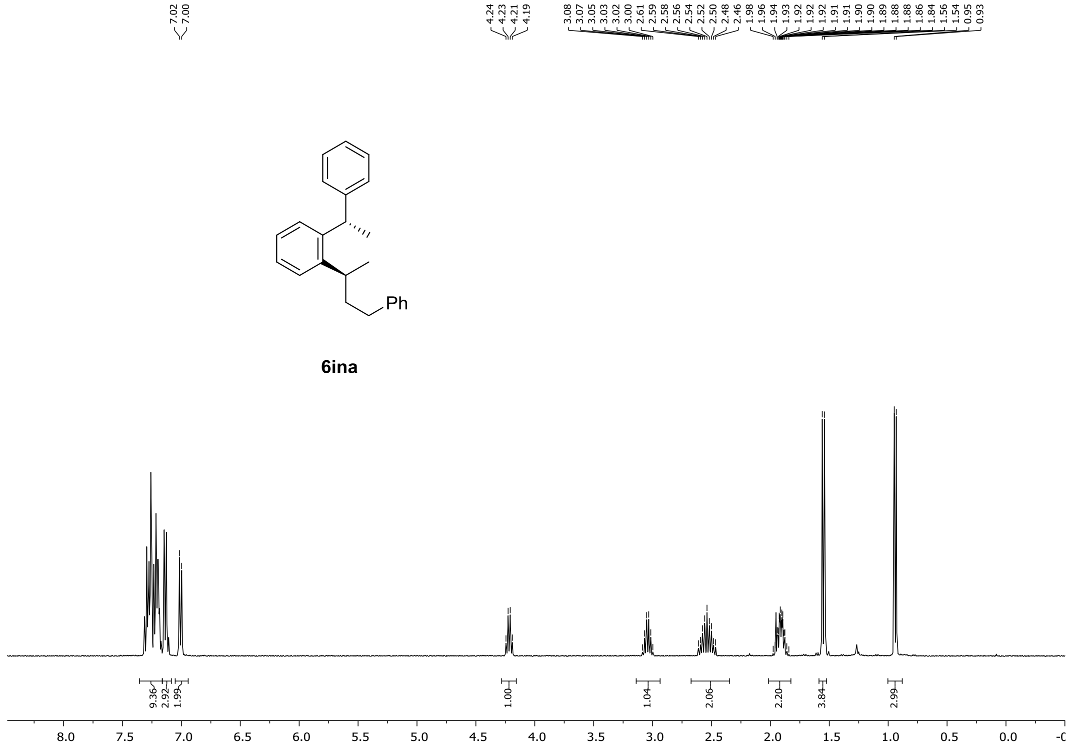

Figure S99: ^{13}C{^{1}H} NMR of **6ina** (101 MHz, $CDCl_3$)

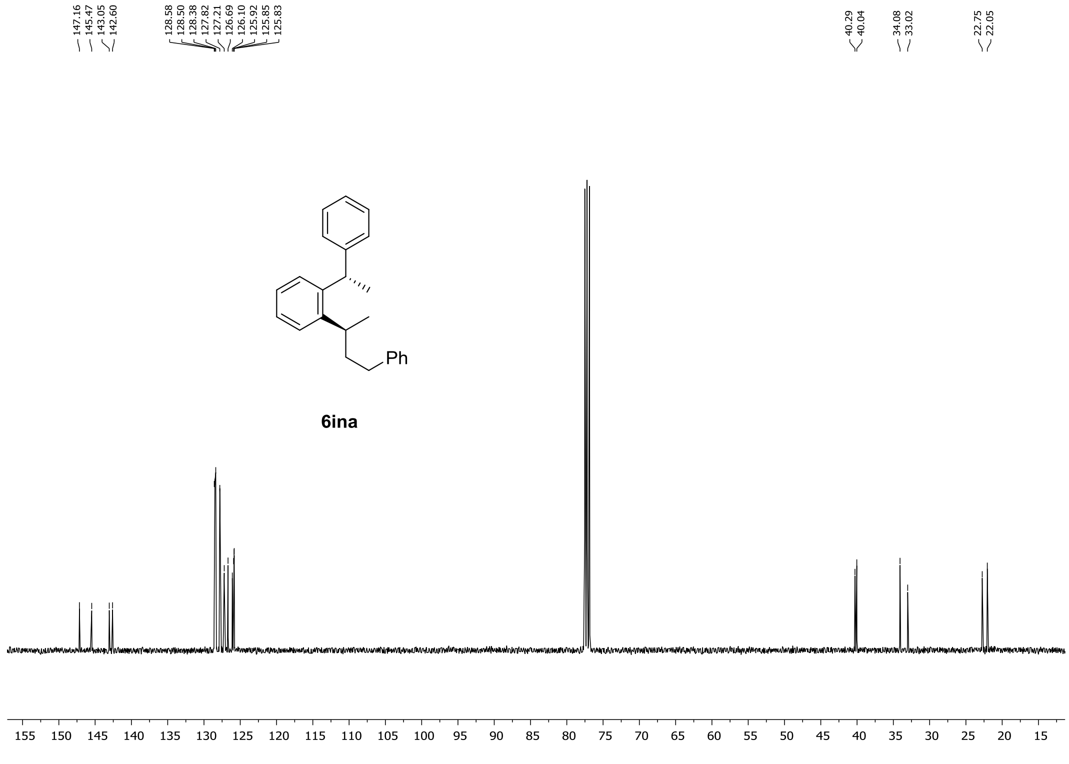

Figure S100: ^{1}H NMR of **6ina’** (400 MHz, $CDCl_3$)

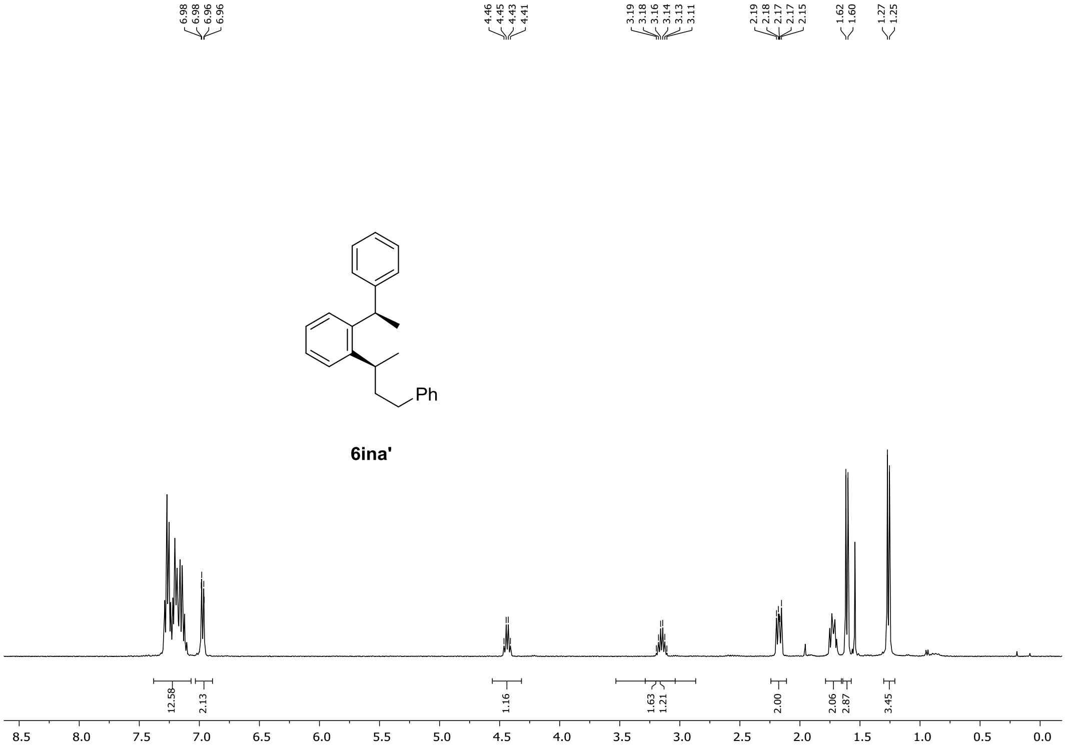

Figure S101: $^{13}C\{^1H\}$ NMR of **6ina’** (101 MHz, $CDCl_3$)

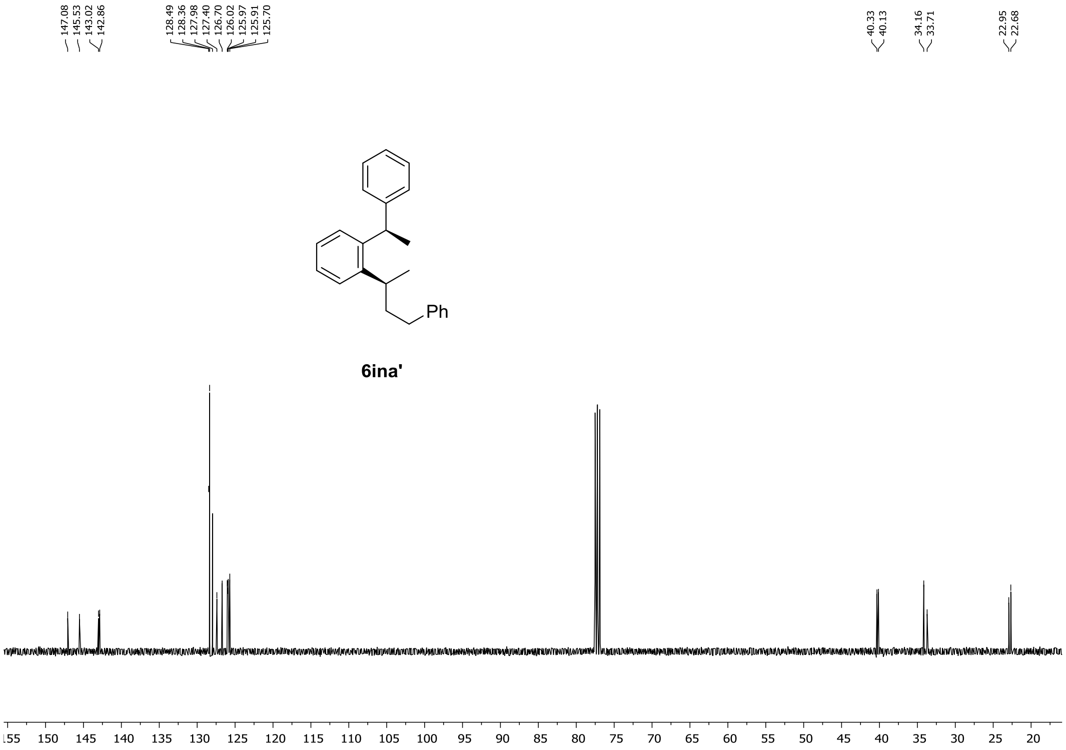

Supplement: Supplementary file 1 — Supplementary [file ANIE-58-1366-s001.pdf]
